# The ecosystem traits index is proposed as a composite index of ecosystem robustness for use in marine resource management

**DOI:** 10.1038/s41598-025-15322-z

**Published:** 2025-09-02

**Authors:** E. A. Fulton, K. Sainsbury

**Affiliations:** 1 CSIRO Environment, Hobart, TAS Australia; 2 Centre for Marine Socioecology, University of Tasmania, Hobart, Australia; 3 Institute Marine and Antarctic Studies, University of Tasmania, Hobart, Australia

**Keywords:** Environmental sciences, Ecological networks, Ecosystem services

## Abstract

Ecosystem structure and function are seldom represented in ecological indicators used for resource management and conservation, despite their central position in stated objectives of international agreements and national policy on maintaining and conserving global ecosystems. Network theory provides capacity to change this situation. Inspired by the use of composite indicators in emergency warning, planning systems and in network analyses in many other applications, we show that a small suite of network-based indicators can provide a practical basis for measuring ecosystem structure in fisheries management. We suggest an Ecosystem Traits Index (ETI) that provides a rating of the combined ecosystem state and structural integrity. This index combines information captured by: the Hub index, which identifies species critical to system function; Gao’s resilience score, which provides a measure of system resilience due to the density of connections and flow patterns in the ecosystem’s food web; and the “Green Band” index (which measures pressure on ecosystem structure due to the mortality from human activities such as harvesting). Applying these indices to marine ecosystems of very different types showed that the combination of indicators is informative in each case and that each ecosystem’s unique state results from fishing pressure, environmental change, and inherent ecosystem structural robustness. Simulation-based tests demonstrated the indicators rapidly respond to, and consistently reflect, ecosystem state changes across marine ecosystem types, though they cannot distinguish the effects of individual stressors such as fishing mortality, habitat modification, climate or other environmental changes. We have focused on fisheries because fishery indicators should, in principle, have utility across any form of marine ecosystem pressure.

**Supplementary Information:**

The online version contains supplementary material available at 10.1038/s41598-025-15322-z.

## Introduction

The restructuring or rebuilding of exploited ecosystems is a key motivator for taking an Ecosystem Approach to Fisheries (EAF)^1^, or Ecosystem-Based Fisheries Management (EBFM)^2,3^. These and similar approaches are included in international fishery agreements (e.g. 1992 UN Fish Stocks Agreement (UN resolution 47/192 (1992)) and increasingly enshrined in national fisheries Acts or regional agreements^4–6^. Since 1995, biological conventions, agreements and policies also include ecosystem components^7^, such as the CBD’s Ecosystem Approach, which calls for explicit consideration and conservation of ecosystem structure and functioning, as does the FAO’s ecosystem approach^1^. So structural indicators are good candidates for use in an ecosystem approach.

Currently, the majority of ecological indicators proposed in the literature for marine ecosystems track the status and trends in biomass or abundance of specific species or species groups^8,9^. This is also true of lists of indicators tracked by the International Council for Exploration of the Sea (ICES), Helsinki Commission (HELCOM) and Oslo and Paris Conventions (OSPAR). This is because data on marine ecosystem function is limited, except around trophic relationships^10^ and habitat dependence in particular system types such as coral reefs or kelp forests^11–15^. Modelling^8^ and field ecology^16,17^ have shown that community or ecosystem structure and function can be inferred from the abundance of species in the system. However, relationships are not always straightforward^18^. Moreover, communicating the combined state of large numbers of abundance indices so as to encapsulate entire ecosystems can be quite challenging, especially within management decision making processes. Climate change is also modifying the relative abundance of species, further complicating interpretations. Consequently, finding a practical means to directly measure and consider ecosystem structure or function is beneficial to ecosystem-based management approaches. Between ecosystem structure and ecosystem function it is much easier to track marine ecosystem structure because it relates directly to biodiversity, trophic and habitat dependencies and it overlaps with data used for many condition indices.

The lack of data on ecosystem function, combined with a desire to provide management-relevant indicators, resulted in the focus here on marine ecosystem structure as a means for operationalising EBFM. This focus on ecosystem structure assumes that healthy structure implies healthy function. While healthy ecosystem structure does not guarantee healthy ecosystem function, a degraded ecosystem structure ultimately degrades ecosystem function. Also, it has been shown that vertical food web diversity (structure) in combination with trait space (functional diversity) can dictate ecosystem function^19^. Further, structural indicators can also capture ecosystem scale features not possible with other types of indicator. Specifically, size-based indicators, which have been used as a proxy for function in marine systems^20^, as many marine ecological processes are related to size^21^. Thus, by using network theory to integrate aspects of ecosystem structure and function into a single index, the method described in this paper can robustly interpret marine ecosystem health/integrity while implying function is also maintained.

Network theory is a very promising way to consider and measure ecosystem structure (a glossary of useful terms for network theory is provided in Supplementary Materials). Networks (e.g. food webs, electricity grids, trade links, road networks etc.) consist of interacting nodes (e.g. species, groups, habitats) connected by edges (e.g. energy transfers, habitat use). Network theory quantifies network structure^22,23^ and can identify critical nodes and properties indicating structural integrity and resilience^24,25^. Within the sub-field of network ecology, these methods document ecological processes^26^, biodiversity, the role of particular species (or functional traits) in ecosystem structure and function, and long-term dynamics and stability^23^. While network summary statistics have existed for many years^27^, recent advances – such as graphical and interpretive motifs^26,28,29^ – make the information more accessible for decision-makers.

The system-specific nature of ecosystems makes finding universally applicable indicators challenging, especially given the diversity of socioecological objectives and contexts those ecosystems are embedded in. The desire to find informative structural and functional indicators for practical ecosystem-based fisheries management, motivated the application and testing of a small set of structural indicators that can be tracked individually and that summarise different aspects of ecosystem structure. The indicators can also be combined as a composite indicator to act as a general index for changes in ecosystem structure and function. Use of the indicators as a warning indicator was heavily inspired by Australia’s fire danger rating system^30^ and we used them to improve communication about the status of the ecosystem’s “integrity”.

## Methods

### Ecosystem traits Index (ETI) – component parts and final index

We propose that summarising network structure and function involves representing three complementary dimensions of ecosystem structure (see the glossary in the supplementary materials for a description of terms):

(i) Topology—how the ecosystem is structured, and which aspects are most important to its integrity (e.g. Hub index^31^) and so should receive additional attention (higher weighting) when reporting on status of ecosystem components;
(ii) Structural resilience (Gao’s resilience) is a more direct measure of health or integrity, which in the marine context is a proxy for an ecosystem’s capacity to maintain overall function given its current state; and
(iii) Distortive pressure results from the mortality that is applied to the ecosystem structure by human activities and is measured here by the Green Band^32^.

These individual aspects can be tracked individually, and they can be combined to create a new composite index of ecosystem traits and integrity (health) referred to here as the Ecosystem Traits Index (ETI). The ETI links these structural aspects of ecosystems with the relative state (relative biomass or abundance) of the ecosystem’s components (the individual species, habitats and functional groups). These indicators draw on ecological principles, including macroecology, and use large scale marine ecosystem patterns to track ecosystem change. However, these indicators (and the names used for them) have been derived, and used, independently of the rich body of terrestrial work on macroecology and functional traits at community scales^33^. Consequently, the term Ecosystem Traits Index can cause confusion because it may be interpreted differently in different ecological disciplines and anyone looking to use the ETI should be aware of this when introducing it to new audiences.

### Topology – hub index

Ecological Network Analysis describes how the number of nodes, density, and patterns of connection determine a network’s structural complexity and sub-webs contributions^23^. We have adopted the previously described Hub Index^31^ as our means of tracking topology, particularly the state of key structural components (and thus gross function) of an ecosystem. The Hub Index is used to identify species that receive high weightings in the calculation of the ETI. The topological importance of Hub species to the ecosystem is reflected in the weightings given both in terms of the relative status or depletion, and the distortive pressure they are under. This is because the loss of “hub species” disproportionately impacts the ecosystem’s structural integrity^31^, making conserving these species important for maintaining ecosystem structure and function.

Criticality analysis concepts from computer science^34^ and engineering^35–38^ can be used to identify critical “hub species” for food web functioning^31^. Tracking the Hub Index for a species or a functional group in the ecosystem gives insight into their changing relative role over time or space.

Hub species can be identified using a combination of commonly used network indices^31^ – degree (the number of predators and prey a species has in the ecosystem), degree-out (the number of predators the group has, or for a habitat the number of species dependent on that habitat) and PageRank (the importance of the flows through that species in the food web, calculated from the number and strength of links to a node). The “Hub Index” of a node species or functional group is given by:

1 $$Hu{b}_{Index}=\text{min}({R}_{degree},{R}_{degree\_out},{R}_{pageRank})$$

where *R*_*degree*_ is the rank for a species/group based on its degree, *R*_*degree_out*_ the rank for a species/group based on the number of flows out of that group and *R*_*pagerank*_ is the rank for a species/group based on its Pagerank. In each case the value 1 indicates the highest score for a measure. This combination of metrics was used because degree identifies species with many “local” connections that connect across many sub-systems (e.g. top predators that feed across an ecosystem), degree-out captures basal groups that provide inputs to many sub-webs, and the PageRank highlights centrally placed prey species (e.g. forage species) with high global connectance. The species ranked in the top 5% for the network, based on the Hub Index score, are considered “hub species” (see Fulton and Sainsbury^31^). Analyses based on large diet databases indicate that individual hub species persist for long periods, making them important entities in management-relevant ecosystem typology^31^.

### Network resilience – gao’s resilience

A network’s resilience indicates its capacity to handle perturbations while maintaining structure and functions, such as network flow. Gao et al^25^ revealed universal patterns in the resilience of complex systems and provided a method for calculating resilience from network structure. Their analytical framework reduced the behaviour of networks into a single resilience function based on a few robust macroscopic structural properties of network structure^39^, and so allows comparison of ecological networks in both time and space.

Based on Gao et al^25^ we provide an interpretable measure of resilience for marine ecosystems and use it as a proxy for the realised health of an ecosystem’s structural and functional integrity. The simple Resilience Score (*R*) indicates in gross terms how far the current fished system is from potential “collapse”, where we use this term to mean a major change in the structure and function of the ecosystem that may or may not be reversible.

The Resilience Score captures an ecosystem’s stability based on its location in two different dimensions of system structure, which influence energy flow in the system. The first dimension is the network density $\langle s\rangle$, the mean weighted degree of the network, with the weights defined by consumption of the prey by predators. This captures the number of connections in the system, the number of potential pathways there are through the web of connections. The second dimension is the heterogeneity of the flows in the system ($\mathcal{H}$), calculated as the variance of the distribution of the weighted degrees in the network, this captures the diversity of motifs (different ecological processes) present and shows if there is both strong and weak connections in the system. The number and diversity of connections and processes in marine food webs have previously been found to be important to resilience^26,40^.

Each dimension has a boundary between stabile and potentially unstable regions. As described in Gao et al.,^25^ the resilience of a food web is governed by three topological characteristics: symmetry S, the heterogeneity of the flows in the system H and the network density $\langle s\rangle .$ Gao et al.^25^ showed that the specific dynamics of how a network responds to perturbation (e.g. loss of a node or link, pressure on the nodes etc.) are fully accounted for by the *eff*ective state of the system (*β*_*eff*_) in the space of all possible conditions (*β*). They explain that the changing value of *β*_*eff*_ summarises the system state within a universal “resilience function” that is a mathematical construct based on the dynamics of complex systems of many types. Gao et al^25^ define a transition surface of *β*_*eff*_ that separates resilient from non-resilient states, i.e.:

2 $${\beta }_{eff}=\langle s\rangle +S\mathcal{H}=\langle s\rangle +\frac{\langle {s}^{in}{s}^{out}\rangle -\langle {s}^{in}\rangle \langle {s}^{out}\rangle }{{\sigma }_{in}{\sigma }_{out}} \cdot \frac{{\sigma }_{in}{\sigma }_{out}}{\langle s\rangle }$$

$$as \; S = \frac{{\langle s^{{in}} s^{{out}} \rangle - \langle s^{{in}} \rangle \langle s^{{out}} \rangle }}{{\sigma _{{in}} \sigma _{{out}} }} \quad and \quad \mathcal{H}=\frac{{\sigma }_{in}{\sigma }_{out}}{\langle s\rangle }$$

where the chevrons $\langle \rangle$ indicate the mean; *s*^*in*^ and *s*^*out*^ are weighted degrees (in and out) and ${\sigma }_{in}^{2}$ and ${\sigma }_{out}^{2}$ are the variances of the marginal probability density functions across species P(*s*^*in*^) and P(*s*^*out*^), respectively. To convert a binary diet connection matrix to the weighted network for the purposes of calculating the weighted degrees, replace the links with the consumption associated with that predator–prey interaction.

**Fig. 1:**
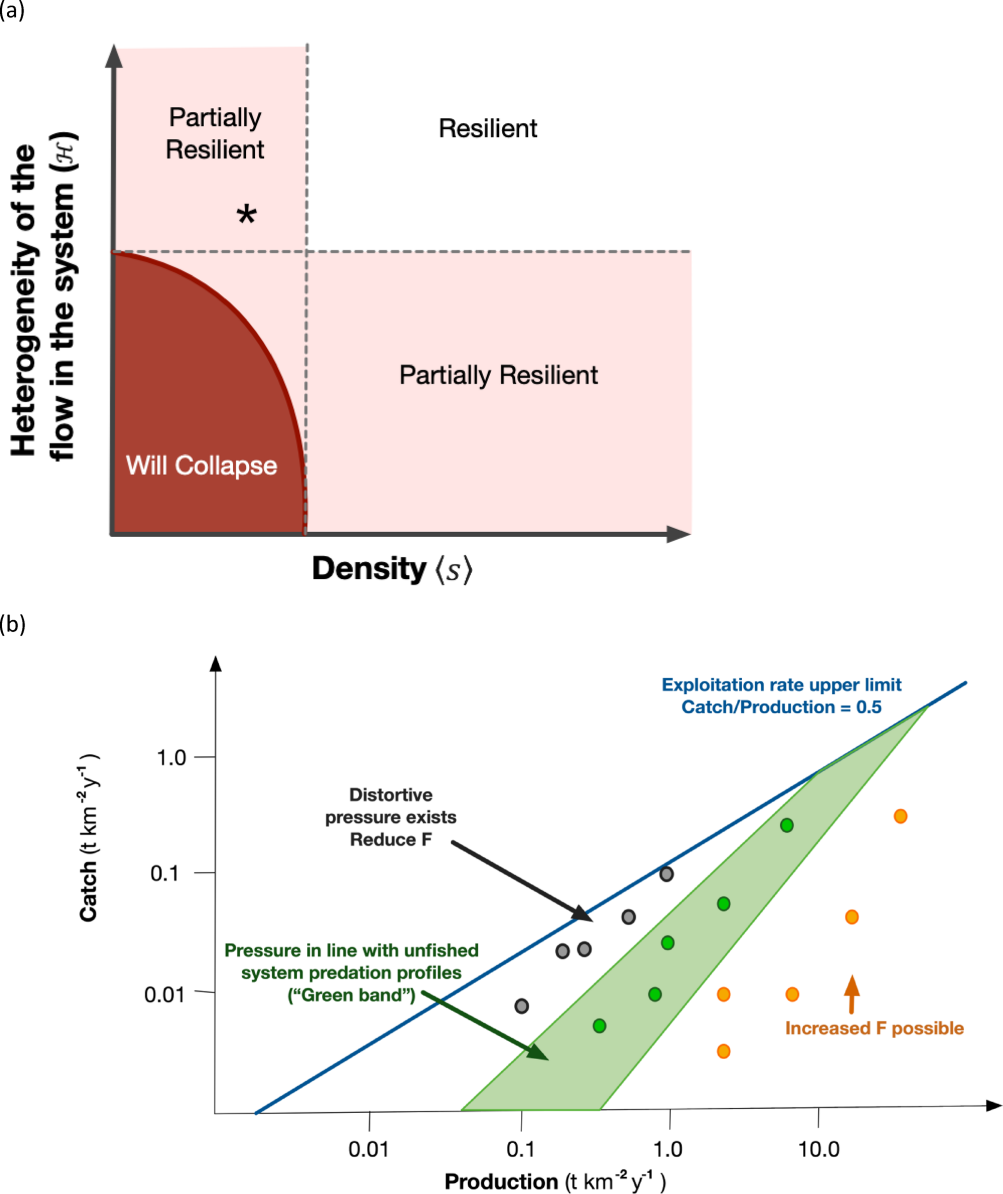
Schematic of components of the ETI: (**a**) Gao’s resilience score used in calculating the ETI, where the position of an ecosystem (for example the spot marked by a * on the plot) determines whether it is resilient in terms of the number (density) and diversity (heterogeneity) of connections in the system; (**b**) “Green Band” plot for looking at distortive pressure on ecosystem structure.

In translating Gao et al.,^25^ for our purposes we took the points at which Gao’s transition surface *β*_*eff*_ (hereafter referred to as the resilience frontier) intercepts the axes of the $(\mathcal{H},$ $\langle s\rangle$) space to define stability boundaries for those dimensions, as shown in Fig. 1a. Interpretation of the Resilience Score is then based on where the ecosystem sits in $(\mathcal{H},$ $\langle s\rangle$) space versus the *β*_*eff*_ surface. Points in the upper right in Fig. 1a are considered completely resilient because they are above the thresholds of stability of both dimensions. Points in the light coloured zone are only partially resilient, as they have passed the boundary in at least one or other dimension but have not yet dropped below the transition surface *β*_*eff*_. Points in the dark coloured bottom left of Fig. 1a are below the transition surface *β*_*eff*_ and so are non-resilient. A perturbation in a partially resilient system could result in the system becoming non-resilient. For example, a partially resilient system with a low < s > score (such as the * on the plot) could fall into the non-resilient zone if basal productivity and hence $\mathcal{H}$ decreased.

To calculate the scalar value of *R* used in calculating the ETI, fully resilient systems are given a resilience score of 1.0, partially resilient systems score 0.8 and non-resilient systems 0.5. These values were chosen for use in the ETI to reflect that resilience is one component of the system state that amplifies the implications of system structure and condition captured by the other terms of the ETI. A fully resilient system is given a value of *R* = 1.0, with lower resilience reflected by progressively lower *R* values, reflecting how a loss of structural resilience amplifies any risks associated with degraded compositional state. The exact values used for *R* are somewhat arbitrary and could be more made more or less precautionary. In the ETI as presented in this paper we are using scores tuned so that final ETI ranks calculated for simulated ecosystem states matched easily to the speed of system responses, level of depletion and rate of rebuilding. However, a more conservative scoring system could use 1.0, 0.5 and 0.25 respectively for these scores.

### Distortive pressure—the green band

Recent papers^32,41^ describe a method relating catch to production. By defining an acceptable band of exploitation pressure, the Green Band defines an acceptable band of exploitation mortality and indicates whether an ecosystem is under distortive patterns of fishing mortality that will ultimately undermine ecosystem structure. We use the Green Band to reflect the sustainability of fishing mortality on a system.

The Green Band is a comparison between current patterns of mortality due to human activity versus patterns of mortality in undisturbed systems. The approach is based on the premise that an ecosystem can sustain its unfished pressure (mortality) profile, as represented by the biomass-production profile. This profile is then used to define a range of acceptable profiles of mortality in a perturbed (exploited) system – the Green Band. By comparing the catch of a species to the Green Band (Fig. 1b) it is possible to determine whether species are being harvested at a rate aligned with that of the natural system (i.e. sit within the Green Band), are being structurally overfished (sit above the Green Band) or have scope for an increase in exploitation (sit below the Green Band).

Heath et al.^32^ describes the derivation of the method in more detail, but the basic steps are:

1. Calculate the unfished profile—a linear regression of biomass against production in log–log space, where the species/habitats/functional groups in the system are the data points. As this step will most likely be conducted using model-based information, the resolution of habitats, species or functional groups will depend on the model. Conversely the model must meet the resolution and intentions of the analysis. Testing described in Fulton et al.^40^ indicates that method is reasonably robust to taxonomic resolution if model building guidelines^42–44^ are followed.
2. On a plot of catch of the fishery (whether it is landed or not) vs production (*P*), the left bound of the “acceptable” pressure levels in the fished system is given by:
  3a $${Y}_{left\_bound}=\text{min}(0.5P,{P}^{1+a})$$
  where *a* is the slope of the regression line in step 1. The right bound is given by:
  3b $${Y}_{right\_bound}=\text{min}(0.5P,{\delta P}^{1+a})$$
  where *δ* is a scalar based on the variance in the points around the regression line in step 1 (i.e. spread of points either side) and typically has a value of approximately 0.01.
3. Species are plotted in the catch vs production space. Species that fall within the Green Band are being harvested at a rate aligned with that of the natural system, those above the Green Band are being structurally overfished and those sitting below the band have scope for an increase in exploitation. The location of species in relation to the Green Band can be tracked through time as a check on the performance of fisheries and their management. The further that a group is above the Green Band the more urgent the need for management action. Similarly, trajectories that begin within the Green Band but track toward the upper band are an early warning that management change is needed to avoid the risk of undesirable ecosystem pressure.

### Composite Index—ETI

While the indices above can be tracked individually, it is often useful to reduce complex information into an easily interpretable composite indicator. In this case reflecting distortive pressures degrading ecosystem structure, while avoiding misdiagnosing situations where high fishing mortality is applied in a “natural distribution” (i.e. mortality ratios between species similar to that of the unfished ecosystem) reduces individual species to low levels.

The ETI combines information captured in the other indices to provide an index of ecosystem integrity to guide management of system robustness and the fishery more generally. An early form of this index was previously used to study fishing in the Gulf of Thailand^45^, but here the methods are described more fully and its management efficacy tested.

**Fig. 2:**
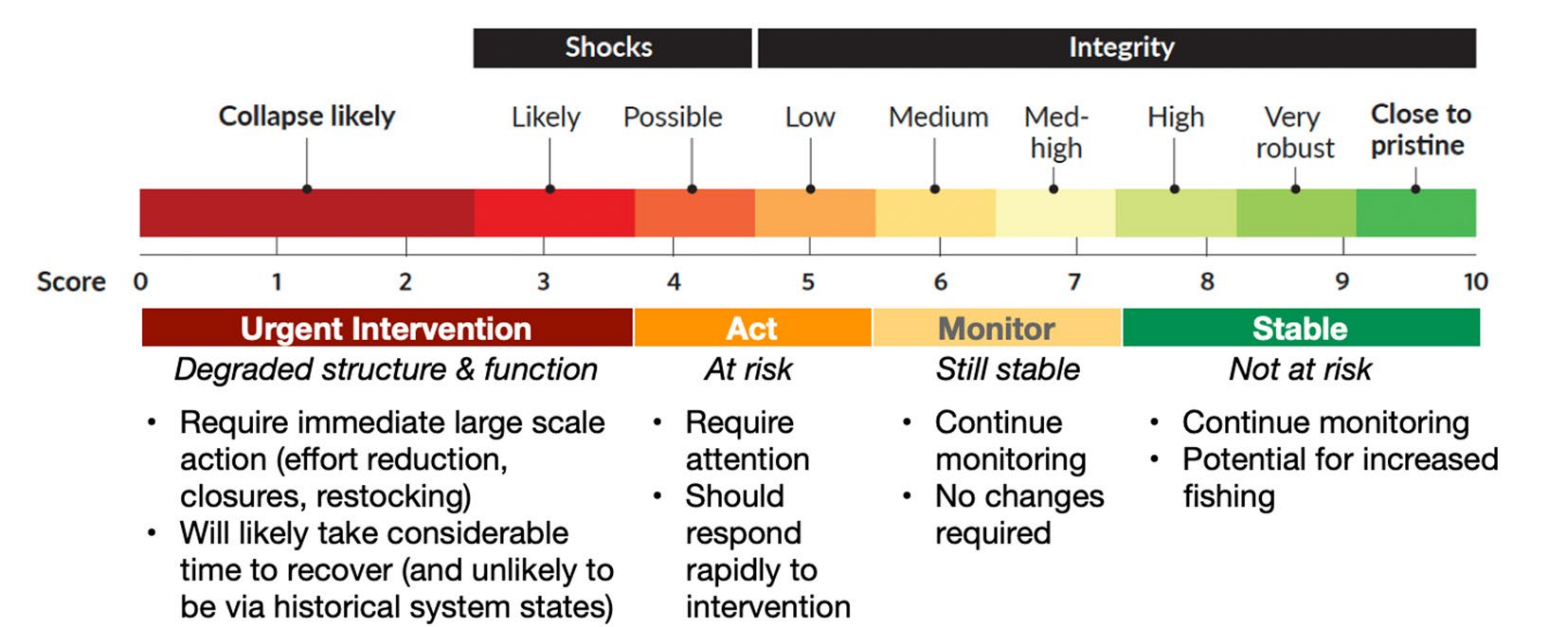
Schematic of the ETI ranks and their related management implications.

Preliminary testing of how best to communicate ETI (as described in the methods and results below) suggests that when tracking the ETI through time (e.g. as a time series) it was effective to plot the raw score but with colouring based on the final qualitative ranking (Fig. 2). At present the ETI has 10 possible ratings.

The steps to construct the ETI are:

1. Classifying each species/group using the scheme in Table S1.
2. Using the qualitative scheme in Table S2, rate each species’ position versus the Green Band and its relative biomass vs the target relative biomass ranges listed in Table S1. Note that the target relative biomass scoring was initially based on targets from fisheries and marine conservation literature, but was expanded to a broader range of values to avoid sharp discontinuities that could arise if single point targets were used where there was observational uncertainty or fluctuation due to interannual environmental variation. The biomass bands are intentionally set conservatively (ranging higher than point targets from literature). These levels allow for some impacts of fishing while also maintaining long-term ecosystem structure and function in ecosystem models used here in concept testing (determined by running simulations at different levels of depletion as described under test 1 below). Alternatively, less conservative and less constraining values could be used, such as limit reference points.
3. Tabulate, by populating the matrix in Table S3, the number of groups per combination of Green Band-relative biomass.
4. Calculate the final value of the ETI using the values from step 3 and the Gao’s resilience score:
  4 $$ETI=R\left(\sum_{j}{W}_{j}\left[\sum_{i}\frac{{\kappa }_{i}{N}_{i,j}}{{N}_{j}}\right]\right)$$

where *i* is the Green Band-relative biomass (GB-RelB) combination (the row of Table S3); *j* is the species classes (columns vulnerable to hub in Table S3); *N*_*ij*_ are the values in the cells of the matrix in Table S3; *N*_*j*_ is the total number of species in class *j*; κ_*j*_ is the combination score (a scalar of how healthy that combination of distortive pressure and relative biomass is, provided for reference in Table S3); *Wj* is the weighting given to species class *j* (see Table S1); and *R* is the Gao’s resilience score. The resulting ETI can be used as is or can be converted into a simple qualitative ranking using Table S4.

### Testing the ETI

The utility and responsiveness of the ETI was explored using model-generated data for.

i. “*Test 1”:* a range of levels of fishing pressure in an Ecopath with Ecosim (EwE^46^) model of southeast Australia, this provides a simple exploration of how the ETI captures different levels of system degradation;
ii. *“Test 2:* historical forcing applied to EwE models for four case study regions—Kerala (south western India)^47^, eastern Bering Sea^48^ (Alaska, USA), southeast Australia^49^ and north-central Chile (unpublished model developed by IFOP, Chile); and
iii. *“Test 3”*: climate and marine heatwave forcing scenarios applied to each of the four case study models.

Full details of the structures and parameters in the Ecopath models used to test the ETI are provided in the supplementary materials (Tables S15–S29).

National fisheries data provided by the national researchers participating in the Lenfest-CSIRO working group (as noted in the Acknowledgements of this paper) were used to update and calibrate the models on time series through to 2017–2018. 2017/18 is a common end to data made available to the project and later data isn’t necessary for the proof of concept provided here.

The model-based tests allowed examination of how robust the methods were to ecosystem type, and to real world levels of noise, trends and data availability. It was also possible to judge responsiveness (whether an indicator reflects change quickly), sensitivity (whether an indicator provides a clear and interpretable signal of real change), and specificity (whether an indicator responds only to change due to a specific stressor), or reliability, of the indicators under conditions of interest to managers such as different levels of fishing pressure and varied environmental forcing. Comparing the magnitude and pattern of results across the different simulations and different model ecosystems allowed examination of whether similar forms of response were driven by common drivers in all systems or whether there would be system specificity (a common issue with marine ecosystem indicators^50^). It was also used to test whether the indicators responded quickly and clearly to both system degradation, and also to recovery. Tests 1 provides insight on sensitivity and responsiveness, while tests 2 and 3 both inform on all three aspects, but particularly specificity and responses under compound drivers (fishing and climate change).

The ecosystem models were used to synthesise system information and to generate values for production, biomass, catch and flows needed to calculate the contributing parts of the ETI. Independent observations of consumption, production and flow data could be substituted for these values if they are available. Key aspects of the modelling include:

(i) *Simple fishing pressure forcing*: two alternative parameterisations of an EwE model for southeast Australia^49^ were forced with fishing pressure set at gF_MMSY_ where *F*_MMSY_ is the multispecies maximum sustainable yield exploitation rate (with full ecosystem compensation, that is allowing for all species in the system to respond dynamically to fishing of any other species in the system) estimated using the inbuilt EwE routine^51^ and g ~ [0, 0.02, 0.05, 0.1, 0.2, 0.5, 0.65, 1.0, 1.5, 2.0, 5.0, 10.0, 20.0]. The alternative EwE parameterisations reflect different strengths for trophic connections within the Australian ecosystem, based on observational data from the region and that the ecosystem is in a global warming hotspot^52^ that has changed over a 20-year period (see supplementary materials for more details on these parameterisations).
(ii) *Case study regions:* The regions were selected for their strongly contrasting ecological, economic and social structures, as they differ in their ecosystem structure and function, but also in their management contexts:
  Northern-central Chile: a highly productive upwelling system, primarily fished by a quasi-industrial artisanal fishery and formal management structure that has historically taken a species-by-species approach to define fisheries.
  Kerala, India: a highly seasonal, biodiverse and productive tropical system that has long been fished by many thousands of fishers using a range of gears, from very simple traditional gears through to highly mechanised offshore operations, and where there has been some overfishing historically and where fisheries management arrangements focus on effort control measures.
  Southeast Australia: a low productivity but highly diverse temperate region targeted by recreational and commercial gears of many types, with the latter under formal individual transferrable quota and other management controls, where there has been over-exploitation of some stocks historically and broader ecosystem management includes an ecological risk assessment approach.
  Eastern Bering Sea, USA: a highly productive sub-Arctic shallow shelf extending 500 km from coastal Alaska that has high catches, a management system that uses individual species catch limits and implements a cap on exploitation at the ecosystem level.

Such strongly contrasting systems were chosen because if the ETI proved possible and useful in these systems it was more likely to be useful generally.

For each case study region historical (see Table S5) and three projected time series were run. All the projections were run under CMIP6 SSP3-7.0 forcing^53,54^, which represents the medium to high end of plausible future climate forcing pathways. While the current emission reduction trajectory and technological advances mean the world is arguably closer to the CMIP6 SSP2-4.5 (a medium forcing scenario^55^), there is little difference in forcing temperatures over the 20-year projection period used here. The first climate projection was run “as is” from the CMIP6 repository, using the temperature increases and productivity shifts. An additional climate change scenario with marine heatwaves was also run – with extreme events imposed on top of the time series used in the first climate projection. These events were characterised from past events ^56–59^ and information available from https://www.marineheatwaves.org. These two sets of projections were run with fishing pressure held at historical (2018–2017) levels to see how the ETI reflected system structure under conditions that are anticipated to stress systems and potentially lead to ecosystem tipping points. The third scenario run for each case study model was a reduction in fishing pressure run with the CMIP6 SSP3-7.0 forcing (with no additional extreme events), but with 50% reduction in commercial fishing pressure. This was used to explore the capacity of the ETI to reflect system responses to reductions in cumulative pressure.

The steps in calculating the ETI involve calculating the hub species and then the constituent indices that combine to give the ETI. As the hub species and Green Band results have been the focus of independent dedicated publications on those individual topics, we do not provide extensive details that are not directly informative for this ETI analysis, though short summarises are presented in the main results section to help readers understand how the suite of species capture important information around the dynamics if ecosystems and their structure. Readers interested in the detail, descriptions of the Green Band results for tests 2 and 3 in each case study region are provided in Fulton et al^41^, while the hub species identification analyses in those locations are in Fulton and Sainsbury^31^. The Gao’s resilience score for each system and final ETI have not been previously described are presented here in detail.

## Results

### Test 1: Varying fishing pressure

#### Hub species

The hub species are consistent across many of the simulations run with differing levels of fishing pressure for the two EwE model variants considered in this test (Table S6). In particular, squid, mesopelagics, toothed whales and euphausiids are consistently hub species under parametrisation 1, while only toothed whales and euphausiids are under parameterisation 2. Of these groups mesopelagics, toothed whales and euphausiids move between being marginal and more clearly important hub species as fishing pressure increases (especially for parameterisation 2).

There were step changes in the identity of hub species as the system restructured under fishing pressure of 0.65–1.5xF_MMSY_ in both cases. Sharks (pelagic and demersal) were hub species before this point, with demersal sharks persisting longer as fishing pressure rose. The greatest changes in the hub species occurred as the system became highly depleted, as the most common hub species were lost from the system.

#### Green band

The Green Band results show a relatively smooth transition as fishing pressure increases, with the majority of species groups receiving a “Fail” score under extreme fishing pressure (Table S2). For parameterisation 2 nearly all species receive a “Fail” score by the 1.5xF_MMSY_ level of fishing pressure (Table S7), whereas for parameterisation 1 there remained a small number of species groups receiving a “Light” or “Acceptable” score even amongst the most extreme fishing pressure levels (Table S7). In general, as fishing pressure increases, more species transition from “Light” to “Acceptable” to “Fail”. However, some reversals or non-linearities occur as the system simplifies under very high fishing pressure, because as species are released from predation pressure or competition their apparent productivity to the fishery expands. For example, under parameterisation 1 at 2F_MMSY_ there is only 1 of the vulnerable species groups (Rays) rated as acceptable, with the other 4 in a Fail state. However, as F increases to 5F_MMSY_, the Rays only remain “acceptable” because of a substantial increase in productivity due to a release from predation pressure. At the same time seals drop to a “light” rating, as a result of a large increase in production due to both predation and competition being released.

The situation for hub species is complicated further because the hub species can change as the ecosystem changes (as noted in Table S6), though the consistency of several groups (bolded in Table S6) moderates this.

#### Gao’s resilience

**Fig. 3:**
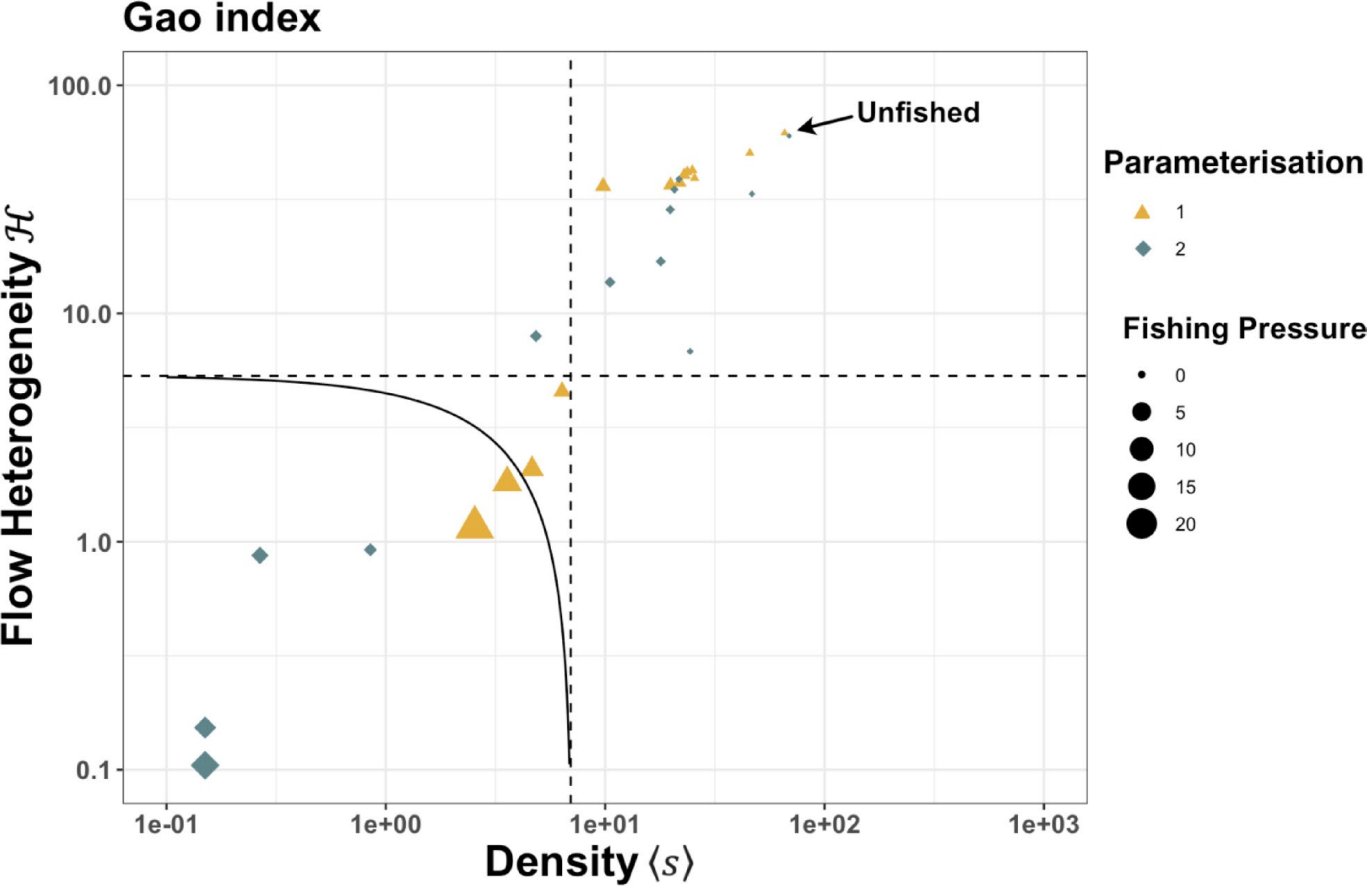
Gao’s resilience plot for two parameterisations for an EwE for southeast Australia.

The system is rated as resilient while fishing pressure remains below 1.5×F_MMSY_ (Fig. 3, Table S7), although it moves closer to the region of partial resilience. For both parameterisations of the model, once pressure exceeds 1.5xF_MMSY_ the value for system density is more likely to be below the threshold for a partially resilient state (Fig. 3). As fishing pressure increases past 2–5 × F_MMSY_ the system moves into the collapse zone. Both model configurations follow this general path of system evolution, but the drop in resilience, and the transition to being on the boundary or inside the collapse zone, happens at lower levels of fishing pressure and more suddenly for parameterisation 2 than for parameterisation 1.

#### ETI

**Fig. 4:**
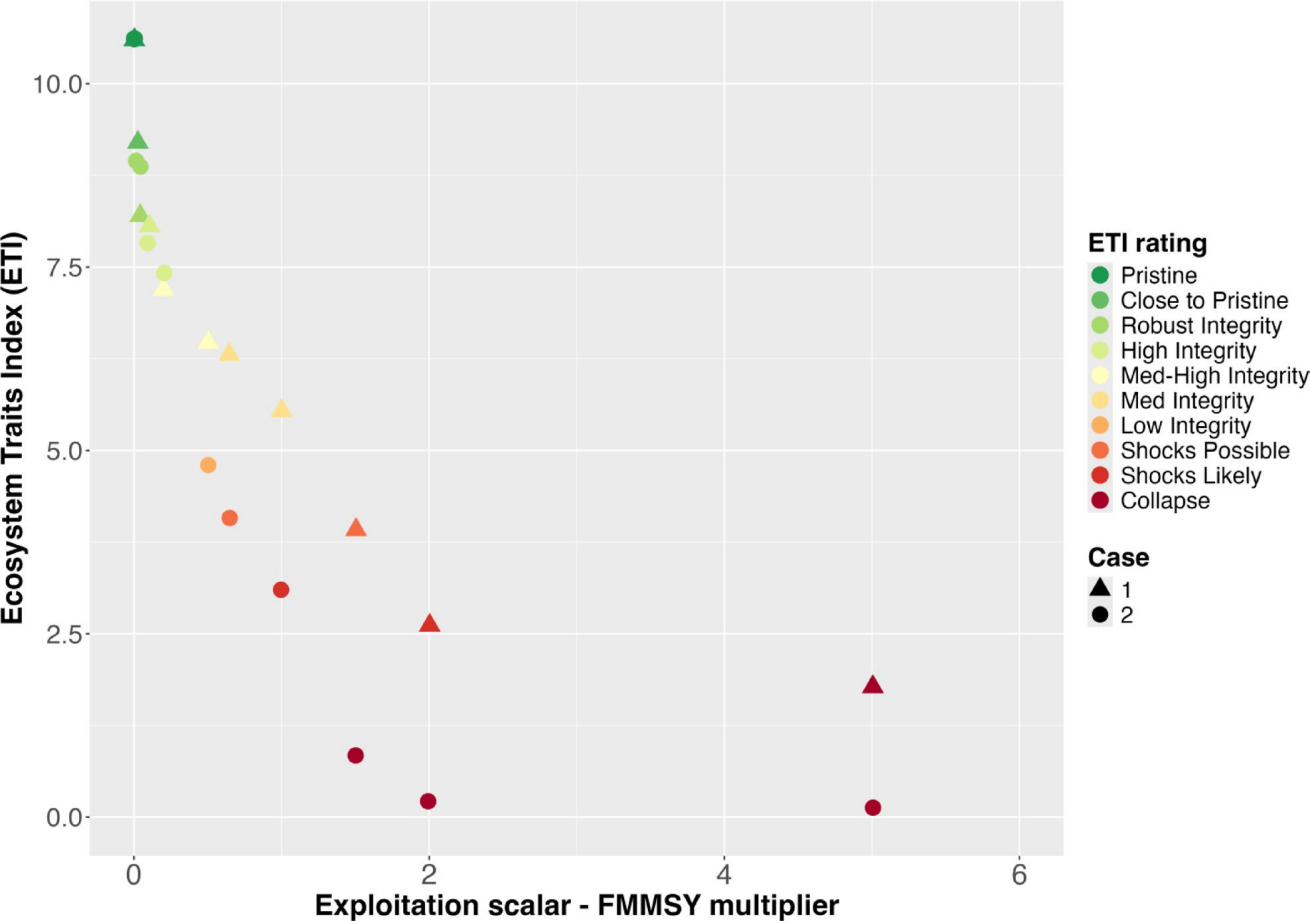
ETI for two depletion cases for an EwE for southeast Australia (x axis truncated at 6 for ease of plot interpretation; 5xF_MMSY_ and higher all have ETI values in the collapsed state).

The ETI calculated from the outputs of simulations using parameterisation 1 of the southeast Australian EwE model decreases rapidly as the fishing pressure reaches and exceeds F_MMSY_. However, parameterisation 2 reaches degraded ETI values (i.e. “shocks possible”) faster than under parameterisation 1 (Fig. 4, Table S8). This shows how quickly fishing can modify a system’s state. The decrease in ETI is quite rapid in both cases, but case 1 retains medium or higher integrity than case 2 until fishing pressure exceeds F_MMSY_, while in case 2 the ETI drops to low integrity when fishing pressure is half of F_MMSY_.

This test shows that the suggested approach, the suite of indicators and their combination within the ETI, is sensitive and responsive to the marine ecosystem state and to how the ecosystem is modelled.

### Test 2: Historical time series of ecosystem state

#### Hub species

The Hub species for each case study (Table S9) highlight the importance of omnivorous predators. Squid, sharks and predatory fish (such as arrowtooth flounder for the East Bering Sea, or Hake in the Chilean model) are identified as hub species across the various modelled systems. The results also highlight the importance of species that provide supporting structure to the overall web such as widely used habitat or broadly consumed prey species, and of different kinds of zooplankton (including Euphausiids and large copepods) as hub species.

#### Green band

In the Chilean or Indian systems very few of the groups are in the Green Band under historical fishing (Table S10), with most groups being above the Green Band indicating a high level of distortive pressure. Although, in each of these systems some species, even amongst the most vulnerable, are not under excessive pressure from fisheries because they do not often directly interact with the fisheries. In the eastern Bering Sea and southeast Australia, the majority of species are within or below the Green Band, indicating low distortive pressure. Nevertheless, there are species above the Green Band in both systems – especially the more vulnerable species (e.g. species with incidental fishery interactions in the eastern Bering Sea) or main target and/or byproduct species.

#### Gao’s resilience

All four systems were in the upper left quadrant of the plot (Figure S1) and are partially resilient. Chile and Kerala show the clearest change through time, decreasing slowly toward the non-resilient threshold. The points for the eastern Bering Sea are tightly clustered in Figure S1, but if the axes are closely examined (Figure S2), the points effectively oscillate along an arc of $\mathcal{H}$ and < s > values. The points for the southeast Australian system are a little more dispersed (Figure S2), they show a small increase in $\mathcal{H}$ initially but a steadily downward trending trajectory after 1988 and have much lower $\mathcal{H}$ scores than for any of the other systems. Compared to other systems, the southeast Australian system has a lower basal productivity and energy flow is concentrated through a smaller number of system pathways (Figure S1).

#### ETI

The ETI for the Australian system showed the greatest variation through time, dropping from high integrity in the 1980s to periods of medium and low integrity in the years leading up to 2020 (Figure S3, Table S11). Kerala’s ETI also varied through time, from medium to low integrity prior to 2001 before suddenly dropping to “shocks possible” (Figure S3, Table S11). The sudden drop in 2001 in Kerala comes shortly after an increase in effort amongst motorised vessels that gave a substantial increase in catch of all fished groups, including many of the main target species (e.g. Indian oil sardine), many pelagic species from anchovies to tuna, various cephalopods and crustacea, and finfish and sharks from deeper waters that previously were not intensively fished. This also marks the start of the period where sudden, but typically short-lived, “outbreaks” of individual species (e.g. triggerfish) occurred.

In contrast, the eastern Bering Sea almost always had medium–high Integrity, with a very slight downward trend from high integrity ceasing in 1987 (Figure S3, Table S11). The Chilean result was not as positive, starting with “shocks possible” initially and dropping to “shocks likely” post 2013 (Figure S3, Table S11).

This test of performance across different ecosystem types and histories of fishery change reinforces that ETI is sensitive and responsive to marine ecosystem state at a level that could be informative for ecosystem management. Some indicators used in the ETI, such as Gao’s resilience, do not show a lot of variation in some systems, but this does not undermine the utility of the overall approach. Comparing results across ecosystems shows that ETI has can detect poorer performing ecosystem and fisheries management.

### Test 3: Future projections

#### Hub species

For each case study region, the hub species for the future projections considered did not differ to those for the historical system state (Table S9), though in southeast Australia mesopelagics moved from marginal to core hub species.

#### Green band

In every region tested, the climate change and marine heatwave scenarios led to productivity shifts for many species, resulting in a 5–350% increase in the number of species above the Green Band (Table S12). The effects on the eastern Bering Sea system are more widespread across the ecosystem than for any other system, with every category having more species above the Green Band – 1.1–1.5 times more for byproduct and target species and a twofold or more increase for other categories. In southeast Australia the target and byproduct species were most effected, particularly where marine heatwaves occurred along with climate change. In Chile and India it was primarily target and hub species that moved above the Green Band, though small numbers of species in other classes also showed this movement.

Vulnerable species were negatively affected by marine heatwaves, and these species often had low catches combined with very low productivity. Consequently, any species already above the Green Band moved even further away from it, while species initially in the Green Band either moved toward its upper bound or moved completely through the Green Band.

Reducing fishing pressure has the opposite effect to the climate change scenarios, with all systems having more species within or below the Green Band when fishing is reduced. This was the case for target and byproduct species in all systems, hub species also benefited in Kerala and north central Chile.

#### Gao’s resilience

For the scenarios examined all systems remained partially resilient throughout the projection period and no system breached the Gao resilience frontier (Figures S2–S7), though the southeast Australian system approached the collapse frontier under the climate change with heatwaves (Figure S7). Where there were observable shifts the resilience values trended toward the collapse frontier as climate warmed, particularly with marine heatwaves, while reducing fishing pressure moved points away from the frontier. Kerala (Figure S5) and southeast Australia (Figure S7) showed the strongest responses, in both cases under the scenario of combined climate change and heatwaves. In contrast, the variation in the values for the eastern Bering Sea was so small as to be effectively unnoticeable (Figure S2).

#### ETI

In all the scenarios (Figure S8, Table S13) the ETI declined through time under climate change, with ETI decreasing still lower with marine heatwaves. Some recovery is possible between heatwaves, but often not to the same level as the matching year in the climate projection with no heatwave. Conversely, the ETI rose slowly and steadily through time when fishing was reduced, and as the system rebuilt.

These tests indicate that the proposed approach responds to increases and decreases in pressure on a marine ecosystem. However, it could not discern fishing effects from climate effects if those drivers both modified ecosystems in the same way, such as be causing similar change in system structure or productivity.

## Discussion

This work develops and applies network-based indicators to several ecosystems and tests whether they could provide useful insights for fisheries and marine ecosystem management. The results of the tests show that the indices respond under combined stressors, including fishing and climate, and how rapidly degradation and recovery in ecosystems can be seen. The proposed ecosystem metric (ETI) reflects change in a timely way, and ecosystem structure responds when fishing pressure is reduced or applied in-line with structural considerations. The results highlight the importance of omnivorous predators and habitat forming species to the topological structure of marine food webs. Some ecosystems are already under distortive pressure from fishing, and climate change and marine heat waves will exacerbate the situation.

Taking a systems approach to fisheries usually relies on performance indicators linked solely to stock status and biomass trends^60–62^. There are exceptions that attempt to more directly consider aspects of structure and function^63^, so an expanded approach focusing on biodiversity and resilience is emerging^64^. Where ecosystem structure and function have been considered it has usually been through habitat dependency (e.g. where kelp or corals are important^13,14^) or through consideration of immediate predators or prey^65,66^. However, network theory provides a more holistic and quantifiable insight into overall system function^26,67,68^. The use of network indices to track the state and integrity of many kinds of systems is increasing the accessibility of these indices for decision makers and for communication^68–70^. This wider application of network theory and indicators has inspired work in fisheries^67,71,72^. Network theory is one of few approaches that can provide generalisable methods and indicators despite the system specificity of responses to perturbation in marine ecosystems.

Previous work on indicators for ecosystem approaches to fisheries and conservation identified many desirable indicator characteristics^73^, but chief among them were specificity, sensitivity and responsiveness. However, ecosystem responses can be strongly nonlinear, not distinguish between stressors, and be context specific. This complicates the search for generally useful indicators^8,16,50,74–78^. The tests here have attempted to validate the sensitivity, specificity and responsiveness of the ETI and its component indicators.

This suite of indicators appears sensitive to ecosystem changes, whether from fishing pressure (Figs. 3 and 4) or climate drivers (supplementary materials), responding rapidly when conditions vary. Importantly, they are responsive to variations typical of real systems and can be calculated and reported with the same rapidity as any other fisheries or conservation reporting mechanisms. Consequently, they are likely to be sensitive enough to be useful for providing contextual guidance to decision-makers or (potentially) being built into fishery harvest control rules. The indicators considered here do not give large or exaggerated change as the ecosystem changes, which is another positive attribute in decision making. The very small change in Gao’s resilience in the eastern Bering Sea is reflective of the small change the model is predicting in that system, in contrast to the much larger changes both modelled and observed seen in Kerala or southeast Australia.

The indicators do not show substantially different responses to the climate change and fisheries stressors, so the ecosystem responses are also similar across these two kinds of stressor. This result provides more evidence that finding indicators that can separately recognise and address all the stressors on an ecosystem may be unachievable. The analyses of historical time series and simulation testing conducted here both suggest that in most cases indicators alone do not allow attribution of specific changes to specific drivers. Although a suitably designed experiment may be able to distinguish attribution by stressor. If stressors do act together, then decision makers may need to respond to the compound outcome in a “no regrets” manner irrespective of the cause.

While the indicators are responsive to ecosystem change the way in which they are calculated means they are not lagging nor leading indicators. While they reflect change, they cannot anticipate it. Advances in network analysis during the last decade^24,25^, and extensive simulation testing, have made it possible to define the indicators that can compare the current state to states where desirable or undesirable change may occur (e.g. when system structure has been reduced so that it can no longer support past ecological function and further species losses or a break down in biogeochemical cycling becomes likely). These are not as certain as leading indicators, but they can provide decision makers with likely outcomes if an indicator is consistently changing in an undesirable direction (e.g. if the species are moving further above the Green Band, closer to the Gao resilience frontier or to lower values of the ETI). Previous work has shown that this kind of directional inference is typically the best that can be expected for ecosystems and that it is informative for decision making^60^. However, while the qualitative ETI bands used to colour the ETI plots can be useful in deciding whether a change in the ETI is substantial or not, or what level of management intervention is required, it is best to follow the ETI value over time to inform decision making.

The tests of the indicators presented here, and the broader set available in Fulton et al.^79^, show that different ecosystems can be in very different states depending on the historical combination of fishing pressure, environmental change and the inherent robustness of the ecosystem structure. Of the systems analysed, Kerala, north central Chile and southeast Australia appear to have undergone moderate or stronger system distortion due to exploitation and other anthropogenic stressors. Further change is projected under climate change, potentially to quite undesirable states. The fisheries scenarios indicate, however, that ecosystem structure could be rebuilt relatively quickly (i.e. within years to decades). The reality may be that climate change may prevent fully recovering ecosystems unless climate change itself halts or reverses.

Nonetheless, the sensitivity of the indicators means that management responses can be made before very large-scale change makes recovery more difficult. For example, managing highly biodiverse fisheries, such as those in Kerala can be extremely difficult, but by pairing a trigger- or indicator-based management approach (such as that used in Western Australia^80^) with the indices discussed here timely management interventions could be made. The test showed that several species in the Kerala modelled system moved from below the Green Band to within the Green Band for marine heatwave projections (Table S13), and this change could have been sufficient to trigger a management response if following a trigger- or indicator-based management approach, thereby forestalling those species from progressing to a position above the Green Band.

It is also important to note that management agencies can apply the commonly applied single species management and still not meet the requirements for ecosystem structure and function. A good example is the eastern Bering Sea, which receives significant scientific monitoring and investment in fisheries and conservation management. This investment is likely why so many species groups in that system are rated as “light” or “acceptable” in the Green Band analyses (Table S9). The proportion of groups below the Green Band in the eastern Bering Sea is higher (at 50%) than for any of the other three systems (where it is 26–40%). Nevertheless, despite compliance with single species management requirements in the eastern Bering Sea there are non-trivial numbers of species groups that are above the Green Band, with over half of these being target species (including Atka Mackerel which is also a Hub species). This is because the reference points of single species management are not aligned with ecosystem Green Band considerations, with the degree of divergence increasing for the most vulnerable species.

An early form of the ETI was used as an ecosystem indicator in the Gulf of Thailand^45^. Principal component and heatmap analyses of that region showed clear evidence of ‘regimes’ in the catch, associated with vastly different levels of potential fishery production that was estimated from an aggregate production model and EwE model^45^, economic returns and ETI scores as it was calculated then (Table S14). The current ETI the Gulf of Thailand shows that the ETI initially decreased over time, from “Close to Pristine” to “Shocks likely” and then to “Collapse likely” for a short period before a reduction in fishing pressure in 2015 eased pressure on the system (Figure S9). This shows how the ETI, along with typically reported socioeconomic metrics (catch, jobs, value per unit effort etc.) can inform decision makers on trade-offs in the system and the implications of different levels of system-wide exploitation and structural distortion.

We have developed and applied the ETI using model derived data. However, it could in principle be calculated based on direct observational data – survey biomasses, catches, diet data and habitat use rates. The Hub species have already been calculated for observational time series as part of testing its efficacy in a management context.^31^ Nevertheless, the scale of ecosystems and the difficulty of collecting data makes calculating ecosystem-relevant information more challenging than following single species. The indices considered here are no exception. There is always concern when working with networks that the extent to which the network can be resolved will ultimately dictate the results. However previous work has shown that the sub-components used to calculate the ETI are reasonably robust to the taxonomic resolution of the model used^31,41^. It is important to have quantitative measures for diets (and habitat dependency if included), because while binary (presence, absence) diet data is sufficient for identifying hub species more complete quantitative data is needed to calculate Gao’s resilience. Additionally, at present calculating the ETI typically relies on ecosystem model output to generate the unfished profile for the Green Band step, because for most ecosystems there is no data on the unfished ecosystem. However, in theory, the unfished profile could be calculated solely from observational data if suitably long time series were available. A useful approach may be that of “space for time” substitution, as recently applied to generate multispecies reference points for coral reefs^81^.

While we have focused on marine applications, the concepts could also be applied terrestrially, for example in managing forests or grasslands^82^, though their actual utility would need to be examined for their different ecosystem structure and function. This study demonstrates that network indices can provide a practical means of extending tactical fisheries management beyond consideration of individual stock status, and we have shown that through applications in several very different ecosystems and fishery management systems.

## Conclusions

This paper describes the performance of a set of potential indicators that directly account for the integrity of ecosystem structure and function, which could be used in ecosystem-based fisheries management. Our results suggest that these structural indicators are a promising way of tracking ecosystem state for management decision making, capturing fundamental properties of system structure and how it is influenced by system perturbations. The indicators are not specific to fisheries because they respond to any drivers that are impacting the ecosystem. Consequently, they should be used in combination with other sources of information, such as multivariate analyses of catch composition, to allow for maximal attribution of the causes of any changes seen in the indices.

We have focused on structural components of the ecosystem because this was an apparent gap in existing ecosystem indices and because it appeared the most tractable with available data and modelling tools. Moreover, these structural ecosystem traits can complement the size-based approaches and models for tracking marine ecosystem processes^20,50^. The expanding diversity of observational platforms, analysis and synthesis power in new computational methods may open the door to more direct measures of ecosystem-scale functional traits in future. For now, however, structurally focused indices, such as the ETI, appear to be a good, available and pragmatic option.

While identification of hub species, a species location with respect to the Green Band, and how an ecosystem is tracking compared to the Gao resilience frontier, could individually inform decision making, they can also be combined into the composite ETI. Some jurisdictions may prefer to consider the detail of the individual indicators, while others may want to use the composite ETI for more general and indicative information.

It is likely that the ETI will evolve, be refined and perhaps simplified. Initial exploration suggests that 10 categories for the ETI is a good balance of communication clarity, explanatory capacity and responsiveness so action can be taken. However, future iterations may be simpler. Nevertheless, incorporation of the ETI, or a similar index, into annual fisheries and conservation reporting could see ecosystem structure and “integrity” become a mainstream component of decision making.

## Supplementary Information

Below is the link to the electronic supplementary material.

Supplementary Material 1

## Data Availability

The scripts used to generate the indicators and the figures in this paper is available from https://github.com/eafulton/ETI-Gao.git. The individual models (and scenarios) have been documented in the supplementary materials. Further details of the models have previously been published and can be accessed via the relevant publications (as noted in the paper) or by directly contacting the corresponding author.

## References

[1] FAO. Fisheries Management. 2. The ecosystem approach to fisheries. FAO Technical Guidelines for Responsible Fisheries 4 (suppl. 2). (FAO, 2003). http://www.fao.org/3/y4470e/y4470e.pdf

[2] García, S. M., Zerbi, A., Aliaume, C., et al. The ecosystem approach to fisheries: issues, terminology, principles, institutional foundations, implementation and outlook. (FAO, Ed.). (Food and Agriculture Organization of the United Nations: Rome, 2003)

[3] Pikitch, E. K. et al. ECOLOGY: Ecosystem-based fishery management. *Science* **305**, 346–347. 10.1126/science.1098222 (2004). 15256658 10.1126/science.1098222

[4] Patrick, W. S. & Link, J. S. Myths that continue to impede progress in ecosystem-based fisheries management. *Fisheries* **40**, 155–160. 10.1080/03632415.2015.1024308 (2015).

[5] Ramírez-Monsalve, P. et al. Ecosystem approach to fisheries management (EAFM) in the EU – current science–policy–society interfaces and emerging requirements. *Mar. Policy* **66**, 83–92. 10.1016/j.marpol.2015.12.030 (2016).

[6] Islam, M. M. et al. Status and potential of ecosystem approach to fisheries management (EAFM) in Bangladesh. *Ocean Coast. Manag.* **219**, 106068. 10.1016/j.ocecoaman.2022.106068 (2022).

[7] Convention on Biological diversity (CBD). The Ecosystem Approach, (CBD Guidelines) Montreal: Secretariat of the Convention on Biological Diversity 50 (2004).

[8] Fulton, E. A., Smith, A. D. M. & Punt, A. E. Which ecological indicators can robustly detect effects of fishing?. *ICES J. Mar. Sci.* **62**, 540–551. 10.1016/j.icesjms.2004.12.012 (2005).

[9] Shin, Y.-J. & Shannon, L. J. Using indicators for evaluating, comparing, and communicating the ecological status of exploited marine ecosystems. 1. The IndiSeas project. *ICES J. Mar. Sci.* **67**, 686–691. 10.1093/icesjms/fsp273 (2010).

[10] Longo, C. et al. Role of trophic models and indicators in current marine fisheries management. *Mar. Ecol. Prog. Ser.* **538**, 257–272 (2015).

[11] Pratchett, M. S. et al. Changes in biodiversity and functioning of reef fish assemblages following coral bleaching and coral loss. *Diversity* **3**, 424–452. 10.3390/d3030424 (2011).

[12] Graham, N. A. J. & Nash, K. L. The importance of structural complexity in coral reef ecosystems. *Coral Reefs* **32**, 315–326. 10.1007/s00338-012-0984-y (2013).

[13] Gaichas, S. K. et al. A framework for incorporating species, fleet, habitat and climate interactions into fishery management. *Front. Mar. Sci.* **3**, 105. 10.3389/fmars.2016.00105 (2016).

[14] Rogers, A., Blanchard, J. L. & Mumby, P. J. Fisheries productivity under progressive coral reef degradation. *J. Appl. Ecol.* **55**, 1041–1049. 10.1111/1365-2664.13051 (2018).

[15] Jouffray, J.-B. et al. Parsing human and biophysical drivers of coral reef regimes. *Proc. R. Soc. B* **286**, 20182544. 10.1098/rspb.2018.2544 (2019). 30963937 10.1098/rspb.2018.2544 PMC6408596

[16] Link, J. S. Translating ecosystem indicators into decision criteria. *ICES J. Mar. Sci.* **62**, 569–576. 10.1016/j.icesjms.2004.12.015 (2005).

[17] Winfree, R. W. et al. Abundance of common species, not species richness, drives delivery of a real-world ecosystem service. *Ecol. Lett.* **18**, 626–635. 10.1111/ele.12424 (2015). 25959973 10.1111/ele.12424

[18] Brose, U. & Hillebrand, H. Biodiversity and ecosystem functioning in dynamic landscapes. *Philos. Trans. R. Soc. B* **371**, 20150267. 10.1098/rstb.2015.0267 (2016). 10.1098/rstb.2015.0267 PMC4843689 27114570

[19] Maureaud, A., Andersen, K. H., Zhang, L. & Lindegren, M. Trait-based food web model reveals the underlying mechanisms of biodiversity–ecosystem functioning relationships. *J. Anim. Ecol.* **89**, 1497–1510 (2019). 10.1111/1365-2656.13207 32162299

[20] Blanchard, J. L. et al. Do climate and fishing influence size-based indicators of Celtic Sea fish community structure?. *ICES J. Mar. Sci.* **62**, 405–411. 10.1016/j.icesjms.2005.01.006 (2005).

[21] Andersen, K. H., Berge, T. & Gonçalves, R. J. Characteristic sizes of life in the oceans, from bacteria to whales. *Annu. Rev. Mar. Sci.* **8**, 3.1-3.25. 10.1146/annurev-marine-122414-034144 (2016). 10.1146/annurev-marine-122414-034144 26163011

[22] Fath, B. D., Scharler, U. M., Ulanowicz, R. E. & Hannon, B. Ecological network analysis: Network construction. *Ecol. Model.* **208**, 49–55. 10.1016/j.ecolmodel.2007.04.029 (2007).

[23] Lau, M. K. et al. Ecological network metrics: Opportunities for synthesis. *Ecosphere*10.1002/ecs2.1900 (2017).

[24] Newman, M. E. J. et al. (eds) *The Structure and Dynamics of Networks* (Princeton, 2006).

[25] Gao, J., Barzel, B. & Barabási, A.-L. Universal resilience patterns in complex networks. *Nature* **530**, 307–312. 10.1038/nature16948 (2016). 26887493 10.1038/nature16948

[26] Yletyinen, J. et al. Regime shifts in marine communities: A complex systems perspective on food web dynamics. *Proc. R. Soc. B Biol. Sci.* **283**, 20152569. 10.1098/rspb.2015.2569 (2016). 10.1098/rspb.2015.2569 PMC4810827 26888032

[27] Ulanowicz, R. E. *Growth and Development: Ecosystems Phenomenology* (New York, 1986).

[28] Rooney, N. & McCann, K. S. Integrating food web diversity, structure and stability. *Trends Ecol. Evol.* **27**, 40–46. 10.1016/j.tree.2011.09.001 (2011). 21944861 10.1016/j.tree.2011.09.001

[29] Condie, S. A., Johnson, P., Fulton, E. A. & Bulman, C. M. Relating food web structure to resilience, keystone status and uncertainty in ecological responses. *Ecosphere* **5**, 81. 10.1890/ES14-00068.1 (2014).

[30] Dowdy, A. J., Mills, G. A., Finkele, K. & de Groote, W. *Australian Fire Weather as Represented by the McArthur Forest Fire Danger Index and the Canadian Forest Fire Weather Index* (Melbourne, 2009).

[31] Fulton, E. A. & Sainsbury, K. Foodweb structure, the Hub Index and identifying species of ecological significance. *Ecol. Ind.* **166**, 112378. 10.1016/j.ecolind.2024.112378 (2024).

[32] Heath, M., Law, R. & Searle, K. Scoping the background information for an ecosystem approach to fisheries in Scottish waters: Review of predator-prey interactions with fisheries, and balanced harvesting. A study commissioned by Fisheries Innovation Scotland (FIS) (2017). http://www.fiscot.org/

[33] He, N. P. et al. Ecosystem traits linking functional traits to macroecology. *Trends Ecol. Evol.* **34**, 200–210. 10.1016/j.tree.2018.11.004 (2019). 30527959 10.1016/j.tree.2018.11.004

[34] Yan, G. et al. Criticality analysis of Internet infrastructure. *Comput. Netw.* **54**, 1169–1182. 10.1016/j.comnet.2009.11.002 (2010).

[35] Dhuli, S. & Singh Y. N. Papers with Code - Network Criticality Analysis for Finite Sized Wireless Sensor Networks. (2014). Available at https://cs.paperswithcode.com/paper/network-criticality-analysis-for-finite-sized [Verified October 2023]

[36] Katina, P. F., Keating, C. B., Zio, E. & Gheorghe, A. V. A criticality-based approach for the analysis of smart grids. *Technol. Econ. Smart Grids Sustain. Energy* **1**, 14. 10.1007/s40866-016-0013-2 (2016).

[37] Jafino, B. Measuring Freight Transport Network Criticality A Case Study in Bangladesh. Master of Science - MSc (Delft University of Technology, 2017).

[38] Kim, S. & Yeo, H. Evaluating link criticality of road network based on the concept of macroscopic fundamental diagram. *Transp. A: Transp. Sci.* **13**, 162–193. 10.1080/23249935.2016.1231231 (2017).

[39] Wilson, K. G. The renormalization group: Critical phenomena and the Kondo problem. *Rev. Mod. Phys.* **47**, 773–840. 10.1103/RevModPhys.47.773 (1975).

[40] Dunne, J. A., Williams, R. J. & Martinez, N. D. Network structure and robustness of marine food webs. *Mar. Ecol. Prog. Ser.* **273**, 291–302 (2004).

[41] Fulton, E. A., Sainsbury, K., Bulman, C. M. et al. (in review). “The green band”: Using production and catch to judge distortive pressure on an ecosystem. bioRxiv 2025.06.09.658403; 10.1101/2025.06.09.658403

[42] Fulton, E. A., Smith, A. D. M. & Johnson, C. R. Effect of complexity on marine ecosystem models. *Mar. Ecol. Prog. Ser.* **253**, 1–16 (2003).

[43] Link, J. S. Adding rigor to ecological network models by evaluating a set of pre-balance diagnostics: A plea for PREBAL. *Ecol. Model.* **221**, 1582–1593 (2010).

[44] Heymans, J. J. et al. *Ecol. Model.* **331**, 173–184 (2016).

[45] Fulton, E. A. et al. Shifting baselines and deciding on the desirable form of multispecies maximum sustainable yield. *ICES J. Mar. Sci.* **79**, 2138–2154. 10.1093/icesjms/fsac150 (2022).

[46] Pauly, D., Christensen, V. & Walters, C. J. Ecopath, Ecosim and Ecospace as tools for evaluating ecosystem impact of fisheries. *ICES J. Mar. Sci.* **57**, 697–706 (2000).

[47] Kuriakose, S., Bulman, C., Fulton, E. A. et al. Ecosystem modelling using Ecopath and Ecosim (EwE) and simulation of the Kerala marine fishery ecosystem. CMFRI Report. (2021). Accessed April 2025: https://fisheryprogress.org/sites/default/files/documents_actions/Ecopath%20model%20Kerala%20Lenfest%20Report%20Aug2021_2.pdf

[48] Aydin, K., Gaichas, S., Ortiz, I. et al. Comparison of the Bering Sea, Gulf of Alaska, and Aleutian Islands Large Marine Ecosystems Through Food Web Modeling. (US Department of Commerce, 2007).

[49] Bulman, C., Condie, S., Furlani, D., He, X., Rathbone, C., Knuckey, I. & Goldsworthy, S. *Trophodynamic modelling of the Eastern Bass Strait Shelf*. (Final Report for the National Oceans Office. CSIRO, NOO, FRDC, Hobart, 2002).

[50] Shin, Y.-J. et al. The specificity of marine ecological indicators to fishing in the face of environmental change: A multi-model evaluation. *Ecol. Ind.* **89**, 317–326. 10.1016/j.ecolind.2018.01.010 (2018).

[51] Walters, C. J., Christensen, V., Martell, S. J. & Kitchell, J. F. Possible ecosystem impacts of applying MSY policies from single-species assessment. *ICES J. Mar. Sci.* **62**, 558–568 (2005).

[52] Hobday, A. J. & Pecl, G. T. Identification of global marine hotspots: Sentinels for change and vanguards for adaptation action. *Rev. Fish Biol. Fisheries* **24**, 415–425. 10.1007/s11160-013-9326-6 (2014).

[53] Boucher, O., Denvil, S., Levavasseur, G., et al. IPSL IPSL-CM6A-LR model output prepared for CMIP6 CMIP. Version 2022–06–03. Earth System Grid Federation. (2018). 10.22033/ESGF/CMIP6.1534

[54] John, J. G., Blanton, C., McHugh, C., et al. NOAA-GFDL GFDL-ESM4 model output prepared for CMIP6 ScenarioMIP. Version YYYYMMDD[1]. Earth System Grid Federation. (2018). 10.22033/ESGF/CMIP6.1414

[55] Pielke, R. Jr., Burgess, M. G. & Ritchie, J. Plausible 2005–2050 emissions scenarios project between 2 °C and 3 °C of warming by 2100. *Environ. Res. Lett.* **17**, 024027. 10.1088/1748-9326/ac4ebf (2022).

[56] Hobday, A. J. et al. Categorizing and naming marine heatwaves. *Oceanography* **31**, 162–173. 10.5670/oceanog.2018.205 (2018).

[57] Holbrook, N. J. et al. A global assessment of marine heatwaves and their drivers. *Nat. Commun.* **10**, 2624. 10.1038/s41467-019-10206-z (2019). 31201309 10.1038/s41467-019-10206-z PMC6570771

[58] Oliver, E. C. J. et al. Projected marine heatwaves in the 21st Century and the potential for ecological impact. *Front. Mar. Sci.* **6**, 734. 10.3389/fmars.2019.00734 (2019).

[59] Oliver, E. C. J. et al. Marine heatwaves. *Ann. Rev. Mar. Sci.* **13**, 313–342. 10.1146/annurev-marine-032720-095144 (2021). 32976730 10.1146/annurev-marine-032720-095144

[60] Jennings, S., Kaiser, M. J. & Reynolds, J. D. *Marine Fisheries Ecology* (Oxford; Malden, MA, USA, 2001).

[61] Bundy, A. et al. The good(ish), the bad, and the ugly: A tripartite classification of ecosystem trends. *ICES J. Mar. Sci.* **67**, 745–768. 10.1093/icesjms/fsp283 (2010).

[62] Smit, K. P., Bernard, A. T. F., Lombard, A. T. & Sink, K. J. Assessing marine ecosystem condition: A review to support indicator choice and framework development. *Ecol. Ind.* **121**, 107148. 10.1016/j.ecolind.2020.107148 (2021).

[63] Large, S. I., Fay, G., Friedland, K. D. & Link, J. S. Quantifying patterns of change in marine ecosystem response to multiple pressures. *PLoS ONE* **10**, e0119922. 10.1371/journal.pone.0119922 (2015). 25781166 10.1371/journal.pone.0119922 PMC4362946

[64] Flensborg, L. C., Maureaud, A. A., Bravo, D. N. & Lindegren, M. An indicator-based approach for assessing marine ecosystem resilience. *ICES J. Mar. Sci.* **80**, 1487–1499. 10.1093/icesjms/fsad07 (2023).

[65] Blamey, L. K., Plagányi, E. E. & Branch, G. M. Was overfishing of predatory fish responsible for a lobster-induced regime shift in the Benguela?. *Ecol. Model.* **273**, 140–150. 10.1016/j.ecolmodel.2013.11.004 (2014).

[66] Punt, A. E. et al. Exploring the implications of the harvest control rule for Pacific sardine, accounting for predator dynamics: A MICE model. *Ecol. Model.* **337**, 79–95. 10.1016/j.ecolmodel.2016.06.004 (2016).

[67] Rocchi, M., Scotti, M., Micheli, F. & Bodini, A. Key species and impact of fishery through food web analysis: A case study from Baja California Sur, Mexico. *J. Mar. Syst.* **165**, 92–102. 10.1016/j.jmarsys.2016.10.003 (2017).

[68] Fath, B. D. et al. Ecological network analysis metrics: The need for an entire ecosystem approach in management and policy. *Ocean Coast. Manag.* **174**, 1–14. 10.1016/j.ocecoaman.2019.03.007 (2019).

[69] Tam, J. C. et al. Towards ecosystem-based management: identifying operational food-web indicators for marine ecosystems. *ICES J. Mar. Sci.* **74**, 2040–2052. 10.1093/icesjms/fsw230 (2017).

[70] de Jonge, V. N. & Schückel, U. A comprehensible short list of ecological network analysis indices to boost real ecosystem-based management and policy making. *Ocean Coast. Manag.* **208**, 105582 (2021).

[71] Bourdaud, P., Gascuel, D., Bentorcha, A. & Brind’AmourdaI, A. New trophic indicators and target values for an ecosystem-based management of fisheries. *Ecol. Ind.* **61**, 588–601. 10.1016/j.ecolind.2015.10.010 (2016).

[72] Ito, M., Halouani, G., Cresson, P., Giraldo, C. & Girardin, R. Detection of fishing pressure using ecological network indicators derived from ecosystem models. *Ecol. Ind.* **147**, 110011. 10.1016/j.ecolind.2023.110011 (2023).

[73] Rice, J. C. & Rochet, M. J. A framework for selecting a suite of indicators for fisheries management. *ICES J. Mar. Sci.* **62**, 516–527. 10.1016/j.icesjms.2005.01.003 (2005).

[74] Link, J. S. et al. Relating marine ecosystem indicators to fishing and environmental drivers: an elucidation of contrasting responses. *ICES J. Mar. Sci.* **67**, 787–795 (2010).

[75] Shannon, L. J. et al. Comparing data-based indicators across upwelling and comparable systems for communicating ecosystem states and trends. *ICES J. Mar. Sci.* **67**, 807–832. 10.1093/icesjms/fsp270 (2010).

[76] Shin, Y.-J. et al. Can simple be useful and reliable? Using ecological indicators to represent and compare the states of marine ecosystems. *ICES J. Mar. Sci.* **67**, 717–731 (2010).

[77] Link, J. S. et al. Emergent properties delineate marine ecosystem perturbation and recovery. *Trends Ecol. Evol.* **30**, 649–661. 10.1016/j.tree.2015.08.011 (2015). 26456382 10.1016/j.tree.2015.08.011

[78] Fu, C. et al. Responses of ecological indicators to fishing pressure under environmental change: Exploring non-linearity and thresholds. *ICES J. Mar. Sci.* **77**, 1516–1531. 10.1093/icesjms/fsz182 (2019).

[79] Fulton, E. A. et al. *Benchmarks for Ecosystem Assessment: Indicators and Guidelines for Practical Ecosystem Based Fishery Management (EBFM)* (CSIRO, 2021).

[80] Newman, S. J. et al. A risk assessment and prioritisation approach to the selection of indicator species for the assessment of multi-species, multi-gear, multi-sector fishery resources. *Mar. Policy* **88**, 11–22. 10.1016/j.marpol.2017.10.028 (2018).

[81] Zamborain-Mason, J. et al. Sustainable reference points for multispecies coral reef fisheries. *Nat. Commun.* **14**, 5368. 10.1038/s41467-023-41040-z (2023). 37666831 10.1038/s41467-023-41040-z PMC10477311

[82] Zhang, M. et al. State-of-the-art and challenges in global grassland degradation studies. *Geogr. Sustain.* **6**, 100229. 10.1016/j.geosus.2024.08.00 (2025).

