# Supplementary material for: The ecosystem traits index is proposed as a composite index of ecosystem robustness for use in marine resource management
Source: Sci Rep. 2025 Sep 2;15:32281. doi: 10.1038/s41598-025-15322-z (PMC12402227; doi:10.1038/s41598-025-15322-z)
Supplement: Supplementary file 1 — Supplementary Material 1 [file 41598_2025_15322_MOESM1_ESM.pdf]

Supplementary Materials

The Ecosystem Traits Index is proposed as a composite index of ecosystem robustness for use in marine resource management

E.A. Fulton, K. Sainsbury

Glossary of terms

Criticality analysis – analysis of network structure to identify nodes or segments (nodes and links) that are critical to performance of the network. The flow through the network would severely degrade If these critical components are lost, leading to the failure or fragmentation of the network.

Degree – The number of connections (in or out) of a node in a network.

Degree in – The number of connections into a node in a network.

Degree out – The number of connections out of a node in a network.

Ecological (functional) traits – characteristics of species (morphological, physiological, or phenological) that influences the ecological processes of a species, how it responds to environmental pressures and its role in the structure and function of an ecosystem.

Ecological integrity – the capacity of an ecosystem to support and maintain ecological processes and biodiversity (content and structure).

Ecosystem structure – the network of biotic and abiotic components making up an ecosystem (note in this paper we focus only on the biotic and infer continuance of abiotic connections).

Ecosystem function – the combined set of ecological processes controlling the flux of matter (including nutrients) and energy through an ecosystem.

Ecosystem health – capacity of the ecosystem to maintain structure and function on ecological and evolutionary time scales.

Functional diversity – the diversity of functional traits (from across the components of an ecosystem) that influence function of an ecosystem (across ecological and evolutionary time scales).

Functional groups – group of species sharing common characteristics (traits such as size, longevity, diet, habitat use) and have a similar role in an ecosystem.

Gao index – a scalar defined based on the position of an ecosystem in a space defined by Network heterogeneity and Network density. If the point representing the ecosystem sits in the area below the collapse horizon (see the main text for the description of how to calculate this transition surface) then it receives the lowest possible score for this semi-quantitative index (0.5 in this application). If it sits in the quadrant defined as being above the point of intersection of the collapse horizon and the x and y axes of the space then the system receives the highest possible score (1). If the point is above one of the intercepts but not both (i.e. is above the collapse horizon

but not in the fully resilient quadrant) then it receives an intermediate score (0.8 in this application).

Green Band – an acceptable band of exploitation pressure that does not put distortive pressure on ecosystem structure, as it matches the biomass-production profile of the unfished/unperturbed state of that ecosystem. If the human induced mortality for those species-groups (e.g. as represented by catch volume) sees them sit within the band they are being fished in a “structurally sustainable” way. If their point sits below the Green Band there is potential to increase the mortality/extraction without imposing structurally distortive pressure via that species group. If the point sits above the Green Band then “structural overfishing” is occurring and pressure should be reduced.

Hub index – minimum of the rank (lowest numerical value) of a species group based on the network indices of degree, degree out and pagerank. Ranks begin at 1 (the highest rank) for the species group with the highest score for that network index and increase to n (where n is the total number of species groups in the network) for the species group with the lowest value of that index. The species with 5% of species groups in the ecosystem with the lowest hub index (effectively the most highly ranked species) are identified as hub species.

Hub species – highly connected species (nodes in the network) that are found to tie the system together structurally and to facilitate trophic flow across a broad part of the system. The loss of these species would lead to bottlenecks or splinter the network degrading its function.

Node – species in an ecological network (or functional groups in less resolved ecological networks), where connections between the nodes may be due to habitat dependency or feeding interactions.

Network density – how many connections a network has versus the total possible. While in some usage it is a normalised value, here it is implemented as the average weighted degree.

Network heterogeneity – a measure of how variable flows are across the network (calculated here based on the variance in the weighted connections in the network).

Network motif – these patterns of interconnections found in complex networks are in effect the structural representation of processes such as predation or competition, as they are defined through the ways nodes interact (directly and indirectly).

Network symmetry – the in–out weighted-degree correlation coefficient (i.e. whether a node with a high inflow has a high outflow).

Pagerank – This index is a variant of the eigenvector centrality concept and is used by companies, such as Google, to rate the importance of websites (to improve search efficiency and web maintenance prioritisation). The index involves counting the number and quality/strength (weight) of links to a node - more important nodes are cross linked with and support more nodes. We use energy flow through the system, as represented by consumption, as the link weights used to calculate this index.

Responsiveness – whether an indicator reflects change quickly.

Sensitivity – whether an indicator provides a clear and interpretable signal of real change.

Specificity – whether an indicator responds only to change due to a specific stressor.

Structural resilience – the capacity of the network structure to continue functioning without degradation when nodes in the system are perturbed.

Symmetry – see network symmetry above.

Topology – how the ecosystem is structured, what is connected to what, and which aspects (connections, species or functional groups) are most important to its ecological integrity.

Tables Used in the Calculation of the Structural Indicators

Table S1: Species classification scheme.

**Classification**	**Description**	**Target relative biomass levels (vs $B_{unfished}$)**	**Weight**
Vulnerable	Slow growing, late maturing species susceptible to fishing pressure (such as marine mammals, seabirds and large sharks)	0.5-0.7	1
Habitat	Biogenic habitat forming species	0.3-0.6	1
Target	Primary target species of a fishery	0.4-0.5	0.5
Byproduct	These species have some value and are landed by fisheries but are not the main targeted species	0.35-0.4	1
Bycatch	Species that are not retained by the fishery	0.2-0.4	0.1
Robust	Fast growing, short lived species (such as productive invertebrates like cephalopods or microfauna like zooplankton)	0.4-0.5	0.1
Hub	Species identified as being a hub species using the degree and PageRank scores.	0.6-0.7	1

Table S2: Qualitative scoring schemes for position relative to the Green Band and for relative biomass. Default target biomass levels are given in Table S1.

**Green Band scoring system**	**Relative biomass scoring scheme**
Fail: Above Green Band	Fail: below target band
Acceptable: In Green Band	Acceptable: within target band
Light: Below Green Band	Light: above target band

Table S3: Matrix of possible combinations of fail (F), light (L) and acceptable (A) values of the Green Band (GB) and relative biomass (RelB) and the associated "combination score" (κ for use in equation 4), for different categories of species.

**GB-RelB**	**Vulnerable**	**Habitat**	**Target**	**Byproduct**	**Bycatch**	**Robust**	**Hub**	**κ***
**F-F**								0
**F-A**								0.25
**F-L**								0.5
**A-F**								0.375
**A-A**								1
**A-L**								1.5
**L-F**								0.75
**L-A**								2
**L-L**								2.5

* Note there is an intentional asymmetry in the □□values (so a F-A gets a lower □□value than a A-F), this is because removing distortive pressure is more immediately important than the relative biomass (which can be environmentally influenced by interannual variation); acting to remove pressure will also benefit relative biomass in the longer term.

Table S4: The ETI Qualitative Rating system. The break points used in the qualitative rating are roughly regular (with a small deviation in the Shocks likely band, which is slightly broader than other bands) to avoid any strong non-linearities in the rating system. In contrast, the breakpoints the simple qualitative "Management Guidance" bands is nonlinear and is based on the dynamic responses seen in the simulated ecosystems as they transitioned through the different ETI values.

**ETI value**	**Qualitative Rating**		**Management Guidance**
< 2.5	Collapse likely		**Urgent Intervention:** *Degraded structure and function*
2.5 – 3.7	Shocks likely		Requires immediate large-scale action; likely to take considerable time to recover
3.7 – 4.6	Shocks possible		**Act:** *At risk*
4.6 – 5.5	Low integrity		Requires attention; should respond rapidly to intervention
5.5 – 6.4	Medium integrity		**Monitor:** *Still stable*
6.4 – 7.3	Medium-High integrity		Continue monitoring; no changes required
7.3 – 8.2	High integrity		**Stable:** *Not at risk*
8.2 – 9.1	Very robust integrity		Continue monitoring; potential for increased activity (exploitation)
9.1 – 10	Close to pristine		
> 10	Pristine		

Information on Case studies

Table S5: Details of the case studies.

Attribute	Eastern Bering Sea	North central Chile	Kerala, India	Southeast Australia
Ecosystem type	Subpolar	Upwelling	Tropical	Temperate
Spatial Extent	500km from Alaskan coast<br>54°15’ to 65°30’N	Chile EEZ<br>24°S to 32°10’S	Indian EEZ off Kerala state<br>8°S to 13°N	Australian EEZ<br>24°29’S to 49°S<br>115°08’E to 164°E
Historical period considered	1971-2017	2006-2018	1985-2019	1985-2019

Tables of results for Test 1

Table S6: Hub species for two variants of southeast Australian EwE under F-forcing (i.e. depletion levels). Names in *italics* are marginal (on the cut off point for the index) and those in bold are groups consistently rated as hub species across the different F levels.

F_{MMSY} Scalar	Parameterisation 1 Hub Species		Parameterisation 2 Hub Species	
0	**Squid**<br>Pelagic sharks<br>Demersal sharks<br>Pelagic prawns	***Mesopelagics***<br>***Toothed whales***<br>***Euphausiids***	Squid<br>Pelagic sharks<br>Demersal sharks<br>Pelagic prawns	***Mesopelagics***<br>***Toothed whales***<br>***Euphausiids***
0.02	As for F = 0		As for F = 0	
0.05	As for F = 0		As for F = 0	
0.1	As for F = 0		As for F = 0	
0.2	As for F = 0		As for F = 0	
0.5	As for F = 0		As for F = 0	
0.65	As for F = 0		Squid<br>Demersal sharks<br>Macrobenthos<br>***Mesopelagics***	***Toothed whales***<br>***Euphausiids***<br>*Primary producers*<br>*Megabenthos*
1	As for F = 0		As for 0.65xF_{MMSY}	
1.5	**Squid**<br>Demersal sharks<br>Macrobenthos<br>***Mesopelagics***	***Toothed whales***<br>***Euphausiids***<br>*Primary producers*<br>*Megabenthos*	Squid<br>Demersal sharks<br>***Mesopelagics***	***Toothed whales***<br>***Euphausiids***<br>*Large Pelagic Predator*
2	**Squid**<br>Demersal sharks<br>**Mesopelagics**<br>**Euphausiids**	***Toothed whales***<br>*Primary producers*<br>*Seals*	Squid<br>**Mesopelagics**<br>**Euphausiids**<br>Whiting	***Toothed whales***<br>*Large Pelagic Predator*<br>*Cardinal fish*<br>*Blue grenadier*<br>*Small Zooplankton*
5	**Squid**<br>Demersal sharks<br>**Mesopelagics**<br>**Euphausiids**	***Toothed whales***<br>*Primary producers*<br>*Shelf Medium Predator*	**Euphausiids**<br>**Toothed whales**	*Macroalgae*<br>*Large Pelagic Predator*
10	**Squid**<br>**Mesopelagics**<br>Pelagic prawns	**Euphausiids**<br>***Toothed whales***<br>*Shelf Small Predator*	**Mesopelagics**<br>**Euphausiids**<br>**Toothed whales**	*Large Pelagic Predator*<br>*Macroalgae*<br>*Baleen whales*
20	**Squid**<br>**Mesopelagics**<br>Pelagic prawns<br>**Euphausiids**	***Toothed whales***<br>*Shelf Small Predator*<br>*Large Pelagic Predator*<br>*Cardinal fish*	As for 10xF_{MMSY}	

Table S7: Green Band and Gao scores for parameterisation 1 (P1) and 2 (P2) of southeast Australian EwE under F forcing (depletion levels). For the Green Band the number of species/groups in the model classified as Acceptable, Light or Fail are shown. For the Gao score it is the score for the entire system – Resilient, Partial or Not-Resilient (collapse likely).

	Vulnerable		Habitat		Target		Byproduct		Bycatch		Robust		Hub		Gao R	
F_{MMSY} Scalar	P1	P2	P1	P2	P1	P2	P1	P2	P1	P2	P1	P2	P1	P2	P1	P2
0	A: 0<br>L: 5<br>F: 0	A: 0<br>L: 5<br>F: 0	A: 0<br>L: 2<br>F: 0	A: 0<br>L: 2<br>F: 0	A: 0<br>L: 23<br>F: 0	A: 0<br>L: 23<br>F: 0	A: 0<br>L: 7<br>F: 0	A: 0<br>L: 7<br>F: 0	A: 0<br>L: 2<br>F: 0	A: 0<br>L: 2<br>F: 0	A: 0<br>L: 11<br>F: 0	A: 0<br>L: 11<br>F: 0	A: 0<br>L: 7<br>F: 0	A: 0<br>L: 7<br>F: 0	R	R
0.02	A: 2<br>L: 2<br>F: 1	A: 3<br>L: 2<br>F: 0	A: 0<br>L: 2<br>F: 0	A: 0<br>L: 2<br>F: 0	A: 10<br>L: 13<br>F: 0	A: 10<br>L: 13<br>F: 0	A: 3<br>L: 4<br>F: 0	A: 3<br>L: 4<br>F: 0	A: 0<br>L: 2<br>F: 0	A: 0<br>L: 2<br>F: 0	A: 1<br>L: 10<br>F: 0	A: 2<br>L: 9<br>F: 0	A: 2<br>L: 5<br>F: 0	A: 1<br>L: 5<br>F: 1	R	R
0.05	A: 4<br>L: 0<br>F: 1	A: 3<br>L: 1<br>F: 1	A: 0<br>L: 2<br>F: 0	A: 1<br>L: 1<br>F: 0	A: 17<br>L: 5<br>F: 1	A: 22<br>L: 1<br>F: 0	A: 5<br>L: 1<br>F: 1	A: 6<br>L: 1<br>F: 0	A: 0<br>L: 2<br>F: 0	A: 0<br>L: 2<br>F: 0	A: 2<br>L: 9<br>F: 0	A: 4<br>L: 7<br>F: 0	A: 1<br>L: 4<br>F: 2	A: 1<br>L: 4<br>F: 2	R	R
0.1	A: 3<br>L: 0<br>F: 2	A: 3<br>L: 1<br>F: 1	A: 0<br>L: 2<br>F: 0	A: 1<br>L: 1<br>F: 0	A: 16<br>L: 4<br>F: 3	A: 20<br>L: 1<br>F: 2	A: 5<br>L: 1<br>F: 1	A: 5<br>L: 0<br>F: 2	A: 0<br>L: 2<br>F: 0	A: 0<br>L: 2<br>F: 0	A: 3<br>L: 8<br>F: 0	A: 7<br>L: 4<br>F: 0	A: 1<br>L: 4<br>F: 2	A: 3<br>L: 2<br>F: 2	R	R
0.2	A: 3<br>L: 0<br>F: 2	A: 2<br>L: 1<br>F: 2	A: 0<br>L: 2<br>F: 0	A: 1<br>L: 1<br>F: 0	A: 16<br>L: 3<br>F: 4	A: 20<br>L: 1<br>F: 2	A: 5<br>L: 1<br>F: 1	A: 5<br>L: 0<br>F: 2	A: 0<br>L: 2<br>F: 0	A: 1<br>L: 1<br>F: 0	A: 3<br>L: 8<br>F: 0	A: 8<br>L: 3<br>F: 0	A: 1<br>L: 4<br>F: 2	A: 3<br>L: 2<br>F: 2	R	R
0.5	A: 2<br>L: 0<br>F: 3	A: 3<br>L: 1<br>F: 1	A: 0<br>L: 2<br>F: 0	A: 0<br>L: 1<br>F: 1	A: 14<br>L: 2<br>F: 7	A: 17<br>L: 1<br>F: 5	A: 4<br>L: 1<br>F: 2	A: 3<br>L: 1<br>F: 3	A: 0<br>L: 2<br>F: 0	A: 2<br>L: 0<br>F: 0	A: 3<br>L: 8<br>F: 0	A: 8<br>L: 3<br>F: 0	A: 1<br>L: 4<br>F: 2	A: 4<br>L: 1<br>F: 2	R	R
0.65	A: 2<br>L: 0<br>F: 3	A: 2<br>L: 2<br>F: 1	A: 0<br>L: 2<br>F: 0	A: 0<br>L: 1<br>F: 1	A: 13<br>L: 2<br>F: 8	A: 17<br>L: 1<br>F: 5	A: 2<br>L: 1<br>F: 4	A: 2<br>L: 1<br>F: 4	A: 0<br>L: 2<br>F: 0	A: 2<br>L: 0<br>F: 0	A: 5<br>L: 6<br>F: 0	A: 7<br>L: 3<br>F: 1	A: 1<br>L: 4<br>F: 2	A: 4<br>L: 1<br>F: 2	R	R
1	A: 2<br>L: 0<br>F: 3	A: 2<br>L: 3<br>F: 0	A: 1<br>L: 1<br>F: 0	A: 0<br>L: 1<br>F: 1	A: 11<br>L: 2<br>F: 10	A: 13<br>L: 5<br>F: 5	A: 2<br>L: 1<br>F: 4	A: 2<br>L: 2<br>F: 3	A: 0<br>L: 2<br>F: 0	A: 2<br>L: 0<br>F: 0	A: 6<br>L: 5<br>F: 0	A: 7<br>L: 3<br>F: 1	A: 1<br>L: 4<br>F: 2	A: 4<br>L: 1<br>F: 2	R	R
1.5	A: 2<br>L: 0<br>F: 3	A: 0<br>L: 0<br>F: 5	A: 1<br>L: 1<br>F: 0	A: 0<br>L: 1<br>F: 1	A: 11<br>L: 2<br>F: 10	A: 8<br>L: 0<br>F: 15	A: 2<br>L: 1<br>F: 4	A: 2<br>L: 0<br>F: 5	A: 0<br>L: 2<br>F: 0	A: 1<br>L: 0<br>F: 1	A: 7<br>L: 4<br>F: 0	A: 5<br>L: 3<br>F: 3	A: 0<br>L: 4<br>F: 3	A: 1<br>L: 1<br>F: 5	R	P
2	A: 1<br>L: 0<br>F: 4	A: 0<br>L: 0<br>F: 5	A: 1<br>L: 1<br>F: 0	A: 0<br>L: 0<br>F: 2	A: 11<br>L: 3<br>F: 9	A: 1<br>L: 0<br>F: 22	A: 2<br>L: 1<br>F: 4	A: 1<br>L: 0<br>F: 6	A: 0<br>L: 2<br>F: 0	A: 1<br>L: 0<br>F: 1	A: 7<br>L: 4<br>F: 0	A: 1<br>L: 1<br>F: 9	A: 1<br>L: 4<br>F: 2	A: 1<br>L: 1<br>F: 5	P	NR

	Vulnerable		Habitat		Target		Byproduct		Bycatch		Robust		Hub		Gao R	
F_{MMSY} Scalar	**P1**	**P2**	**P1**	**P2**	**P1**	**P2**	**P1**	**P2**	**P1**	**P2**	**P1**	**P2**	**P1**	**P2**	**P1**	**P2**
5	A: 1<br>L: 1<br>F: 3	A: 0<br>L: 0<br>F: 5	A: 1<br>L: 1<br>F: 0	A: 0<br>L: 0<br>F: 2	A: 4<br>L: 4<br>F: 15	A: 0<br>L: 0<br>F: 23	A: 1<br>L: 1<br>F: 5	A: 0<br>L: 0<br>F: 7	A: 0<br>L: 2<br>F: 0	A: 0<br>L: 0<br>F: 2	A: 5<br>L: 4<br>F: 2	A: 0<br>L: 2<br>F: 9	A: 3<br>L: 2<br>F: 2	A: 0<br>L: 1<br>F: 6	P (but very close to the collapse frontier)	NR
10	A: 0<br>L: 1<br>F: 4	A: 0<br>L: 0<br>F: 5	A: 2<br>L: 0<br>F: 0	A: 0<br>L: 0<br>F: 2	A: 2<br>L: 4<br>F: 17	A: 0<br>L: 0<br>F: 23	A: 0<br>L: 1<br>F: 6	A: 0<br>L: 0<br>F: 7	A: 1<br>L: 1<br>F: 0	A: 0<br>L: 0<br>F: 2	A: 1<br>L: 4<br>F: 6	A: 0<br>L: 2<br>F: 9	A: 4<br>L: 1<br>F: 2	A: 0<br>L: 1<br>F: 6	NR	NR
20	A: 1<br>L: 0<br>F: 4	A: 0<br>L: 0<br>F: 5	A: 1<br>L: 0<br>F: 1	A: 0<br>L: 0<br>F: 2	A: 2<br>L: 3<br>F: 18	A: 0<br>L: 0<br>F: 23	A: 1<br>L: 0<br>F: 6	A: 0<br>L: 0<br>F: 7	A: 2<br>L: 0<br>F: 0	A: 0<br>L: 0<br>F: 2	A: 3<br>L: 4<br>F: 4	A: 0<br>L: 2<br>F: 9	A: 3<br>L: 1<br>F: 3	A: 0<br>L: 1<br>F: 6	NR	NR

Table S8: Summary of system state corresponding to different levels of depletion in the test 1 simulations – relative biomass levels and corresponding green band, Gao resilience and ETI scores.

		F_{MMSY} Scalar												
**Property**		**0**	**0.02**	**0.05**	**0.1**	**0.2**	**0.5**	**0.65**	**1**	**1.5**	**2**	**5**	**10**	**20**
Parameterisation 1														
Relative Biomasses – Proportion of groups > target biomass band														
Vulnerable	(B > 0.7)	1.0	1.0	1.0	1.0	1.0	1.0	1.0	1.0	1.0	0.83	0.5	0.17	0.17
Habitat	(B > 0.6)	1.0	1.0	1.0	1.0	1.0	1.0	1.0	1.0	1.0	0.5	0.0	0.0	0.0
Target	(B > 0.5)	1.0	0.95	0.86	0.86	0.86	0.86	0.82	0.77	0.5	0.41	0.23	0.23	0.14
Byproduct	(B > 0.4)	1.0	1.0	1.0	1.0	1.0	0.9	0.9	0.9	0.8	0.7	0.3	0.3	0.3
Bycatch	(B > 0.4)	1.0	1.0	1.0	1.0	1.0	1.0	1.0	1.0	1.0	1.0	0.5	0.5	0.25
Robust	(B > 0.5)	1.0	1.0	1.0	1.0	1.0	1.0	1.0	1.0	1.0	1.0	0.9	0.5	0.5
Hub	(B > 0.7)	1.0	1.0	1.0	1.0	1.0	1.0	1.0	1.0	0.84	0.67	0.33	0.33	0.16
Relative Biomasses – Proportion of groups within target biomass band														
Vulnerable		0.0	0.0	0.0	0.0	0.0	0.0	0.0	0.0	0.0	0.17	0.17	0.0	0.0
Habitat		0.0	0.0	0.0	0.0	0.0	0.0	0.0	0.0	0.0	0.5	0.5	0.0	0.0
Target		0.0	0.05	0.14	0.14	0.09	0.05	0.09	0.14	0.27	0.09	0	0.0	0.0
Byproduct		0.0	0.0	0.0	0.0	0.0	0.1	0.1	0	0.1	0.0	0.2	0.0	0.0
Bycatch		0.0	0.0	0.0	0.0	0.0	0.0	0.0	0.0	0.0	0.0	0.5	0.25	0.0
Robust		0.0	0.0	0.0	0.0	0.0	0.0	0.0	0.0	0.0	0.0	0.1	0.5	0.3
Hub		0.0	0.0	0.0	0.0	0.0	0.0	0.0	0.0	0.16	0.16	0.34	0.33	0.17
Relative Biomasses – Proportion of groups < target biomass band														
Vulnerable	(B < 0.5)	0.0	0.0	0.0	0.0	0.0	0.0	0.0	0.0	0.0	0.0	0.33	0.83	0.83
Habitat	(B < 0.3)	0.0	0.0	0.0	0.0	0.0	0.0	0.0	0.0	0.0	0.0	0.5	1.0	1.0
Target	(B < 0.4)	0.0	0.0	0.0	0.0	0.05	0.09	0.09	0.09	0.23	0.5	0.77	0.77	0.86
Byproduct	(B < 0.35)	0.0	0.0	0.0	0.0	0.0	0.0	0.0	0.1	0.1	0.3	0.5	0.7	0.7
Bycatch	(B < 0.2)	0.0	0.0	0.0	0.0	0.0	0.0	0.0	0.0	0.0	0.0	0.0	0.25	0.75
Robust	(B < 0.4)	0.0	0.0	0.0	0.0	0.0	0.0	0.0	0.0	0.0	0.0	0.0	0.0	0.2
Hub	(B < 0.6)	0.0	0.0	0.0	0.0	0.0	0.0	0.0	0.0	0.0	0.17	0.33	0.34	0.67
Green Band - Proportion of groups < Green Band														
Vulnerable		1.0	0.4	0.0	0.0	0.0	0.0	0.0	0.0	0.0	0..0	0.0	0.2	0.0
Habitat		1.0	1.0	1.0	1.0	1.0	1.0	1.0	0.5	0.5	0.5	0.5	0.0	0.0
Target		1.0	0.57	0.22	0.17	0.13	0.09	0.09	0.09	0.09	0.13	0.17	0.17	0.13
Byproduct		1.0	0.57	0.14	0.14	0.14	0.14	0.14	0.14	0.14	0.14	0.14	0.14	0.0
Bycatch		1.0	1.0	1.0	1.0	1.0	1.0	1.0	1.0	1.0	1.0	1.0	0.5	0.0
Robust		1.0	0.91	0.82	0.73	0.73	0.73	0.55	0.45	0.36	0.36	0.36	0.36	0.36

Hub	1.0	0.71	0.57	0.57	0.57	0.57	0.57	0.57	0.57	0.57	0.29	0.14	0.14
Green Band - Proportion of groups within the Green Band													
Vulnerable	0.0	0.4	0.8	0.6	0.6	0.4	0.4	0.4	0.4	0.2	0.2	0.0	0.2
Habitat	0.0	0..0	0.0	0.0	0.0	0.0	0.0	0.5	0.5	0.5	0.5	1.0	0.5
Target	0.0	0.43	0.74	0.7	0.7	0.61	0.57	0.48	0.48	0.48	0.17	0.09	0.09
Byproduct	0.0	0.43	0.71	0.71	0.71	0.57	0.29	0.29	0.29	0.29	0.14	0.0	0.14
Bycatch	0.0	0.0	0.0	0.0	0.0	0.0	0.0	0.0	0.0	0.0	0.0	0.5	1
Robust	0.0	0.09	0.18	0.27	0.27	0.27	0.45	0.55	0.64	0.64	0.45	0.09	0.27
Hub	0.0	0.29	0.14	0.14	0.14	0.14	0.14	0.14	0,0	0.14	0.43	0.57	0.43
Green Band - Proportion of groups > Green Band													
Vulnerable	0.0	0.2	0.2	0.4	0.4	0.6	0.6	0.6	0.6	0.8	0.6	0.8	0.8
Habitat	0.0	0.0	0.0	0.0	0.0	0.0	0.0	0.0	0.0	0.0	0.0	0.0	0.5
Target	0.0	0.0	0.04	0.13	0.17	0.3	0.35	0.43	0.43	0.39	0.65	0.74	0.78
Byproduct	0.0	0.0	0.14	0.14	0.14	0.29	0.57	0.57	0.57	0.57	0.71	0.86	0.86
Bycatch	0.0	0.0	0.0	0.0	0.0	0.0	0.0	0.0	0.0	0.0	0.0	0.0	0.0
Robust	0.0	0.0	0.0	0.0	0.0	0.0	0.0	0.0	0.0	0.0	0.18	0.55	0.36
Hub	0.0	0.0	0.29	0.29	0.29	0.29	0.29	0.29	0.43	0.29	0.29	0.29	0.43
Gao R	1.0	1.0	1.0	1.0	1.0	1.0	1.0	1.0	1.0	0.8	0.8	0.5	0.5
ETI	10.6	9.2	8.2	8.06	7.19	6.47	6.31	5.54	3.92	2.615	1.775	1.725	1.73
Parameterisation 2													
Relative Biomasses – Proportion of groups > target biomass band													
Vulnerable (B > 0.7)	1.0	1.0	1.0	1.0	0.83	0.0	0.0	0.0	0.0	0.0	0.0	0.0	0.0
Habitat (B > 0.6)	1.0	1.0	1.0	1.0	1.0	1.0	1.0	0.5	0.5	0.5	0.0	0.0	0.0
Target (B > 0.5)	1.0	0.9	0.86	0.86	0.86	0.41	0.18	0.09	0	0.0	0.0	0.0	0.0
Byproduct (B > 0.4)	1.0	1.0	1.0	1.0	0.9	0.4	0.3	0.2	0.1	0.0	0.0	0.0	0.0
Bycatch (B > 0.4)	1.0	1.0	1.0	1.0	1.0	0.75	0.75	0.5	0.25	0.0	0.0	0.0	0.0
Robust (B > 0.5)	1.0	1.0	1.0	1.0	1.0	0.9	0.8	0.5	0.2	0.1	0.0	0.0	0.0
Hub (B > 0.7)	1.0	1.0	1.0	1.0	1.0	0.0	0.0	0.0	0.0	0.0	0.0	0.0	0.0
Relative Biomasses – Proportion of groups within target biomass band													
Vulnerable	0.0	0.0	0.0	0.0	0.17	0.5	0.0	0.0	0.0	0.0	0.0	0.0	0.0
Habitat	0.0	0.0	0.0	0.0	0.0	0.0	0.0	0.5	0.0	0.0	0.0	0.0	0.0
Target	0.0	0.05	0.09	0.09	0.09	0.5	0.59	0.32	0.05	0.0	0.0	0.0	0.0
Byproduct	0.0	0.0	0.0	0.0	0.1	0.5	0.3	0.3	0.1	0.0	0.0	0.0	0.0
Bycatch	0.0	0.0	0.0	0.0	0.0	0.25	0.25	0.25	0	0.0	0.0	0.0	0.0
Robust	0.0	0.0	0.0	0.0	0.0	0.1	0.2	0.4	0.3	0.2	0.0	0.0	0.0
Hub	0.0	0.0	0.0	0.0	0.0	1.0	0.67	0.67	0.33	0.33	0.0	0.0	0.0

Relative Biomasses – Proportion of groups < target biomass band													
Vulnerable (B < 0.5)	0.0	0.0	0.0	0.0	0.0	0.5	1.0	1.0	1.0	1.0	1.0	1.0	1.0
Habitat (B < 0.3)	0.0	0.0	0.0	0.0	0.0	0.0	0.0	0.0	0.5	0.5	1.0	1.0	1.0
Target (B < 0.4)	0.0	0.05	0.05	0.05	0.05	0.09	0.23	0.59	0.95	1.0	1.0	1.0	1.0
Byproduct (B < 0.35)	0.0	0.0	0.0	0.0	0.0	0.1	0.4	0.5	0.8	1.0	1.0	1.0	1.0
Bycatch (B < 0.2)	0.0	0.0	0.0	0.0	0.0	0.0	0.0	0.25	0.75	1.0	1.0	1.0	1.0
Robust (B < 0.4)	0.0	0.0	0.0	0.0	0.0	0.0	0.0	0.1	0.5	0.7	1.0	1.0	1.0
Hub (B < 0.6)	0.0	0.0	0.0	0.0	0.0	0.0	0.33	0.33	0.67	0.67	1.0	1.0	1.0
Green Band - Proportion of groups < Green Band													
Vulnerable	1.0	0.4	0.2	0.2	0.2	0.2	0.4	0.6	0.0	0.0	0.0	0.0	0.0
Habitat	1.0	1.0	0.5	0.5	0.5	0.5	0.5	0.5	0.5	0.0	0.0	0.0	0.0
Target	1.0	0.57	0.04	0.04	0.04	0.04	0.04	0.22	0.0	0.0	0.0	0.0	0.0
Byproduct	1.0	0.57	0.14	0.0	0.0	0.14	0.14	0.29	0.0	0.0	0.0	0.0	0.0
Bycatch	1.0	1.0	1.0	1.0	0.5	0.0	0.0	0.0	0.0	0.0	0.0	0.0	0.0
Robust	1.0	0.82	0.64	0.36	0.27	0.27	0.27	0.27	0.27	0.09	0.18	0.18	0.18
Hub	1.0	0.71	0.57	0.29	0.29	0.14	0.14	0.14	0.14	0.14	0.14	0.14	0.14
Green Band - Proportion of groups within the Green Band													
Vulnerable	0.0	0.6	0.6	0.6	0.4	0.6	0.4	0.4	0.0	0.0	0.0	0.0	0.0
Habitat	0.0	0.0	0.5	0.6	0.4	0.6	0.4	0.4	0.0	0.0	0.0	0.0	0.0
Target	0.0	0.43	0.96	0.5	0.5	0	0	0	0.0	0.0	0.0	0.0	0.0
Byproduct	0.0	0.43	0.86	0.87	0.87	0.74	0.74	0.57	0.35	0.04	0.0	0.0	0.0
Bycatch	0.0	0.0	0.0	0.71	0.71	0.43	0.29	0.29	0.29	0.14	0.0	0.0	0.0
Robust	0.0	0.18	0.36	0.0	0.5	1.0	1.0	1.0	0.5	0.5	0.0	0.0	0.0
Hub	0.0	0.14	0.14	0.64	0.73	0.73	0.64	0.64	0.45	0.09	0.0	0.0	0.0
Green Band - Proportion of groups > Green Band													
Vulnerable	0.0	0.0	0.2	0.2	0.4	0.2	0.2	0.0	1.0	1.0	1.0	1.0	1.0
Habitat	0.0	0.0	0.0	0.0	0.0	0.5	0.5	0.5	0.5	1.0	1.0	1.0	1.0
Target	0.0	0.0	0.0	0.09	0.09	0.22	0.22	0.22	0.65	0.96	1.0	1.0	1.0
Byproduct	0.0	0.0	0.0	0.29	0.29	0.43	0.57	0.43	0.71	0.86	1.0	1.0	1.0
Bycatch	0.0	0.0	0.0	0.0	0.0	0.0	0.0	0.0	0.5	0.5	1.0	1.0	1.0
Robust	0.0	0.0	0.0	0.0	0.0	0.0	0.09	0.09	0.27	0.82	0.82	0.82	0.82
Hub	0.0	0.14	0.29	0.29	0.29	0.29	0.29	0.29	0.71	0.71	0.86	0.86	0.86
Gao R	1.0	1.0	1.0	1.0	1.0	1.0	1.0	1.0	1.0	0.8	0.5	0.5	0.5
ETI	10.6	8.95	8.87	7.83	7.42	4.8	4.08	3.1	0.84	0.215	0.13	0.13	0.13

Table S9: Hub species identified for each case study system. The Hub species listed in italics are marginal (on the cut off point for that index).

**Southeast Australia**	**Chile**	**India**	**Eastern Bering Sea**
Squid	Hake	Squid	Arrowtooth
Demersal sharks	Anchovy	Benthic Carnivores	Euphausiids
Macrobenthos	*Zooplankton*	Large Zooplankton	Copepods
Megabenthos		*Medium Benthic Carnivores shelf*	*Squids*
Mesopelagics			*Benthic Amphipods*
Primary producers			*Alaska Skate*
Euphausiids			

Table S10: Green Band summary for each case study location - the number of species per taxa class above (fail, F), below (light, L) or within the Green Band (acceptable, A).

	**North Central Chile**			**Kerala, India**			**Southeast Australia**			**Eastern Bering Sea**		
**Classification**	**F**	**A**	**L**	**F**	**A**	**L**	**F**	**A**	**L**	**F**	**A**	**L**
**Vulnerable**	1	0	2	2	0	1	3	1	0	16	8	9
**Habitat**	0	0	0	0	0	1	0	0	1	1	1	4
**Target**	3	1	1	16	5	0	7	4	3	7	6	1
**Byproduct**	2	0	0	6	0	3	2	11	7	9	10	8
**Bycatch**	0	0	0	0	0	2	0	2	1	0	1	23
**Robust**	0	0	2	0	0	3	0	0	5	0	1	11
**Hub**	1	1	1	2	1	1	0	1	4	1	1	4

Figure S1: Gao resilience plot based on EwE models for each case study region (Eastern Bering Sea; Kerala, India; north central Chile and southeast Australia) forced with historical fishing effort (and matching historical catch levels).

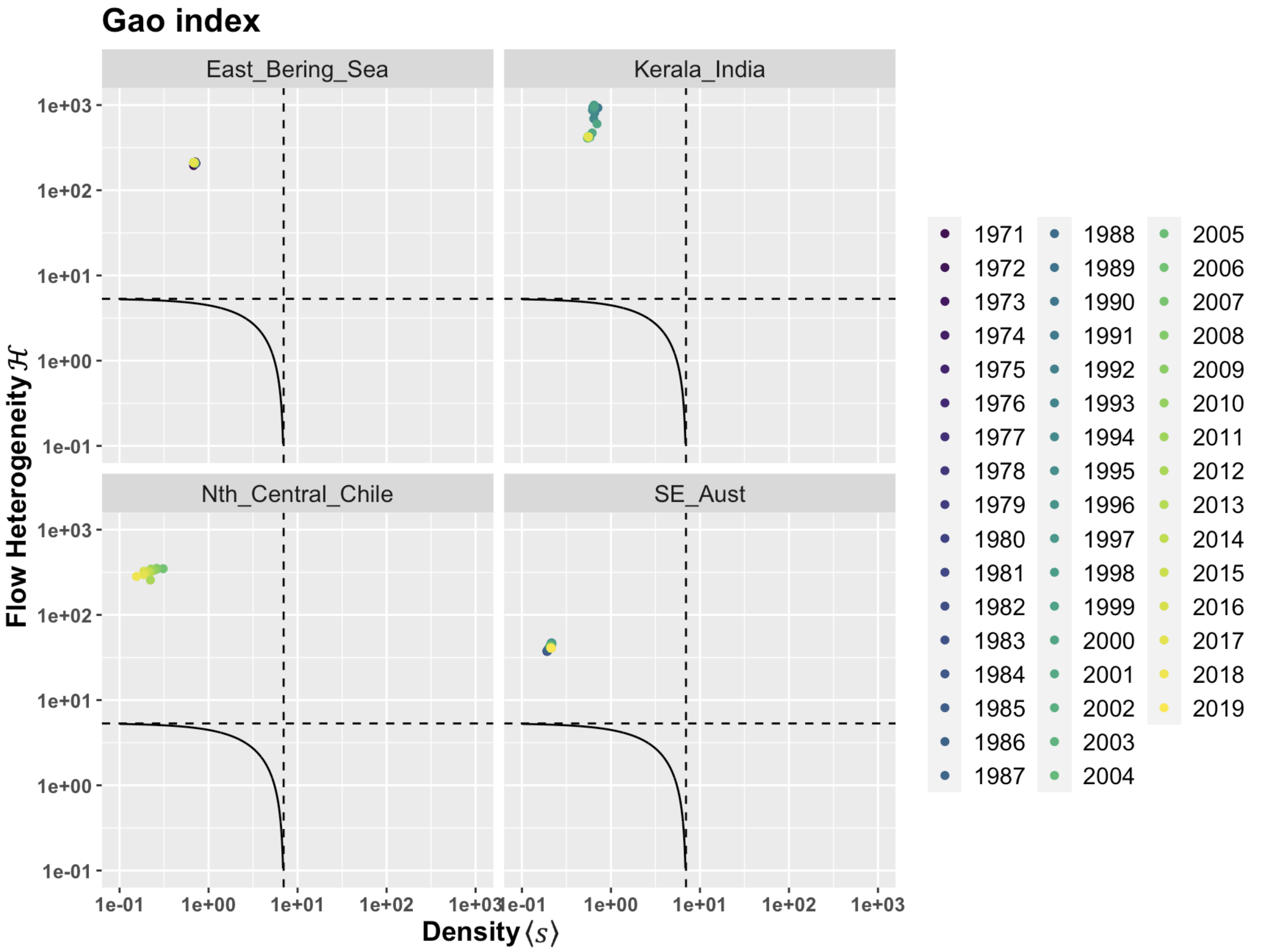

Figure S2: Zoomed Gao resilience plot for the four case study locations forced with historical fishing effort (and matching historical catch levels) for (a), eastern Bering Sea and (b) southeast Australia.

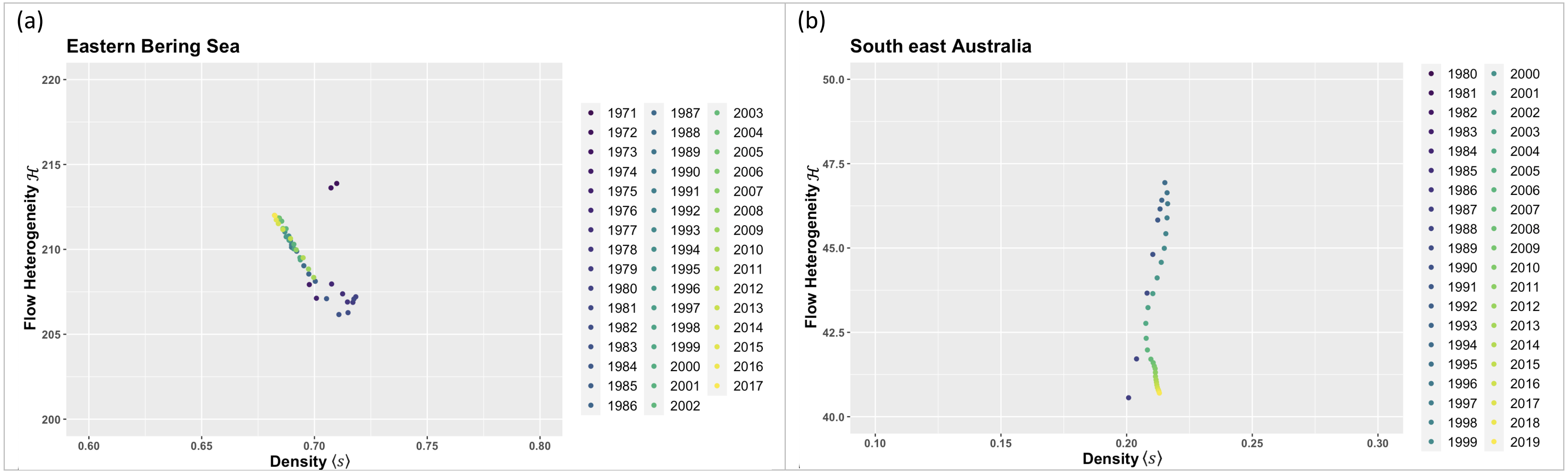

Figure S3: ETI time series based on EwE models for each case study region (Eastern Bering Sea; Kerala, India; north central Chile and southeast Australia) forced with historical fishing effort (and matching historical catch levels).

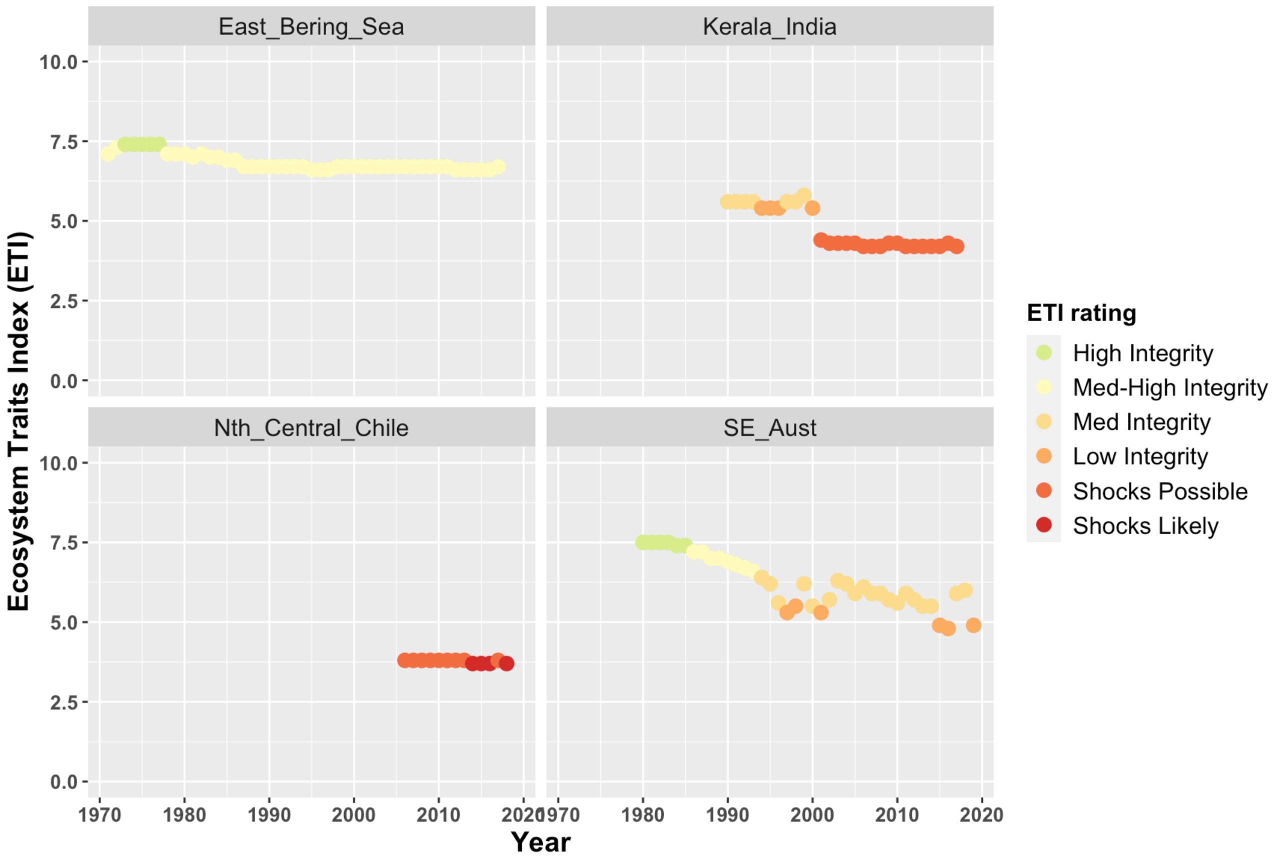

Table S11: 2015-2017 relative biomass values for the test 2 simulations – historical forcing of the models of the four case study regions – and corresponding green band, Gao resilience and ETI scores.

**Property**	**Eastern Bering Sea**	**Kerala, India**	**North central Chile**	**Southeast Australia**
Relative Biomasses – Proportion of groups > target biomass band				
Vulnerable (B > 0.7)	0.67 – 0.87	0.0	0.0	0.67
Habitat (B > 0.6)	0.83	0.0	-	0.0 – 0.5
Target (B > 0.5)	0.6 – 0.67	0.17	0.2 – 0.4	0.13 – 0.23
Byproduct (B > 0.4)	0.81	0.0	1.0	0.18
Bycatch (B > 0.4)	1.0	0.0	-	0.8
Robust (B > 0.5)	1.0	0.1	0.0	0.75
Hub (B > 0.7)	0.83	0.0	0.33	0.67
Relative Biomasses – Proportion of groups within target biomass band				
Vulnerable	0.03 – 0.13	0.0	0.0 – 0.33	0.17
Habitat	0.0	0.0	-	0.0 – 0.5
Target	0.07 – 0.13	0.0 – 0.09	0.0 – 0.2	0.45
Byproduct	0.0	0.15	0.0	0.54 – 0.64
Bycatch	0.0	0.0	-	0.2
Robust	0.0	0.4	0.0	0.125
Hub	0.0 – 0.17	0.0 – 0.5	0.0	0.0
Relative Biomasses – Proportion of groups < target biomass band				
Vulnerable (B < 0.5)	0.09 – 0.13	1.0	0.67 – 1.0	0.17
Habitat (B < 0.3)	0.17	1.0	-	0.0 – 1.0
Target (B < 0.4)	0.2 – 0.33	0.74 – 0.83	0.4 – 0.6	0.32 – 0.41
Byproduct (B < 0.35)	0.19	0.85	0.0	0.18 – 0.27
Bycatch (B < 0.2)	0.0	1.0	-	0.0
Robust (B < 0.4)	0.0	0.5	1.0	0.125
Hub (B < 0.6)	0.0 – 0.17	0.5 – 1.0	0.67	0.33
Green Band - Proportion of groups < Green Band				
Vulnerable	0.27 – 0.33	0.33	0.33 – 0.67	0.0 – 0.33
Habitat	0.67 – 0.83	1.0	-	0.5 – 1.0
Target	0.13 – 0.17	0.0 – 0.3	0.0 – 0.4	0.13 – 0.36
Byproduct	0.3 – 0.48	0.33 – 0.71	0.0 – 0.67	0.25 – 0.35
Bycatch	0.96 – 1.0	0.6	-	0.33 – 0.4
Robust	0.92 – 1.0	0.8	1.0	1.0
Hub	0.67 – 0.83	0.2 – 0.25	0.33	0.33 – 0.8
Green Band - Proportion of groups within the Green Band				
Vulnerable	0.14 – 0.33	0.0	0.0 – 0.33	0.0 – 0.5
Habitat	0.09 – 0.17	0.0	-	0.0 – 0.5
Target	0.27 – 0.67	0.24 – 0.41	0.0 – 0.2	0.28 – 0.63
Byproduct	0.26 – 0.37	0.0 – 0.28	0.0	0.42 – 0.55
Bycatch	0.0 – 0.04	0.4	-	0.4 – 0.67
Robust	0.0 – 0.08	0.2	0.0	0.0
Hub	0.09 – 0.17	0.25 – 0.4	0.0 – 0.33	0.2 – 0.33
Green Band - Proportion of groups > Green Band				
Vulnerable	0.34 – 0.51	0.67	0.33 – 0.67	0.5 – 0.75
Habitat	0.09 – 0.17	0.0	-	0.0
Target	0.27 – 0.6	0.29 – 0.76	0.2 – 0.6	0.23 – 0.5
Byproduct	0.19 – 0.33	0.0 – 0.67	1.0	0.1 – 0.33
Bycatch	0.0	0.0	0.0 – 1.0	0.0 – 0.2
Robust	0.0	0.0	-	0.0
Hub	0.0 – 0.17	0.4 – 0.5	0.33	0.0 – 0.33
Gao R	0.8	0.8	0.8	0.8
ETI	6.6 – 6.7	4.2 – 4.3	3.7 – 3.8	4.9 – 5.9

Table S12: Green Band summary (median over the last five years of the projection) for each case study for scenario - the number of species per taxa class above (fail, F), below (light, L) or within the Green Band (acceptable, A). Numbers in bold are where the values differ to the historical values given in Table S9.

**Scenario**		**North Central Chile**			**Kerala, India**			**Southeast Australia**			**Eastern Bering Sea**		
**Climate change**	**Classification**	**F**	**A**	**L**	**F**	**A**	**L**	**F**	**A**	**L**	**F**	**A**	**L**
	**Vulnerable**	1	**1**	**1**	2	0	1	3	1	0	**24**	**0**	9
	**Habitat**	0	0	0	0	0	1	0	0	1	**5**	**0**	**1**
	**Target**	**5**	**0**	**0**	**17**	**4**	0	7	4	3	**8**	**5**	1
	**Byproduct**	2	0	0	6	**1**	**2**	**5**	**8**	7	**10**	**13**	**4**
	**Bycatch**	0	0	0	0	0	2	0	2	1	**6**	**4**	**14**
	**Robust**	0	0	2	0	**2**	**1**	0	0	5	**2**	**0**	**10**
	**Hub**	**2**	1	**0**	**3**	1	**0**	0	1	4	**2**	**1**	**3**
**Climate change with marine heatwaves**	**Classification**	**F**	**A**	**L**	**F**	**A**	**L**	**F**	**A**	**L**	**F**	**A**	**L**
	**Vulnerable**	**2**	0	**1**	2	0	1	3	1	0	**27**	**0**	**6**
	**Habitat**	0	0	0	0	0	1	0	0	1	**5**	**0**	**1**
	**Target**	**5**	**0**	**0**	**18**	**3**	**0**	**10**	**2**	**2**	**10**	**3**	**1**
	**Byproduct**	2	0	0	6	**2**	**1**	**7**	**9**	**4**	**13**	**10**	**4**
	**Bycatch**	0	0	0	0	0	2	**1**	**0**	1	**7**	**3**	**14**
	**Robust**	**1**	0	**1**	0	**2**	**1**	0	0	5	**2**	**0**	**10**
	**Hub**	**2**	1	**0**	**3**	1	**0**	0	1	4	**2**	**2**	**2**
**Reduction in fishing pressure**	**Classification**	**F**	**A**	**L**	**F**	**A**	**L**	**F**	**A**	**L**	**F**	**A**	**L**
	**Vulnerable**	1	0	2	2	0	1	3	1	0	**14**	**10**	9
	**Habitat**	0	0	0	0	0	1	0	0	1	1	**2**	**3**
	**Target**	**1**	**2**	1	**14**	**7**	0	**5**	**6**	3	7	**5**	**2**
	**Byproduct**	1	1	0	**3**	**3**	3	2	**10**	**8**	**8**	**11**	8
	**Bycatch**	0	0	0	0	0	2	0	**1**	**2**	0	1	23
	**Robust**	0	0	2	0	0	3	0	0	5	0	1	11
	**Hub**	1	**0**	**2**	**1**	**2**	1	0	1	4	1	1	4

Figure S4: Gao resilience plot for eastern Bering Sea for the 3 future scenarios.

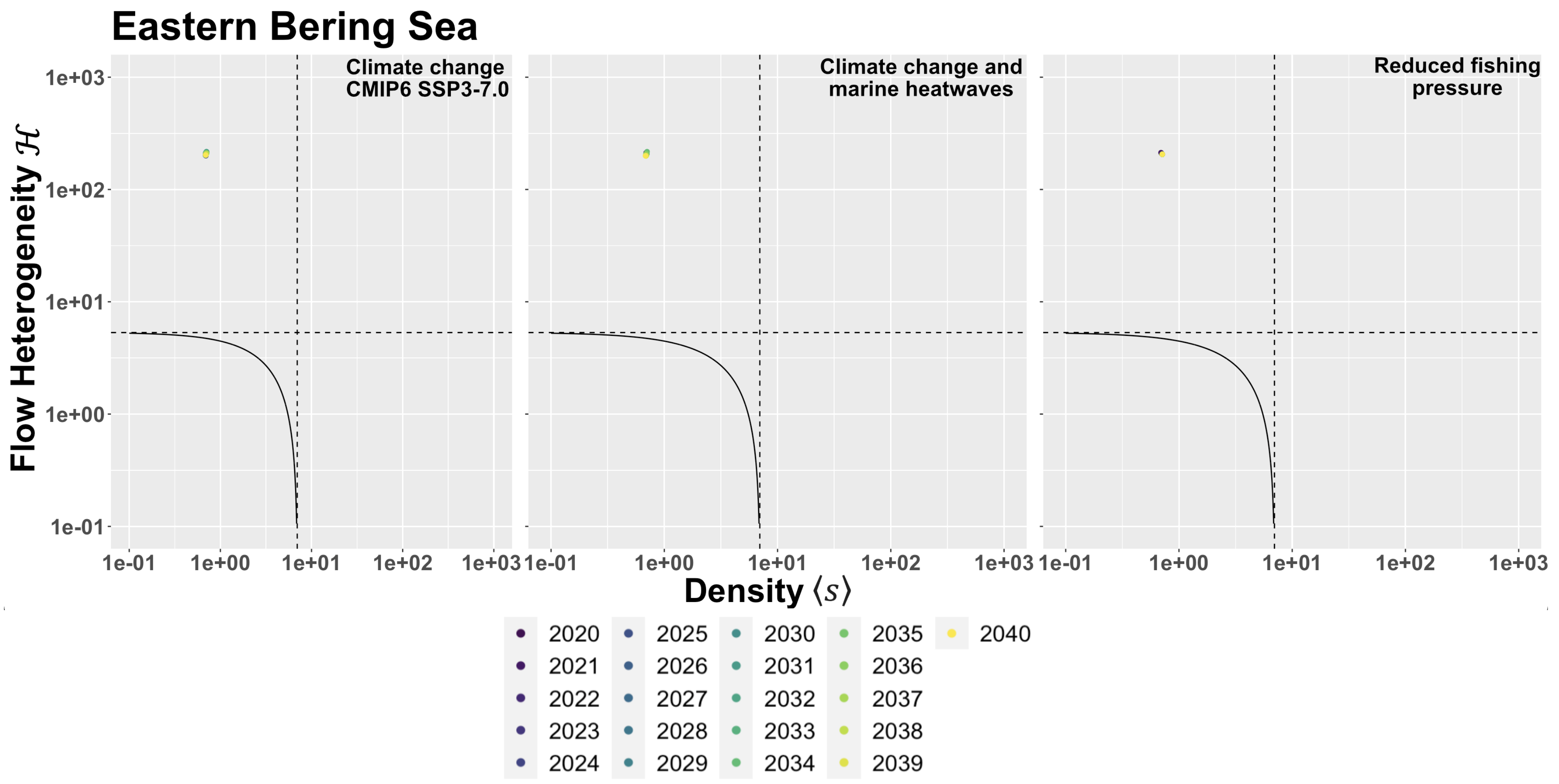

Figure S5: Gao resilience plot for Kerala, India, for the 3 future scenarios.

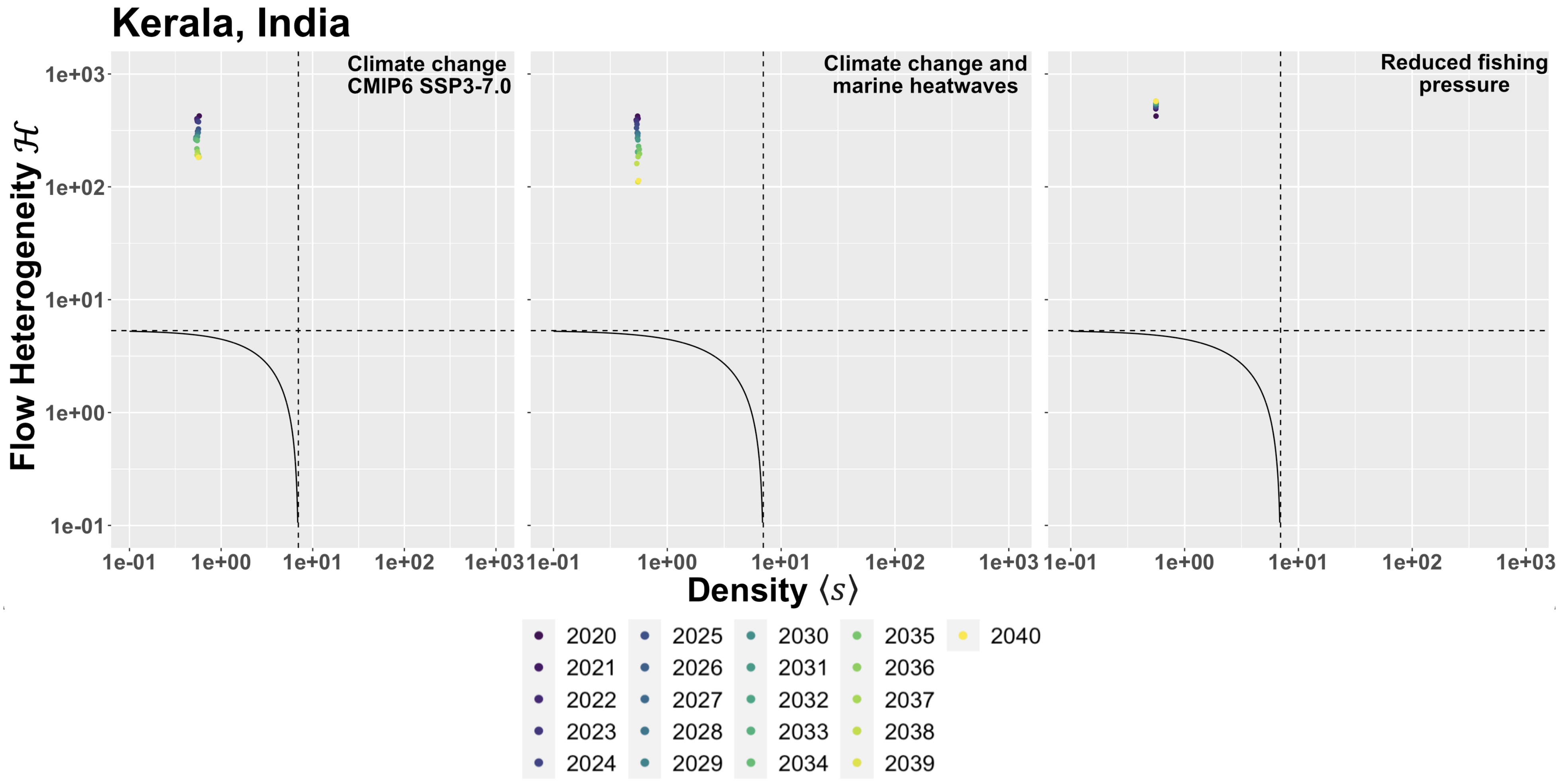

Figure S6: Gao resilience plot for North Central Chile for the 3 future scenarios.

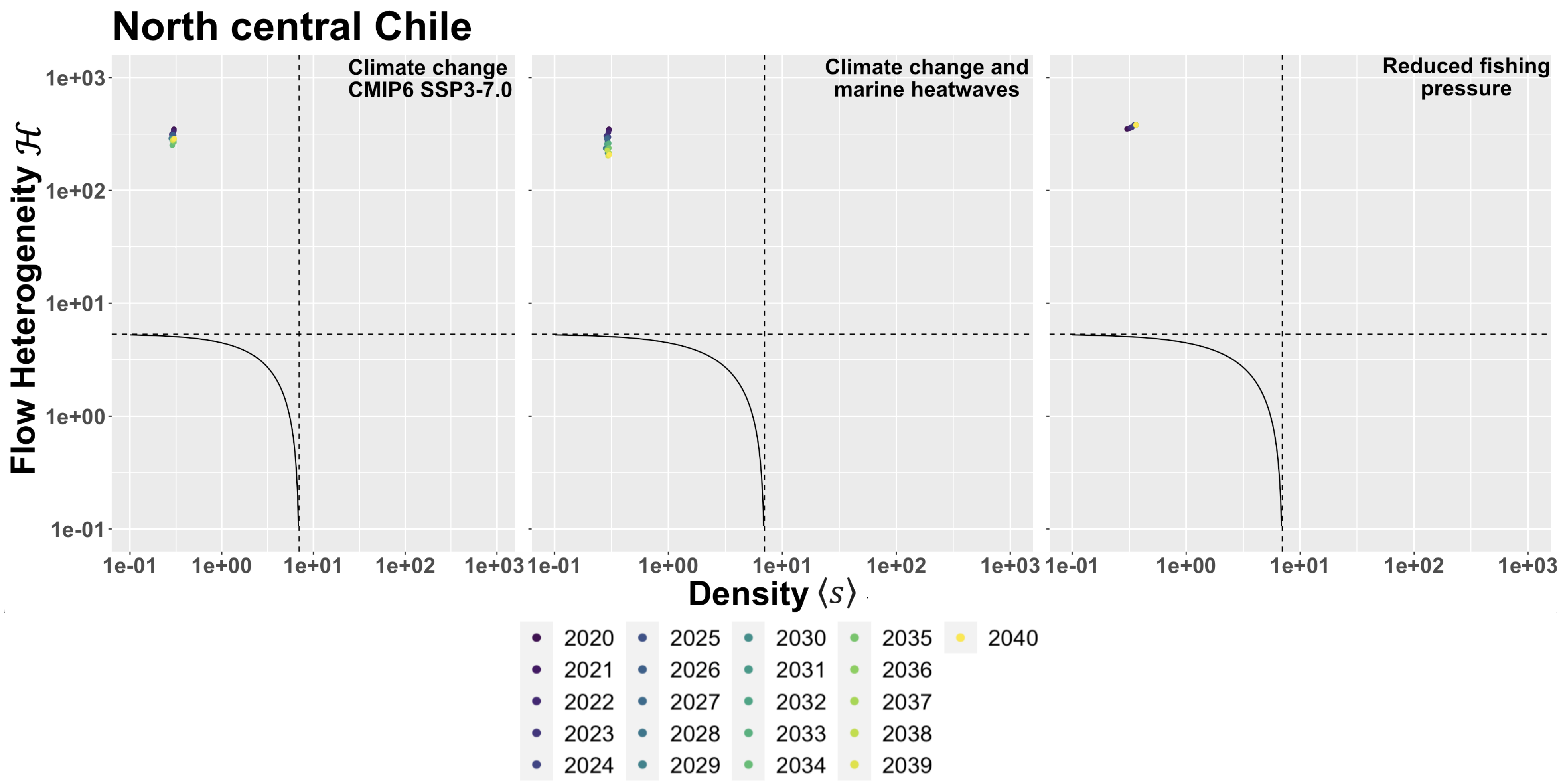

Figure S7: Gao resilience plot for southeast Australia for the 3 future scenarios.

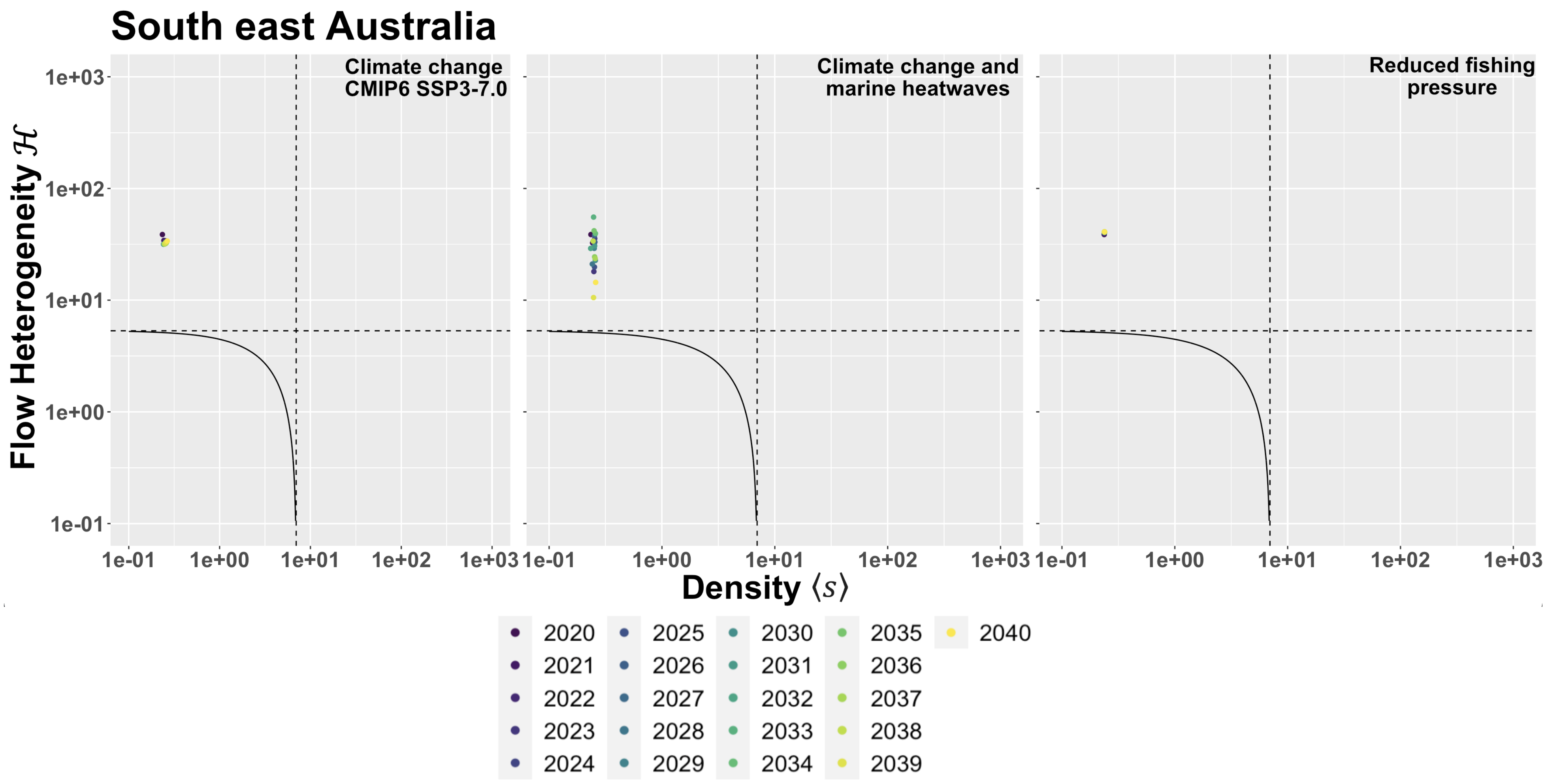

Figure S8: ETI time series for each case study for the 3 future scenarios.

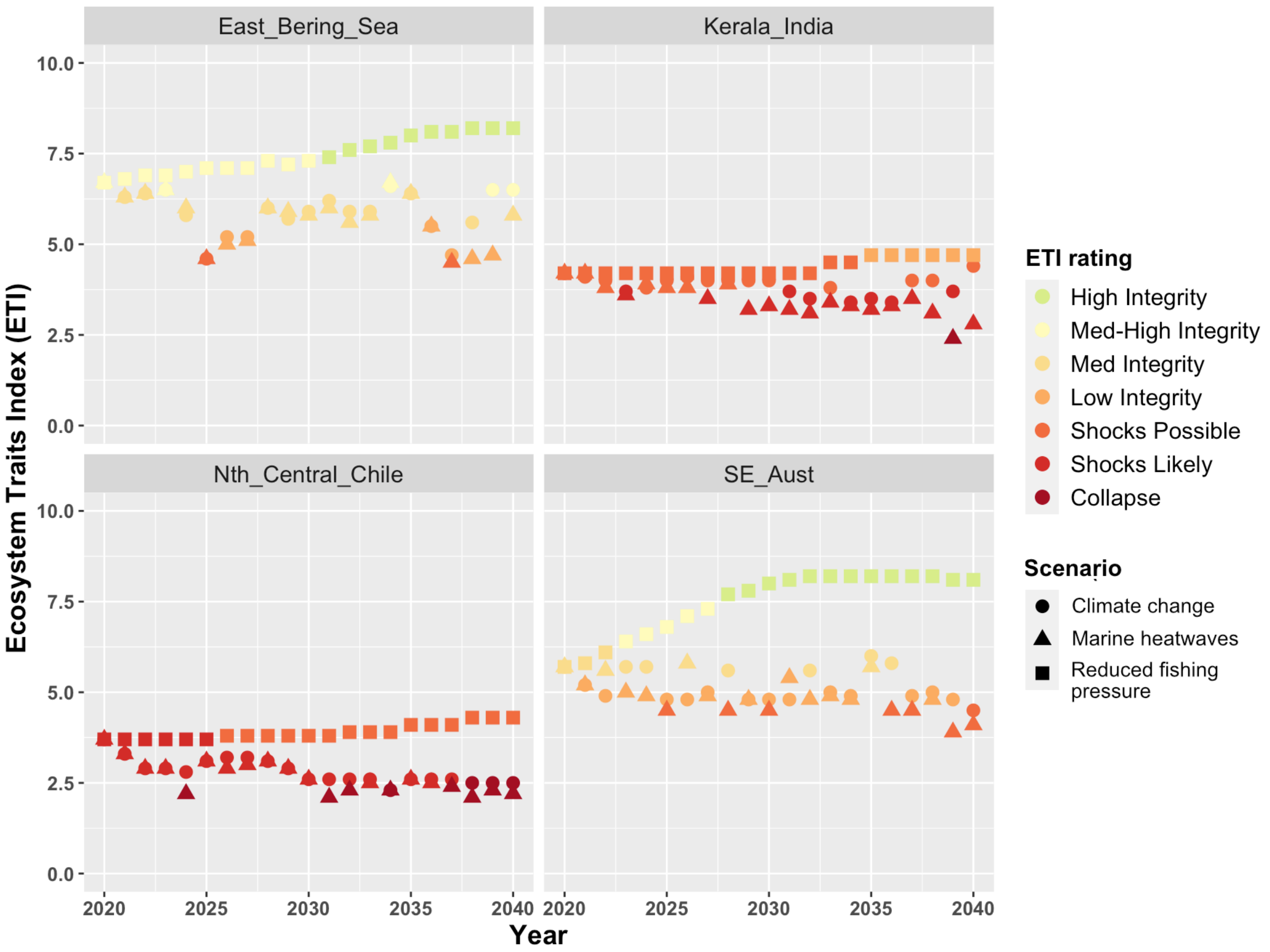

Table S13: 2035-2040 Relative biomass values for each model corresponding to different levels of depletion in the test 3 simulations and corresponding green band, Gao resilience and ETI scores.

(a) Eastern Bering Sea

**Property**	**Climate change (CC)**	**CC + marine heatwaves**	**Reduced fishing**
Relative Biomasses – Proportion of groups > target biomass band			
Vulnerable (B > 0.7)	0.91	0.61 – 0.69	0.93 – 1.0
Habitat (B > 0.6)	1.0	0.5 – 1.0	0.93 – 1.0
Target (B > 0.5)	0.73 – 0.8	0.67 – 0.8	0.8 – 1.0
Byproduct (B > 0.4)	1.0	0.93 – 1.0	0.91 – 1.0
Bycatch (B > 0.4)	1.0	0.96 – 1.0	1.0
Robust (B > 0.5)	1.0	0.69 – 1.0	1.0
Hub (B > 0.7)	0.83 – 1.0	0.33 – 1.0	0.93 – 1.0
Relative Biomasses – Proportion of groups within target biomass band			
Vulnerable	0.03 – 0.09	0.1 – 0.18	0.0 – 0.07
Habitat	0.0	0.0 – 0.5	0.0 – 0.07
Target	0.07 – 0.13	0.0 – 0.07	0.0 – 0.2
Byproduct	0.0	0.0 – 0.04	0.0 – 0.09
Bycatch	0.0	0.0 – 0.04	0.0
Robust	0.0	0.0 – 0.15	0.0
Hub	0.0 – 0.17	0.0 – 0.17	0.0 – 0.07
Relative Biomasses – Proportion of groups < target biomass band			
Vulnerable (B < 0.5)	0.07 – 0.09	0.21	0.0
Habitat (B < 0.3)	0.0	0.0 – 0.17	0.0
Target (B < 0.4)	0.13 – 0.2	0.2 – 0.27	0.0
Byproduct (B < 0.35)	0.0	0.0 – 0.04	0.0
Bycatch (B < 0.2)	0.0	0.0 – 0.04	0.0
Robust (B < 0.4)	0.0	0.0 – 0.16	0.0
Hub (B < 0.6)	0.0	0.0 – 0.67	0.0
Green Band - Proportion of groups < Green Band			
Vulnerable	0.27 – 0.33	0.27 – 0.33	0.27 – 0.33
Habitat	0.67 – 0.83	0.67 – 0.83	0.67 – 0.83
Target	0.13 – 0.17	0.13 – 0.2	0.17 – 0.21
Byproduct	0.3 – 0.44	0.3 – 0.41	0.44 – 0.48
Bycatch	0.96 – 1.0	0.96 – 1.0	1.0
Robust	0.92 – 1.0	0.92 – 1.0	0.92 – 1.0
Hub	0.67 – 0.83	0.67 – 0.83	0.67 – 0.83
Green Band - Proportion of groups within the Green Band			
Vulnerable	0.14 – 0.33	0.0 – 0.12	0.24 – 0.33
Habitat	0.09 – 0.17	0.09 – 0.17	0.17 – 0.21
Target	0.27 – 0.47	0.27 – 0.47	0.47 – 0.67
Byproduct	0.22 – 0.26	0.18 – 0.26	0.22 – 0.33
Bycatch	0.0 – 0.04	0.0 – 0.04	0.0 – 0.04
Robust	0.0 – 0.08	0.0 – 0.08	0.0 – 0.08
Hub	0.09 – 0.17	0.09 – 0.17	0.09 – 0.17
Green Band - Proportion of groups > Green Band			
Vulnerable	0.34 – 0.58	0.43 – 0.72	0.48 – 0.58
Habitat	0.09 – 0.17	0.09 – 0.17	0.17 – 0.27
Target	0.27 – 0.4	0.33 – 0.53	0.4 – 0.67
Byproduct	0.19 – 0.33	0.33 – 0.44	0.29 – 0.33
Bycatch	0.0	0.0	0.0
Robust	0.0	0.0	0.0
Hub	0.0 – 0.17	0.0 – 0.17	0.0
Gao R	0.8	0.8	0.8
ETI	4.7 – 6.5	4.7 – 6.4	8.0 – 8.2

(b) Kerala, India

**Property**	**Climate change (CC)**	**CC + marine heatwaves**	**Reduced fishing**
Relative Biomasses – Proportion of groups > target biomass band			
Vulnerable (B > 0.7)	0.0	0.0	0.0
Habitat (B > 0.6)	0.03 – 0.05	0.03 – 0.05	0.1
Target (B > 0.5)	0.1 – 0.18	0.0 – 0.08	0.28
Byproduct (B > 0.4)	0.0	0.0	0.0 – 0.3
Bycatch (B > 0.4)	0.0	0.0	0.0 – 0.5
Robust (B > 0.5)	0.0 – 0.05	0.0 – 0.05	0.1
Hub (B > 0.7)	0.0	0.0	0.6
Relative Biomasses – Proportion of groups within target biomass band			
Vulnerable	0.0 – 0.03	0.0 – 0.03	0.67
Habitat	0.05 – 0.09	0.05 – 0.09	0.4 – 0.6
Target	0.2 – 0.35	0.2 – 0.3	0.48
Byproduct	0.0 – 0.08	0.0 – 0.08	0.25 – 0.7
Bycatch	0.0	0.0	0.0 – 0.5
Robust	0.3	0.3	0.7
Hub	0.0 – 0.3	0.0 – 0.2	0.0
Relative Biomasses – Proportion of groups < target biomass band			
Vulnerable (B < 0.5)	0.3 – 0.33	0.3 – 0.33	0.33
Habitat (B < 0.3)	0.86 – 0.97	0.88 – 0.97	0.3 – 0.5
Target (B < 0.4)	0.47 – 0.7	0.62 – 0.8	0.24
Byproduct (B < 0.35)	0.92 – 1.0	0.92 – 1.0	0.0 – 0.45
Bycatch (B < 0.2)	1.0	1.0	0.5
Robust (B < 0.4)	0.65 – 0.7	0.65 – 0.7	0.2
Hub (B < 0.6)	0.7 – 0.9	0.8 – 1.0	0.4
Green Band - Proportion of groups < Green Band			
Vulnerable	0.33	0.33	0.33
Habitat	1.0	1.0	1.0
Target	0.0 – 0.04	0.0 – 0.04	0.08
Byproduct	0.52 – 0.57	0.42 – 0.57	0.57
Bycatch	0.6	0.5	0.65
Robust	0.8	0.6	0.85
Hub	0.0	0.0	0.2
Green Band - Proportion of groups within the Green Band			
Vulnerable	0.0	0.0	0.0
Habitat	0.0	0.0	0.0
Target	0.33 – 0.39	0.29 – 0.33	0.29
Byproduct	0.0 – 0.21	0.0 – 0.14	0.29
Bycatch	0.4	0.5	0.35
Robust	0.2	0.4	0.15
Hub	0.25 – 0.4	0.2 – 0.4	0.4
Green Band - Proportion of groups > Green Band			
Vulnerable	0.67	0.67	0.67
Habitat	0.0	0.0	0.0
Target	0.57 – 0.67	0.67 – 0.71	0.63
Byproduct	0.27 – 0.48	0.44	0.14
Bycatch	0.0	0.0	0.0
Robust	0.0	0.0	0.0
Hub	0.6 – 0.75	0.6 – 0.8	0.4
Gao R	0.8 (trending down)	0.8 (trending down)	0.8
ETI	3.5 – 4.4	2.4 – 3.5	4.7

(c) North central Chile

Property	Climate change (CC)	CC + marine heatwaves	Reduced fishing
Relative Biomasses – Proportion of groups > target biomass band			
Vulnerable (B > 0.7)	0.33 – 0.5	0.0	0.33 – 0.4
Habitat (B > 0.6)	-	-	-
Target (B > 0.5)	0.0 – 0.2	0.0	0.2 – 0.3
Byproduct (B > 0.4)	0.2 – 0.5	0.5	0.6 – 0.8
Bycatch (B > 0.4)	-	-	-
Robust (B > 0.5)	0.0	0.0	0.3
Hub (B > 0.7)	0.33	0.33	0.33
Relative Biomasses – Proportion of groups within target biomass band			
Vulnerable	0.0	0.0	0.27 – 0.67
Habitat	-	-	-
Target	0.0	0.0	0.2
Byproduct	0.0	0.0	0.0
Bycatch	-	-	-
Robust	0.0	0.0	0.0
Hub	0.0	0.0	0.17 – 0.34
Relative Biomasses – Proportion of groups < target biomass band			
Vulnerable (B < 0.5)	0.5 – 0.67	1.0	0.0 – 0.33
Habitat (B < 0.3)	-	-	-
Target (B < 0.4)	0.8 – 1.0	1.0	0.5 – 0.6
Byproduct (B < 0.35)	0.5 – 0.8	0.5	0.0 –0.2
Bycatch (B < 0.2)	-	-	-
Robust (B < 0.4)	1.0	1.0	0.7
Hub (B < 0.6)	0.67	0.67	0.3 – 0.5
Green Band - Proportion of groups < Green Band			
Vulnerable	0.67	0.56	0.6 – 0.67
Habitat	-	-	-
Target	0.2	0.2	0.2
Byproduct	0.0	0.0	0.0 – 0.33
Bycatch	-	-	-
Robust	0.5	0.5	1.0
Hub	0.33	0.0 – 0.33	0.33
Green Band - Proportion of groups within the Green Band			
Vulnerable	0.0	0.0	0.0 – 0.2
Habitat	-	-	-
Target	0.4 – 0.6	0.3	0.6
Byproduct	0.0	0.0	0.0 – 0.67
Bycatch	-	-	-
Robust	0.0	0.0	0.0
Hub	0.33	0.33	0.33
Green Band - Proportion of groups > Green Band			
Vulnerable	0.33	0.44	0.2 – 0.33
Habitat	-	-	-
Target	0.2 – 0.4	0.5	0.2
Byproduct	1.0	1.0	0.33
Bycatch	-	-	-
Robust	0.5	0.5	0.0
Hub	0.34	0.67	0.34
Gao R	0.8 (trending down)	0.8 (trending down)	0.8
ETI	2.5 – 2.6	2.1 – 2.6	4.1 – 4.3

(d) Southeast Australia

Property	Climate change (CC)	CC + marine heatwaves	Reduced fishing
Relative Biomasses – Proportion of groups > target biomass band			
Vulnerable (B > 0.7)	0.13 – 0.25	0.25	0.13 – 0.75
Habitat (B > 0.6)	0.0	0.0	0.0 – 0.25
Target (B > 0.5)	0.23 – 0.39	0.15 – 0.28	0.23 – 0.69
Byproduct (B > 0.4)	0.25 – 1.0	0.23 – 0.47	0.38 – 0.62
Bycatch (B > 0.4)	0.8 – 1.0	0.25 – 1.0	0.25 – 1.0
Robust (B > 0.5)	0.8 – 0.9	0.4 – 0.6	0.8 – 1.0
Hub (B > 0.7)	0.6 – 0.8	0.4 – 0.8	0.8 – 1.0
Relative Biomasses – Proportion of groups within target biomass band			
Vulnerable	0.0 – 0.13	0.0 – 0.13	0.0 – 0.13
Habitat	0.0	0.0	0.25 – 0.5
Target	0.07 – 0.31	0 .0 – 0.17	0.07 – 0.37
Byproduct	0.05 – 0.17	0.0 – 0.05	0.05 – 0.47
Bycatch	0.0 – 0.25	0.0 – 0.1	0.0 – 0.25
Robust	0.0 – 0.1	0.0 – 0.2	0.0 – 0.1
Hub	0.0 – 0.2	0.0 – 0.2	0.0 – 0.2
Relative Biomasses – Proportion of groups < target biomass band			
Vulnerable (B < 0.5)	0.63 – 0.75	0.63 – 0.75	0.1 – 0.17
Habitat (B < 0.3)	1.0	1.0	0.0 – 0.25
Target (B < 0.4)	0.31 – 0.77	0.62 – 0.85	0.31 – 0.39
Byproduct (B < 0.35)	0.38 – 0.62	0.47 – 0.76	0.18 – 0.37
Bycatch (B < 0.2)	0.0 – 0.75	0.0 – 1.0	0.0 – 0.1
Robust (B < 0.4)	0.0 – 0.2	0.2 – 0.6	0.0 – 0.2
Hub (B < 0.6)	0.0 – 0.2	0.0 – 0.6	0.0 – 0.1
Green Band - Proportion of groups < Green Band			
Vulnerable	0.4 – 0.5	0.3 – 0.5	0.5 – 0.8
Habitat	1.0	1.0	1.0
Target	0.09 – 0.16	0.09 – 0.31	0.15 – 0.26
Byproduct	0.23 – 0.33	0.2 – 0.28	0.42 – 0.5
Bycatch	0.6 – 0.75	0.55 – 0.75	0.75
Robust	0.9 – 1.0	0.8 – 1.0	1.0
Hub	0.6 – 0.8	0.5 – 0.8	0.8
Green Band - Proportion of groups within the Green Band			
Vulnerable	0.3 – 0.38	0.28 – 0.33	0.25 – 0.38
Habitat	0.0	0.0	0.0
Target	0.59 – 0.65	0.29 – 0.38	0.41 – 0.59
Byproduct	0.48 – 0.67	0.33 – 0.38	0.41 – 0.48
Bycatch	0.25 – 0.4	0.25 – 0.45	0.25
Robust	0.0 – 0.1	0.0 – 0.1	0.0
Hub	0.2 – 0.4	0.2 – 0.5	0.2
Green Band - Proportion of groups > Green Band			
Vulnerable	0.13 – 0.23	0.13 – 0.33	0.0 – 0.13
Habitat	0.0	0.0	0.0
Target	0.19 – 0.31	0.31 – 0.38	0.15
Byproduct	0.10 – 0.19	0.14 – 0.23	0.09
Bycatch	0.0	0.0 – 0.1	0.0
Robust	0.0	0.0	0.0
Hub	0.0	0.0	0.0
Gao R	0.8	0.8 (trending down)	0.8
ETI	6 - 4.5	5.7 - 4.1	8.1 – 8.2

Gulf of Thailand

Table S14: Summary of system level socioecological indicators of different states of the Gulf of Thailand marine ecosystem and fisheries, simulated data from Fulton et al (2022). For each indicator, a higher value is typically considered more desirable. mt is million tonnes.

Time period	Biodiversity	Biological system resilience§	ETI Mean (and range)	Catch composition diversity	Total catch (mt MMSY)	Profitability per unit effort	Total value ($ billion)*	Employment (000)
1950-1964	14.9 – 15.6	2.1 – 2.4	8.65 (7.6 - 9.2) Very robust integrity	7.4 – 13.2	0.21	6.7 – 33.2	0.71 – 1.12	24.7 – 82.2
1965-1974	13.9 – 14.5	1.7 – 1.9	6.52 (5.8 – 7.6) Medium integrity	3.4 – 4.1	0.83	7.4 – 22.7	1.93 – 3.67	35.6 – 170.4
1971-2017	8.3 – 11.4	1.2 – 1.3	4.9 (2.4 – 5.9) Low integrity	2.5 – 2.8	1.50	<0.01 – 5.8	1.62 – 4.85	176.2 – 642.1
1989-2017	7.5 – 10.9	0.9 – 1.1	4.3 (2.4 – 5.4) Shocks possible#	2.4 – 2.5	1.62	<0.01 – 4.7	1.72 – 5.08‡	605.4 – 1097.7

§ Derived from the Gao resilience score, for this ecosystem, the maximum resilience score (for an unfished system) was 2.5-3.0 and the "collapse boundary" was at 0.6-0.8.
* Calculated using 2018 prices
‡ This can be much higher if invertebrates become dominant in the catch
# This period spends a third of the time with shocks or collapse likely

Figure S9: Plot of ETI through time for the Gulf of Thailand based on information reported in Fulton et al (2022).

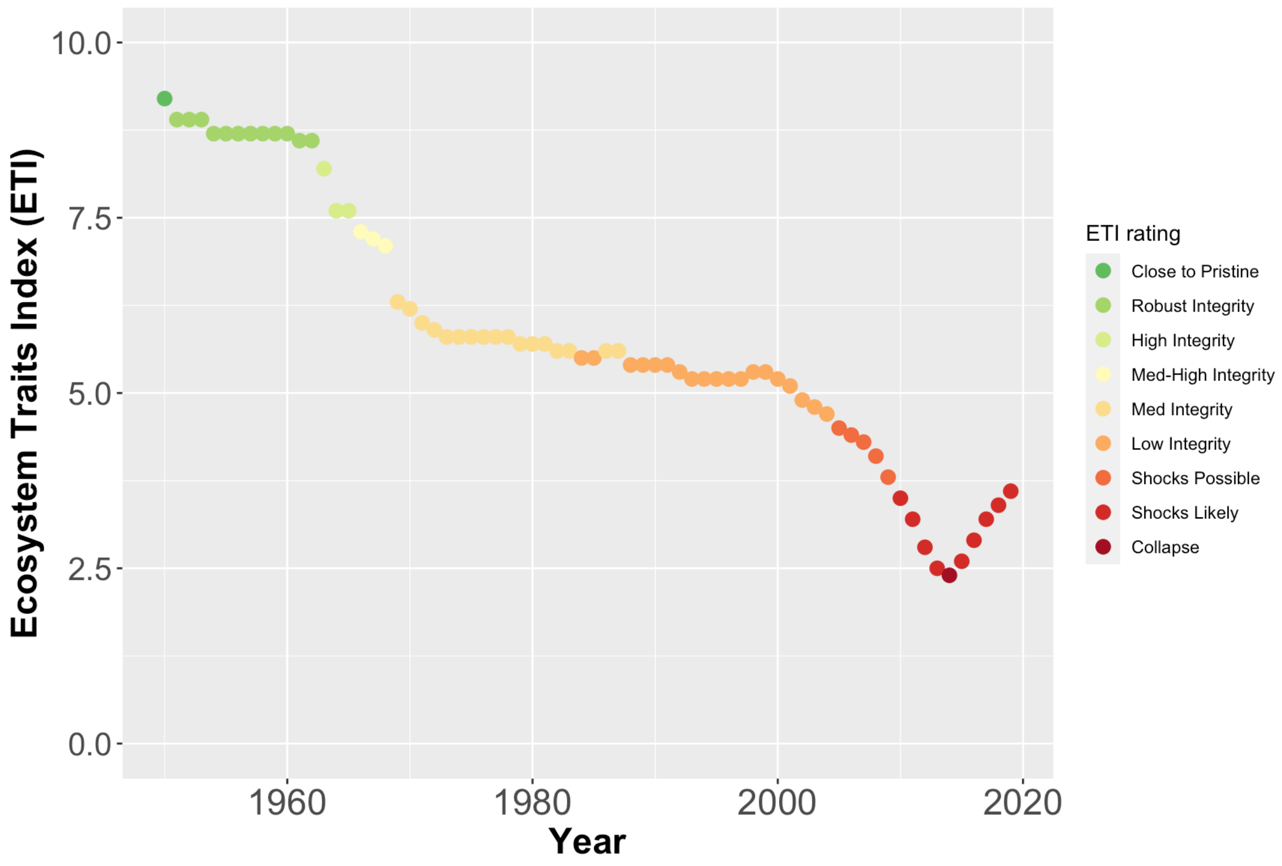

EwE Models

Table S15**:** Ecopath base parameters for the eastern Bering Sea (converted from the Rpath version of the model)

**Group**	**Biomass (tkm^{-2})**	***Mortality* or Production / Biomass (year^{-1})**	**Consumption / Biomass (year^{-1})**	**Ecotrophic Efficiency**	**Biomass Accumulation (tkm^{-2})**	**Biomass Accumulation year^{-1})**
Transient KIllers	0	0.025	11.157	0	0	0
Sperm and Beaked Whales	0.018	0.047	6.609	0	0	0
Resident Killers	0.001	0.025	11.157	0.17	0	0
Porpoises	0.004	0.05	30	0.055	0	0
Belugas	0.012	0.112	30	0.041	0	0
Gray Whales	0.033	0.063	8.873	0.039	0	0
Humpbacks	0.012	0.038	7.577	0.102	0	0
Fin Whales	0.455	0.027	6.517	0.093	0	0
Sei Whales	0.006	0.04	8.788	0.062	0	0
Right Whales	0.004	0.033	8	0.076	0	0
Minke Whales	0.024	0.051	7.782	0.052	0	0
Bowhead Whales	0.007	0.01	8.68	0.247	0	0
Sea Otters	0.001	0.117	73	0.022	0	0
Walrus Bd Seals	0.114	0.051	15.371	0.006	0	0
N Fur Seal Juv	0.001	0.252	82.2	0.021	0	0
N Fur Seal Ad	0.034	0.091	37.716	0.038	0	0
Steller Sea Lions Juv	0	0.194	55.252	0.063	0	0
Steller Sea Lions Ad	0.001	0.11	30.493	0.765	0	0
Resident Seals	0.013	0.083	17.439	0.045	0	0
Wintering Seals	0.03	0.069	19.197	0.87	0	0
Shearwater	0	0.1	73	0.085	0	0
Murres	0.008	0.169	72	0.05	0	0
Kittiwakes	0.001	0.077	110	0.111	0	0
Auklets	0.002	0.169	110	0.05	0	0
Puffins	0	0.04	73	0.213	0	0
Fulmars	0.001	0.055	73	0.154	0	0
Storm Petrels	0	0.12	144	0.07	0	0
Cormorants	0	0.159	73	0.054	0	0
Gulls	0	0.166	73	0.051	0	0
Albatross Jaeger	0	0.068	75	0.126	0	0
Sleeper shark	0.053	0.1	3	0.161	0	0
W. Pollock Juv	4.493	1.453	8.405	0.979	0	0
W. Pollock Ad	22.76	0.667	3.131	0.78	0	0
P. Cod Juv	0.186	1.782	8.828	0.798	0	0
P. Cod Ad	2.815	0.412	2.257	0.462	0	0
Herring Juv	0.171	1.052	7.79	0.804	0	0
Herring Ad	2.734	0.32	2.927	0.163	0	0
Arrowtooth Juv	0.006	0.81	6.139	0.983	0	0
Arrowtooth Ad	1.146	0.18	1.184	0.755	0	0
Kamchatka fl. Juv	0	0.81	6.139	0.034	0	0
Kamchatka fl. Ad	0.019	0.18	1.497	0.559	0	0
Gr. Turbot Juv	0.007	1.701	7.885	0.942	0	0
Gr. Turbot Ad	1.526	0.18	1.081	0.14	0	0
P. Halibut Juv	0.002	2.4	9.303	0.713	0	0
P. Halibut Ad	0.223	0.19	1.096	0.704	0	0
YF. Sole Juv	0.425	0.244	1.531	0.785	0	0

Group	Biomass (tkm^{-2})	*Mortality* or Production / Biomass (year^{-1})	Consumption / Biomass (year^{-1})	Ecotrophic Efficiency	Biomass Accumulation (tkm^{-2})	Biomass Accumulation year^{-1})
YF. Sole Ad	1.764	0.174	0.603	0.991	0	0
FH. Sole Juv	0.147	0.394	4.145	0.85	0	0
FH. Sole Ad	2.091	0.26	1.222	0.126	0	0
N. Rock sole Juv	0.157	0.292	3.482	0.761	0	0
N. Rock sole Ad	3.761	0.232	1.122	0.378	0	0
AK Plaice	1.068	0.2	2	0.52	0	0
Dover Sole	0	0.2	2	0.389	0	0
Rex Sole	0.041	0.2	2	0.475	0	0
Misc. Flatfish	0.222	0.2	2	0.819	0	0
Alaska skate	0.68	0.2	2	0.192	0	0
Other skates	0.093	0.2	2	0.232	0	0
Sablefish Juv	0.004	0.266	2.964	0.954	0	0
Sablefish Ad	0.054	0.19	1.002	0.303	0	0
Eelpouts	2.372	0.4	2	0.946	0	0
Grenadiers	0.968	0.15	2	0.268	0	0
Misc. fish deep	0.008	0.2	2	0.806	0	0
POP	0.167	0.1	2	0.845	0	0
Sharpchin Rock	0.002	0.1	2	0.798	0	0
Northern Rock	0.028	0.1	2	0.798	0	0
Dusky Rock	0.001	0.1	2	0.516	0	0
Shortraker Rock	0.009	0.1	2	0.447	0	0
Rougheye Rock	0.004	0.1	2	0.611	0	0
Shortspine Thorns	0.005	0.15	0.5	0.322	0	0
Other Sebastes	0.011	0.1	2	0.798	0	0
Atka mackerel Juv	0.071	0.9	12.019	0	0	0
Atka mackerel Ad	0.169	0.35	4.991	0.599	0	0
Greenlings	0.001	0.4	2	0.874	0	0
Lg. Sculpins	0.54	0.4	2	0.065	0	0
Other sculpins	1.137	0.4	2	0.88	0	0
Misc. fish shallow	1.168	0.4	2	0.838	0	0
Octopi	0.192	0.8	3.65	0.821	0	0
Squids	0.927	3.2	10.67	0.95	0	0
Salmon returning	0.164	1.65	11.6	0.975	0	0
Salmon outgoing	0.014	1.28	13.56	0.804	0	0
Bathylagidae	0.162	0.8	3.65	0.997	0	0
Myctophidae	0.826	0.8	3.65	0.986	0	0
Capelin	1.245	0.8	3.65	0.817	0	0
Sandlance	2.528	0.8	3.65	0.847	0	0
Eulachon	0.552	0.8	3.65	0.813	0	0
Oth. managed forage	1.051	0.8	3.65	0.839	0	0
Oth. pelagic smelt	0.499	0.8	3.65	0.802	0	0
Bairdi	0.522	1.601	3.092	0.749	0	0
King Crab	0.238	0.659	3.207	0.646	0	0
Opilio	2.179	1.295	3.117	0.524	0	0
Pandalidae	6.742	0.576	2.409	0.938	0	0
NP shrimp	12.702	0.576	2.409	0.806	0	0
Sea stars	2.471	1.21	6.05	0.005	0	0
Brittle stars	2.95	1.21	6.05	0.786	0	0
Urchins dollars cucumbers	1.167	0.61	3.05	0.595	0	0
Snails	0.807	1.81	9.05	0.784	0	0
Hermit crabs	1.774	0.82	4.1	0.79	0	0

Group	Biomass (tkm^{-2})	*Mortality* or Production / Biomass (year^{-1})	Consumption / Biomass (year^{-1})	Ecotrophic Efficiency	Biomass Accumulation (tkm^{-2})	Biomass Accumulation year^{-1})
Misc. crabs	0.71	0.82	4.1	0.814	0	0
Misc. Crustacean	8.886	7.4	37	0.8	0	0
Benthic Amphipods	12.796	7.4	37	0.801	0	0
Anemones	0.11	1	5	0.115	0	0
Corals	0.013	0.046	0.23	0.511	0	0
Hydroids	0.236	1	5	0.799	0	0
Urochordata	0.354	3.58	17.9	0.026	0	0
Sea Pens	0.013	0.092	0.461	0.003	0	0
Sponges	0.054	1	5	0.511	0	0
Bivalves	61.873	1.3	6.5	0.341	0	0
Polychaetes	21.687	2.97	14.85	0.204	0	0
Misc. worms	3.669	2.23	11.15	0.748	0	0
Scyphozoid Jellies	0.338	0.88	3	0.55	0	0
Fish Larvae	0.012	5.475	15.643	0.801	0	0
Chaetognaths	0.705	5.475	15.643	0.832	0	0
Euphausiids	17.831	5.475	15.643	0.905	0	0
Mysids	1.5	5.475	15.643	0.82	0	0
Pelagic Amphipods	1.695	2.5	7.143	0.848	0	0
Gelatinous filter feeders	0.774	5.475	15.643	0.842	0	0
Pteropods	0.232	5.475	15.643	0.795	0	0
Copepods	26.857	6	27.74	0.831	0	0
Pelagic microbes	45	36.5	104.286	0.258	0	0
Benthic microbes	22.061	36.5	104.286	0.8	0	0
Macroalgae	0.7501763	4		0.8000059	0	0
Lg Phytoplankton	4.350356	101.794		0.8	0	0
Sm Phytoplankton	39.5039	110.9194		0.8000001	0	0
Outside Production	4	1		0	0	0
Discards	0.6062543			0.08720814	0	0
Offal	2.054416			0.5810784	0	0
Pelagic Detritus	1			0.8773113	196.8834	196.8834
Benthic Detritus	1			0.92233	267.1387	267.1387
Outside Detritus	1.00E-05			0	0	0

Table S16: Diet matrix for the eastern Bering Sea Ecopath model (converted from the Rpath version of the model)

Group	Transient Killers	Sperm and Beaked Whales	Resident Killers	Porpoises	Belugas	Gray Whales	Humpbacks	Fin Whales	Sei Whales	Right Whales	Minke Whales	Bowhead Whales	Sea Otters	Walrus Bd Seals	N Fur Seal Juv	N Fur Seal Ad	Steller Sea Lions Juv	Steller Sea Lions Ad	Resident Seals	Wintering Seals	Shearwater	Murres	Kittiwakes	Auklets	Puffins	Fulmars	Storm Petrels	Cormorants	Gulls	Albatross Jaeger
Transient KIllers	0	0	0	0	0	0	0	0	0	0	0	0	0	0	0	0	0	0	0	0	0	0	0	0	0	0	0	0	0	0
Sperm and Beaked Whales	0	0	0	0	0	0	0	0	0	0	0	0	0	0	0	0	0	0	0	0	0	0	0	0	0	0	0	0	0	0
Resident Killers	0	0	0	0	0	0	0	0	0	0	0	0	0	0	0	0	0	0	0	0	0	0	0	0	0	0	0	0	0	0
Porpoises	0.006	0	0	0	0	0	0	0	0	0	0	0	0	0	0	0	0	0	0	0	0	0	0	0	0	0	0	0	0	0
Belugas	0.02	0	0	0	0	0	0	0	0	0	0	0	0	0	0	0	0	0	0	0	0	0	0	0	0	0	0	0	0	0
Gray Whales	0.054	0	0	0	0	0	0	0	0	0	0	0	0	0	0	0	0	0	0	0	0	0	0	0	0	0	0	0	0	0
Humpbacks	0.02	0	0	0	0	0	0	0	0	0	0	0	0	0	0	0	0	0	0	0	0	0	0	0	0	0	0	0	0	0
Fin Whales	0.749	0	0	0	0	0	0	0	0	0	0	0	0	0	0	0	0	0	0	0	0	0	0	0	0	0	0	0	0	0
Sei Whales	0.01	0	0	0	0	0	0	0	0	0	0	0	0	0	0	0	0	0	0	0	0	0	0	0	0	0	0	0	0	0
Right Whales	0.006	0	0	0	0	0	0	0	0	0	0	0	0	0	0	0	0	0	0	0	0	0	0	0	0	0	0	0	0	0
Minke Whales	0.041	0	0	0	0	0	0	0	0	0	0	0	0	0	0	0	0	0	0	0	0	0	0	0	0	0	0	0	0	0
Bowhead Whales	0.012	0	0	0	0	0	0	0	0	0	0	0	0	0	0	0	0	0	0	0	0	0	0	0	0	0	0	0	0	0
Sea Otters	0.001	0	0	0	0	0	0	0	0	0	0	0	0	0	0	0	0	0	0	0	0	0	0	0	0	0	0	0	0	0
Walrus Bd Seals	0	0	0	0	0	0	0	0	0	0	0	0	0	0	0	0	0	0	0	0	0	0	0	0	0	0	0	0	0	0
N Fur Seal Juv	0.003	0	0	0	0	0	0	0	0	0	0	0	0	0	0	0	0	0	0	0	0	0	0	0	0	0	0	0	0	0
N Fur Seal Ad	0.054	0	0	0	0	0	0	0	0	0	0	0	0	0	0	0	0	0	0	0	0	0	0	0	0	0	0	0	0	0
Steller Sea Lions Juv	0	0	0	0	0	0	0	0	0	0	0	0	0	0	0	0	0	0	0	0	0	0	0	0	0	0	0	0	0	0
Steller Sea Lions Ad	0	0	0	0	0	0	0	0	0	0	0	0	0	0	0	0	0	0	0	0	0	0	0	0	0	0	0	0	0	0
Resident Seals	0.023	0	0	0	0	0	0	0	0	0	0	0	0	0	0	0	0	0	0	0	0	0	0	0	0	0	0	0	0	0
Wintering Seals	0	0	0	0	0	0	0	0	0	0	0	0	0	0.001	0	0	0	0	0	0	0	0	0	0	0	0	0	0	0	0
Shearwater	0	0	0	0	0	0	0	0	0	0	0	0	0	0	0	0	0	0	0	0	0	0	0	0	0	0	0	0	0	0
Murres	0	0	0	0	0	0	0	0	0	0	0	0	0	0	0	0	0	0	0	0	0	0	0	0	0	0.001	0	0	0	0
Kittiwakes	0	0	0	0	0	0	0	0	0	0	0	0	0	0	0	0	0	0	0	0	0	0	0	0	0	0	0	0	0	0
Auklets	0	0	0	0	0	0	0	0	0	0	0	0	0	0	0	0	0	0	0	0	0	0	0	0	0	0	0	0	0	0
Puffins	0	0	0	0	0	0	0	0	0	0	0	0	0	0	0	0	0	0	0	0	0	0	0	0	0	0	0	0	0	0
Fulmars	0	0	0	0	0	0	0	0	0	0	0	0	0	0	0	0	0	0	0	0	0	0	0	0	0	0	0	0	0	0
Storm Petrels	0	0	0	0	0	0	0	0	0	0	0	0	0	0	0	0	0	0	0	0	0	0	0	0	0	0	0	0	0	0
Cormorants	0	0	0	0	0	0	0	0	0	0	0	0	0	0	0	0	0	0	0	0	0	0	0	0	0	0	0	0	0	0
Gulls	0	0	0	0	0	0	0	0	0	0	0	0	0	0	0	0	0	0	0	0	0	0	0	0	0	0	0	0	0	0
Albatross Jaeger	0	0	0	0	0	0	0	0	0	0	0	0	0	0	0	0	0	0	0	0	0	0	0	0	0	0	0	0	0	0

Group	Transient Killers	Sperm and Beaked Whales	Resident Killers	Porpoises	Belugas	Gray Whales	Humpbacks	Fin Whales	Sei Whales	Right Whales	Minke Whales	Bowhead Whales	Sea Otters	Walrus Bd Seals	N Fur Seal Juv	N Fur Seal Ad	Steller Sea Lions Juv	Steller Sea Lions Ad	Resident Seals	Wintering Seals	Shearwater	Murres	Kittiwakes	Auklets	Puffins	Fulmars	Storm Petrels	Cormorants	Gulls	Albatross Jaeger
Sleeper shark	0	0.001	0	0	0	0	0	0	0	0	0	0	0	0	0	0	0	0	0	0	0	0	0	0	0	0	0	0	0	0
W. Pollock Juv	0	0	0.054	0.114	0.227	0	0.14	0.071	0.099	0	0.216	0	0.018	0	0.305	0.305	0.035	0	0.017	0.052	0	0.27	0.223	0.003	0.111	0.293	0.016	0.024	0.002	0.173
W. Pollock Ad	0	0	0.447	0	0	0	0	0	0	0	0	0	0.149	0	0	0	0.295	0.33	0.145	0.436	0	0	0	0	0	0	0	0	0	0
P. Cod Juv	0	0	0.002	0.005	0.01	0	0	0	0	0	0	0	0.001	0	0.007	0.007	0.07	0.07	0.045	0.005	0	0.2	0.01	0	0.005	0.013	0.001	0.001	0	0.008
P. Cod Ad	0	0.058	0.06	0	0	0	0	0	0	0	0	0	0	0	0	0	0	0	0	0	0	0	0	0	0	0	0	0	0	0
Herring Juv	0	0	0	0.001	0.001	0	0.001	0	0.001	0	0.001	0	0	0	0.061	0.061	0.03	0.03	0.028	0.044	0	0	0.003	0	0	0	0	0.127	0	0.001
Herring Ad	0	0	0.015	0	0	0	0	0	0	0	0	0	0.005	0	0	0	0	0	0	0	0	0	0	0	0	0	0	0	0	0
Arrowtooth Juv	0	0	0.002	0	0	0	0	0	0	0	0	0	0	0	0	0	0	0	0	0	0	0	0	0	0	0	0	0	0	0
Arrowtooth Ad	0	0	0.023	0	0	0	0	0	0	0	0	0	0	0	0	0	0	0	0	0	0	0	0	0	0	0	0	0	0	0
Kamchatka fl. Juv	0	0	0	0	0	0	0	0	0	0	0	0	0	0	0	0	0	0	0	0	0	0	0	0	0	0	0	0	0	0
Kamchatka fl. Ad	0	0	0.001	0	0	0	0	0	0	0	0	0	0	0	0	0	0	0	0	0	0	0	0	0	0	0	0	0	0	0
Gr. Turbot Juv	0	0	0.001	0	0	0	0	0	0	0	0	0	0	0	0	0	0	0	0	0	0	0	0	0	0	0	0	0	0	0
Gr. Turbot Ad	0	0	0.008	0	0	0	0	0	0	0	0	0	0	0	0	0	0	0	0	0	0	0	0	0	0	0	0	0	0	0
P. Halibut Juv	0	0	0.001	0	0	0	0	0	0	0	0	0	0	0	0	0	0	0	0	0	0	0	0	0	0	0	0	0	0	0
P. Halibut Ad	0	0	0.005	0	0	0	0	0	0	0	0	0	0	0	0	0	0	0	0	0	0	0	0	0	0	0	0	0	0	0
YF. Sole Juv	0	0	0.012	0	0	0	0	0	0	0	0	0	0	0	0.018	0.018	0.027	0.027	0.02	0.009	0	0	0	0	0	0	0	0	0	0
YF. Sole Ad	0	0	0.117	0	0	0	0	0	0	0	0	0	0	0	0	0	0	0	0	0	0	0	0	0	0	0	0	0	0	0
FH. Sole Juv	0	0	0.003	0	0	0	0	0	0	0	0	0	0	0	0.004	0.004	0.007	0.007	0.005	0.002	0	0	0	0	0	0	0	0	0	0
FH. Sole Ad	0	0	0.029	0	0	0	0	0	0	0	0	0	0	0	0	0	0	0	0	0	0	0	0	0	0	0	0	0	0	0
N. Rock sole Juv	0	0	0.008	0	0	0	0	0	0	0	0	0	0	0	0.012	0.012	0.018	0.018	0.013	0.006	0	0	0	0	0	0	0	0	0	0
N. Rock sole Ad	0	0	0.078	0	0	0	0	0	0	0	0	0	0	0	0	0	0	0	0	0	0	0	0	0	0	0	0	0	0	0
AK Plaice	0	0	0.026	0	0	0	0	0	0	0	0	0	0	0	0.04	0.04	0.059	0.059	0.044	0.019	0	0	0	0	0	0	0	0	0	0
Dover Sole	0	0	0	0	0	0	0	0	0	0	0	0	0	0	0	0	0	0	0	0	0	0	0	0	0	0	0	0	0	0
Rex Sole	0	0	0.001	0	0	0	0	0	0	0	0	0	0	0	0.002	0.002	0.002	0.002	0.002	0.001	0	0	0	0	0	0	0	0	0	0
Misc. Flatfish	0	0	0.004	0	0	0	0	0	0	0	0	0	0	0	0.006	0.006	0.008	0.008	0.006	0.003	0	0	0	0	0	0	0	0	0	0
Alaska skate	0	0.016	0	0	0	0	0	0	0	0	0	0	0	0	0	0	0	0	0	0	0	0	0	0	0	0	0	0	0	0
Other skates	0	0.002	0	0	0	0	0	0	0	0	0	0	0	0	0	0	0	0	0	0	0	0	0	0	0	0	0	0	0	0
Sablefish Juv	0	0	0	0	0	0	0	0	0	0	0	0	0	0	0.005	0	0	0	0	0	0	0	0	0	0	0	0	0	0	0
Sablefish Ad	0	0.001	0	0	0	0	0	0	0	0	0	0	0	0	0	0	0	0	0	0	0	0	0	0	0	0	0	0	0	0
Eelpouts	0	0.044	0	0.096	0.192	0	0	0	0	0	0	0	0	0	0	0	0.01	0.01	0.057	0.06	0	0	0	0	0	0	0	0	0	0
Grenadiers	0	0.023	0	0	0	0	0	0	0	0	0	0	0	0	0	0	0	0	0	0	0	0	0	0	0	0	0	0	0	0
Misc. fish deep	0	0	0	0	0	0	0	0	0	0	0	0	0	0	0	0	0	0	0	0	0	0	0	0	0	0	0	0	0	0
POP	0	0.004	0	0	0	0	0	0	0	0	0	0	0	0	0.001	0.001	0.003	0.003	0.003	0	0	0.001	0.001	0.001	0.001	0.001	0.001	0.001	0.001	0
Sharpchin Rock	0	0	0	0	0	0	0	0	0	0	0	0	0	0	0	0	0	0	0	0	0	0	0	0	0	0	0	0	0	0
Northern Rock	0	0.001	0	0	0	0	0	0	0	0	0	0	0	0	0	0	0	0	0	0	0	0	0	0	0	0	0	0	0	0

Group	Transient Killers	Sperm and Beaked Whales	Resident Killers	Porpoises	Belugas	Gray Whales	Humpbacks	Fin Whales	Sei Whales	Right Whales	Minke Whales	Bowhead Whales	Sea Otters	Walrus Bd Seals	N Fur Seal Juv	N Fur Seal Ad	Steller Sea Lions Juv	Steller Sea Lions Ad	Resident Seals	Wintering Seals	Shearwater	Murres	Kittiwakes	Auklets	Puffins	Fulmars	Storm Petrels	Cormorants	Gulls	Albatross Jaeger
Dusky Rock	0	0	0	0	0	0	0	0	0	0	0	0	0	0	0	0	0	0	0	0	0	0	0	0	0	0	0	0	0	0
Shortraker Rock	0	0	0	0	0	0	0	0	0	0	0	0	0	0	0	0	0	0	0	0	0	0	0	0	0	0	0	0	0	0
Rougheye Rock	0	0	0	0	0	0	0	0	0	0	0	0	0	0	0	0	0	0	0	0	0	0	0	0	0	0	0	0	0	0
Shortspine Thorns	0	0	0	0	0	0	0	0	0	0	0	0	0	0	0	0	0	0	0	0	0	0	0	0	0	0	0	0	0	0
Other Sebastes	0	0	0	0	0	0	0	0	0	0	0	0	0	0	0	0	0	0	0	0	0	0	0	0	0	0	0	0	0	0
Atka mackerel Juv	0	0	0	0	0	0	0	0	0	0	0	0	0	0	0	0	0	0	0	0	0	0	0	0	0	0	0	0	0	0
Atka mackerel Ad	0	0	0	0	0	0	0	0	0	0	0	0	0	0	0.005	0.005	0.001	0.001	0.011	0	0	0	0	0	0	0	0	0	0	0
Greenlings	0	0	0	0	0	0	0	0	0	0	0	0	0	0	0	0	0	0	0	0	0	0	0	0	0	0	0	0	0	0
Lg. Sculpins	0	0	0	0	0	0	0	0	0	0	0	0	0	0	0	0	0	0	0	0	0	0	0	0	0	0	0	0	0	0
Other sculpins	0	0	0	0.04	0.08	0	0	0	0	0	0	0	0	0	0.001	0.001	0.06	0.06	0.057	0.079	0	0	0	0	0	0	0	0	0	0
Misc. fish shallow	0	0	0	0.026	0.053	0	0.033	0.016	0	0	0	0	0	0	0	0	0.03	0.03	0.153	0.099	0	0	0	0	0	0	0	0	0	0
Octopi	0	0	0	0	0.009	0	0	0	0	0	0	0	0	0.01	0.001	0.001	0.181	0.181	0.113	0.01	0	0	0	0	0	0	0	0	0	0
Squids	0	0.85	0	0.5	0.067	0	0.005	0	0.05	0	0.02	0	0.05	0	0.305	0.305	0.03	0.03	0.028	0	0.26	0.034	0.007	0.001	0.038	0.585	0.606	0	0	0.5
Salmon returning	0	0	0.004	0.008	0.017	0	0.01	0.005	0	0	0.016	0	0	0	0.01	0.01	0.005	0.005	0.003	0	0	0	0	0	0	0	0	0	0	0
Salmon outgoing	0	0	0	0	0	0	0	0	0	0	0	0	0	0	0.01	0.01	0.005	0.005	0.003	0	0	0	0	0	0	0	0	0	0	0
Bathylagidae	0	0	0.004	0.008	0	0	0	0	0	0	0	0	0	0	0	0	0	0	0	0	0.014	0	0	0	0	0	0	0.025	0	0.012
Myctophidae	0	0	0.014	0.029	0	0	0	0	0	0	0	0	0	0	0.041	0.045	0	0	0	0	0.048	0.002	0.12	0	0	0	0	0.089	0	0.044
Capelin	0	0	0.011	0.024	0.048	0	0.03	0.015	0	0	0.046	0	0.004	0	0.156	0.156	0.06	0.06	0.062	0.084	0.406	0.052	0.071	0.008	0.099	0.01	0.006	0.074	0.134	0.037
Sandlance	0	0	0.04	0.085	0.17	0	0.105	0.053	0	0	0.161	0	0.013	0	0.01	0.01	0.04	0.04	0	0	0.141	0.184	0.25	0.027	0.351	0.035	0.021	0.423	0.473	0.129
Eulachon	0	0	0.008	0.016	0.032	0	0.02	0.01	0	0	0.03	0	0.003	0	0	0	0	0	0	0	0.027	0.035	0.047	0.005	0.066	0.007	0.004	0.049	0.089	0.024
Oth. managed forage	0	0	0.016	0.033	0.067	0	0.041	0.021	0	0	0.063	0	0.005	0	0	0	0	0	0	0	0.055	0.072	0.098	0.01	0.138	0.014	0.008	0.102	0.186	0.051
Oth. pelagic smelt	0	0	0.007	0.014	0.028	0	0.017	0.009	0	0	0.027	0	0.002	0	0	0	0	0	0	0	0.023	0.03	0.041	0.004	0.058	0.006	0.003	0.043	0.078	0.021
Bairdi	0	0	0	0	0	0.001	0	0	0	0	0	0	0	0.001	0	0	0.001	0.001	0.001	0	0	0	0	0	0	0	0	0	0	0
King Crab	0	0	0	0	0	0	0	0	0	0	0	0	0	0	0	0	0	0	0.001	0	0	0	0	0	0	0	0	0	0	0
Opilio	0	0	0	0	0	0.002	0	0	0	0	0	0.001	0	0.004	0	0	0.004	0.004	0.004	0	0	0	0	0	0	0	0	0.001	0	0
Pandalidae	0	0	0	0	0	0.005	0	0	0	0	0	0.002	0	0.008	0	0	0.004	0.004	0.064	0.02	0	0	0	0	0	0	0	0.003	0	0
NP shrimp	0	0	0	0	0	0.008	0	0	0	0	0	0.004	0	0.013	0	0	0.006	0.006	0.106	0.034	0	0	0	0	0	0	0	0.005	0	0
Sea stars	0	0	0	0	0	0.002	0	0	0	0	0	0.001	0	0	0	0	0	0	0	0	0	0	0	0	0	0	0	0	0	0
Brittle stars	0	0	0	0	0	0.002	0	0	0	0	0	0.001	0	0	0	0	0	0	0	0	0	0	0	0	0	0	0	0	0	0
Urchins dollars cucumbers	0	0	0	0	0	0.001	0	0	0	0	0	0	0.462	0	0	0	0	0	0	0	0	0	0	0	0	0	0	0	0	0
Snails	0	0	0	0	0	0.001	0	0	0	0	0	0	0	0.061	0	0	0.001	0.001	0	0	0	0	0	0	0	0	0	0	0	0
Hermit crabs	0	0	0	0	0	0.001	0	0	0	0	0	0.001	0	0.004	0	0	0.003	0.003	0.004	0.007	0	0	0	0	0	0	0	0	0	0
Misc. crabs	0	0	0	0	0	0.001	0	0	0	0	0	0	0.288	0.001	0	0	0.001	0.001	0.002	0.003	0	0	0	0	0	0	0	0	0	0

Group	Transient Killers	Sperm and Beaked Whales	Resident Killers	Porpoises	Belugas	Gray Whales	Humpbacks	Fin Whales	Sei Whales	Right Whales	Minke Whales	Bowhead Whales	Sea Otters	Walrus Bd Seals	N Fur Seal Juv	N Fur Seal Ad	Steller Sea Lions Juv	Steller Sea Lions Ad	Resident Seals	Wintering Seals	Shearwater	Murres	Kittiwakes	Auklets	Puffins	Fulmars	Storm Petrels	Cormorants	Gulls	Albatross Jaeger
Misc. Crustacean	0	0	0	0	0	0.007	0	0	0	0	0	0.003	0	0	0	0	0	0	0	0	0.01	0.006	0.004	0.001	0.063	0	0.001	0.004	0.001	0
Benthic Amphipods	0	0	0	0	0	0.9	0	0	0	0	0	0.004	0	0	0	0	0	0	0	0	0	0	0.005	0.001	0	0	0.002	0.006	0.002	0
Anemones	0	0	0	0	0	0	0	0	0	0	0	0	0	0	0	0	0	0	0	0	0	0	0	0	0	0	0	0	0	0
Corals	0	0	0	0	0	0	0	0	0	0	0	0	0	0	0	0	0	0	0	0	0	0	0	0	0	0	0	0	0	0
Hydroids	0	0	0	0	0	0	0	0	0	0	0	0	0	0	0	0	0	0	0	0	0	0	0	0	0	0	0	0	0	0
Urochordata	0	0	0	0	0	0	0	0	0	0	0	0	0	0	0	0	0	0	0	0	0	0	0	0	0	0	0	0	0	0
Sea Pens	0	0	0	0	0	0	0	0	0	0	0	0	0	0	0	0	0	0	0	0	0	0	0	0	0	0	0	0	0	0
Sponges	0	0	0	0	0	0	0	0	0	0	0	0	0	0	0	0	0	0	0	0	0	0	0	0	0	0	0	0	0	0
Bivalves	0	0	0	0	0	0.049	0	0	0	0	0	0.022	0	0.714	0	0	0.001	0.001	0	0	0	0	0.027	0.005	0	0.001	0.008	0	0.009	0
Polychaetes	0	0	0	0	0	0.017	0	0	0	0	0	0.008	0	0	0	0	0	0	0	0	0	0	0.009	0.002	0	0	0.003	0	0.003	0
Misc. worms	0	0	0	0	0	0.003	0	0	0	0	0	0.001	0	0.183	0	0	0	0	0	0.026	0	0	0.001	0	0	0	0	0	0	0
Scyphozoid Jellies	0	0	0	0	0	0	0	0	0	0	0	0	0	0	0	0	0	0	0	0	0	0	0	0	0	0	0	0	0	0
Fish Larvae	0	0	0	0	0	0	0	0	0	0	0	0	0	0	0	0	0	0	0	0	0	0	0	0	0	0	0	0	0	0
Chaetognaths	0	0	0	0	0	0	0.016	0.016	0.001	0.001	0.01	0.014	0	0	0	0	0	0	0	0	0	0	0	0	0	0	0	0	0	0
Euphausiids	0	0	0	0	0	0	0.495	0.5	0.042	0.042	0.333	0.458	0	0	0	0	0	0	0	0	0.007	0.043	0.031	0.355	0.026	0.013	0.122	0.009	0.003	0
Mysids	0	0	0	0	0	0	0.03	0.03	0.003	0.003	0.02	0.028	0	0	0	0	0	0	0	0	0	0.003	0.002	0.022	0.002	0.001	0.007	0	0	0
Pelagic Amphipods	0	0	0	0	0	0	0.047	0.047	0.004	0.004	0.032	0.043	0	0	0	0	0	0	0	0	0	0.004	0.003	0.034	0.003	0.001	0.012	0	0	0
Gelatinous filter feeders	0	0	0	0	0	0	0	0	0	0	0	0	0	0	0	0	0	0	0	0	0	0	0	0	0	0	0	0	0	0
Pteropods	0	0	0	0	0	0	0.007	0.007	0.001	0.001	0.005	0.006	0	0	0	0	0	0	0	0	0	0	0	0	0	0	0	0	0	0
Copepods	0	0	0	0	0	0	0.005	0.2	0.8	0.95	0.02	0.4	0	0	0	0	0	0	0	0	0.01	0.063	0.046	0.522	0.039	0.019	0.179	0.013	0.005	0
Pelagic microbes	0	0	0	0	0	0	0	0	0	0	0	0	0	0	0	0	0	0	0	0	0	0	0	0	0	0	0	0	0	0
Benthic microbes	0	0	0	0	0	0	0	0	0	0	0	0	0	0	0	0	0	0	0	0	0	0	0	0	0	0	0	0	0	0
Macroalgae	0	0	0	0	0	0	0	0	0	0	0	0	0	0	0	0	0	0	0	0	0	0	0	0	0	0	0	0	0	0
Lg Phytoplankton	0	0	0	0	0	0	0	0	0	0	0	0	0	0	0	0	0	0	0	0	0	0	0	0	0	0	0	0	0	0
Sm Phytoplankton	0	0	0	0	0	0	0	0	0	0	0	0	0	0	0	0	0	0	0	0	0	0	0	0	0	0	0	0	0	0
Outside Production	0	0	0	0	0	0	0	0	0	0	0	0	0	0	0	0	0	0	0	0	0	0	0	0	0	0	0	0	0	0
Discards	0	0	0	0	0	0	0	0	0	0	0	0	0	0	0	0	0	0	0	0	0	0	0	0	0	0	0	0	0.011	0
Offal	0	0	0	0	0	0	0	0	0	0	0	0	0	0	0	0	0	0	0	0	0	0	0	0	0	0	0	0	0	0
Pelagic Detritus	0	0	0	0	0	0	0	0	0	0	0	0	0	0	0	0	0	0	0	0	0	0	0	0	0	0	0	0	0	0
Benthic Detritus	0	0	0	0	0	0	0	0	0	0	0	0	0	0	0	0	0	0	0	0	0	0	0	0	0	0	0	0	0	0
Outside Detritus	0	0	0	0	0	0	0	0	0	0	0	0	0	0	0	0	0	0	0	0	0	0	0	0	0	0	0	0	0	0

Table S16: Diet matrix for the eastern Bering Sea - continued

Group	Sleeper shark	W. Pollock Juv	W. Pollock Ad	P. Cod Juv	P. Cod Ad	Herring Juv	Herring Ad	Arrowtooth Juv	Arrowtooth Ad	Kamchatka fl. Juv	Kamchatka fl. Ad	Gr. Turbot Juv	Gr. Turbot Ad	P. Halibut Juv	P. Halibut Ad	YF. Sole Juv	YF. Sole Ad	FH. Sole Juv	FH. Sole Ad	N. Rock sole Juv	N. Rock sole Ad	AK Plaice	Dover Sole	Rex Sole	Misc. Flatfish	Alaska skate	Other skates	Sablefish Juv	Sablefish Ad	Eelpouts
Transient KIllers	0	0	0	0	0	0	0	0	0	0	0	0	0	0	0	0	0	0	0	0	0	0	0	0	0	0	0	0	0	0
Sperm and Beaked Whales	0	0	0	0	0	0	0	0	0	0	0	0	0	0	0	0	0	0	0	0	0	0	0	0	0	0	0	0	0	0
Resident Killers	0	0	0	0	0	0	0	0	0	0	0	0	0	0	0	0	0	0	0	0	0	0	0	0	0	0	0	0	0	0
Porpoises	0	0	0	0	0	0	0	0	0	0	0	0	0	0	0	0	0	0	0	0	0	0	0	0	0	0	0	0	0	0
Belugas	0	0	0	0	0	0	0	0	0	0	0	0	0	0	0	0	0	0	0	0	0	0	0	0	0	0	0	0	0	0
Gray Whales	0	0	0	0	0	0	0	0	0	0	0	0	0	0	0	0	0	0	0	0	0	0	0	0	0	0	0	0	0	0
Humpbacks	0	0	0	0	0	0	0	0	0	0	0	0	0	0	0	0	0	0	0	0	0	0	0	0	0	0	0	0	0	0
Fin Whales	0	0	0	0	0	0	0	0	0	0	0	0	0	0	0	0	0	0	0	0	0	0	0	0	0	0	0	0	0	0
Sei Whales	0	0	0	0	0	0	0	0	0	0	0	0	0	0	0	0	0	0	0	0	0	0	0	0	0	0	0	0	0	0
Right Whales	0	0	0	0	0	0	0	0	0	0	0	0	0	0	0	0	0	0	0	0	0	0	0	0	0	0	0	0	0	0
Minke Whales	0	0	0	0	0	0	0	0	0	0	0	0	0	0	0	0	0	0	0	0	0	0	0	0	0	0	0	0	0	0
Bowhead Whales	0	0	0	0	0	0	0	0	0	0	0	0	0	0	0	0	0	0	0	0	0	0	0	0	0	0	0	0	0	0
Sea Otters	0	0	0	0	0	0	0	0	0	0	0	0	0	0	0	0	0	0	0	0	0	0	0	0	0	0	0	0	0	0
Walrus Bd Seals	0	0	0	0	0	0	0	0	0	0	0	0	0	0	0	0	0	0	0	0	0	0	0	0	0	0	0	0	0	0
N Fur Seal Juv	0	0	0	0	0	0	0	0	0	0	0	0	0	0	0	0	0	0	0	0	0	0	0	0	0	0	0	0	0	0
N Fur Seal Ad	0	0	0	0	0	0	0	0	0	0	0	0	0	0	0	0	0	0	0	0	0	0	0	0	0	0	0	0	0	0
Steller Sea Lions Juv	0	0	0	0	0	0	0	0	0	0	0	0	0	0	0	0	0	0	0	0	0	0	0	0	0	0	0	0	0	0
Steller Sea Lions Ad	0	0	0	0	0	0	0	0	0	0	0	0	0	0	0	0	0	0	0	0	0	0	0	0	0	0	0	0	0	0
Resident Seals	0	0	0	0	0	0	0	0	0	0	0	0	0	0	0	0	0	0	0	0	0	0	0	0	0	0	0	0	0	0
Wintering Seals	0	0	0	0	0	0	0	0	0	0	0	0	0	0	0	0	0	0	0	0	0	0	0	0	0	0	0	0	0	0
Shearwater	0	0	0	0	0	0	0	0	0	0	0	0	0	0	0	0	0	0	0	0	0	0	0	0	0	0	0	0	0	0
Murres	0	0	0	0	0	0	0	0	0	0	0	0	0	0	0	0	0	0	0	0	0	0	0	0	0	0	0	0	0	0
Kittiwakes	0	0	0	0	0	0	0	0	0	0	0	0	0	0	0	0	0	0	0	0	0	0	0	0	0	0	0	0	0	0
Auklets	0	0	0	0	0	0	0	0	0	0	0	0	0	0	0	0	0	0	0	0	0	0	0	0	0	0	0	0	0	0
Puffins	0	0	0	0	0	0	0	0	0	0	0	0	0	0	0	0	0	0	0	0	0	0	0	0	0	0	0	0	0	0
Fulmars	0	0	0	0	0	0	0	0	0	0	0	0	0	0	0	0	0	0	0	0	0	0	0	0	0	0	0	0	0	0
Storm Petrels	0	0	0	0	0	0	0	0	0	0	0	0	0	0	0	0	0	0	0	0	0	0	0	0	0	0	0	0	0	0
Cormorants	0	0	0	0	0	0	0	0	0	0	0	0	0	0	0	0	0	0	0	0	0	0	0	0	0	0	0	0	0	0
Gulls	0	0	0	0	0	0	0	0	0	0	0	0	0	0	0	0	0	0	0	0	0	0	0	0	0	0	0	0	0	0
Albatross Jaeger	0	0	0	0	0	0	0	0	0	0	0	0	0	0	0	0	0	0	0	0	0	0	0	0	0	0	0	0	0	0

Group	Sleeper shark	W. Pollock Juv	W. Pollock Ad	P. Cod Juv	P. Cod Ad	Herring Juv	Herring Ad	Arrowtooth Juv	Arrowtooth Ad	Kamchatka fl. Juv	Kamchatka fl. Ad	Gr. Turbot Juv	Gr. Turbot Ad	P. Halibut Juv	P. Halibut Ad	YF. Sole Juv	YF. Sole Ad	FH. Sole Juv	FH. Sole Ad	N. Rock sole Juv	N. Rock sole Ad	AK Plaice	Dover Sole	Rex Sole	Misc. Flatfish	Alaska skate	Other skates	Sablefish Juv	Sablefish Ad	Eelpouts
Sleeper shark	0	0	0	0	0	0	0	0	0	0	0	0	0	0	0	0	0	0	0	0	0	0	0	0	0	0	0	0	0	0
W. Pollock Juv	0	0.01	0.044	0.007	0.029	0	0	0.219	0.469	0.423	0.581	0.012	0.271	0	0.019	0	0.01	0	0.168	0	0.001	0	0	0	0.032	0.002	0	0	0	0
W. Pollock Ad	0.2	0	0.085	0	0.259	0	0	0	0.195	0	0.213	0	0.105	0	0.521	0	0	0	0	0	0	0	0	0	0	0.405	0.449	0	0.459	0.004
P. Cod Juv	0	0	0	0	0	0	0	0	0	0	0	0	0	0	0	0	0	0	0	0	0	0	0	0	0	0	0.004	0	0	0
P. Cod Ad	0	0	0.001	0	0.005	0	0	0	0	0	0	0	0.001	0	0.047	0	0	0	0	0	0	0	0	0	0	0.006	0	0	0	0
Herring Juv	0	0	0	0	0.002	0	0	0	0	0	0	0	0	0	0	0	0	0	0	0	0.002	0	0	0	0	0	0	0	0	0
Herring Ad	0	0	0.001	0	0	0	0	0	0.013	0	0	0	0.012	0	0.003	0	0	0	0	0	0	0	0	0	0	0.002	0	0	0	0.001
Arrowtooth Juv	0	0	0	0	0	0	0	0	0.003	0	0	0	0	0	0	0	0	0	0	0	0	0	0	0	0	0	0	0	0	0
Arrowtooth Ad	0.12	0	0.001	0	0.001	0	0	0	0	0	0	0	0	0	0.001	0	0	0	0	0	0	0	0	0	0	0.026	0	0	0	0
Kamchatka fl. Juv	0	0	0	0	0	0	0	0	0	0	0	0	0	0	0	0	0	0	0	0	0	0	0	0	0	0	0	0	0	0
Kamchatka fl. Ad	0.007	0	0	0	0	0	0	0	0	0	0	0	0	0	0	0	0	0	0	0	0	0	0	0	0	0	0	0	0	0
Gr. Turbot Juv	0	0	0	0	0	0	0	0	0	0	0	0	0	0	0	0	0	0	0	0	0	0	0	0	0	0	0	0	0	0
Gr. Turbot Ad	0.044	0	0	0	0	0	0	0	0	0	0	0	0	0	0	0	0	0	0	0	0	0	0	0	0	0	0	0	0	0
P. Halibut Juv	0	0	0	0	0	0	0	0.001	0	0	0	0	0	0	0	0	0	0	0	0	0	0	0	0	0	0.001	0	0	0	0
P. Halibut Ad	0.028	0	0	0	0.001	0	0	0	0	0	0	0	0	0	0	0	0	0	0	0	0	0	0	0	0	0	0	0	0	0
YF. Sole Juv	0	0	0	0	0	0	0	0	0	0	0	0	0	0	0	0	0	0	0	0	0	0	0	0	0	0	0	0	0	0
YF. Sole Ad	0	0	0	0	0.006	0	0	0	0	0	0	0	0	0	0.033	0	0	0	0	0	0	0	0	0	0	0.007	0.042	0	0	0
FH. Sole Juv	0	0	0	0	0.001	0	0	0.001	0.011	0	0	0	0	0	0.001	0	0	0	0.002	0	0	0	0	0	0	0	0	0	0	0
FH. Sole Ad	0	0	0	0	0.003	0	0	0	0	0	0.001	0	0	0	0.011	0	0	0	0	0	0	0	0	0	0	0	0	0	0	0
N. Rock sole Juv	0	0	0	0	0	0	0	0	0	0	0	0	0	0	0	0	0	0	0	0	0	0	0	0	0	0	0	0	0	0
N. Rock sole Ad	0	0	0	0	0.013	0	0	0	0	0	0	0	0	0	0.032	0	0	0	0	0	0	0	0	0	0	0.085	0.02	0	0	0
AK Plaice	0	0	0	0	0	0	0	0	0	0	0	0	0	0	0.005	0	0	0	0	0	0	0	0	0	0	0	0	0	0	0
Dover Sole	0	0	0	0	0	0	0	0	0	0	0	0	0	0	0	0	0	0	0	0	0	0	0	0	0	0	0	0	0	0
Rex Sole	0	0	0	0	0	0	0	0	0	0	0	0	0	0	0.001	0	0	0	0	0	0	0	0	0	0	0	0	0	0	0
Misc. Flatfish	0	0	0	0	0.001	0	0	0	0	0	0	0	0	0	0.027	0	0	0	0	0	0	0	0	0	0	0.002	0	0	0	0
Alaska skate	0	0	0	0	0	0	0	0	0	0	0	0	0	0	0	0	0	0	0	0	0	0	0	0	0	0	0	0	0	0
Other skates	0	0	0	0	0	0	0	0	0	0	0	0	0	0	0	0	0	0	0	0	0	0	0	0	0	0	0	0	0	0
Sablefish Juv	0	0	0	0	0	0	0	0	0	0	0	0	0	0	0	0	0	0	0	0	0	0	0	0	0	0	0	0	0	0
Sablefish Ad	0	0	0	0	0	0	0	0	0	0	0	0	0	0	0.001	0	0	0	0	0	0	0	0	0	0	0	0	0	0	0
Eelpouts	0	0	0.001	0	0.04	0	0	0	0.043	0	0.053	0	0.073	0	0.009	0	0	0.003	0.002	0	0.001	0	0	0	0.042	0.087	0.032	0	0	0.018
Grenadiers	0.2	0	0	0	0	0	0	0	0	0	0	0	0	0	0	0	0	0	0	0	0	0	0	0	0	0	0	0	0	0
Misc. fish deep	0	0	0	0	0	0	0	0	0	0	0	0	0	0	0	0	0	0	0	0	0	0	0	0	0	0	0	0	0	0
POP	0	0	0	0	0	0	0	0	0	0	0	0	0	0	0	0	0	0	0	0	0	0	0	0	0	0	0	0	0	0
Sharpchin Rock	0	0	0	0	0	0	0	0	0	0	0	0	0	0	0	0	0	0	0	0	0	0	0	0	0	0	0	0	0	0
Northern Rock	0	0	0	0	0	0	0	0	0	0	0	0	0	0	0	0	0	0	0	0	0	0	0	0	0	0	0	0	0	0
Dusky Rock	0	0	0	0	0	0	0	0	0	0	0	0	0	0	0	0	0	0	0	0	0	0	0	0	0	0	0	0	0	0
Shortraker Rock	0	0	0	0	0	0	0	0	0	0	0	0	0	0	0	0	0	0	0	0	0	0	0	0	0	0	0	0	0	0
Rougheye Rock	0	0	0	0	0	0	0	0	0	0	0	0	0	0	0	0	0	0	0	0	0	0	0	0	0	0	0	0	0	0

Group	Sleeper shark	W. Pollock Juv	W. Pollock Ad	P. Cod Juv	P. Cod Ad	Herring Juv	Herring Ad	Arrowtooth Juv	Arrowtooth Ad	Kamchatka fl. Juv	Kamchatka fl. Ad	Gr. Turbot Juv	Gr. Turbot Ad	P. Halibut Juv	P. Halibut Ad	YF. Sole Juv	YF. Sole Ad	FH. Sole Juv	FH. Sole Ad	N. Rock sole Juv	N. Rock sole Ad	AK Plaice	Dover Sole	Rex Sole	Misc. Flatfish	Alaska skate	Other skates	Sablefish Juv	Sablefish Ad	Eelpouts
Shortspine Thorns	0	0	0	0	0	0	0	0	0	0	0	0	0	0	0	0	0	0	0	0	0	0	0	0	0	0	0	0	0	0
Other Sebastes	0	0	0	0	0	0	0	0	0	0	0	0	0	0	0	0	0	0	0	0	0	0	0	0	0	0	0	0	0	0
Atka mackerel Juv	0	0	0	0	0	0	0	0	0	0	0	0	0	0	0	0	0	0	0	0	0	0	0	0	0	0	0	0	0	0
Atka mackerel Ad	0	0	0	0	0.002	0	0	0	0	0	0	0	0	0	0.005	0	0	0	0	0	0	0	0	0	0	0	0	0	0	0
Greenlings	0	0	0	0	0	0	0	0	0	0	0	0	0	0	0	0	0	0	0	0	0	0	0	0	0	0	0	0	0	0
Lg. Sculpins	0	0	0	0	0.002	0	0	0	0	0	0	0	0	0	0.002	0	0	0	0	0	0	0	0	0	0	0	0	0	0	0
Other sculpins	0	0	0.001	0.01	0.011	0	0	0.026	0.009	0	0.003	0	0.022	0	0.022	0	0.002	0.008	0.001	0.001	0	0	0	0	0	0.024	0.002	0	0	0.014
Misc. fish shallow	0.02	0	0	0	0.004	0	0	0.002	0	0	0.008	0	0.01	0	0.015	0	0	0	0.005	0	0	0	0	0	0	0.004	0.033	0	0	0.001
Octopi	0.02	0	0	0	0.006	0	0	0	0	0	0	0	0	0	0.005	0	0	0	0	0	0	0	0	0	0	0	0	0	0.039	0
Squids	0.2	0	0.002	0	0.001	0	0	0	0.009	0	0.002	0	0.325	0	0.003	0	0	0.003	0	0	0	0	0	0	0	0	0	0.1	0.111	0
Salmon returning	0.05	0	0	0	0.002	0	0	0	0	0	0	0	0	0	0	0	0	0	0	0	0	0	0	0	0	0.026	0	0	0	0
Salmon outgoing	0	0	0	0	0	0	0	0	0	0	0	0	0	0	0	0	0	0	0	0	0	0	0	0	0	0	0	0	0	0
Bathylagidae	0	0	0.001	0	0	0	0	0	0	0	0	0	0.051	0	0	0	0	0	0	0	0	0	0	0	0	0	0	0	0.018	0
Myctophidae	0	0.001	0.003	0	0	0	0	0	0.003	0	0.001	0	0.064	0	0	0	0	0	0	0	0	0	0	0	0	0	0	0	0.001	0
Capelin	0	0	0.001	0	0.004	0	0	0	0.012	0	0	0	0	0	0.039	0	0	0	0.001	0	0	0	0	0	0.046	0	0	0	0	0
Sandlance	0	0.001	0.002	0.006	0.024	0	0	0	0	0	0	0	0	0	0.034	0.002	0.006	0	0.003	0	0.141	0.007	0	0	0	0.029	0	0	0	0
Eulachon	0	0	0	0	0	0	0	0	0.021	0	0	0	0	0	0	0	0	0	0	0	0	0	0	0	0	0	0	0	0	0
Oth. managed forage	0	0	0.001	0.003	0.008	0	0	0.004	0.006	0.008	0.03	0	0.01	0	0.003	0	0.001	0	0.008	0	0.001	0	0	0	0.008	0.003	0	0	0.308	0.012
Oth. pelagic smelt	0	0	0	0	0	0	0	0	0	0	0	0	0	0	0	0	0	0	0	0	0	0	0	0	0	0	0	0	0	0
Bairdi	0	0	0	0	0.048	0	0	0	0	0	0.001	0	0	0	0.026	0	0.003	0	0.007	0	0	0	0.015	0	0.006	0.005	0.056	0	0	0.001
King Crab	0	0	0	0	0.01	0	0	0	0	0	0	0	0	0	0.002	0	0	0	0	0	0	0	0	0	0	0	0	0	0	0
Opilio	0	0	0	0.001	0.074	0	0	0	0	0	0	0	0	0	0.034	0	0.007	0	0.01	0	0.001	0.006	0	0	0	0.031	0.022	0	0	0.093
Pandalidae	0.003	0.001	0.036	0.01	0.053	0	0	0.054	0.039	0.028	0.023	0	0.004	0	0.01	0	0	0.061	0.108	0.002	0	0	0.222	0	0.096	0.001	0	0	0.001	0
NP shrimp	0.003	0.005	0.015	0.177	0.076	0	0	0.419	0.022	0.403	0.039	0.004	0.005	0.693	0.005	0.061	0.073	0.111	0.102	0.01	0.031	0	0.265	0.545	0.1	0.065	0.111	0	0.005	0.014
Sea stars	0	0	0	0	0	0	0	0	0	0	0	0	0	0	0	0	0	0	0	0	0	0	0	0	0	0.001	0	0	0.001	0
Brittle stars	0	0	0	0	0	0	0	0	0	0	0	0	0	0	0	0.01	0.032	0.142	0.248	0.001	0.013	0.002	0.01	0	0.06	0	0	0	0	0.182
Urchins dollars cucumbers	0	0	0	0	0	0	0	0	0	0	0	0	0	0	0	0.022	0.046	0	0	0.006	0.023	0.015	0	0	0	0	0	0	0	0
Snails	0.003	0	0	0	0.006	0	0	0	0	0	0	0	0	0	0.002	0.007	0.01	0	0.02	0	0.001	0.004	0	0	0	0	0	0	0	0
Hermit crabs	0.003	0	0.001	0.007	0.07	0	0	0.005	0	0	0	0	0.003	0	0.047	0	0.026	0	0.016	0	0.006	0	0	0	0.001	0.022	0.201	0	0	0.003
Misc. crabs	0	0	0	0	0.014	0	0	0	0	0	0	0	0	0	0.007	0.001	0.002	0	0.001	0	0.001	0	0	0	0	0.001	0.017	0	0	0.005
Misc. Crustacean	0	0.016	0.001	0.007	0.001	0	0	0	0	0	0	0	0	0	0	0.435	0.009	0.012	0.001	0.004	0.012	0.002	0.002	0	0.005	0	0	0	0	0.005
Benthic Amphipods	0	0.057	0.02	0.392	0.028	0	0	0.002	0.001	0.026	0.007	0.002	0.003	0	0	0.208	0.075	0.274	0.023	0.349	0.096	0.1	0.089	0.118	0.064	0.009	0.005	0	0	0.328
Anemones	0	0	0	0	0	0	0	0	0	0	0	0	0	0	0	0	0	0	0	0	0	0	0	0	0	0	0	0	0	0
Corals	0	0	0	0	0	0	0	0	0	0	0	0	0	0	0	0	0	0	0	0	0	0	0	0	0	0	0	0	0	0
Hydroids	0	0	0	0	0	0	0	0	0	0	0	0	0	0	0	0	0	0	0	0	0	0	0	0	0	0	0	0	0	0.002
Urochordata	0	0	0	0	0.001	0	0	0	0	0	0	0	0	0	0	0.005	0.005	0	0.001	0.001	0.001	0	0	0	0	0	0	0.15	0	0

Group	Sleeper shark	W. Pollock Juv	W. Pollock Ad	P. Cod Juv	P. Cod Ad	Herring Juv	Herring Ad	Arrowtooth Juv	Arrowtooth Ad	Kamchatka fl. Juv	Kamchatka fl. Ad	Gr. Turbot Juv	Gr. Turbot Ad	P. Halibut Juv	P. Halibut Ad	YF. Sole Juv	YF. Sole Ad	FH. Sole Juv	FH. Sole Ad	N. Rock sole Juv	N. Rock sole Ad	AK Plaice	Dover Sole	Rex Sole	Misc. Flatfish	Alaska skate	Other skates	Sablefish Juv	Sablefish Ad	Eelpouts
Sea Pens	0	0	0	0	0	0	0	0	0	0	0	0	0	0	0	0	0	0	0	0	0	0	0	0	0	0	0	0	0	0
Sponges	0	0	0	0	0	0	0	0	0	0	0	0	0	0	0	0	0	0	0	0	0	0	0	0	0	0	0	0	0	0
Bivalves	0	0.001	0	0	0.002	0	0	0	0	0	0	0	0	0	0.004	0.053	0.115	0.002	0.024	0.061	0.073	0.155	0.011	0	0.07	0	0	0	0	0.053
Polychaetes	0	0.006	0.002	0.041	0.036	0	0	0	0.001	0.029	0.002	0	0	0	0	0.142	0.253	0.012	0.039	0.536	0.472	0.584	0.282	0.205	0.244	0.003	0.003	0	0	0.239
Misc. worms	0	0	0.002	0.002	0.034	0	0	0	0	0	0.001	0	0	0	0.002	0.008	0.156	0.01	0.005	0.006	0.11	0.123	0.054	0.033	0.02	0	0	0	0	0.024
Scyphozoid Jellies	0	0	0	0	0	0	0	0	0	0	0	0	0	0	0	0	0.005	0	0	0	0	0	0	0	0	0	0	0	0.016	0
Fish Larvae	0	0	0	0	0	0	0	0	0	0	0	0	0	0	0	0	0	0	0	0	0	0	0	0	0	0	0	0	0	0
Chaetognaths	0	0.048	0.01	0	0	0	0	0	0	0	0	0	0	0	0	0	0	0.003	0.001	0	0	0	0	0	0	0	0	0	0	0
Euphausiids	0	0.329	0.35	0.033	0.024	0.879	0.957	0.246	0.092	0.078	0.03	0.974	0.04	0	0.003	0.026	0.138	0.152	0.117	0.005	0.012	0.001	0.035	0.099	0.001	0	0	0.5	0	0
Mysids	0	0.094	0.012	0.297	0.025	0	0	0.02	0.001	0.005	0.001	0	0	0.306	0.001	0.011	0.005	0.196	0.046	0.017	0.001	0	0.002	0	0.195	0	0	0	0	0
Pelagic Amphipods	0	0.005	0.016	0	0	0	0	0	0	0	0	0.008	0	0	0	0	0	0	0	0	0	0	0.012	0	0.005	0	0	0	0	0.001
Gelatinous filter feeders	0	0.006	0.014	0	0	0	0	0	0	0	0	0	0	0	0	0	0.002	0	0	0	0.001	0	0	0	0	0	0	0	0	0
Pteropods	0	0	0.001	0	0	0	0	0	0	0	0	0	0	0	0	0	0.004	0.006	0	0	0.001	0	0	0	0	0	0	0.1	0	0
Copepods	0	0.419	0.365	0.006	0	0.121	0.043	0	0	0	0	0	0	0	0	0.004	0	0.004	0	0	0	0	0	0	0.003	0	0	0.15	0	0
Pelagic microbes	0	0	0	0	0	0	0	0	0	0	0	0	0	0	0	0	0	0	0	0	0	0	0	0	0	0	0	0	0	0.001
Benthic microbes	0	0	0	0	0	0	0	0	0	0	0	0	0	0	0	0	0	0	0	0	0	0	0	0	0	0	0	0	0	0
Macroalgae	0	0	0	0	0	0	0	0	0	0	0	0	0	0	0	0	0	0	0	0	0	0	0	0	0	0	0	0	0	0
Lg Phytoplankton	0	0	0	0	0	0	0	0	0	0	0	0	0	0	0	0	0	0	0	0	0	0	0	0	0	0	0	0	0	0
Sm Phytoplankton	0	0	0	0	0	0	0	0	0	0	0	0	0	0	0	0	0	0	0	0	0	0	0	0	0	0	0	0	0	0
Outside Production	0	0	0	0	0	0	0	0	0	0	0	0	0	0	0	0	0	0	0	0	0	0	0	0	0	0	0	0	0	0
Discards	0	0	0	0	0	0	0	0	0	0	0	0	0	0	0	0	0	0	0	0	0	0	0	0	0	0	0	0	0	0
Offal	0.1	0	0.011	0	0.073	0	0	0	0.048	0	0.003	0	0	0	0.017	0.003	0.011	0	0.04	0	0	0	0	0	0	0.154	0.001	0	0.039	0.001
Pelagic Detritus	0	0	0	0	0	0	0	0	0	0	0	0	0	0	0	0	0	0	0	0	0	0	0	0	0	0	0	0	0	0
Benthic Detritus	0	0	0	0	0	0	0	0	0	0	0	0	0	0	0	0	0	0	0	0	0	0	0	0	0	0	0	0	0	0
Outside Detritus	0	0	0	0	0	0	0	0	0	0	0	0	0	0	0	0	0	0	0	0	0	0	0	0	0	0	0	0	0	0

Table S16: Diet matrix for the eastern Bering Sea – continued

**Group**	Grenadiers	Misc. fish deep	POP	Sharpchin Rock	Northern Rock	Dusky Rock	Shortraker Rock	Rougheye Rock	Shortspine Thorns	Other Sebastes	Atka mackerel Juv	Atka mackerel Ad	Greenlings	Lg. Sculpins	Other sculpins	Misc. fish shallow	Octopi	Squids	Salmon returning	Salmon outgoing	Bathylagidae	Myctophidae	Capelin	Sandlance	Eulachon	Oth. managed forage	Oth. pelagic smelt	Bairdi	King Crab	Opilio
Transient KIllers	0	0	0	0	0	0	0	0	0	0	0	0	0	0	0	0	0	0	0	0	0	0	0	0	0	0	0	0	0	0
Sperm and Beaked Whales	0	0	0	0	0	0	0	0	0	0	0	0	0	0	0	0	0	0	0	0	0	0	0	0	0	0	0	0	0	0
Resident Killers	0	0	0	0	0	0	0	0	0	0	0	0	0	0	0	0	0	0	0	0	0	0	0	0	0	0	0	0	0	0
Porpoises	0	0	0	0	0	0	0	0	0	0	0	0	0	0	0	0	0	0	0	0	0	0	0	0	0	0	0	0	0	0
Belugas	0	0	0	0	0	0	0	0	0	0	0	0	0	0	0	0	0	0	0	0	0	0	0	0	0	0	0	0	0	0
Gray Whales	0	0	0	0	0	0	0	0	0	0	0	0	0	0	0	0	0	0	0	0	0	0	0	0	0	0	0	0	0	0
Humpbacks	0	0	0	0	0	0	0	0	0	0	0	0	0	0	0	0	0	0	0	0	0	0	0	0	0	0	0	0	0	0
Fin Whales	0	0	0	0	0	0	0	0	0	0	0	0	0	0	0	0	0	0	0	0	0	0	0	0	0	0	0	0	0	0
Sei Whales	0	0	0	0	0	0	0	0	0	0	0	0	0	0	0	0	0	0	0	0	0	0	0	0	0	0	0	0	0	0
Right Whales	0	0	0	0	0	0	0	0	0	0	0	0	0	0	0	0	0	0	0	0	0	0	0	0	0	0	0	0	0	0
Minke Whales	0	0	0	0	0	0	0	0	0	0	0	0	0	0	0	0	0	0	0	0	0	0	0	0	0	0	0	0	0	0
Bowhead Whales	0	0	0	0	0	0	0	0	0	0	0	0	0	0	0	0	0	0	0	0	0	0	0	0	0	0	0	0	0	0
Sea Otters	0	0	0	0	0	0	0	0	0	0	0	0	0	0	0	0	0	0	0	0	0	0	0	0	0	0	0	0	0	0
Walrus Bd Seals	0	0	0	0	0	0	0	0	0	0	0	0	0	0	0	0	0	0	0	0	0	0	0	0	0	0	0	0	0	0
N Fur Seal Juv	0	0	0	0	0	0	0	0	0	0	0	0	0	0	0	0	0	0	0	0	0	0	0	0	0	0	0	0	0	0
N Fur Seal Ad	0	0	0	0	0	0	0	0	0	0	0	0	0	0	0	0	0	0	0	0	0	0	0	0	0	0	0	0	0	0
Steller Sea Lions Juv	0	0	0	0	0	0	0	0	0	0	0	0	0	0	0	0	0	0	0	0	0	0	0	0	0	0	0	0	0	0
Steller Sea Lions Ad	0	0	0	0	0	0	0	0	0	0	0	0	0	0	0	0	0	0	0	0	0	0	0	0	0	0	0	0	0	0
Resident Seals	0	0	0	0	0	0	0	0	0	0	0	0	0	0	0	0	0	0	0	0	0	0	0	0	0	0	0	0	0	0
Wintering Seals	0	0	0	0	0	0	0	0	0	0	0	0	0	0	0	0	0	0	0	0	0	0	0	0	0	0	0	0	0	0
Shearwater	0	0	0	0	0	0	0	0	0	0	0	0	0	0	0	0	0	0	0	0	0	0	0	0	0	0	0	0	0	0
Murres	0	0	0	0	0	0	0	0	0	0	0	0	0	0	0	0	0	0	0	0	0	0	0	0	0	0	0	0	0	0
Kittiwakes	0	0	0	0	0	0	0	0	0	0	0	0	0	0	0	0	0	0	0	0	0	0	0	0	0	0	0	0	0	0
Auklets	0	0	0	0	0	0	0	0	0	0	0	0	0	0	0	0	0	0	0	0	0	0	0	0	0	0	0	0	0	0
Puffins	0	0	0	0	0	0	0	0	0	0	0	0	0	0	0	0	0	0	0	0	0	0	0	0	0	0	0	0	0	0
Fulmars	0	0	0	0	0	0	0	0	0	0	0	0	0	0	0	0	0	0	0	0	0	0	0	0	0	0	0	0	0	0
Storm Petrels	0	0	0	0	0	0	0	0	0	0	0	0	0	0	0	0	0	0	0	0	0	0	0	0	0	0	0	0	0	0
Cormorants	0	0	0	0	0	0	0	0	0	0	0	0	0	0	0	0	0	0	0	0	0	0	0	0	0	0	0	0	0	0
Gulls	0	0	0	0	0	0	0	0	0	0	0	0	0	0	0	0	0	0	0	0	0	0	0	0	0	0	0	0	0	0
Albatross Jaeger	0	0	0	0	0	0	0	0	0	0	0	0	0	0	0	0	0	0	0	0	0	0	0	0	0	0	0	0	0	0
Sleeper shark	0	0	0	0	0	0	0	0	0	0	0	0	0	0	0	0	0	0	0	0	0	0	0	0	0	0	0	0	0	0

Group	Grenadiers	Misc. fish deep	POP	Sharpchin Rock	Northern Rock	Dusky Rock	Shortraker Rock	Rougheye Rock	Shortspine Thorns	Other Sebastes	Atka mackerel Juv	Atka mackerel Ad	Greenlings	Lg. Sculpins	Other sculpins	Misc. fish shallow	Octopi	Squids	Salmon returning	Salmon outgoing	Bathylagidae	Myctophidae	Capelin	Sandlance	Eulachon	Oth. managed forage	Oth. pelagic smelt	Bairdi	King Crab	Opilio
W. Pollock Juv	0	0	0	0	0	0	0	0	0	0	0	0	0.047	0.095	0	0	0	0	0	0	0	0	0	0	0	0	0	0	0	0
W. Pollock Ad	0	0	0	0	0	0	0	0	0	0	0	0	0	0.035	0	0	0	0	0	0	0	0	0	0	0	0	0	0	0	0
P. Cod Juv	0	0	0	0	0	0	0	0	0	0	0	0	0	0	0.04	0	0	0	0	0	0	0	0	0	0	0	0	0	0	0
P. Cod Ad	0	0	0	0	0	0	0	0	0	0	0	0	0	0.001	0	0	0	0	0	0	0	0	0	0	0	0	0	0	0	0
Herring Juv	0	0	0	0	0	0	0	0	0	0	0	0	0	0.005	0	0	0	0	0	0	0	0	0	0	0	0	0	0	0	0
Herring Ad	0	0	0	0	0	0	0	0	0	0	0	0	0	0.002	0	0	0	0	0	0	0	0	0	0	0	0	0	0	0	0
Arrowtooth Juv	0	0	0	0	0	0	0	0	0	0	0	0	0	0	0	0	0	0	0	0	0	0	0	0	0	0	0	0	0	0
Arrowtooth Ad	0	0	0	0	0	0	0	0	0	0	0	0	0	0	0	0	0	0	0	0	0	0	0	0	0	0	0	0	0	0
Kamchatka fl. Juv	0	0	0	0	0	0	0	0	0	0	0	0	0	0	0	0	0	0	0	0	0	0	0	0	0	0	0	0	0	0
Kamchatka fl. Ad	0	0	0	0	0	0	0	0	0	0	0	0	0	0	0	0	0	0	0	0	0	0	0	0	0	0	0	0	0	0
Gr. Turbot Juv	0	0	0	0	0	0	0	0	0	0	0	0	0	0	0	0	0	0	0	0	0	0	0	0	0	0	0	0	0	0
Gr. Turbot Ad	0	0	0	0	0	0	0	0	0	0	0	0	0	0	0	0	0	0	0	0	0	0	0	0	0	0	0	0	0	0
P. Halibut Juv	0	0	0	0	0	0	0	0	0	0	0	0	0	0	0	0	0	0	0	0	0	0	0	0	0	0	0	0	0	0
P. Halibut Ad	0	0	0	0	0	0	0	0	0	0	0	0	0	0	0	0	0	0	0	0	0	0	0	0	0	0	0	0	0	0
YF. Sole Juv	0	0	0	0	0	0	0	0	0	0	0	0	0	0.022	0.01	0	0	0	0	0	0	0	0	0	0	0	0	0	0	0
YF. Sole Ad	0	0	0	0	0	0	0	0	0	0	0	0	0	0.005	0	0	0	0	0	0	0	0	0	0	0	0	0	0	0	0
FH. Sole Juv	0	0	0	0	0	0	0	0	0	0	0	0	0	0	0	0.007	0	0	0	0	0	0	0	0	0	0	0	0	0	0
FH. Sole Ad	0	0	0	0	0	0	0	0	0	0	0	0	0	0.002	0	0	0	0	0	0	0	0	0	0	0	0	0	0	0	0
N. Rock sole Juv	0	0	0	0	0	0	0	0	0	0	0	0	0	0.007	0	0	0	0	0	0	0	0	0	0	0	0	0	0	0	0
N. Rock sole Ad	0	0	0	0	0	0	0	0	0	0	0	0	0	0.002	0	0	0	0	0	0	0	0	0	0	0	0	0	0	0	0
AK Plaice	0	0	0	0	0	0	0	0	0	0	0	0	0	0.001	0	0	0	0	0	0	0	0	0	0	0	0	0	0	0	0
Dover Sole	0	0	0	0	0	0	0	0	0	0	0	0	0	0	0	0	0	0	0	0	0	0	0	0	0	0	0	0	0	0
Rex Sole	0	0	0	0	0	0	0	0	0	0	0	0	0	0	0	0	0	0	0	0	0	0	0	0	0	0	0	0	0	0
Misc. Flatfish	0	0	0	0	0	0	0	0	0	0	0	0	0.03	0.001	0	0	0	0	0	0	0	0	0	0	0	0	0	0	0	0
Alaska skate	0	0	0	0	0	0	0	0	0	0	0	0	0	0	0	0	0	0	0	0	0	0	0	0	0	0	0	0	0	0
Other skates	0	0	0	0	0	0	0	0	0	0	0	0	0	0	0	0	0	0	0	0	0	0	0	0	0	0	0	0	0	0
Sablefish Juv	0	0	0	0	0	0	0	0	0	0	0	0	0	0	0	0	0	0	0	0	0	0	0	0	0	0	0	0	0	0
Sablefish Ad	0	0	0	0	0	0	0	0	0	0	0	0	0	0	0	0	0	0	0	0	0	0	0	0	0	0	0	0	0	0
Eelpouts	0	0	0	0	0	0	0	0	0	0	0	0	0	0.033	0	0	0	0	0	0	0	0	0	0	0	0	0	0	0	0
Grenadiers	0	0	0	0	0	0	0	0	0	0	0	0	0	0	0	0	0	0	0	0	0	0	0	0	0	0	0	0	0	0
Misc. fish deep	0	0	0.004	0	0	0	0	0	0	0	0	0	0	0	0	0	0	0	0	0	0	0	0	0	0	0	0	0	0	0
POP	0	0	0	0	0	0	0	0	0	0	0	0	0	0	0	0	0	0	0	0	0	0	0	0	0	0	0	0	0	0
Sharpchin Rock	0	0	0	0	0	0	0	0	0	0	0	0	0	0	0	0	0	0	0	0	0	0	0	0	0	0	0	0	0	0
Northern Rock	0	0	0	0	0	0	0	0	0	0	0	0	0	0	0	0	0	0	0	0	0	0	0	0	0	0	0	0	0	0
Dusky Rock	0	0	0	0	0	0	0	0	0	0	0	0	0	0	0	0	0	0	0	0	0	0	0	0	0	0	0	0	0	0
Shortraker Rock	0	0	0	0	0	0	0	0	0	0	0	0	0	0	0	0	0	0	0	0	0	0	0	0	0	0	0	0	0	0
Rougheye Rock	0	0	0	0	0	0	0	0	0	0	0	0	0	0	0	0	0	0	0	0	0	0	0	0	0	0	0	0	0	0
Shortspine Thorns	0	0	0	0	0	0	0	0	0	0	0	0	0	0	0	0	0	0	0	0	0	0	0	0	0	0	0	0	0	0

Group	Grenadiers	Misc. fish deep	POP	Sharpchin Rock	Northern Rock	Dusky Rock	Shortraker Rock	Rougheye Rock	Shortspine Thorns	Other Sebastes	Atka mackerel Juv	Atka mackerel Ad	Greenlings	Lg. Sculpins	Other sculpins	Misc. fish shallow	Octopi	Squids	Salmon returning	Salmon outgoing	Bathylagidae	Myctophidae	Capelin	Sandlance	Eulachon	Oth. managed forage	Oth. pelagic smelt	Bairdi	King Crab	Opilio
Other Sebastes	0	0	0	0	0	0	0	0	0	0	0	0	0	0	0	0	0	0	0	0	0	0	0	0	0	0	0	0	0	0
Atka mackerel Juv	0	0	0	0	0	0	0	0	0	0	0	0	0	0	0	0	0	0	0	0	0	0	0	0	0	0	0	0	0	0
Atka mackerel Ad	0	0	0	0	0	0	0	0	0	0	0	0	0	0	0	0	0	0	0	0	0	0	0	0	0	0	0	0	0	0
Greenlings	0	0	0	0	0	0	0	0	0	0	0	0	0	0	0	0	0	0	0	0	0	0	0	0	0	0	0	0	0	0
Lg. Sculpins	0	0	0	0	0	0	0	0	0	0	0	0	0.178	0.002	0	0	0	0	0	0	0	0	0	0	0	0	0	0	0	0
Other sculpins	0	0	0	0.001	0	0	0.006	0	0	0.001	0	0	0.15	0.021	0	0	0	0	0	0	0	0	0	0	0	0	0	0	0	0
Misc. fish shallow	0	0	0	0	0	0	0	0.001	0	0	0	0	0	0.019	0.05	0	0	0	0	0	0	0	0	0	0	0	0	0	0	0
Octopi	0	0	0	0	0	0	0	0	0	0	0	0	0	0.014	0	0	0	0	0	0	0	0	0	0	0	0	0	0	0	0
Squids	0.5	0.5	0	0.131	0.054	0	0.009	0.62	0	0.131	0	0	0	0	0	0	0	0	0.2	0	0	0	0	0	0	0	0	0	0	0
Salmon returning	0	0	0	0	0	0	0	0	0	0	0	0	0	0	0	0	0	0	0	0	0	0	0	0	0	0	0	0	0	0
Salmon outgoing	0	0	0	0	0	0	0	0	0	0	0	0	0	0	0	0	0	0	0	0	0	0	0	0	0	0	0	0	0	0
Bathylagidae	0	0	0.007	0	0	0	0	0	0	0	0	0	0	0	0	0	0	0	0	0	0	0	0	0	0	0	0	0	0	0
Myctophidae	0	0	0.01	0.001	0	0	0.003	0.002	0	0.001	0	0	0	0	0	0	0	0.025	0	0	0	0	0	0	0	0	0	0	0	0
Capelin	0	0	0	0	0	0	0	0	0	0	0	0	0	0.012	0	0	0	0.025	0	0	0	0	0	0	0	0	0	0	0	0
Sandlance	0	0	0	0	0	0	0	0	0	0	0	0	0	0.009	0	0	0	0.025	0	0	0	0	0	0	0	0	0	0	0	0
Eulachon	0	0	0	0	0	0	0	0	0	0	0	0	0	0	0	0	0	0.025	0	0	0	0	0	0	0	0	0	0	0	0
Oth. managed forage	0	0	0	0	0	0	0	0	0	0	0	0	0	0.058	0	0	0	0.025	0	0	0	0	0	0	0	0	0	0	0	0
Oth. pelagic smelt	0	0	0	0	0	0	0	0	0	0	0	0	0	0	0	0	0	0.025	0	0	0	0	0	0	0	0	0	0	0	0
Bairdi	0	0	0	0	0	0	0	0	0	0	0	0.006	0	0.005	0.035	0	0.05	0	0	0	0	0	0	0	0	0	0	0	0	0
King Crab	0	0	0	0	0	0	0	0	0	0	0	0	0	0.018	0	0	0	0	0	0	0	0	0	0	0	0	0	0	0	0
Opilio	0	0	0	0	0	0	0	0	0	0	0	0	0	0.039	0	0	0.05	0	0	0	0	0	0	0	0	0	0	0	0	0
Pandalidae	0	0	0	0.167	0	0	0.83	0.001	0.002	0.167	0	0	0	0.007	0.042	0.019	0	0	0	0	0	0	0	0	0	0	0	0	0	0
NP shrimp	0.5	0.5	0	0.064	0.018	0	0	0.138	0.153	0.064	0	0	0.43	0.19	0.501	0.207	0	0	0	0	0	0	0	0	0	0	0	0	0	0
Sea stars	0	0	0	0	0	0	0	0	0	0	0	0	0	0	0	0	0	0	0	0	0	0	0	0	0	0	0	0	0.006	0.001
Brittle stars	0	0	0	0	0	0	0	0	0	0	0	0	0	0	0	0	0	0	0	0	0	0	0	0	0	0	0	0.083	0.027	0.072
Urchins dollars cucumbers	0	0	0	0	0	0	0	0	0	0	0	0	0	0	0	0	0	0	0	0	0	0	0	0	0	0	0	0.015	0.166	0.007
Snails	0	0	0	0	0	0	0	0	0	0	0	0	0	0.001	0	0	0.4	0	0	0	0	0	0	0	0	0	0	0.038	0.198	0.048
Hermit crabs	0	0	0	0.004	0.011	0	0	0	0	0.004	0	0	0	0.019	0	0.001	0.1	0	0	0	0	0	0	0	0	0	0	0.014	0.016	0.03
Misc. crabs	0	0	0	0	0	0	0	0	0	0	0	0	0.004	0.019	0.019	0	0	0	0	0	0	0	0	0	0	0	0	0.014	0.016	0.03
Misc. Crustacean	0	0	0	0	0	0	0	0	0	0	0	0	0.001	0.001	0.014	0	0	0	0	0	0	0	0	0	0	0	0	0.005	0.015	0.017
Benthic Amphipods	0	0	0.001	0.085	0	0.046	0	0.014	0.362	0.085	0	0	0.032	0.06	0.195	0.092	0	0	0	0	0	0	0	0	0	0	0	0.038	0.009	0.048
Anemones	0	0	0	0	0	0	0	0	0	0	0	0	0	0	0	0	0	0	0	0	0	0	0	0	0	0	0	0	0	0.002
Corals	0	0	0	0	0	0	0	0	0	0	0	0	0	0	0	0	0	0	0	0	0	0	0	0	0	0	0	0	0	0
Hydroids	0	0	0	0	0	0	0	0	0	0	0	0	0.001	0	0.037	0	0	0	0	0	0	0	0	0	0	0	0	0.012	0.046	0.006

Group	Grenadiers	Misc. fish deep	POP	Sharpchin Rock	Northern Rock	Dusky Rock	Shortraker Rock	Rougheye Rock	Shortspine Thorns	Other Sebastes	Atka mackerel Juv	Atka mackerel Ad	Greenlings	Lg. Sculpins	Other sculpins	Misc. fish shallow	Octopi	Squids	Salmon returning	Salmon outgoing	Bathylagidae	Myctophidae	Capelin	Sandlance	Eulachon	Oth. managed forage	Oth. pelagic smelt	Bairdi	King Crab	Opilio
Urochordata	0	0	0	0	0	0	0	0	0	0	0	0	0	0	0	0	0	0	0	0	0	0	0	0	0	0	0	0	0.009	0
Sea Pens	0	0	0	0	0	0	0	0	0	0	0	0	0	0	0	0	0	0	0	0	0	0	0	0	0	0	0	0	0	0
Sponges	0	0	0	0	0	0	0	0	0	0	0	0	0	0	0	0	0	0	0	0	0	0	0	0	0	0	0	0.002	0	0.003
Bivalves	0	0	0	0	0	0	0	0	0	0	0	0	0.012	0	0	0	0.4	0	0	0	0	0	0	0	0	0	0	0.308	0.184	0.252
Polychaetes	0	0	0	0.017	0	0.084	0	0	0	0.017	0	0	0.071	0.11	0.026	0	0	0	0	0	0	0	0	0	0	0	0	0.384	0.189	0.285
Misc. worms	0	0	0	0	0	0	0	0	0	0	0	0	0	0.019	0	0	0	0	0	0	0	0	0	0	0	0	0	0	0.026	0.024
Scyphozoid Jellies	0	0	0	0	0	0	0	0	0	0	0	0	0	0	0	0.064	0	0	0	0	0	0	0	0	0	0	0	0	0	0
Fish Larvae	0	0	0	0	0	0	0	0	0	0	0	0	0	0	0	0	0	0	0	0	0	0	0	0	0	0	0	0	0	0
Chaetognaths	0	0	0.002	0.016	0.058	0	0	0	0	0.016	0	0	0	0	0	0	0	0.025	0.2	0	0	0	0	0	0	0	0	0	0	0
Euphausiids	0	0	0.213	0.188	0.04	0.68	0.02	0.194	0	0.188	0.9	0.984	0	0.01	0	0.012	0	0.42	0.2	0.25	0.9	0.9	0.9	0.9	0.9	0.9	0.9	0	0	0.001
Mysids	0	0	0.712	0.129	0.005	0	0.132	0.026	0.483	0.129	0	0	0.043	0.076	0.003	0.035	0	0.025	0	0	0	0	0	0	0	0	0	0	0	0.004
Pelagic Amphipods	0	0	0	0	0.001	0	0	0	0	0	0	0	0.001	0.046	0.008	0.014	0	0.14	0	0.25	0	0	0	0	0	0	0	0	0	0
Gelatinous filter feeders	0	0	0	0	0	0	0	0	0	0	0	0.004	0	0.025	0	0.55	0	0.04	0	0	0	0	0	0	0	0	0	0	0	0
Pteropods	0	0	0	0.001	0.002	0	0	0	0	0.001	0	0	0	0	0	0	0	0	0.2	0	0	0	0	0	0	0	0	0	0	0
Copepods	0	0	0.051	0.197	0.809	0.19	0	0.004	0	0.197	0.1	0.006	0	0	0.019	0	0	0.2	0.2	0.5	0.1	0.1	0.1	0.1	0.1	0.1	0.1	0	0	0
Pelagic microbes	0	0	0	0	0	0	0	0	0	0	0	0	0	0	0	0	0	0	0	0	0	0	0	0	0	0	0	0	0	0
Benthic microbes	0	0	0	0	0	0	0	0	0	0	0	0	0	0	0	0	0	0	0	0	0	0	0	0	0	0	0	0	0	0
Macroalgae	0	0	0	0	0	0	0	0	0	0	0	0	0	0	0	0	0	0	0	0	0	0	0	0	0	0	0	0	0.025	0.004
Lg Phytoplankton	0	0	0	0	0	0	0	0	0	0	0	0	0	0	0	0	0	0	0	0	0	0	0	0	0	0	0	0	0	0
Sm Phytoplankton	0	0	0	0	0	0	0	0	0	0	0	0	0	0	0	0	0	0	0	0	0	0	0	0	0	0	0	0	0	0
Outside Production	0	0	0	0	0	0	0	0	0	0	0	0	0	0	0	0	0	0	0	0	0	0	0	0	0	0	0	0	0	0
Discards	0	0	0	0	0	0	0	0	0	0	0	0	0	0	0	0	0	0	0	0	0	0	0	0	0	0	0	0.006	0.003	0.006
Offal	0	0	0	0	0	0	0	0	0	0	0	0	0	0.008	0	0	0	0	0	0	0	0	0	0	0	0	0	0.006	0.003	0.006
Pelagic Detritus	0	0	0	0	0	0	0	0	0	0	0	0	0	0	0	0	0	0	0	0	0	0	0	0	0	0	0	0	0	0
Benthic Detritus	0	0	0	0	0	0	0	0	0	0	0	0	0	0	0	0	0	0	0	0	0	0	0	0	0	0	0	0.078	0.06	0.154
Outside Detritus	0	0	0	0	0	0	0	0	0	0	0	0	0	0	0	0	0	0	0	0	0	0	0	0	0	0	0	0	0	0

Table S16: Diet matrix for the eastern Bering Sea – continued

Group	Pandalidae	NP shrimp	Sea stars	Brittle stars	Urchins dollars cucumbers	Snails	Hermit crabs	Misc. crabs	Misc. Crustacean	Benthic Amphipods	Anemones	Corals	Hydroids	Urochordata	Sea Pens	Sponges	Bivalves	Polychaetes	Misc. worms	Scyphozoid Jellies	Fish Larvae	Chaetognaths	Euphausiids	Mysids	Pelagic Amphipods	Gelatinous filter feeders	Pteropods	Copepods	Pelagic microbes	Benthic microbes
Transient KIllers	0	0	0	0	0	0	0	0	0	0	0	0	0	0	0	0	0	0	0	0	0	0	0	0	0	0	0	0	0	0
Sperm and Beaked Whales	0	0	0	0	0	0	0	0	0	0	0	0	0	0	0	0	0	0	0	0	0	0	0	0	0	0	0	0	0	0
Resident Killers	0	0	0	0	0	0	0	0	0	0	0	0	0	0	0	0	0	0	0	0	0	0	0	0	0	0	0	0	0	0
Porpoises	0	0	0	0	0	0	0	0	0	0	0	0	0	0	0	0	0	0	0	0	0	0	0	0	0	0	0	0	0	0
Belugas	0	0	0	0	0	0	0	0	0	0	0	0	0	0	0	0	0	0	0	0	0	0	0	0	0	0	0	0	0	0
Gray Whales	0	0	0	0	0	0	0	0	0	0	0	0	0	0	0	0	0	0	0	0	0	0	0	0	0	0	0	0	0	0
Humpbacks	0	0	0	0	0	0	0	0	0	0	0	0	0	0	0	0	0	0	0	0	0	0	0	0	0	0	0	0	0	0
Fin Whales	0	0	0	0	0	0	0	0	0	0	0	0	0	0	0	0	0	0	0	0	0	0	0	0	0	0	0	0	0	0
Sei Whales	0	0	0	0	0	0	0	0	0	0	0	0	0	0	0	0	0	0	0	0	0	0	0	0	0	0	0	0	0	0
Right Whales	0	0	0	0	0	0	0	0	0	0	0	0	0	0	0	0	0	0	0	0	0	0	0	0	0	0	0	0	0	0
Minke Whales	0	0	0	0	0	0	0	0	0	0	0	0	0	0	0	0	0	0	0	0	0	0	0	0	0	0	0	0	0	0
Bowhead Whales	0	0	0	0	0	0	0	0	0	0	0	0	0	0	0	0	0	0	0	0	0	0	0	0	0	0	0	0	0	0
Sea Otters	0	0	0	0	0	0	0	0	0	0	0	0	0	0	0	0	0	0	0	0	0	0	0	0	0	0	0	0	0	0
Walrus Bd Seals	0	0	0	0	0	0	0	0	0	0	0	0	0	0	0	0	0	0	0	0	0	0	0	0	0	0	0	0	0	0
N Fur Seal Juv	0	0	0	0	0	0	0	0	0	0	0	0	0	0	0	0	0	0	0	0	0	0	0	0	0	0	0	0	0	0
N Fur Seal Ad	0	0	0	0	0	0	0	0	0	0	0	0	0	0	0	0	0	0	0	0	0	0	0	0	0	0	0	0	0	0
Steller Sea Lions Juv	0	0	0	0	0	0	0	0	0	0	0	0	0	0	0	0	0	0	0	0	0	0	0	0	0	0	0	0	0	0
Steller Sea Lions Ad	0	0	0	0	0	0	0	0	0	0	0	0	0	0	0	0	0	0	0	0	0	0	0	0	0	0	0	0	0	0
Resident Seals	0	0	0	0	0	0	0	0	0	0	0	0	0	0	0	0	0	0	0	0	0	0	0	0	0	0	0	0	0	0
Wintering Seals	0	0	0	0	0	0	0	0	0	0	0	0	0	0	0	0	0	0	0	0	0	0	0	0	0	0	0	0	0	0
Shearwater	0	0	0	0	0	0	0	0	0	0	0	0	0	0	0	0	0	0	0	0	0	0	0	0	0	0	0	0	0	0
Murres	0	0	0	0	0	0	0	0	0	0	0	0	0	0	0	0	0	0	0	0	0	0	0	0	0	0	0	0	0	0
Kittiwakes	0	0	0	0	0	0	0	0	0	0	0	0	0	0	0	0	0	0	0	0	0	0	0	0	0	0	0	0	0	0
Auklets	0	0	0	0	0	0	0	0	0	0	0	0	0	0	0	0	0	0	0	0	0	0	0	0	0	0	0	0	0	0
Puffins	0	0	0	0	0	0	0	0	0	0	0	0	0	0	0	0	0	0	0	0	0	0	0	0	0	0	0	0	0	0
Fulmars	0	0	0	0	0	0	0	0	0	0	0	0	0	0	0	0	0	0	0	0	0	0	0	0	0	0	0	0	0	0
Storm Petrels	0	0	0	0	0	0	0	0	0	0	0	0	0	0	0	0	0	0	0	0	0	0	0	0	0	0	0	0	0	0
Cormorants	0	0	0	0	0	0	0	0	0	0	0	0	0	0	0	0	0	0	0	0	0	0	0	0	0	0	0	0	0	0
Gulls	0	0	0	0	0	0	0	0	0	0	0	0	0	0	0	0	0	0	0	0	0	0	0	0	0	0	0	0	0	0
Albatross Jaeger	0	0	0	0	0	0	0	0	0	0	0	0	0	0	0	0	0	0	0	0	0	0	0	0	0	0	0	0	0	0
Sleeper shark	0	0	0	0	0	0	0	0	0	0	0	0	0	0	0	0	0	0	0	0	0	0	0	0	0	0	0	0	0	0
W. Pollock Juv	0	0	0	0	0	0	0	0	0	0	0	0	0	0	0	0	0	0	0	0.001	0	0	0	0	0	0	0	0	0	0

Group	Pandalidae	NP shrimp	Sea stars	Brittle stars	Urchins dollars cucumbers	Snails	Hermit crabs	Misc. crabs	Misc. Crustacean	Benthic Amphipods	Anemones	Corals	Hydroids	Urochordata	Sea Pens	Sponges	Bivalves	Polychaetes	Misc. worms	Scyphozoid Jellies	Fish Larvae	Chaetognaths	Euphausiids	Mysids	Pelagic Amphipods	Gelatinous filter feeders	Pteropods	Copepods	Pelagic microbes	Benthic microbes
W. Pollock Ad	0	0	0	0	0	0	0	0	0	0	0	0	0	0	0	0	0	0	0	0	0	0	0	0	0	0	0	0	0	0
P. Cod Juv	0	0	0	0	0	0	0	0	0	0	0	0	0	0	0	0	0	0	0	0	0	0	0	0	0	0	0	0	0	0
P. Cod Ad	0	0	0	0	0	0	0	0	0	0	0	0	0	0	0	0	0	0	0	0	0	0	0	0	0	0	0	0	0	0
Herring Juv	0	0	0	0	0	0	0	0	0	0	0	0	0	0	0	0	0	0	0	0.001	0	0	0	0	0	0	0	0	0	0
Herring Ad	0	0	0	0	0	0	0	0	0	0	0	0	0	0	0	0	0	0	0	0	0	0	0	0	0	0	0	0	0	0
Arrowtooth Juv	0	0	0	0	0	0	0	0	0	0	0	0	0	0	0	0	0	0	0	0	0	0	0	0	0	0	0	0	0	0
Arrowtooth Ad	0	0	0	0	0	0	0	0	0	0	0	0	0	0	0	0	0	0	0	0	0	0	0	0	0	0	0	0	0	0
Kamchatka fl. Juv	0	0	0	0	0	0	0	0	0	0	0	0	0	0	0	0	0	0	0	0	0	0	0	0	0	0	0	0	0	0
Kamchatka fl. Ad	0	0	0	0	0	0	0	0	0	0	0	0	0	0	0	0	0	0	0	0	0	0	0	0	0	0	0	0	0	0
Gr. Turbot Juv	0	0	0	0	0	0	0	0	0	0	0	0	0	0	0	0	0	0	0	0	0	0	0	0	0	0	0	0	0	0
Gr. Turbot Ad	0	0	0	0	0	0	0	0	0	0	0	0	0	0	0	0	0	0	0	0	0	0	0	0	0	0	0	0	0	0
P. Halibut Juv	0	0	0	0	0	0	0	0	0	0	0	0	0	0	0	0	0	0	0	0	0	0	0	0	0	0	0	0	0	0
P. Halibut Ad	0	0	0	0	0	0	0	0	0	0	0	0	0	0	0	0	0	0	0	0	0	0	0	0	0	0	0	0	0	0
YF. Sole Juv	0	0	0	0	0	0	0	0	0	0	0	0	0	0	0	0	0	0	0	0	0	0	0	0	0	0	0	0	0	0
YF. Sole Ad	0	0	0	0	0	0	0	0	0	0	0	0	0	0	0	0	0	0	0	0	0	0	0	0	0	0	0	0	0	0
FH. Sole Juv	0	0	0	0	0	0	0	0	0	0	0	0	0	0	0	0	0	0	0	0	0	0	0	0	0	0	0	0	0	0
FH. Sole Ad	0	0	0	0	0	0	0	0	0	0	0	0	0	0	0	0	0	0	0	0	0	0	0	0	0	0	0	0	0	0
N. Rock sole Juv	0	0	0	0	0	0	0	0	0	0	0	0	0	0	0	0	0	0	0	0	0	0	0	0	0	0	0	0	0	0
N. Rock sole Ad	0	0	0	0	0	0	0	0	0	0	0	0	0	0	0	0	0	0	0	0	0	0	0	0	0	0	0	0	0	0
AK Plaice	0	0	0	0	0	0	0	0	0	0	0	0	0	0	0	0	0	0	0	0	0	0	0	0	0	0	0	0	0	0
Dover Sole	0	0	0	0	0	0	0	0	0	0	0	0	0	0	0	0	0	0	0	0	0	0	0	0	0	0	0	0	0	0
Rex Sole	0	0	0	0	0	0	0	0	0	0	0	0	0	0	0	0	0	0	0	0	0	0	0	0	0	0	0	0	0	0
Misc. Flatfish	0	0	0	0	0	0	0	0	0	0	0	0	0	0	0	0	0	0	0	0	0	0	0	0	0	0	0	0	0	0
Alaska skate	0	0	0	0	0	0	0	0	0	0	0	0	0	0	0	0	0	0	0	0	0	0	0	0	0	0	0	0	0	0
Other skates	0	0	0	0	0	0	0	0	0	0	0	0	0	0	0	0	0	0	0	0	0	0	0	0	0	0	0	0	0	0
Sablefish Juv	0	0	0	0	0	0	0	0	0	0	0	0	0	0	0	0	0	0	0	0	0	0	0	0	0	0	0	0	0	0
Sablefish Ad	0	0	0	0	0	0	0	0	0	0	0	0	0	0	0	0	0	0	0	0	0	0	0	0	0	0	0	0	0	0
Eelpouts	0	0	0	0	0	0	0	0	0	0	0	0	0	0	0	0	0	0	0	0	0	0	0	0	0	0	0	0	0	0
Grenadiers	0	0	0	0	0	0	0	0	0	0	0	0	0	0	0	0	0	0	0	0	0	0	0	0	0	0	0	0	0	0
Misc. fish deep	0	0	0	0	0	0	0	0	0	0	0	0	0	0	0	0	0	0	0	0	0	0	0	0	0	0	0	0	0	0
POP	0	0	0	0	0	0	0	0	0	0	0	0	0	0	0	0	0	0	0	0	0	0	0	0	0	0	0	0	0	0
Sharpchin Rock	0	0	0	0	0	0	0	0	0	0	0	0	0	0	0	0	0	0	0	0	0	0	0	0	0	0	0	0	0	0
Northern Rock	0	0	0	0	0	0	0	0	0	0	0	0	0	0	0	0	0	0	0	0	0	0	0	0	0	0	0	0	0	0
Dusky Rock	0	0	0	0	0	0	0	0	0	0	0	0	0	0	0	0	0	0	0	0	0	0	0	0	0	0	0	0	0	0
Shortraker Rock	0	0	0	0	0	0	0	0	0	0	0	0	0	0	0	0	0	0	0	0	0	0	0	0	0	0	0	0	0	0

Group	Pandalidae	NP shrimp	Sea stars	Brittle stars	Urchins dollars cucumbers	Snails	Hermit crabs	Misc. crabs	Misc. Crustacean	Benthic Amphipods	Anemones	Corals	Hydroids	Urochordata	Sea Pens	Sponges	Bivalves	Polychaetes	Misc. worms	Scyphozoid Jellies	Fish Larvae	Chaetognaths	Euphausiids	Mysids	Pelagic Amphipods	Gelatinous filter feeders	Pteropods	Copepods	Pelagic microbes	Benthic microbes
Rougheye Rock	0	0	0	0	0	0	0	0	0	0	0	0	0	0	0	0	0	0	0	0	0	0	0	0	0	0	0	0	0	0
Shortspine Thorns	0	0	0	0	0	0	0	0	0	0	0	0	0	0	0	0	0	0	0	0	0	0	0	0	0	0	0	0	0	0
Other Sebastes	0	0	0	0	0	0	0	0	0	0	0	0	0	0	0	0	0	0	0	0	0	0	0	0	0	0	0	0	0	0
Atka mackerel Juv	0	0	0	0	0	0	0	0	0	0	0	0	0	0	0	0	0	0	0	0	0	0	0	0	0	0	0	0	0	0
Atka mackerel Ad	0	0	0	0	0	0	0	0	0	0	0	0	0	0	0	0	0	0	0	0	0	0	0	0	0	0	0	0	0	0
Greenlings	0	0	0	0	0	0	0	0	0	0	0	0	0	0	0	0	0	0	0	0	0	0	0	0	0	0	0	0	0	0
Lg. Sculpins	0	0	0	0	0	0	0	0	0	0	0	0	0	0	0	0	0	0	0	0	0	0	0	0	0	0	0	0	0	0
Other sculpins	0	0	0	0	0	0	0	0	0	0	0	0	0	0	0	0	0	0	0	0	0	0	0	0	0	0	0	0	0	0
Misc. fish shallow	0	0	0	0	0	0	0	0	0	0	0	0	0	0	0	0	0	0	0	0	0	0	0	0	0	0	0	0	0	0
Octopi	0	0	0	0	0	0	0	0	0	0	0	0	0	0	0	0	0	0	0	0	0	0	0	0	0	0	0	0	0	0
Squids	0	0	0	0	0	0	0	0	0	0	0	0	0	0	0	0	0	0	0	0.001	0	0	0	0	0	0	0	0	0	0
Salmon returning	0	0	0	0	0	0	0	0	0	0	0	0	0	0	0	0	0	0	0	0	0	0	0	0	0	0	0	0	0	0
Salmon outgoing	0	0	0	0	0	0	0	0	0	0	0	0	0	0	0	0	0	0	0	0	0	0	0	0	0	0	0	0	0	0
Bathylagidae	0	0	0	0	0	0	0	0	0	0	0	0	0	0	0	0	0	0	0	0.001	0	0	0	0	0	0	0	0	0	0
Myctophidae	0	0	0	0	0	0	0	0	0	0	0	0	0	0	0	0	0	0	0	0.001	0	0	0	0	0	0	0	0	0	0
Capelin	0	0	0	0	0	0	0	0	0	0	0	0	0	0	0	0	0	0	0	0.001	0	0	0	0	0	0	0	0	0	0
Sandlance	0	0	0	0	0	0	0	0	0	0	0	0	0	0	0	0	0	0	0	0.001	0	0	0	0	0	0	0	0	0	0
Eulachon	0	0	0	0	0	0	0	0	0	0	0	0	0	0	0	0	0	0	0	0.001	0	0	0	0	0	0	0	0	0	0
Oth. managed forage	0	0	0	0	0	0	0	0	0	0	0	0	0	0	0	0	0	0	0	0.001	0	0	0	0	0	0	0	0	0	0
Oth. pelagic smelt	0	0	0	0	0	0	0	0	0	0	0	0	0	0	0	0	0	0	0	0.001	0	0	0	0	0	0	0	0	0	0
Bairdi	0	0	0.005	0	0	0	0	0	0	0	0	0	0	0	0	0	0	0	0	0	0	0	0	0	0	0	0	0	0	0
King Crab	0	0	0	0	0	0	0	0	0	0	0	0	0	0	0	0	0	0	0	0	0	0	0	0	0	0	0	0	0	0
Opilio	0	0	0.005	0	0	0	0	0	0	0	0	0	0	0	0	0	0	0	0	0	0	0	0	0	0	0	0	0	0	0
Pandalidae	0	0	0.005	0	0	0	0	0	0	0	0	0	0	0	0	0	0	0	0	0	0	0	0	0	0	0	0	0	0	0
NP shrimp	0	0	0.005	0	0	0	0	0	0	0	0	0	0	0	0	0	0	0	0	0	0	0	0	0	0	0	0	0	0	0
Sea stars	0	0	0	0	0	0	0	0	0	0	0	0	0	0	0	0	0	0	0	0	0	0	0	0	0	0	0	0	0	0
Brittle stars	0	0	0.03	0	0	0	0	0	0	0	0	0	0	0	0	0	0	0	0	0	0	0	0	0	0	0	0	0	0	0
Urchins dollars cucumbers	0	0	0	0	0	0	0	0	0	0	0	0	0	0	0	0	0	0	0	0	0	0	0	0	0	0	0	0	0	0
Snails	0	0	0.005	0	0	0	0	0	0	0	0	0	0	0	0	0	0	0	0	0	0	0	0	0	0	0	0	0	0	0
Hermit crabs	0	0	0.005	0	0	0	0	0	0	0	0	0	0	0	0	0	0	0	0	0	0	0	0	0	0	0	0	0	0	0
Misc. crabs	0	0	0	0	0	0	0	0	0	0	0	0	0	0	0	0	0	0	0	0	0	0	0	0	0	0	0	0	0	0
Misc. Crustacean	0	0	0	0	0	0	0	0	0	0	0.125	0.125	0.125	0.125	0.125	0.125	0.125	0	0	0	0	0	0	0	0	0	0	0	0	0
Benthic Amphipods	0.3	0.3	0	0.1	0	0	0	0	0	0	0.125	0.125	0.125	0.125	0.125	0.125	0.125	0	0	0	0	0	0	0	0	0	0	0	0	0
Anemones	0	0	0	0	0	0	0	0	0	0	0	0	0	0	0	0	0	0	0	0	0	0	0	0	0	0	0	0	0	0

Group	Pandalidae	NP shrimp	Sea stars	Brittle stars	Urchins dollars cucumbers	Snails	Hermit crabs	Misc. crabs	Misc. Crustacean	Benthic Amphipods	Anemones	Corals	Hydroids	Urochordata	Sea Pens	Sponges	Bivalves	Polychaetes	Misc. worms	Scyphozoid Jellies	Fish Larvae	Chaetognaths	Euphausiids	Mysids	Pelagic Amphipods	Gelatinous filter feeders	Pteropods	Copepods	Pelagic microbes	Benthic microbes
Corals	0	0	0	0	0	0	0	0	0	0	0	0	0	0	0	0	0	0	0	0	0	0	0	0	0	0	0	0	0	0
Hydroids	0	0	0	0	0	0	0	0	0	0	0	0	0	0	0	0	0	0	0	0	0	0	0	0	0	0	0	0	0	0
Urochordata	0	0	0	0	0	0	0	0	0	0	0	0	0	0	0	0	0	0	0	0	0	0	0	0	0	0	0	0	0	0
Sea Pens	0	0	0	0	0	0	0	0	0	0	0	0	0	0	0	0	0	0	0	0	0	0	0	0	0	0	0	0	0	0
Sponges	0	0	0	0	0	0	0	0	0	0	0	0	0	0	0	0	0	0	0	0	0	0	0	0	0	0	0	0	0	0
Bivalves	0.1	0.1	0.91	0	0	0.2	0.25	0.25	0	0	0	0	0	0	0	0	0	0	0	0	0	0	0	0	0	0	0	0	0	0
Polychaetes	0	0	0.02	0	0	0.2	0.25	0.25	0	0	0	0	0	0	0	0	0	0	0	0	0	0	0	0	0	0	0	0	0	0
Misc. worms	0	0	0.01	0	0	0.2	0.25	0.25	0	0	0	0	0	0	0	0	0	0	0	0	0	0	0	0	0	0	0	0	0	0
Scyphozoid Jellies	0	0	0	0	0	0	0	0	0	0	0	0	0	0	0	0	0	0	0	0	0	0	0	0	0	0	0	0	0	0
Fish Larvae	0	0	0	0	0	0	0	0	0	0	0	0	0	0	0	0	0	0	0	0.05	0	0	0	0	0	0	0	0	0	0
Chaetognaths	0	0	0	0	0	0	0	0	0	0	0	0	0	0	0	0	0	0	0	0	0	0	0	0	0	0	0	0	0	0
Euphausiids	0.2	0.2	0	0	0	0	0	0	0	0	0	0	0	0	0	0	0	0	0	0.64	0	0.05	0	0	0	0	0	0	0	0
Mysids	0	0	0	0	0	0	0	0	0	0	0	0	0	0	0	0	0	0	0	0	0	0.05	0	0	0	0	0	0	0	0
Pelagic Amphipods	0	0	0	0	0	0	0	0	0	0	0	0	0	0	0	0	0	0	0	0	0	0.05	0	0	0	0	0	0	0	0
Gelatinous filter feeders	0	0	0	0	0	0	0	0	0	0	0	0	0	0	0	0	0	0	0	0.05	0	0.05	0	0	0	0	0	0	0	0
Pteropods	0	0	0	0	0	0	0	0	0	0	0	0	0	0	0	0	0	0	0	0	0	0.05	0	0	0	0	0	0	0	0
Copepods	0	0	0	0	0	0	0	0	0	0	0	0	0	0	0	0	0	0	0	0.15	0.25	0.25	0.25	0.25	0.25	0.25	0.25	0	0	0
Pelagic microbes	0	0	0	0	0	0	0	0	0	0	0	0	0	0	0	0	0	0	0	0.05	0.15	0.15	0.15	0.15	0.15	0.15	0.15	0.5	0	0
Benthic microbes	0	0	0	0	0	0	0	0	0.5	0.5	0.15	0.15	0.15	0.15	0.15	0.15	0.15	0.5	0.5	0	0	0	0	0	0	0	0	0	0	0
Macroalgae	0	0	0	0	0.25	0.2	0	0	0	0	0	0	0	0	0	0	0	0	0	0	0	0	0	0	0	0	0	0	0	0
Lg Phytoplankton	0	0	0	0	0	0	0	0	0	0	0	0	0	0	0	0	0	0	0	0.05	0.5	0.25	0.5	0.5	0.5	0.5	0.5	0.25	0	0
Sm Phytoplankton	0	0	0	0	0	0	0	0	0	0	0	0	0	0	0	0	0	0	0	0	0.1	0.1	0.1	0.1	0.1	0.1	0.1	0.25	0.7	0
Outside Production	0	0	0	0	0	0	0	0	0	0	0	0	0	0	0	0	0	0	0	0	0	0	0	0	0	0	0	0	0	0
Discards	0	0	0	0	0	0	0	0	0	0	0	0	0	0	0	0	0	0	0	0	0	0	0	0	0	0	0	0	0	0
Offal	0	0	0	0	0	0	0	0	0	0	0	0	0	0	0	0	0	0	0	0	0	0	0	0	0	0	0	0	0	0
Pelagic Detritus	0	0	0	0	0	0	0	0	0	0	0	0	0	0	0	0	0	0	0	0	0	0	0	0	0	0	0	0	0.3	0
Benthic Detritus	0.4	0.4	0	0.9	0.75	0.2	0.25	0.25	0.5	0.5	0.6	0.6	0.6	0.6	0.6	0.6	0.6	0.5	0.5	0	0	0	0	0	0	0	0	0	0	1
Outside Detritus	0	0	0	0	0	0	0	0	0	0	0	0	0	0	0	0	0	0	0	0	0	0	0	0	0	0	0	0	0	0

Table S17: Landed catch for the eastern Bering Sea Ecopath model (converted from the Rpath version of the model; units tkm^{-2}year^{-1})

Group	Pollock Trawl	Cod Trawl	Cod Pots	Cod Longline	Atka Trawl	RSflats_TWL	YFSflats_TWL	ATFflats_TWL	FHSflats_TWL	Oth_Flatfish_TWL	Turbot Trawl	Turbot Longline	Sablefish Longline	Rockfish Trawl	Halibut Longline	Crab Pots	Salmon Fishery	Herring Fishery	Indigenous	Subsistence
Transient KIllers	0	0	0	0	0	0	0	0	0	0	0	0	0	0	0	0	0	0	0	0
Sperm and Beaked Whales	0	0	0	0	0	0	0	0	0	0	0	0	0	0	0	0	0	0	0	0
Resident Killers	0	0	0	0	0	0	0	0	0	0	0	0	0	0	0	0	0	0	0	0
Porpoises	0	0	0	0	0	0	0	0	0	0	0	0	0	0	0	0	0	0	0	0
Belugas	0	0	0	0	0	0	0	0	0	0	0	0	0	0	0	0	0	0	2.52E-05	0
Gray Whales	0	0	0	0	0	0	0	0	0	0	0	0	0	0	0	0	0	0	0	0
Humpbacks	0	0	0	0	0	0	0	0	0	0	0	0	0	0	0	0	0	0	0	0
Fin Whales	0	0	0	0	0	0	0	0	0	0	0	0	0	0	0	0	0	0	0	0
Sei Whales	0	0	0	0	0	0	0	0	0	0	0	0	0	0	0	0	0	0	0	0
Right Whales	0	0	0	0	0	0	0	0	0	0	0	0	0	0	0	0	0	0	0	0
Minke Whales	0	0	0	0	0	0	0	0	0	0	0	0	0	0	0	0	0	0	1.83E-07	0
Bowhead Whales	0	0	0	0	0	0	0	0	0	0	0	0	0	0	0	0	0	0	0	0
Sea Otters	0	0	0	0	0	0	0	0	0	0	0	0	0	0	0	0	0	0	2.97E-08	0
Walrus Bd Seals	0	0	0	0	0	0	0	0	0	0	0	0	0	0	0	0	0	0	3.09E-05	0
N Fur Seal Juv	0	0	0	0	0	0	0	0	0	0	0	0	0	0	0	0	0	0	0	0
N Fur Seal Ad	0	0	0	0	0	0	0	0	0	0	0	0	0	0	0	0	0	0	3.68E-05	0
Steller Sea Lions Juv	0	0	0	0	0	0	0	0	0	0	0	0	0	0	0	0	0	0	0	0
Steller Sea Lions Ad	0	0	0	0	0	0	0	0	0	0	0	0	0	0	0	0	0	0	4.90E-05	0
Resident Seals	0	0	0	0	0	0	0	0	0	0	0	0	0	0	0	0	0	0	1.48E-05	0
Wintering Seals	0	0	0	0	0	0	0	0	0	0	0	0	0	0	0	0	0	0	3.16E-05	0
Shearwater	3.00E-10	0	0	7.00E-10	0	0	0	0	0	0	0	0	0	0	0	0	0	0	0	0
Murres	5.40E-09	0	0	1.43E-08	0	0	0	0	0	0	0	0	0	0	0	0	0	0	0	0
Kittiwakes	4.00E-10	0	0	1.20E-09	0	0	0	0	0	0	0	0	0	0	0	0	0	0	0	0
Auklets	1.20E-09	0	0	3.10E-09	0	0	0	0	0	0	0	0	0	0	0	0	0	0	0	0
Puffins	3.00E-10	0	0	8.00E-10	0	0	0	0	0	0	0	0	0	0	0	0	0	0	0	0
Fulmars	3.00E-10	0	0	9.00E-10	0	0	0	0	0	0	0	0	0	0	0	0	0	0	0	0
Storm Petrels	0	0	0	0	0	0	0	0	0	0	0	0	0	0	0	0	0	0	0	0
Cormorants	1.00E-10	0	0	3.00E-10	0	0	0	0	0	0	0	0	0	0	0	0	0	0	0	0
Gulls	1.00E-10	0	0	2.00E-10	0	0	0	0	0	0	0	0	0	0	0	0	0	0	0	0
Albatross Jaeger	1.00E-10	0	0	2.00E-10	0	0	0	0	0	0	0	0	0	0	0	0	0	0	0	0
Sleeper shark	1.43E-05	0	0	1.11E-07	0	0	0	0	3.00E-10	0	0	1.06E-06	0	0	0	0	0	0	0	0
W. Pollock Juv	0	0	0	0	0	0	0	0	0	0	0	0	0	0	0	0	0	0	0	0

Group	Pollock Trawl	Cod Trawl	Cod Pots	Cod Longline	Atka Trawl	RSflats_TWL	YFSflats_TWL	ATFflats_TWL	FHSflats_TWL	Oth_Flatfish_TWL	Turbot Trawl	Turbot Longline	Sablefish Longline	Rockfish Trawl	Halibut Longline	Crab Pots	Salmon Fishery	Herring Fishery	Indigenous	Subsistence
W. Pollock Ad	3.91E-01	5.33E-03	1.50E-08	4.42E-04	3.15E-05	2.28E-03	3.91E-03	2.98E-05	6.67E-04	0	1.82E-07	2.69E-06	0	6.38E-06	0	0	0	0	0	0
P. Cod Juv	0	0	0	0	0	0	0	0	0	0	0	0	0	0	0	0	0	0	0	0
P. Cod Ad	2.96E-02	5.83E-02	9.14E-03	1.01E-01	1.14E-04	7.20E-03	1.56E-02	2.26E-05	2.04E-03	0	4.54E-05	1.08E-04	2.94E-05	5.96E-04	0	0	0	0	0	0
Herring Juv	0	0	0	0	0	0	0	0	0	0	0	0	0	0	0	0	0	0	0	0
Herring Ad	0	0	0	0	0	0	0	0	0	0	0	0	0	0	0	0	0	2.15E-02	0	0
Arrowtooth Juv	0	0	0	0	0	0	0	0	0	0	0	0	0	0	0	0	0	0	0	0
Arrowtooth Ad	3.26E-03	6.28E-04	5.57E-07	9.58E-04	2.28E-05	7.79E-05	2.94E-04	1.53E-04	1.50E-05	0	1.68E-04	1.45E-04	8.97E-06	1.50E-04	0	0	0	0	0	0
Kamchatka fl. Juv	0	0	0	0	0	0	0	0	0	0	0	0	0	0	0	0	0	0	0	0
Kamchatka fl. Ad	0	0	0	0	0	0	0	0	0	0	0	0	0	0	0	0	0	0	0	0
Gr. Turbot Juv	0	0	0	0	0	0	0	0	0	0	0	0	0	0	0	0	0	0	0	0
Gr. Turbot Ad	5.05E-05	1.47E-05	2.09E-06	1.46E-04	1.06E-05	5.93E-06	7.83E-05	2.67E-06	1.51E-05	0	1.82E-03	1.90E-03	3.03E-05	5.58E-05	0	0	0	0	0	0
P. Halibut Juv	0	0	0	0	0	0	0	0	0	0	0	0	0	0	0	0	0	0	0	0
P. Halibut Ad	0	0	0	0	0	0	0	0	0	0	0	0	0	0	8.05E-03	0	0	0	0	0
YF. Sole Juv	0	0	0	0	0	0	0	0	0	0	0	0	0	0	0	0	0	0	0	0
YF. Sole Ad	4.29E-04	1.07E-03	1.27E-08	6.06E-07	5.20E-08	2.54E-03	1.30E-01	1.18E-06	3.83E-03	0	1.46E-08	1.60E-08	0	1.46E-05	0	0	0	0	0	0
FH. Sole Juv	0	0	0	0	0	0	0	0	0	0	0	0	0	0	0	0	0	0	0	0
FH. Sole Ad	1.17E-03	3.28E-04	6.37E-08	1.42E-05	9.81E-06	6.94E-04	2.18E-03	1.33E-05	1.94E-03	0	2.78E-06	3.82E-07	2.89E-08	7.74E-05	0	0	0	0	0	0
N. Rock sole Juv	0	0	0	0	0	0	0	0	0	0	0	0	0	0	0	0	0	0	0	0
N. Rock sole Ad	2.62E-03	1.64E-03	5.22E-08	4.49E-06	5.18E-06	2.79E-02	5.97E-03	7.31E-06	1.61E-03	0	3.06E-08	0	0	7.50E-06	0	0	0	0	0	0
AK Plaice	1.24E-03	3.47E-04	6.73E-08	1.50E-05	1.04E-05	7.33E-04	2.30E-03	1.41E-05	2.05E-03	1.54E-06	2.94E-06	4.03E-07	3.05E-08	8.18E-05	0	0	0	0	0	0
Dover Sole	1.38E-08	0	0	0	0	0	0	2.41E-07	9.14E-08	0	0	0	0	0	0	0	0	0	0	0
Rex Sole	8.55E-05	1.71E-04	0	0	4.54E-05	2.87E-05	2.74E-05	4.46E-06	7.01E-05	0	5.10E-05	0	0	2.64E-05	0	0	0	0	0	0
Misc. Flatfish	2.95E-04	8.28E-05	1.60E-08	3.59E-06	2.47E-06	1.75E-04	5.49E-04	3.36E-06	4.90E-04	0	7.01E-07	9.61E-08	7.30E-09	1.95E-05	0	0	0	0	0	0
Alaska skate	2.05E-04	5.99E-05	2.12E-07	3.11E-03	0	5.00E-05	1.87E-04	1.94E-06	3.57E-05	0	1.17E-05	1.73E-06	0	3.14E-05	0	0	0	0	0	0
Other skates	3.32E-05	9.69E-06	3.43E-08	5.03E-04	0	8.08E-06	3.03E-05	3.14E-07	5.77E-06	0	1.89E-06	2.79E-07	0	5.07E-06	0	0	0	0	0	0
Sablefish Juv	0	0	0	0	0	0	0	0	0	0	0	0	0	0	0	0	0	0	0	0
Sablefish Ad	7.32E-06	1.46E-05	1.45E-05	1.93E-04	4.79E-05	1.93E-05	2.70E-06	3.08E-06	7.51E-06	0	3.37E-04	5.25E-04	1.16E-03	5.91E-06	3.35E-04	0	0	0	0	0
Eelpouts	0	0	0	0	0	0	0	0	0	0	0	0	0	0	0	0	0	0	0	0
Grenadiers	3.19E-05	0	1.91E-08	0	0	0	1.79E-07	1.56E-08	0	0	1.81E-07	4.07E-06	0	0	0	0	0	0	0	0
Misc. fish deep	0	0	0	0	0	0	0	0	0	0	0	0	0	0	0	0	0	0	0	0
POP	8.66E-05	4.06E-04	4.13E-08	4.15E-06	1.13E-04	1.33E-05	1.24E-06	1.01E-06	2.51E-05	0	5.45E-08	2.23E-06	4.50E-07	7.66E-03	0	0	0	0	0	0
Sharpchin Rock	1.74E-06	6.44E-06	1.00E-09	1.33E-06	1.01E-07	1.55E-07	2.60E-09	3.71E-07	4.80E-09	0	3.50E-09	2.77E-07	5.88E-07	6.18E-05	0	0	0	0	0	0
Northern Rock	3.30E-05	1.22E-04	1.85E-08	2.53E-05	1.91E-06	2.93E-06	4.88E-08	7.05E-06	9.13E-08	0	6.74E-08	5.26E-06	1.12E-05	1.17E-03	0	0	0	0	0	0
Dusky Rock	2.34E-06	2.05E-06	1.38E-08	2.10E-06	0	0	9.29E-08	1.36E-08	0	0	0	8.56E-08	4.24E-08	0	0	0	0	0	0	0
Shortraker Rock	8.54E-06	9.47E-07	7.00E-10	9.82E-06	6.66E-06	0	1.03E-08	1.10E-06	1.73E-06	0	2.34E-05	7.72E-06	3.84E-06	1.80E-04	0	0	0	0	0	0

Group	Pollock Trawl	Cod Trawl	Cod Pots	Cod Longline	Atka Trawl	RSflats_TWL	YFSflats_TWL	ATFflats_TWL	FHSflats_TWL	Oth_Flatfish_TWL	Turbot Trawl	Turbot Longline	Sablefish Longline	Rockfish Trawl	Halibut Longline	Crab Pots	Salmon Fishery	Herring Fishery	Indigenous	Subsistence
Rougheye Rock	5.57E-07	1.25E-07	0	6.09E-06	4.57E-06	0	6.90E-09	6.08E-07	7.13E-07	0	1.47E-05	3.82E-06	2.72E-06	1.19E-04	0	0	0	0	0	0
Shortspine Thorns	4.27E-06	6.74E-06	3.40E-09	1.22E-06	3.31E-06	0	2.59E-07	1.30E-06	3.59E-06	0	2.43E-05	1.23E-05	7.04E-05	0	0	0	0	0	0	0
Other Sebastes	1.12E-05	7.75E-06	9.78E-07	5.49E-05	9.99E-06	7.56E-06	2.29E-06	6.72E-06	3.29E-06	0	6.63E-05	3.21E-05	5.06E-05	7.84E-05	0	0	0	0	0	0
Atka mackerel Juv	0	0	0	0	0	0	0	0	0	0	0	0	0	0	0	0	0	0	0	0
Atka mackerel Ad	5.31E-05	2.02E-05	9.67E-08	1.65E-07	1.63E-03	1.44E-05	1.56E-07	1.19E-05	7.38E-06	0	1.22E-04	0	0	1.68E-04	0	0	0	0	0	0
Greenlings	0	0	0	0	0	0	0	0	0	0	0	0	0	0	0	0	0	0	0	0
Lg. Sculpins	8.05E-05	1.28E-05	2.12E-06	9.62E-06	0	1.68E-05	4.46E-04	0	5.05E-06	0	1.62E-07	6.00E-10	0	0	0	0	0	0	0	0
Other sculpins	0	0	0	0	0	0	0	0	0	0	0	0	0	0	0	0	0	0	0	0
Misc. fish shallow	9.52E-06	8.93E-07	1.04E-08	8.31E-08	0	2.88E-08	7.22E-07	0	0	0	0	0	0	0	0	0	0	0	0	0
Octopi	1.45E-06	6.18E-06	4.27E-05	2.97E-06	0	2.59E-05	6.61E-07	0	0	0	0	6.70E-09	0	0	0	0	0	0	0	0
Squids	3.58E-04	9.62E-05	0	0	6.40E-09	0	8.24E-08	6.00E-10	6.52E-07	0	3.37E-07	0	0	9.75E-08	0	0	0	0	0	0
Salmon returning	0	0	0	0	0	0	0	0	0	0	0	0	0	0	0	0	1.69E-01	0	0	2.01E-06
Salmon outgoing	0	0	0	0	0	0	0	0	0	0	0	0	0	0	0	0	0	0	0	0
Bathylagidae	0	0	0	0	0	0	0	0	0	0	0	0	0	0	0	0	0	0	0	0
Myctophidae	2.32E-07	0	0	0	0	0	0	0	0	0	0	0	0	0	0	0	0	0	0	0
Capelin	1.24E-08	0	0	0	0	0	0	0	0	0	0	0	0	0	0	0	0	0	0	0
Sandlance	4.00E-10	0	0	0	0	0	0	0	0	0	0	0	0	0	0	0	0	0	0	0
Eulachon	1.61E-06	0	0	0	0	0	0	0	0	0	0	0	0	0	0	0	0	0	0	0
Oth. managed forage	4.02E-07	0	0	0	0	0	0	0	0	0	0	0	0	0	0	0	0	0	0	0
Oth. pelagic smelt	5.76E-05	0	0	0	0	1.60E-08	9.90E-08	0	1.70E-09	0	0	0	0	0	0	0	0	0	0	0
Bairdi	0	0	0	0	0	0	0	0	0	0	0	0	0	0	0	4.74E-02	0	0	0	0
King Crab	0	0	0	0	0	0	0	0	0	0	0	0	0	0	0	1.88E-02	0	0	0	0
Opilio	0	0	0	0	0	0	0	0	0	0	0	0	0	0	0	3.01E-01	0	0	0	0
Pandalidae	1.62E-07	0	1.00E-10	0	0	0	4.27E-08	0	1.96E-08	0	0	0	0	0	0	0	0	0	0	0
NP shrimp	0	0	0	0	0	0	0	0	0	0	0	0	0	0	0	0	0	0	0	0
Sea stars	4.18E-06	3.08E-08	2.00E-08	2.54E-07	0	8.16E-06	2.56E-05	0	2.04E-07	0	2.08E-07	2.20E-09	0	0	0	0	0	0	0	0
Brittle stars	0	0	0	0	0	0	0	0	0	0	0	0	0	0	0	0	0	0	0	0
Urchins dollars cucumbers	3.94E-08	2.25E-08	2.00E-10	0	0	4.32E-08	2.43E-07	0	0	0	0	0	0	0	0	0	0	0	0	0
Snails	6.70E-09	0	1.87E-08	0	0	0	1.63E-07	0	0	0	0	0	0	0	0	0	0	0	0	0
Hermit crabs	1.46E-07	5.47E-08	8.46E-08	4.80E-09	0	1.33E-06	1.66E-06	0	1.94E-08	0	0	0	0	0	0	0	0	0	0	0
Misc. crabs	0	0	0	0	0	0	0	0	0	0	0	0	0	0	0	3.46E-04	0	0	0	0
Misc. Crustacean	0	0	0	0	0	0	0	0	0	0	0	0	0	0	0	0	0	0	0	0
Benthic Amphipods	0	0	0	0	0	0	0	0	0	0	0	0	0	0	0	0	0	0	0	0
Anemones	1.27E-07	0	0	1.51E-07	0	1.25E-07	2.62E-08	0	2.00E-09	0	0	0	0	0	0	0	0	0	0	0

Group	Pollock Trawl	Cod Trawl	Cod Pots	Cod Longline	Atka Trawl	RSflats_TWL	YFSflats_TWL	ATFflats_TWL	FHSflats_TWL	Oth_Flatfish_TWL	Turbot Trawl	Turbot Longline	Sablefish Longline	Rockfish Trawl	Halibut Longline	Crab Pots	Salmon Fishery	Herring Fishery	Indigenous	Subsistence
Corals	0	1.93E-08	0	0	0	0	1.26E-07	0	0	0	0	0	0	0	0	0	0	0	0	0
Hydroids	0	0	0	0	0	0	0	0	0	0	0	0	0	0	0	0	0	0	0	0
Urochordata	0	0	0	0	0	1.96E-06	4.02E-06	0	1.08E-07	0	0	0	0	0	0	0	0	0	0	0
Sea Pens	1.80E-07	0	0	0	0	0	0	0	0	0	0	0	0	0	0	0	0	0	0	0
Sponges	3.00E-09	1.47E-06	1.03E-08	0	0	0	4.34E-07	0	5.00E-10	0	0	0	0	0	0	0	0	0	0	0
Bivalves	0	0	2.33E-08	0	0	0	0	0	0	0	0	0	0	0	0	0	0	0	0	0
Polychaetes	0	0	0	0	0	0	0	0	0	0	0	0	0	0	0	0	0	0	0	0
Misc. worms	0	0	0	0	0	0	0	0	0	0	0	0	0	0	0	0	0	0	0	0
Scyphozoid Jellies	3.33E-03	7.84E-07	2.44E-08	0	0	3.72E-06	2.91E-05	0	1.03E-08	0	0	0	0	0	0	0	0	0	0	0
Fish Larvae	0	0	0	0	0	0	0	0	0	0	0	0	0	0	0	0	0	0	0	0
Chaetognaths	0	0	0	0	0	0	0	0	0	0	0	0	0	0	0	0	0	0	0	0
Euphausiids	0	0	0	0	0	0	0	0	0	0	0	0	0	0	0	0	0	0	0	0
Mysids	0	0	0	0	0	0	0	0	0	0	0	0	0	0	0	0	0	0	0	0
Pelagic Amphipods	0	0	0	0	0	0	0	0	0	0	0	0	0	0	0	0	0	0	0	0
Gelatinous filter feeders	0	0	0	0	0	0	0	0	0	0	0	0	0	0	0	0	0	0	0	0
Pteropods	0	0	0	0	0	0	0	0	0	0	0	0	0	0	0	0	0	0	0	0
Copepods	0	0	0	0	0	0	0	0	0	0	0	0	0	0	0	0	0	0	0	0
Pelagic microbes	0	0	0	0	0	0	0	0	0	0	0	0	0	0	0	0	0	0	0	0
Benthic microbes	0	0	0	0	0	0	0	0	0	0	0	0	0	0	0	0	0	0	0	0
Macroalgae	0	0	0	0	0	0	0	0	0	0	0	0	0	0	0	0	0	0	0	0
Lg Phytoplankton	0	0	0	0	0	0	0	0	0	0	0	0	0	0	0	0	0	0	0	0
Sm Phytoplankton	0	0	0	0	0	0	0	0	0	0	0	0	0	0	0	0	0	0	0	0
Outside Production	0	0	0	0	0	0	0	0	0	0	0	0	0	0	0	0	0	0	0	0
Discards	0	0	0	0	0	0	0	0	0	0	0	0	0	0	0	0	0	0	0	0
Offal	0	0	0	0	0	0	0	0	0	0	0	0	0	0	0	0	0	0	0	0
Pelagic Detritus	0	0	0	0	0	0	0	0	0	0	0	0	0	0	0	0	0	0	0	0
Benthic Detritus	0	0	0	0	0	0	0	0	0	0	0	0	0	0	0	0	0	0	0	0
Outside Detritus	0	0	0	0	0	0	0	0	0	0	0	0	0	0	0	0	0	0	0	0

Table S18: Discards for the eastern Bering Sea Ecopath model (converted from the Rpath version of the model; units $tkm^{-2}year^{-1}$)

Group	Pollock Trawl	Cod Trawl	Cod Pots	Cod Longline	Atka Trawl	RSflats_TWL	YFSflats_TWL	ATFflats_TWL	FHSflats_TWL	Oth_Flatfish_TWL	Turbot Trawl	Turbot Longline	Sablefish Longline	Rockfish Trawl	Halibut Longline	Crab Pots	Salmon Fishery	Herring Fishery	Indigenous	Subsistence
Transient KIllers	0	0	0	0	0	0	0	0	0	0	0	0	0	0	0	0	0	0	0	0
Sperm and Beaked Whales	0	0	0	0	0	0	0	0	0	0	0	0	0	0	0	0	0	0	0	0
Resident Killers	2.29E-06	5.74E-07	0	1.24E-06	5.16E-08	6.27E-07	7.84E-07	3.92E-08	1.18E-07	0	4.95E-08	7.90E-09	6.00E-10	5.19E-08	0	0	0	0	0	0
Porpoises	3.49E-07	8.73E-08	0	1.82E-07	7.80E-09	9.54E-08	1.19E-07	6.00E-09	1.79E-08	0	7.50E-09	1.00E-09	1.00E-10	7.90E-09	0	0	0	0	0	0
Belugas	0	0	0	0	0	0	0	0	0	0	0	0	0	0	0	0	0	0	2.52E-08	0
Gray Whales	0	0	0	0	0	0	0	0	0	0	0	0	0	0	0	0	0	0	0	0
Humpbacks	8.35E-06	2.09E-06	0	0	1.88E-07	2.28E-06	2.85E-06	1.43E-07	4.28E-07	0	1.80E-07	0	0	1.89E-07	0	0	0	0	0	0
Fin Whales	0	0	0	0	0	0	0	0	0	0	0	0	0	0	0	0	0	0	0	0
Sei Whales	0	0	0	0	0	0	0	0	0	0	0	0	0	0	0	0	0	0	0	0
Right Whales	0	0	0	0	0	0	0	0	0	0	0	0	0	0	0	0	0	0	0	0
Minke Whales	1.20E-06	3.00E-07	0	0	2.70E-08	3.28E-07	4.11E-07	2.05E-08	6.16E-08	0	2.59E-08	0	0	2.72E-08	0	0	0	0	2.00E-10	0
Bowhead Whales	0	0	0	0	0	0	0	0	0	0	0	0	0	0	0	0	0	0	0	0
Sea Otters	0	0	0	0	0	0	0	0	0	0	0	0	0	0	0	0	0	0	0	0
Walrus Bd Seals	2.96E-06	7.41E-07	0	0	6.66E-08	8.10E-07	1.01E-06	5.06E-08	1.52E-07	0	6.39E-08	0	0	6.71E-08	0	0	0	0	3.09E-08	0
N Fur Seal Juv	0	0	0	0	0	0	0	0	0	0	0	0	0	0	0	0	0	0	0	0
N Fur Seal Ad	6.15E-08	1.54E-08	0	0	1.40E-09	1.68E-08	2.10E-08	1.10E-09	3.20E-09	0	1.30E-09	0	0	1.40E-09	0	0	0	0	3.68E-08	0
Steller Sea Lions Juv	0	0	0	0	0	0	0	0	0	0	0	0	0	0	0	0	0	0	0	0
Steller Sea Lions Ad	7.50E-07	1.53E-07	0	6.40E-08	1.25E-08	1.80E-07	2.18E-07	1.10E-08	3.30E-08	0	1.68E-08	3.50E-10	2.50E-11	1.25E-08	0	0	0	0	1.54E-08	0
Resident Seals	9.33E-08	2.33E-08	3.29E-08	4.36E-08	2.10E-09	2.55E-08	3.19E-08	1.60E-09	4.80E-09	0	2.00E-09	3.00E-10	0	2.10E-09	0	0	0	0	1.48E-08	0
Wintering Seals	1.30E-07	3.25E-08	0	2.34E-08	2.90E-09	3.55E-08	4.44E-08	2.20E-09	6.70E-09	0	2.80E-09	1.00E-10	0	2.90E-09	0	0	0	0	3.16E-08	0
Shearwater	4.71E-08	6.00E-09	3.00E-09	1.45E-06	1.50E-09	0	2.90E-09	0	0	0	1.60E-09	2.72E-08	1.37E-08	0	0	0	0	0	0	0
Murres	9.60E-07	1.23E-07	6.10E-08	2.95E-05	3.02E-08	0	5.81E-08	0	0	0	3.17E-08	5.53E-07	2.79E-07	0	0	0	0	0	0	0
Kittiwakes	7.81E-08	1.00E-08	5.00E-09	2.40E-06	2.50E-09	0	4.70E-09	0	0	0	2.60E-09	4.50E-08	2.27E-08	0	0	0	0	0	0	0
Auklets	2.07E-07	2.64E-08	1.31E-08	6.35E-06	6.50E-09	0	1.25E-08	0	0	0	6.80E-09	1.19E-07	6.01E-08	0	0	0	0	0	0	0
Puffins	5.60E-08	7.20E-09	3.60E-09	1.72E-06	1.80E-09	0	3.40E-09	0	0	0	1.90E-09	3.23E-08	1.63E-08	0	0	0	0	0	0	0
Fulmars	6.13E-08	7.80E-09	3.90E-09	1.88E-06	1.90E-09	0	3.70E-09	0	0	0	2.00E-09	3.53E-08	1.78E-08	0	0	0	0	0	0	0
Storm Petrels	2.00E-10	0	0	6.30E-09	0	0	0	0	0	0	0	1.00E-10	1.00E-10	0	0	0	0	0	0	0
Cormorants	1.77E-08	2.30E-09	1.10E-09	5.42E-07	6.00E-10	0	1.10E-09	0	0	0	6.00E-10	1.02E-08	5.10E-09	0	0	0	0	0	0	0
Gulls	1.24E-08	1.60E-09	8.00E-10	3.80E-07	4.00E-10	0	7.00E-10	0	0	0	4.00E-10	7.10E-09	3.60E-09	0	0	0	0	0	0	0
Albatross Jaeger	1.18E-08	1.50E-09	7.00E-10	3.62E-07	4.00E-10	0	7.00E-10	0	0	0	4.00E-10	6.80E-09	3.40E-09	0	0	0	0	0	0	0
Sleeper shark	2.39E-04	3.03E-05	0	1.81E-04	0	0	1.69E-07	5.06E-07	4.90E-06	0	8.68E-06	4.81E-05	2.53E-05	5.71E-05	1.01E-04	0	0	0	0	0
W. Pollock Juv	0	0	0	0	0	0	0	0	0	0	0	0	0	0	0	0	0	0	0	0

Group	Pollock Trawl	Cod Trawl	Cod Pots	Cod Longline	Atka Trawl	RSflats_TWL	YFSflats_TWL	ATFflats_TWL	FHSflats_TWL	Oth_Flatfish_TWL	Turbot Trawl	Turbot Longline	Sablefish Longline	Rockfish Trawl	Halibut Longline	Crab Pots	Salmon Fishery	Herring Fishery	Indigenous	Subsistence
W. Pollock Ad	1.96E+00	4.90E-02	1.01E-05	5.91E-03	2.29E-04	3.20E-02	4.48E-02	1.06E-04	7.60E-03	0	1.55E-05	9.45E-07	4.63E-07	7.77E-04	0	0	0	0	0	0
P. Cod Juv	0	0	0	0	0	0	0	0	0	0	0	0	0	0	0	0	0	0	0	0
P. Cod Ad	1.24E-02	6.21E-02	8.97E-03	1.01E-01	2.55E-05	4.60E-03	7.63E-03	8.05E-07	6.98E-04	0	5.26E-07	5.52E-06	3.25E-06	9.18E-05	9.13E-04	0	0	0	0	0
Herring Juv	0	0	0	0	0	0	0	0	0	0	0	0	0	0	0	0	0	0	0	0
Herring Ad	6.36E-03	3.11E-06	0	0	0	5.02E-05	1.13E-03	7.81E-08	6.37E-05	0	0	0	0	3.89E-07	0	0	0	2.15E-05	0	0
Arrowtooth Juv	0	0	0	0	0	0	0	0	0	0	0	0	0	0	0	0	0	0	0	0
Arrowtooth Ad	6.59E-03	5.96E-03	3.78E-06	3.27E-03	2.45E-04	3.26E-03	2.99E-03	1.02E-04	4.29E-03	0	1.00E-03	3.62E-04	2.33E-04	1.63E-03	2.29E-04	0	0	0	0	0
Kamchatka fl. Juv	0	0	0	0	0	0	0	0	0	0	0	0	0	0	0	0	0	0	0	0
Kamchatka fl. Ad	0	0	0	0	0	0	0	0	0	0	0	0	0	0	0	0	0	0	0	0
Gr. Turbot Juv	0	0	0	0	0	0	0	0	0	0	0	0	0	0	0	0	0	0	0	0
Gr. Turbot Ad	2.88E-04	8.39E-05	6.56E-06	4.74E-04	4.13E-05	3.40E-05	3.05E-05	2.67E-06	2.65E-04	0	7.67E-04	8.31E-04	1.95E-03	4.00E-05	0	0	0	0	0	0
P. Halibut Juv	0	0	0	0	0	0	0	0	0	0	0	0	0	0	0	0	0	0	0	0
P. Halibut Ad	2.37E-03	3.93E-03	6.53E-06	8.08E-04	1.96E-05	2.01E-03	1.17E-03	8.95E-05	9.30E-05	0	4.60E-04	1.30E-06	5.39E-05	2.08E-04	2.00E-04	0	0	0	0	0
YF. Sole Juv	0	0	0	0	0	0	0	0	0	0	0	0	0	0	0	0	0	0	0	0
YF. Sole Ad	1.36E-03	1.34E-03	2.57E-05	1.34E-04	9.35E-05	5.80E-03	8.68E-02	4.92E-06	3.16E-03	0	3.58E-08	1.80E-08	5.94E-08	1.35E-05	0	0	0	0	0	0
FH. Sole Juv	0	0	0	0	0	0	0	0	0	0	0	0	0	0	0	0	0	0	0	0
FH. Sole Ad	4.59E-03	2.38E-03	4.97E-07	1.98E-04	4.41E-05	3.05E-03	9.66E-03	4.34E-06	2.10E-03	0	9.67E-06	2.90E-05	6.14E-07	7.27E-05	0	0	0	0	0	0
N. Rock sole Juv	0	0	0	0	0	0	0	0	0	0	0	0	0	0	0	0	0	0	0	0
N. Rock sole Ad	8.75E-03	7.97E-03	5.35E-07	2.99E-05	6.29E-05	4.13E-02	1.31E-02	5.19E-06	1.51E-03	0	7.66E-07	1.91E-06	3.50E-08	1.85E-05	0	0	0	0	0	0
AK Plaice	4.85E-03	2.52E-03	5.25E-07	2.09E-04	4.66E-05	3.22E-03	1.02E-02	4.59E-06	2.22E-03	2.49E-06	1.02E-05	3.06E-05	6.49E-07	7.68E-05	0	0	0	0	0	0
Dover Sole	1.10E-08	6.89E-08	0	7.50E-09	9.00E-10	6.40E-09	6.02E-07	7.61E-08	6.93E-08	0	1.48E-08	1.11E-08	0	5.36E-08	0	0	0	0	0	0
Rex Sole	7.41E-05	2.23E-05	2.20E-09	0	9.02E-06	1.25E-05	9.39E-06	1.39E-06	2.49E-05	0	1.88E-05	0	0	0	0	0	0	0	0	0
Misc. Flatfish	1.16E-03	6.00E-04	1.25E-07	4.98E-05	1.11E-05	7.69E-04	2.44E-03	1.09E-06	5.30E-04	0	2.44E-06	7.30E-06	1.55E-07	1.83E-05	0	0	0	0	0	0
Alaska skate	8.49E-04	1.65E-03	2.77E-08	1.45E-02	9.53E-06	1.09E-03	1.84E-03	7.94E-06	3.26E-04	0	4.96E-05	9.42E-05	1.19E-04	7.31E-05	6.94E-06	0	0	0	0	0
Other skates	1.37E-04	2.67E-04	4.50E-09	2.34E-03	1.54E-06	1.76E-04	2.98E-04	1.28E-06	5.26E-05	0	8.01E-06	1.52E-05	1.92E-05	1.18E-05	1.18E-04	0	0	0	0	0
Sablefish Juv	0	0	0	0	0	0	0	0	0	0	0	0	0	0	0	0	0	0	0	0
Sablefish Ad	3.94E-06	5.69E-07	3.41E-08	2.07E-05	9.26E-06	3.45E-06	1.63E-08	1.21E-06	1.41E-05	0	2.45E-05	5.18E-06	6.38E-06	4.96E-06	0	0	0	0	0	0
Eelpouts	0	0	0	0	0	0	0	0	0	0	0	0	0	0	0	0	0	0	0	0
Grenadiers	9.05E-05	6.13E-06	6.98E-06	7.38E-04	1.84E-06	0	1.47E-06	7.28E-07	7.80E-06	0	6.97E-05	1.62E-03	1.07E-03	6.46E-04	0	0	0	0	0	0
Misc. fish deep	0	0	0	0	0	0	0	0	0	0	0	0	0	0	0	0	0	0	0	0
POP	4.44E-04	5.24E-04	7.70E-09	8.60E-06	4.04E-05	7.43E-05	4.81E-06	4.96E-07	2.12E-04	0	2.92E-07	2.66E-07	7.96E-08	6.49E-04	3.90E-06	0	0	0	0	0
Sharpchin Rock	2.26E-06	7.14E-06	8.10E-09	7.42E-07	4.21E-06	6.77E-08	9.00E-10	2.27E-07	2.49E-08	0	2.66E-08	2.30E-09	3.56E-07	7.13E-06	3.90E-06	0	0	0	0	0
Northern Rock	4.29E-05	1.36E-04	1.53E-07	1.41E-05	7.99E-05	1.29E-06	1.74E-08	2.56E-06	4.74E-07	0	5.06E-07	4.36E-08	6.76E-06	1.35E-04	3.90E-06	0	0	0	0	0
Dusky Rock	3.43E-06	3.09E-06	2.50E-09	5.33E-07	1.89E-06	1.41E-07	0	0	4.14E-07	0	0	0	0	0	3.90E-06	0	0	0	0	0
Shortraker Rock	1.00E-10	0	0	6.00E-10	2.00E-10	4.00E-10	0	0	1.00E-10	0	0	2.00E-10	3.00E-10	1.70E-09	3.90E-06	0	0	0	0	0

Group	Pollock Trawl	Cod Trawl	Cod Pots	Cod Longline	Atka Trawl	RSflats_TWL	YFSflats_TWL	ATFflats_TWL	FHSflats_TWL	Oth_Flatfish_TWL	Turbot Trawl	Turbot Longline	Sablefish Longline	Rockfish Trawl	Halibut Longline	Crab Pots	Salmon Fishery	Herring Fishery	Indigenous	Subsistence
Rougheye Rock	1.00E-10	0	0	4.00E-10	1.00E-10	3.00E-10	0	0	1.00E-10	0	0	1.00E-10	2.00E-10	1.10E-09	3.90E-06	0	0	0	0	0
Shortspine Thorns	3.55E-06	7.09E-07	0	1.86E-07	0	0	0	2.32E-08	1.75E-07	0	2.11E-07	3.34E-07	3.24E-07	0	3.90E-06	0	0	0	0	0
Other Sebastes	4.02E-05	9.01E-05	1.53E-06	5.90E-05	6.91E-05	6.29E-06	1.77E-06	1.18E-06	2.53E-06	0	8.19E-06	3.77E-06	4.19E-05	5.26E-05	3.90E-06	0	0	0	0	0
Atka mackerel Juv	4.00E-10	0	0	0	0	0	0	0	0	0	0	0	0	0	0	0	0	0	0	0
Atka mackerel Ad	3.18E-04	2.92E-03	3.76E-04	1.51E-03	3.18E-05	7.77E-04	3.10E-03	3.71E-06	1.93E-04	0	2.58E-05	1.20E-06	5.08E-06	9.76E-05	2.40E-05	0	0	0	0	0
Greenlings	0	0	0	0	0	0	0	0	0	0	0	0	0	0	0	0	0	0	0	0
Lg. Sculpins	9.84E-06	2.81E-06	5.96E-08	1.35E-07	5.98E-06	6.38E-06	5.64E-07	0	2.65E-07	0	9.75E-06	1.59E-08	0	0	0	0	0	0	0	0
Other sculpins	2.28E-06	7.30E-05	6.73E-05	3.12E-05	1.13E-07	5.11E-05	2.27E-06	6.60E-08	6.81E-07	0	5.05E-07	5.94E-08	2.90E-08	0	0	0	0	0	0	0
Misc. fish shallow	7.85E-04	5.85E-05	1.71E-07	1.47E-08	1.94E-06	1.44E-07	3.43E-06	4.78E-07	3.05E-06	0	8.03E-06	6.00E-10	4.40E-09	1.45E-05	0	0	0	0	0	0
Octopi	4.20E-04	5.39E-05	0	7.91E-07	2.39E-08	9.34E-06	8.87E-06	4.76E-07	5.16E-07	0	1.05E-07	0	7.90E-09	6.17E-06	0	0	1.69E-04	0	0	2.01E-06
Squids	0	0	0	0	0	0	0	0	0	0	0	0	0	0	0	0	0	0	0	0
Salmon returning	0	0	0	0	0	0	0	0	0	0	0	0	0	0	0	0	0	0	0	0
Salmon outgoing	1.81E-07	0	1.10E-09	0	0	0	9.40E-09	0	3.00E-10	0	9.60E-09	0	0	0	0	0	0	0	0	0
Bathylagidae	4.00E-10	0	0	0	0	0	0	0	0	0	0	0	0	0	0	0	0	0	0	0
Myctophidae	1.40E-09	0	0	0	0	9.90E-09	3.31E-08	0	0	0	0	0	0	0	0	0	0	0	0	0
Capelin	1.93E-06	0	0	0	4.60E-09	0	4.80E-09	0	0	0	0	0	0	0	0	0	0	0	0	0
Sandlance	1.64E-07	2.25E-06	0	1.02E-08	0	9.17E-07	4.37E-07	0	1.06E-06	0	0	0	0	0	0	0	0	0	0	0
Eulachon	4.15E-05	1.33E-06	0	0	0	2.21E-06	5.79E-06	2.02E-08	3.77E-07	0	1.57E-08	0	0	0	0	0	0	0	0	0
Oth. managed forage	5.13E-04	3.27E-04	1.26E-04	4.56E-06	2.50E-08	4.12E-04	3.65E-04	1.09E-06	1.31E-04	0	5.66E-06	3.00E-10	1.70E-09	2.39E-06	0	4.74E-05	0	0	0	0
Oth. pelagic smelt	7.98E-05	1.41E-05	2.01E-04	7.68E-07	1.45E-07	3.75E-04	9.46E-05	1.51E-06	1.06E-05	0	2.55E-06	9.50E-09	4.28E-07	3.75E-07	0	1.88E-05	0	0	0	0
Bairdi	2.62E-03	1.55E-04	2.34E-05	3.54E-05	4.46E-07	1.00E-03	2.02E-03	2.64E-06	5.28E-04	0	1.42E-04	1.00E-09	5.93E-08	2.80E-06	0	3.01E-04	0	0	0	0
King Crab	7.21E-08	1.14E-07	8.00E-10	2.00E-10	9.50E-09	1.50E-07	1.11E-06	2.19E-08	3.34E-07	0	1.03E-07	0	0	7.44E-08	0	0	0	0	0	0
Opilio	0	0	0	0	0	0	0	0	0	0	0	0	0	0	0	0	0	0	0	0
Pandalidae	2.84E-05	2.13E-04	2.74E-05	3.12E-04	7.29E-07	1.26E-03	4.48E-03	3.38E-06	1.36E-04	0	1.11E-05	3.15E-07	8.80E-07	1.62E-05	1.66E-06	0	0	0	0	0
NP shrimp	0	0	0	0	0	0	0	0	0	0	0	0	0	0	0	0	0	0	0	0
Sea stars	1.58E-06	2.47E-05	7.08E-07	1.76E-06	1.26E-06	2.69E-05	7.22E-06	1.82E-08	6.58E-07	0	5.04E-07	2.78E-08	2.43E-07	3.11E-08	0	0	0	0	0	0
Brittle stars	3.82E-07	8.40E-07	0	0	0	5.71E-07	1.62E-05	0	0	0	0	0	0	0	0	0	0	0	0	0
Urchins dollars cucumbers	4.56E-06	1.62E-05	2.12E-06	9.16E-07	5.50E-07	5.56E-05	2.05E-04	9.65E-08	6.52E-06	0	1.89E-07	1.41E-08	4.00E-10	2.96E-07	0	0	0	0	0	0
Snails	0	0	0	0	0	0	0	0	0	0	0	0	0	0	0	3.46E-07	0	0	0	0
Hermit crabs	0	0	0	0	0	0	0	0	0	0	0	0	0	0	0	0	0	0	0	0
Misc. crabs	0	0	0	0	0	0	0	0	0	0	0	0	0	0	0	0	0	0	0	0
Misc. Crustacean	4.53E-06	2.71E-05	3.54E-08	2.22E-04	2.99E-07	7.00E-05	4.96E-05	1.77E-07	6.69E-06	0	2.47E-06	9.21E-08	2.05E-06	2.99E-08	0	0	0	0	0	0
Benthic Amphipods	1.24E-07	2.72E-06	6.54E-08	2.20E-06	1.05E-08	1.28E-06	1.12E-05	0	2.09E-07	0	8.84E-08	2.84E-08	2.45E-07	4.30E-07	0	0	0	0	0	0
Anemones	0	0	0	0	0	0	0	0	0	0	0	0	0	0	0	0	0	0	0	0

Group	Pollock Trawl	Cod Trawl	Cod Pots	Cod Longline	Atka Trawl	RSflats_TWL	YFSflats_TWL	ATFflats_TWL	FHSflats_TWL	Oth_Flatfish_TWL	Turbot Trawl	Turbot Longline	Sablefish Longline	Rockfish Trawl	Halibut Longline	Crab Pots	Salmon Fishery	Herring Fishery	Indigenous	Subsistence
Corals	1.67E-06	5.14E-05	9.40E-09	2.23E-06	4.01E-08	8.58E-05	1.33E-04	3.70E-09	3.10E-05	0	5.07E-07	0	2.00E-10	8.37E-07	0	0	0	0	0	0
Hydroids	5.07E-07	4.51E-07	0	5.25E-06	7.47E-08	2.41E-07	0	0	1.10E-08	0	7.20E-09	9.00E-10	0	2.44E-07	0	0	0	0	0	0
Urochordata	1.09E-05	9.27E-05	2.77E-07	2.33E-06	1.56E-06	4.84E-04	4.77E-05	5.89E-08	1.89E-06	0	2.65E-08	1.03E-08	5.40E-09	5.47E-06	0	0	0	0	0	0
Sea Pens	0	0	3.57E-08	7.55E-08	0	0	0	0	0	0	0	0	0	0	0	0	0	0	0	0
Sponges	0	0	0	0	0	0	0	0	0	0	0	0	0	0	0	0	0	0	0	0
Bivalves	0	0	0	0	0	0	0	0	0	0	0	0	0	0	0	0	0	0	0	0
Polychaetes	1.24E-02	1.17E-03	6.83E-06	1.83E-05	2.57E-05	7.65E-04	8.32E-04	1.06E-06	2.52E-05	0	8.43E-07	3.00E-09	0	7.22E-07	0	0	0	0	0	0
Misc. worms	0	0	0	0	0	0	0	0	0	0	0	0	0	0	0	0	0	0	0	0
Scyphozoid Jellies	0	0	0	0	0	0	0	0	0	0	0	0	0	0	0	0	0	0	0	0
Fish Larvae	0	0	0	0	0	0	0	0	0	0	0	0	0	0	0	0	0	0	0	0
Chaetognaths	0	0	0	0	0	0	0	0	0	0	0	0	0	0	0	0	0	0	0	0
Euphausiids	0	0	0	0	0	0	0	0	0	0	0	0	0	0	0	0	0	0	0	0
Mysids	0	0	0	0	0	0	0	0	0	0	0	0	0	0	0	0	0	0	0	0
Pelagic Amphipods	0	0	0	0	0	0	0	0	0	0	0	0	0	0	0	0	0	0	0	0
Gelatinous filter feeders	0	0	0	0	0	0	0	0	0	0	0	0	0	0	0	0	0	0	0	0
Pteropods	0	0	0	0	0	0	0	0	0	0	0	0	0	0	0	0	0	0	0	0
Copepods	0	0	0	0	0	0	0	0	0	0	0	0	0	0	0	0	0	0	0	0
Pelagic microbes	0	0	0	0	0	0	0	0	0	0	0	0	0	0	0	0	0	0	0	0
Benthic microbes	0	0	0	0	0	0	0	0	0	0	0	0	0	0	0	0	0	0	0	0
Macroalgae	0	0	0	0	0	0	0	0	0	0	0	0	0	0	0	0	0	0	0	0
Lg Phytoplankton	0	0	0	0	0	0	0	0	0	0	0	0	0	0	0	0	0	0	0	0
Sm Phytoplankton	0	0	0	0	0	0	0	0	0	0	0	0	0	0	0	0	0	0	0	0
Outside Production	0	0	0	0	0	0	0	0	0	0	0	0	0	0	0	0	0	0	0	0
Discards	0	0	0	0	0	0	0	0	0	0	0	0	0	0	0	0	0	0	0	0
Offal	0	0	0	0	0	0	0	0	0	0	0	0	0	0	0	0	0	0	0	0
Pelagic Detritus	0	0	0	0	0	0	0	0	0	0	0	0	0	0	0	0	0	0	0	0
Benthic Detritus	0	0	0	0	0	0	0	0	0	0	0	0	0	0	0	0	0	0	0	0
Outside Detritus	4E-10	0	0	0	0	0	0	0	0	0	0	0	0	0	0	0	0	0	0	0

Table S19: Ecopath base parameters for the Kerala Ecopath model

Group	Biomass (tkm^{-2})	Production / Biomass (year^{-1})	Consumption / Biomass (year^{-1})	Ecotrophic Efficiency
Marine Mammals	0.019	0.1	12.75	0
Sharks coastal	0.042	1.181	3.7	0.747
Sharks offshore	0.037	1.098	3.43	0.806
Sharks demersal	0.018	0.93	4.463	0.476
Guitarfishes & Rays	0.077	0.89	2.964	0.364
Large pelagics-offshore	0.342	1.053	2.698	0.663
Large Pelagics-inshore	0.205	1.206	5.091	0.989
Large Bentho Pelagics shelf	0.044	0.694	4.757	0.934
Large benthopelagic deep	0.62	0.563	2.254	0.98
Medium Bentho Pelagics shelf	1.519	1.425	8.904	0.996
Medium benthopelagic deep	0.707	1.012	6.467	0.95
Small Bentho Pelagics shelf	1.55	2.105	10.95	0.919
Small benthopelagic deep	3.5	1.713	13	0.998
Mesopelagics	1.2	1.68	11.602	0.762
Tunas coastal	0.44	1.552	13.6	0.673
Tunas offshore	0.193	1.172	17.603	0.743
Ribbonfishes	0.852	1.292	3.75	0.938
Large Benthic Carnivores shelf	1.667	0.778	5.324	0.993
Large Benthic Carnivores deep	0.145	0.623	4.32	0.95
Medium Benthic Carnivores shelf	1.687	1.386	7.697	0.792
Medium Benthic Carnivores deep	4.795	0.87	4.538	0.95
Small Benthic Carnivores shelf	2.95	2.17	11.272	0.968
Small Benthic Carnivores deep	8.812	0.925	4.484	0.95
Benthic omnivores shelf	0.288	0.94	26.98	0.98
Threadfin breams	1.364	2.046	10.938	1
Indian Mackerel	1.889	2.259	21.7	1
Oil Sardine	7.856	2.605	27.6	1
Other Clupeids inshore	1.5	2.884	20.7	0.999
Anchovies	8	2.39	17.133	0.994
Crabs & Lobsters	0.091	4.785	8.5	0.997
Deep Sea Shrimps	0.75	3.941	10.9	0.97
Coastal Shrimps	5.3	6.87	19.2	0.999
Squids	1.592	4.25	16.64	0.946
Cuttlefishes	0.59	3.552	16.64	0.998
Octopus	0.15	3.81	12.5	0.999
Commercial molluscs	0.44	4.301	12.5	0.996
Benthic detritivore	53.478	2.9	9	0.95
Macrozobenthos (Benthic carnivore)	15	3.1	12.482	0.991
Benthic grazers	30.558	0.73	2.5	0.95
Shelf filter feeders	5	1.6	12	0.719
Deep filter feeders	4	1.2	12	0.274
Gelatinous zooplankton	3.212	4.85	16.167	0.75
Large Zooplankton	6.538	35.3	160	0.75
Micro Zooplankton	14	67.5	250	0.818
Phytoplankton	58.5	70		0.953
Macroalgae	1554.967	2.78		0.95
Detritus	15			0.9

Table S20: Diet matrix for the Kerala Ecopath model

Group	Marine Mammals	Sharks coastal	Sharks offshore	Sharks demersal	Guitarfishes & Rays	Large pelagics-offshore	Large Pelagics-inshore	Large Bentho Pelagics shelf	Large benthopelagic deep	Medium Bentho Pelagics shelf	Medium benthopelagic deep	Small Bentho Pelagics shelf	Small benthopelagic deep	Mesopelagics	Tunas coastal	Tunas offshore	Ribbonfishes	Large Benthic Carnivores shelf	Large Benthic Carnivores deep	Medium Benthic Carnivores shelf	Medium Benthic Carnivores deep	Small Benthic Carnivores shelf	Small Benthic Carnivores deep	Benthic omnivores shelf	Threadfin breams	Indian Mackerel	Oil Sardine	Other Clupeids inshore	Anchovies	Crabs & Lobsters
Marine Mammals	0	0	0	0	0	0	0	0	0	0	0	0	0	0	0	0	0	0	0	0	0	0	0	0	0	0	0	0	0	0
Sharks coastal	0	0	0	0	0	0	0	0	0	0	0	0	0	0	0	0	0	0	0	0	0	0	0	0	0	0	0	0	0	0
Sharks offshore	0	0	0	0	0	0	0	0	0	0	0	0	0	0	0	0	0	0	0	0	0	0	0	0	0	0	0	0	0	0
Sharks demersal	0	0	0	0	0	0	0	0	0	0	0	0	0	0	0	0	0	0	0	0	0	0	0	0	0	0	0	0	0	0
Guitarfishes & Rays	0	0	0	0	0	0	0	0	0	0	0	0	0	0	0	0	0	0	0	0	0	0	0	0	0	0	0	0	0	0
Large pelagics-offshore	0	0	0	0	0	0	0.053	0	0	0.005	0	0	0	0	0	0	0	0	0	0	0	0	0	0	0	0	0	0	0	0
Large Pelagics-inshore	0	0.054	0	0	0	0	0	0	0	0	0	0	0	0	0	0	0	0	0	0	0	0	0	0	0	0	0	0	0.054	0
Large Bentho Pelagics shelf	0	0	0	0	0	0	0	0	0	0	0	0	0	0	0	0	0	0	0	0	0	0	0	0	0	0	0	0	0	0
Large benthopelagic deep	0	0	0	0	0	0.003	0	0	0	0	0	0	0	0	0	0.1	0	0	0	0	0	0	0	0	0	0	0	0	0	0
Medium Bentho Pelagics shelf	0	0.059	0.16	0	0	0.042	0.184	0	0	0.02	0	0	0	0	0	0	0.19	0.002	0	0	0	0	0	0	0	0	0	0	0.059	0.16
Medium benthopelagic deep	0	0	0	0	0	0	0	0	0	0	0	0	0	0	0	0.2	0	0	0	0	0	0	0	0	0	0	0	0	0	0
Small Bentho Pelagics shelf	0	0.359	0	0.005	0	0	0.11	0.078	0	0.111	0.009	0	0	0	0.018	0.013	0.053	0	0	0.003	0	0	0	0	0	0	0	0	0.359	0
Small benthopelagic deep	0	0	0.159	0.116	0.01	0.234	0	0	0	0	0.255	0	0.05	0	0.2	0	0	0	0	0	0.05	0	0	0	0	0	0	0	0	0.159
Mesopelagics	0	0	0	0	0	0	0	0	0	0.002	0.21	0	0	0.01	0.05	0	0	0	0.1	0	0.01	0	0	0	0	0	0	0	0	0
Tunas coastal	0	0	0	0	0	0	0.001	0	0	0.003	0	0	0	0	0	0	0	0	0	0.003	0	0.003	0	0	0	0	0	0	0	0
Tunas offshore	0	0	0.345	0	0	0	0	0	0	0	0	0	0	0	0	0	0	0	0	0	0	0	0	0	0	0	0	0	0	0.345
Ribbonfishes	0	0	0	0	0.001	0	0.059	0	0	0.007	0	0	0	0	0	0	0.055	0.02	0	0.007	0	0.001	0	0	0	0	0	0	0	0
Large Benthic Carnivores shelf	0.113	0	0	0	0.007	0.005	0.093	0.126	0	0.008	0	0	0	0	0.01	0	0.038	0.019	0	0.008	0	0	0	0	0.002	0	0	0.113	0	0
Large Benthic Carnivores deep	0	0	0	0	0	0	0	0	0	0	0	0	0	0	0	0	0	0	0.011	0	0	0	0	0	0	0	0	0	0	0
Medium Benthic Carnivores shelf	0	0.025	0	0	0	0.003	0.043	0	0	0.006	0	0	0	0	0	0	0	0.025	0.013	0.029	0	0	0	0	0.001	0	0	0	0.025	0
Medium Benthic Carniv. deep	0	0	0.095	0.55	0	0	0.026	0	0	0	0	0	0	0	0	0	0	0	0	0.003	0.01	0	0	0	0	0	0	0	0	0.095
Small Benthic Carnivores shelf	0	0	0.02	0	0.093	0.026	0.009	0	0	0.059	0	0	0	0	0.001	0	0	0.11	0.243	0.066	0	0.001	0	0	0.08	0	0	0	0	0.02
Small Benthic Carnivores deep	0	0	0	0	0	0	0	0	0	0	0.055	0	0.04	0	0	0	0	0	0	0.02	0.11	0	0	0	0	0	0	0	0	0
Benthic omnivores shelf	0	0.05	0	0.05	0.05	0	0	0	0	0	0	0	0	0	0	0	0	0.055	0	0	0	0	0	0	0	0	0	0	0.05	0

Group	Marine Mammals	Sharks coastal	Sharks offshore	Sharks demersal	Guitarfishes & Rays	Large pelagics-offshore	Large Pelagics-inshore	Large Bentho Pelagics shelf	Large benthopelagic deep	Medium Bentho Pelagics shelf	Medium benthopelagic deep	Small Bentho Pelagics shelf	Small benthopelagic deep	Mesopelagics	Tunas coastal	Tunas offshore	Ribbonfishes	Large Benthic Carnivores shelf	Large Benthic Carnivores deep	Medium Benthic Carnivores shelf	Medium Benthic Carnivores deep	Small Benthic Carnivores shelf	Small Benthic Carnivores deep	Benthic omnivores shelf	Threadfin breams	Indian Mackerel	Oil Sardine	Other Clupeids inshore	Anchovies	Crabs & Lobsters
Threadfin breams	0.116	0	0	0	0.325	0.048	0.097	0	0	0.04	0.015	0	0	0	0	0	0	0.032	0.096	0.03	0	0	0	0	0.006	0	0	0.116	0	0
Indian Mackerel	0.099	0	0	0	0	0.035	0.026	0.008	0	0.054	0	0	0	0	0	0.2	0	0	0	0.009	0	0	0	0	0	0	0	0.099	0	0
Oil Sardine	0.35	0	0	0	0	0.047	0.007	0.031	0	0.06	0	0	0	0	0.158	0.1	0.038	0.014	0	0.029	0	0	0	0	0	0	0	0.35	0	0
Other Clupeids inshore	0.015	0	0.042	0	0	0.007	0.027	0	0	0.029	0	0	0	0	0.006	0	0.012	0	0	0.001	0	0	0	0	0	0	0	0.015	0	0.042
Anchovies	0.012	0.096	0	0.098	0.013	0.434	0.04	0.633	0	0.206	0	0.061	0	0	0.203	0	0.38	0.166	0	0.038	0	0.07	0	0	0.02	0	0	0.012	0.096	0
Crabs & Lobsters	0.035	0.019	0	0	0.018	0.004	0.007	0	0	0.005	0	0	0	0	0.002	0	0	0.005	0	0.003	0	0.001	0	0	0	0	0	0.035	0.019	0
Deep Sea Shrimps	0.12	0	0	0	0.216	0.005	0.001	0.009	0	0	0.02	0	0	0	0.025	0	0	0.007	0.049	0.042	0.02	0	0.001	0	0	0	0	0.12	0	0
Coastal Shrimps	0.099	0.012	0	0	0.251	0.002	0.001	0.092	0	0.129	0	0.1	0	0	0.326	0	0.14	0.073	0.184	0.1	0.01	0.055	0.001	0	0.1	0	0	0.099	0.012	0
Squids	0.04	0	0.13	0.181	0	0.073	0.021	0.004	0.1	0.033	0.05	0.016	0.01	0.01	0	0.1	0.046	0.13	0.02	0.05	0.08	0	0	0	0.066	0	0	0.04	0	0.13
Cuttlefishes	0	0.017	0	0	0	0	0.002	0	0.1	0	0	0	0	0	0	0.06	0	0.045	0.02	0.05	0.011	0	0	0	0.01	0	0	0	0.017	0
Octopus	0	0.187	0.042	0	0	0.009	0	0	0	0.008	0	0	0	0	0	0	0	0	0.19	0.002	0.006	0	0	0	0.001	0	0	0	0.187	0.042
Commercial molluscs	0	0.107	0	0	0	0	0	0	0	0	0.001	0	0	0	0.001	0	0	0.001	0	0.014	0.001	0.003	0	0	0.02	0	0	0	0.107	0
Benthic detritivore	0	0	0	0	0	0	0.067	0	0.01	0.072	0.02	0.15	0.05	0	0	0.1	0.03	0	0	0.042	0.187	0.09	0.3	0.025	0.081	0	0	0	0	0
Macrozobenthos (Benthic carnivore)	0	0.015	0	0	0.016	0.02	0.126	0.007	0.065	0.07	0.31	0.523	0.1	0	0	0.125	0	0.096	0.075	0.029	0.216	0.11	0.4	0	0.33	0	0	0	0.015	0
Benthic grazers	0	0	0	0	0	0	0	0	0.025	0.067	0	0.05	0	0	0	0	0	0.14	0	0.11	0.053	0.048	0	0	0.19	0	0	0	0	0
Shelf filter feeders	0	0	0	0	0	0	0	0	0	0	0	0	0	0	0	0	0	0.06	0	0.046	0.073	0.052	0	0	0.02	0	0	0	0	0
Deep filter feeders	0	0	0	0	0	0	0	0	0	0	0	0	0	0	0	0	0	0	0	0	0	0	0	0	0	0	0	0	0	0
Gelatinous zooplankton	0	0	0	0	0	0	0	0	0.2	0	0.055	0	0.2	0.105	0	0	0	0	0	0	0	0	0	0	0.031	0	0	0	0	0
Large Zooplankton	0	0.001	0	0	0	0	0	0.001	0.5	0	0	0.1	0.55	0.81	0	0.002	0	0	0	0.016	0.163	0.04	0.299	0.2	0	0.187	0	0	0.001	0
Micro Zooplankton	0	0	0.004	0	0	0	0	0	0	0	0	0	0	0.055	0	0	0	0	0	0.145	0	0.179	0	0.25	0	0.196	0.204	0	0	0.004
Phytoplankton	0	0	0	0	0	0	0	0	0	0	0	0	0	0.01	0	0	0	0	0	0.002	0	0.028	0	0	0	0.511	0.796	0	0	0
Macroalgae	0	0	0	0	0	0	0	0	0	0	0	0	0	0	0	0	0	0	0	0	0	0	0	0.5	0	0	0	0	0	0
Detritus	0	0	0.004	0	0	0.005	0	0.012	0	0.002	0	0	0	0	0	0	0.018	0	0	0.103	0	0.32	0	0.025	0.042	0.107	0	0	0	0.004

Table S20: Diet matrix for the Kerala Ecopath model - continued

**Group**	Deep Sea Shrimps	Coastal Shrimps	Squids	Cuttlefishes	Octopus	Commercial molluscs	Benthic detritivore	Macrozobenthos (Benthic carnivore)	Benthic grazers	Shelf filter feeders	Deep filter feeders	Gelatinous zooplankton	Large Zooplankton	Micro Zooplankton
Marine Mammals	0	0	0	0	0	0	0	0	0	0	0	0	0	0
Sharks coastal	0	0	0	0	0	0	0	0	0	0	0	0	0	0
Sharks offshore	0	0	0	0	0	0	0	0	0	0	0	0	0	0
Sharks demersal	0	0	0	0	0	0	0	0	0	0	0	0	0	0
Guitarfishes & Rays	0	0	0	0	0	0	0	0	0	0	0	0	0	0
Large pelagics-offshore	0	0	0	0	0	0	0	0	0	0	0	0	0	0
Large Pelagics-inshore	0	0	0	0	0	0	0	0	0	0	0	0	0	0
Large Bentho Pelagics shelf	0	0	0	0	0	0	0	0	0	0	0	0	0	0
Large benthopelagic deep	0	0	0	0	0	0	0	0	0	0	0	0	0	0
Medium Bentho Pelagics shelf	0	0	0	0	0	0	0	0	0	0	0	0	0	0
Medium benthopelagic deep	0	0	0	0	0	0	0	0	0	0	0	0	0	0
Small Bentho Pelagics shelf	0	0	0	0.03	0.1	0	0	0	0	0	0	0	0	0
Small benthopelagic deep	0	0	0	0	0	0	0	0	0	0	0	0	0	0
Mesopelagics	0	0	0.01	0	0	0	0	0	0	0	0	0	0	0
Tunas coastal	0	0	0	0	0	0	0	0	0	0	0	0	0	0
Tunas offshore	0	0	0	0	0	0	0	0	0	0	0	0	0	0
Ribbonfishes	0	0	0	0	0	0	0	0	0	0	0	0	0	0
Large Benthic Carnivores shelf	0	0	0.002	0	0	0	0	0	0	0	0	0	0	0
Large Benthic Carnivores deep	0	0	0.001	0	0	0	0	0	0	0	0	0	0	0
Medium Benthic Carnivores shelf	0	0	0.001	0.03	0	0	0	0	0	0	0	0	0	0
Medium Benthic Carniv. deep	0	0	0.022	0.05	0	0	0	0	0	0	0	0	0	0
Small Benthic Carnivores shelf	0	0	0.001	0.06	0.09	0	0	0	0	0	0	0	0	0
Small Benthic Carnivores deep	0	0	0.001	0.15	0.01	0	0	0	0	0	0	0	0	0
Benthic omnivores shelf	0	0	0	0	0	0	0	0	0	0	0	0	0	0
Threadfin breams	0	0	0.001	0	0	0	0	0	0	0	0	0	0	0
Indian Mackerel	0	0	0.026	0	0	0	0	0	0	0	0	0	0	0
Oil Sardine	0	0	0.224	0.05	0	0	0	0	0	0	0	0	0	0
Other Clupeids inshore	0	0	0.055	0.013	0	0	0	0	0	0	0	0	0	0
Anchovies	0	0	0.03	0.1	0	0	0	0	0	0	0	0	0	0

Group	Deep Sea Shrimps	Coastal Shrimps	Squids	Cuttlefishes	Octopus	Commercial molluscs	Benthic detritivore	Macrozobenthos (Benthic carnivore)	Benthic grazers	Shelf filter feeders	Deep filter feeders	Gelatinous zooplankton	Large Zooplankton	Micro Zooplankton
Crabs & Lobsters	0	0	0	0.002	0.045	0	0	0	0	0	0	0	0	0
Deep Sea Shrimps	0	0	0.005	0	0.1	0	0	0	0	0	0	0	0	0
Coastal Shrimps	0	0	0.04	0.07	0.1	0	0	0	0	0	0	0	0	0
Squids	0	0.01	0.048	0.045	0	0	0	0	0	0	0	0	0	0
Cuttlefishes	0	0	0	0.1	0	0	0	0	0	0	0	0	0	0
Octopus	0	0	0	0.002	0	0	0	0	0	0	0	0	0	0
Commercial molluscs	0	0	0	0	0	0.13	0	0.001	0	0	0	0	0	0
Benthic detritivore	0.11	0.667	0.057	0.121	0.051	0	0.03	0.5	0	0	0	0	0	0
Macrozobenthos (Benthic carnivore)	0	0.094	0.122	0.026	0.051	0	0	0.035	0	0	0	0	0	0
Benthic grazers	0.052	0	0	0.001	0.041	0	0	0	0	0	0	0	0	0
Shelf filter feeders	0	0	0	0.025	0.011	0	0	0.001	0	0	0	0	0	0
Deep filter feeders	0.05	0	0	0.025	0	0	0	0.001	0	0	0	0	0	0
Gelatinous zooplankton	0	0	0.072	0	0	0	0	0	0	0	0	0	0	0
Large Zooplankton	0.417	0.019	0.207	0.1	0.401	0.08	0.08	0.245	0	0	0	0.25	0	0
Micro Zooplankton	0.248	0.016	0.076	0	0	0.06	0.13	0.16	0	0	0	0.25	0.58	0
Phytoplankton	0	0.004	0	0	0	0.12	0	0.007	0.12	0.8	0.8	0.5	0.22	1
Macroalgae	0	0	0	0	0	0	0.01	0	0.62	0	0	0	0	0
Detritus	0.123	0.19	0	0	0	0.61	0.75	0.05	0.26	0.2	0.2	0	0.2	0

Table S21: Landed catch for the Kerala Ecopath model (units $tkm^{-2}year^{-1}$). The Fleet names come from the Indian catch database codes. No discards are included in this model.

**Group**	MDTN	MGN	MHL	MOTHS	MPS	MRS	MTN	NM	OBBS	OBGN	OBHL	OBOTHS	OBRS	OBTN	OBSS
Marine Mammals	0	0	0	0	0	0	0	0	0	0	0	0	0	0	0
Sharks coastal	1.14E-03	1.85E-03	1.03E-03	1.32E-02	0	4.83E-08	1.08E-04	5.19E-05	4.56E-07	9.76E-04	6.14E-05	1.81E-04	7.40E-06	9.55E-08	0
Sharks offshore	1.01E-03	2.03E-03	9.48E-04	1.02E-02	0	4.97E-07	4.16E-05	3.87E-05	0	9.94E-04	3.52E-04	6.54E-04	4.57E-06	0	0
Sharks demersal	1.29E-03	3.46E-04	4.10E-04	4.89E-03	0	3.81E-06	1.15E-04	2.11E-06	5.35E-07	6.77E-04	1.29E-05	9.49E-05	0	1.55E-07	0
Guitarfishes & Rays	4.08E-03	1.65E-03	4.60E-04	9.35E-03	0	2.54E-04	1.04E-03	1.80E-04	2.12E-05	6.10E-03	1.34E-03	4.02E-04	5.76E-06	1.64E-05	0
Large pelagics-offshore	1.76E-03	5.70E-03	2.57E-03	2.81E-02	2.39E-06	1.03E-04	6.62E-05	6.62E-06	6.63E-05	1.10E-02	7.39E-03	1.29E-03	4.19E-04	4.66E-07	0
Large Pelagics-inshore	2.74E-02	2.02E-03	2.12E-03	1.75E-02	1.81E-04	4.27E-03	1.24E-03	1.08E-04	2.51E-03	4.25E-02	1.47E-02	1.43E-03	1.88E-03	5.14E-05	1.55E-06
Large Bentho Pelagics shelf	5.13E-03	3.83E-04	4.39E-04	2.79E-03	1.72E-04	1.52E-03	1.10E-04	5.69E-05	2.96E-04	1.38E-03	1.81E-03	4.46E-05	1.02E-04	1.71E-06	0
Large benthopelagic deep	2.58E-05	0	0	4.99E-07	0	0	0	0	0	3.74E-07	0	1.21E-06	0	0	0
Medium Bentho Pelagics shelf	2.62E-01	9.72E-04	3.87E-04	2.71E-02	5.42E-03	7.11E-02	8.30E-03	4.26E-03	3.24E-02	2.44E-02	1.31E-02	3.40E-04	4.31E-02	2.62E-04	2.48E-03
Medium benthopelagic deep	8.55E-05	8.51E-06	1.67E-06	1.56E-05	0	3.61E-06	1.12E-05	1.05E-07	1.99E-06	1.28E-05	5.19E-05	0	8.69E-05	0	0
Small Bentho Pelagics shelf	1.59E-02	2.14E-04	5.27E-06	1.25E-03	1.32E-04	6.00E-03	1.35E-03	4.36E-04	6.72E-03	2.75E-03	1.32E-03	6.49E-05	3.14E-02	2.85E-04	1.94E-06
Small benthopelagic deep	9.12E-05	0	6.11E-07	4.22E-07	0	0	0	4.55E-07	0	0	0	0	0	0	0
Mesopelagics	6.73E-06	0	0	0	0	0	0	0	0	0	0	0	0	0	0
Tunas coastal	2.03E-03	2.63E-03	1.90E-03	1.32E-02	9.88E-04	1.15E-02	2.58E-05	5.92E-05	1.48E-03	5.90E-02	4.92E-02	1.10E-03	8.50E-04	6.29E-05	3.13E-04
Tunas offshore	1.01E-03	7.38E-03	2.45E-03	3.51E-02	1.62E-05	1.61E-04	8.74E-06	0	0	9.62E-03	4.90E-03	1.45E-03	5.39E-05	0	0
Ribbonfishes	1.46E-01	3.81E-04	4.21E-03	1.99E-02	1.70E-06	6.60E-04	5.80E-03	4.77E-05	5.25E-03	1.01E-02	2.14E-03	1.94E-04	4.78E-04	2.07E-04	2.68E-04
Large Benthic Carnivores shelf	1.51E-01	1.93E-03	1.76E-03	1.78E-02	8.64E-05	2.83E-03	5.62E-03	1.07E-03	2.57E-03	1.81E-02	5.30E-03	9.24E-05	4.91E-03	7.30E-05	2.81E-04
Large Benthic Carnivores deep	3.57E-03	2.39E-04	9.60E-04	4.86E-03	1.84E-08	1.43E-07	2.03E-04	5.72E-06	1.70E-05	1.84E-03	1.34E-03	3.98E-05	2.57E-07	5.75E-07	0
Medium Benthic Carnivores shelf	1.25E-01	1.03E-03	2.62E-04	6.56E-03	1.62E-03	9.05E-03	1.51E-02	5.46E-03	1.19E-02	1.81E-02	3.99E-03	8.92E-05	2.18E-02	2.39E-03	0
Medium Benthic Carniv. deep	1.29E-03	4.66E-07	1.65E-06	9.12E-05	0	0	1.00E-05	0	0	6.17E-06	4.06E-06	6.57E-06	0	0	0
Small Benthic Carnivores shelf	9.10E-02	4.13E-06	5.13E-06	3.58E-03	1.60E-05	6.82E-03	7.74E-02	1.17E-02	4.71E-03	1.70E-02	1.57E-04	2.29E-06	1.64E-02	8.59E-03	5.06E-04
Small Benthic Carnivores deep	8.17E-04	1.17E-05	1.83E-06	3.21E-05	0	3.73E-07	1.99E-07	0	0	2.23E-05	0	0	0	0	0
Benthic omnivores shelf	1.74E-04	9.82E-06	1.15E-05	1.53E-05	0	2.28E-04	9.00E-05	3.05E-04	3.33E-05	2.21E-04	2.42E-05	0	3.53E-04	5.94E-07	0
Threadfin breams	4.28E-01	1.02E-05	4.09E-06	2.99E-02	0	9.08E-05	8.11E-03	1.10E-04	1.14E-03	3.01E-03	1.65E-03	5.67E-07	4.33E-05	9.95E-06	0
Indian Mackerel	9.37E-02	6.34E-05	5.98E-08	1.25E-02	2.26E-02	2.26E-01	3.93E-03	6.17E-03	6.86E-03	1.54E-01	8.02E-03	2.10E-04	9.14E-02	5.71E-05	8.39E-04
Oil Sardine	2.30E-02	7.30E-06	0	3.67E-03	6.49E-03	1.18E+00	1.55E-02	3.09E-02	6.11E-03	5.59E-02	0	1.75E-06	1.00E+00	7.20E-05	0
Other Clupeids inshore	3.33E-02	2.38E-04	0	1.21E-03	2.38E-04	8.35E-02	1.23E-02	2.47E-02	2.88E-02	3.39E-02	2.28E-05	5.73E-06	9.11E-02	1.47E-03	1.52E-03
Anchovies	5.02E-02	5.24E-06	0	1.16E-03	8.98E-08	7.56E-02	8.16E-03	4.79E-03	2.09E-02	9.16E-04	0	1.80E-06	2.79E-01	1.11E-03	2.65E-03
Crabs & Lobsters	2.22E-02	0	0	3.35E-04	2.64E-07	3.47E-06	1.19E-02	2.43E-03	1.97E-04	1.56E-03	0	7.05E-06	8.47E-05	2.57E-03	0
Deep Sea Shrimps	9.39E-02	0	0	1.43E-03	0	0	1.74E-04	0	0	0	0	0	0	2.39E-05	0
Coastal Shrimps	1.78E-01	0	0	1.13E-03	3.39E-05	3.01E-02	9.19E-02	3.51E-03	5.04E-03	1.56E-03	0	6.58E-06	4.94E-02	3.28E-02	0
Squids	1.47E-01	6.18E-05	1.60E-03	1.11E-02	1.38E-05	8.65E-04	8.20E-03	6.86E-04	1.73E-02	1.53E-03	7.57E-03	6.11E-05	1.09E-03	1.54E-03	1.42E-04
Cuttlefishes	1.44E-01	6.12E-05	5.43E-03	1.59E-02	5.14E-07	2.65E-05	2.46E-03	1.27E-04	1.37E-05	2.54E-05	3.69E-02	3.42E-05	1.62E-03	8.59E-04	0
Octopus	4.21E-02	1.14E-05	1.04E-06	1.77E-03	0	0	1.04E-03	3.15E-06	2.59E-07	1.78E-07	2.57E-05	0	1.44E-07	0	0
Commercial molluscs	2.53E-03	0	0	2.53E-08	0	0	5.71E-03	1.21E-04	0	1.85E-04	0	6.05E-06	0	0	0
Benthic detritivore	0	0	0	0	0	0	0	0	0	0	0	0	0	0	0
Macrozobenthos (Benthic carnivore)	3.21E-03	0	0	2.30E-08	1.86E-07	6.01E-07	1.82E-02	3.22E-07	0	4.03E-07	0	0	4.73E-05	3.97E-03	0
Benthic grazers	1.49E-06	0	0	0	0	0	4.35E-07	0	0	0	0	0	0	0	0
Shelf filter feeders	0	0	0	0	0	0	0	0	0	0	0	0	0	0	0

**Group**	MDTN	MGN	MHL	MOTHS	MPS	MRS	MTN	NM	OBBS	OBGN	OBHL	OBOTHS	OBRS	OBTN	OBSS
Deep filter feeders	0	0	0	0	0	0	0	0	0	0	0	0	0	0	0
Gelatinous zooplankton	0	0	0	0	0	0	0	0	0	0	0	0	0	0	0
Large Zooplankton	0	0	0	0	0	3.31E-05	4.78E-05	6.42E-06	0	0	0	0	1.93E-05	6.31E-06	0
Micro Zooplankton	0	0	0	0	0	0	0	0	0	0	0	0	0	0	0
Phytoplankton	0	0	0	0	0	0	0	0	0	0	0	0	0	0	0
Macroalgae	0	0	0	0	0	0	0	0	0	0	0	0	0	0	0
Detritus	0	0	0	0	0	0	0	0	0	0	0	0	0	0	0

Table S22: Ecopath base parameters for the north central Chilean Ecopath model

Group	Biomass (tkm^{-2})	Production / Biomass ($year^{-1}$)	Consumption / Biomass ($year^{-1}$)	Ecotrophic Efficiency
Phytoplankton	302	120		0.594
Zooplankton	45.229	242.5	956	0.99
Euphausiids	112.295	13	31.707	0.99
Small fish	43.557	2.88	14.2	0.95
Anchovy	12.6	4.799	12.04	0.95
Sardine	1.046	2.14	14.2	0.81
Horse mackerel	20.441	0.53	10.8	0.296
Jack mackerel	99.459	0.17	14.2	0.189
Hake	10.092	1.55	5.159	0.749
Sea lions	0.144	0.25	20	0
Marine birds	0.065	0.5	20	0
Jumbo squid	7.45	3	8.571	0.535
Demersal crustaceans	4.443	4	14.104	0.958
Sword fish	0.019	0.5	5	0.95
Sharks	0.008	0.362	2.413	0.95
Detritus	1			0.614

Table S23: Diet matrix for the north central Chilean Ecopath model

Group	Zooplankton	Euphausiids	Small fish	Anchovy	Sardine	Horse mackerel	Jack mackerel	Hake	Sea lions	Marine birds	Jumbo squid	Demersal crustaceans	Sword fish	Sharks
Phytoplankton	0.45	0.45	0.6	0.6	0.05	0	0	0	0	0	0	0	0	0
Zooplankton	0.2	0.55	0.1	0.1	0.535	0.63	0.02	0	0	0	0	0	0	0
Euphausiids	0	0	0.14	0.14	0.415	0.26	0.9	0.053	0	0	0	0	0	0
Small fish	0	0	0	0	0	0.028	0.08	0	0	0	0	0	0	0.22
Anchovy	0	0	0.015	0.015	0	0.082	0	0.145	0.155	0.682	0.198	0	0	0
Sardine	0	0	0	0	0	0	0	0.015	0.015	0.076	0.014	0	0	0
Horse mackerel	0	0	0	0	0	0	0	0.009	0.06	0	0.009	0	0	0.29
Jack mackerel	0	0	0	0	0	0	0	0.009	0.06	0	0.009	0	0	0
Hake	0	0	0	0	0	0	0	0.085	0.18	0.242	0.1	0	0.45	0
Sea lions	0	0	0	0	0	0	0	0	0	0	0	0	0	0
Marine birds	0	0	0	0	0	0	0	0	0	0	0	0	0	0
Jumbo squid	0	0	0	0	0	0	0	0	0	0	0.15	0	0	0.305
Demersal crustaceans	0	0	0	0	0	0	0	0.23	0.08	0	0.068	0	0.23	0.1
Sword fish	0	0	0	0	0	0	0	0	0	0	0	0	0	0.025
Sharks	0	0	0	0	0	0	0	0	0	0	0	0	0	0
Detritus	0.35	0	0	0	0	0	0	0	0	0	0	1	0	0
Import	0	0	0.145	0.145	0	0	0	0.456	0.45	0	0.453	0	0.32	0.06

Table S24: Landed catch for the north central Chilean Ecopath model (units $tkm^{-2}year^{-1}$). No discards are included in this model.

**Group**	Purse seine	Trawler	Longline	Small longline	Squider
Phytoplankton	0	0	0	0	0
Zooplankton	0	0	0	0	0
Euphausiids	0	0	0	0	0
Small fish	0	0	0	0	0
Anchovy	6.26E+00	0	0	0	0
Sardine	1.05E-02	0	0	0	0
Horse mackerel	2.04E+00	0	0	0	0
Jack mackerel	2.04E+00	0	0	0	0
Hake	0	0	0	2.13E-02	0
Sea lions	0	0	0	0	0
Marine birds	0	0	0	0	0
Jumbo squid	0	0	0	0	2.37E+00
Demersal crustaceans	0	4.55E-01	0	0	0
Sword fish	0	0	8.75E-03	0	0
Sharks	0	0	2.66E-03	0	0
Detritus	0	0	0	0	0
Import	0	0	0	0	0

Table S25**:** Ecopath base parameters for the SE Australian Ecopath model (showing both biomass variants, other base parameters being identical across the model variants).

**Group**	**Biomass (tkm^{-2}) Variant 1**	**Biomass (tkm^{-2}) Variant 2**	**Production / Biomass ($year^{-1}$)**	**Consumption / Biomass ($year^{-1}$)**	**Ecotrophic Efficiency**	**Biomass Accumulation (tkm^{-2})**	**Biomass Accumulation $year^{-1}$)**
Toothed whale	0.013	0.013	0.02	13	0.634	0	0
Baleen whale	0.006	0.006	0.02	11.2	0	0	0
Seal	0.051	0.043	0.18	38.898	0.724	0.004	0.105
Seabirds	0.003	0.003	0.8	80	0.363	0	0
Penguins	0.001	0.001	0.8	80	0.692	0	0
Tuna/billfish	1.131	0.077	0.48	6.8	0.4	0	0
Pelagic sharks	0.004	0.004	0.2	1.2	0.95	0	0
Demersal sharks	1.215	1.231	0.18	1.8	0.806	0	0
Rays	1.200	1.220	0.35	3.5	0.661	0	0
Warehous	0.900	0.962	0.28	2.4	0.787	0	0
Redbait	2.200	0.917	0.49	4.1	0.974	0	0
Redfish	1.070	1.162	0.31	3.4	0.646	0	0
Ling	0.440	0.470	0.22	2.4	0.767	0	0
Dories	0.390	0.405	0.3	2.8	0.826	0	0
Jack mackerel	6.000	6.064	0.47	3.3	0.647	0	0
Jackass morwong	0.628	0.659	0.22	2.9	0.975	0	0
Flathead	0.434	0.432	0.52	3.5	0.803	0	0
Gemfish	0.220	0.136	0.44	2.1	0.893	0	0
ShOceanPerch	0.274	0.192	0.26	2.6	0.995	0	0
Chinaman leatherjacket	0.011	0.005	0.36	2.3	0.565	0	0
Cucumberfish	2.490	2.669	0.52	4.7	0.987	0	0
Whiting	1.691	1.301	0.9	5.4	0.799	0	0
Cardinal	4.088	5.504	0.77	6.4	0.95	0	0
ShSmInvertFeeder	5.210	4.735	0.65	4.67	1	0	0
ShSmPredator	0.800	0.553	0.55	4.46	0.999	0	0
ShMedInvertFeeder	1.350	1.189	0.36	3.4	0.957	0	0
ShMedPredator	0.510	0.437	0.4	2.93	0.996	0	0
ShLInvertFeeder	0.120	0.090	0.21	2	0.437	0	0
ShLPredator	3.000	1.149	0.22	1.84	0.51	0	0
Blue-eye trevalla	0.350	0.052	0.2	1.4	0.557	0	0
Blue grenadier	0.820	0.160	0.27	2.9	0.973	0	0
SlopeOceanPerch	0.180	0.101	0.26	3.1	0.935	0	0
Deepsea Cod	0.470	0.065	0.25	2.2	0.294	0	0
Oreos	0.084	0.013	0.35	2.7	0.655	0	0
SlopeSmInvertFeeder	0.200	0.111	0.47	4.13	0.985	0	0
SlopeSmPredator	0.453	0.273	0.4	3.24	0.743	0	0
SlopeMInverFeeder	3.300	2.033	0.29	3.5	0.616	0	0
SlopeMPredator	0.300	0.179	0.305	2.5	0.961	0	0
SlopeLInvertFeeder	1.229	0.610	0.44	2.9	0.95	0	0
SlopeLPredator	0.120	0.077	0.2	2.34	0.512	0	0
PelSmInvertFeeder	6.193	19.599	0.76	8.85	0.98	0	0
PelMInvertFeeder	0.131	0.139	0.46	3.4	0.88	0	0
PelMPredator	0.320	0.358	0.32	2.85	0.974	0	0
PelLInvertFeeder	0.039	0.040	0.12	2.6	0.935	0	0
PelLPredator	0.009	0.003	0.26	3.1	0.95	0	0
Mesopelagics	200	61.044	0.83	8	0.871	0	0
Squid	1.640	2.271	2.75	10	0.996	0	0
PelagicPrawns	3.024	2.095	1.6	10	0.95	0	0

Group	Biomass (tkm^{-2}) Variant 1	Biomass (tkm^{-2}) Variant 2	Production / Biomass ($year^{-1}$)	Consumption / Biomass ($year^{-1}$)	Ecotrophic Efficiency	Biomass Accumulation (tkm^{-2})	Biomass Accumulation $year^{-1}$)
Macrobenthos	26.319	30.922	1.6	6	0.95	0	0
Megabenthos	7.203	8.995	2.5	5.85	0.95	0	0
Polychaeta	5.749	5.921	2	22	0.95	0	0
Gelatinous nekton	2.575	1.089	3	10	0.95	0	0
Euphausids	122.163	123.401	5	32	0.95	0	0
L zooplankton	10.938	40.956	5	32	0.95	0	0
Sm zooplankton	32.601	76.907	20	70	0.95	0	0
Primary producers	15.000	28.881	298		0.634	0	0
Benthic producer	0.006	0.008	4.43		0	0	0
Detritus	200	200			0.724	0.004	0.105
Discards	0.170	0.831			0.363	0	0

Table S26**:** Diet matrix for the SE Australian Ecopath model – Variant 1

**Group**	Toothed whale	Baleen whale	Seal	Seabirds	Penguins	Tuna/billfish	Pelagic sharks	Demersal sharks	Rays	Warehous	Redbait	Redfish	Ling	Dories	Jack mackerel	Jackass morwong	Flathead	Gemfish	ShOceanPerch	Chinaman leatherjacket	Cucumberfish	Whiting	Cardinal	ShSmInvertFeeder	ShSmPredator	ShMedInvertFeeder	ShMedPredator	ShLInvertFeeder	ShLPredator	Blue-eye trevalla
Toothed whale	0.001	0	0	0	0	0	0	0	0	0	0	0	0	0	0	0	0	0	0	0	0	0	0	0	0	0	0	0	0	0
Baleen whale	0	0	0	0	0	0	0	0	0	0	0	0	0	0	0	0	0	0	0	0	0	0	0	0	0	0	0	0	0	0
Seal	0.002	0	0	0	0	0	0.025	0	0	0	0	0	0	0	0	0	0	0	0	0	0	0	0	0	0	0	0	0	0	0
Seabirds	0.005	0	0	0	0	0	0.009	0	0	0	0	0	0	0	0	0	0	0	0	0	0	0	0	0	0	0	0	0	0	0
Penguins	0.003	0	0	0	0	0	0.009	0	0	0	0	0	0	0	0	0	0	0	0	0	0	0	0	0	0	0	0	0	0	0
Tuna/billfish	0.002	0	0	0	0	0	0.025	0	0	0	0	0	0	0	0	0	0	0	0	0	0	0	0	0	0	0	0	0	0	0
Pelagic sharks	0.002	0	0	0	0	0	0.006	0	0	0	0	0	0	0	0	0	0	0	0	0	0	0	0	0	0	0	0	0	0	0
Demersal sharks	0	0	0	0	0	0	0.134	0	0	0	0	0	0	0	0	0	0	0	0	0	0	0	0	0	0	0	0.01	0	0.03	0
Rays	0	0	0	0	0	0	0.134	0.006	0	0	0	0	0	0	0	0	0	0	0	0	0	0	0	0	0	0	0.01	0	0.05	0
Warehous	0	0	0.045	0	0.1	0	0.048	0	0	0	0	0	0	0	0	0	0	0	0	0	0	0	0	0	0	0	0	0	0.01	0
Redbait	0	0.01	0.251	0.01	0	0.159	0	0.008	0	0	0	0	0	0.096	0	0	0	0	0	0	0	0	0	0	0	0	0.05	0	0.017	0
Redfish	0	0	0.024	0	0	0	0.009	0.023	0	0	0	0	0	0.039	0	0	0	0	0	0	0	0	0	0	0	0	0	0	0.002	0
Ling	0	0	0.012	0	0	0	0.001	0.001	0	0	0	0	0	0	0	0	0.014	0	0	0	0	0	0	0	0	0	0	0	0	0
Dories	0	0	0.01	0	0	0	0.008	0.001	0	0	0	0	0	0	0	0	0.01	0.05	0	0	0	0	0	0	0	0	0.03	0	0	0
Jack mackerel	0.078	0	0.3	0	0	0.228	0.045	0.21	0	0	0	0	0	0.245	0	0	0.005	0.249	0	0	0	0	0	0	0	0	0.06	0	0.032	0
Jackass morwong	0	0	0.016	0	0	0	0	0.011	0	0	0	0	0	0	0	0	0	0	0	0	0	0	0	0	0	0	0.02	0	0.009	0
Flathead	0	0	0.035	0	0	0	0	0.007	0	0	0	0	0	0	0	0.001	0	0	0	0	0	0	0	0	0	0	0.01	0	0	0
Gemfish	0	0	0.015	0	0	0	0.008	0	0	0	0	0	0	0	0	0	0	0	0	0	0	0	0	0	0	0	0	0	0	0
ShOceanPerch	0	0	0.005	0	0	0	0	0.001	0	0	0	0	0	0	0	0	0.002	0	0	0	0	0	0	0	0	0	0	0	0.002	0
Chinaman leatherjacket	0	0	0	0	0	0	0	0	0	0	0	0	0	0	0	0	0	0	0	0	0	0	0	0	0	0	0	0	0	0
Cucumberfish	0	0	0	0	0	0	0	0.007	0	0	0	0.01	0.08	0.152	0	0.002	0.128	0.05	0.08	0	0	0	0	0	0.1	0.04	0.04	0	0.04	0
Whiting	0	0	0.01	0	0	0	0	0.003	0.02	0	0	0	0	0.006	0	0	0.1	0	0	0	0	0	0	0	0.1	0	0.002	0	0.083	0
Cardinal	0	0	0.008	0	0	0	0	0.037	0	0.018	0	0.253	0.192	0.16	0.006	0	0.05	0.071	0.074	0	0	0	0	0	0.101	0	0.025	0	0.181	0
ShSmInvertFeeder	0	0	0.092	0	0	0	0	0.03	0.022	0	0	0.01	0.477	0.224	0.001	0.007	0.085	0	0.083	0.1	0.01	0.005	0	0.002	0.151	0.064	0.251	0	0.014	0
ShSmPredator	0	0	0.051	0	0	0	0	0	0	0	0	0.01	0	0	0	0	0	0	0	0.1	0.005	0	0	0.001	0	0	0.012	0	0	0
ShMedInvertFeeder	0	0	0	0.005	0	0	0	0.013	0	0	0	0.001	0	0.019	0	0.004	0.025	0	0.082	0	0.001	0.005	0	0.001	0.005	0.001	0.025	0	0.01	0
ShMedPredator	0	0	0.04	0.005	0	0	0	0.005	0	0	0	0	0	0.017	0	0.002	0.005	0	0	0	0	0	0	0	0	0	0	0	0	0
ShLInvertFeeder	0	0	0	0	0.1	0	0	0	0	0	0	0	0	0	0	0	0	0	0	0	0	0	0	0	0	0	0	0	0	0
ShLPredator	0	0	0	0	0	0	0	0.008	0	0	0	0	0	0	0	0	0.01	0.169	0.007	0	0	0	0	0	0	0	0.02	0	0	0
Blue-eye trevalla	0.01	0	0	0	0	0	0	0.001	0	0	0	0	0	0	0	0	0	0	0	0	0	0	0	0	0	0	0	0	0	0
Blue grenadier	0	0	0	0	0	0	0	0	0	0	0	0	0	0	0	0	0	0.06	0	0	0	0	0	0	0	0	0	0	0	0.01
SlopeOceanPerch	0	0	0	0	0	0	0	0	0	0	0	0.001	0	0	0	0	0	0	0	0	0	0	0	0	0	0	0	0	0.002	0
Deepsea Cod	0	0	0	0	0	0	0	0	0	0	0	0	0	0	0	0	0	0	0	0	0	0	0	0	0	0	0	0	0	0
Oreos	0	0	0	0	0	0	0	0	0	0	0	0	0	0	0	0	0	0	0	0	0	0	0	0	0	0	0	0	0	0
SlopeSmInvertFeeder	0	0.02	0	0	0	0	0	0	0.001	0	0	0	0	0	0	0	0	0.016	0	0.1	0	0	0	0	0	0	0	0	0	0.01

Group	Toothed whale	Baleen whale	Seal	Seabirds	Penguins	Tuna/billfish	Pelagic sharks	Demersal sharks	Rays	Warehous	Redbait	Redfish	Ling	Dories	Jack mackerel	Jackass morwong	Flathead	Gemfish	ShOceanPerch	Chinaman leatherjacket	Cucumberfish	Whiting	Cardinal	ShSmInvertFeeder	ShSmPredator	ShMedInvertFeeder	ShMedPredator	ShLInvertFeeder	ShLPredator	Blue-eye trevalla
SlopeSmPredator	0	0.01	0	0	0	0	0	0.006	0.004	0	0	0.001	0.01	0	0	0	0	0	0.014	0.1	0.001	0	0	0	0	0	0	0	0	0.01
SlopeMInverFeeder	0	0	0	0	0	0	0	0.022	0.01	0	0	0.004	0	0	0	0.002	0	0.111	0	0	0	0	0	0	0	0	0	0	0	0.01
SlopeMPredator	0	0	0	0	0.1	0	0	0.006	0	0	0	0	0	0	0	0	0	0	0	0	0	0	0	0	0	0	0	0	0	0
SlopeLInvertFeeder	0	0	0	0	0	0	0	0.005	0	0	0	0.014	0.056	0	0	0	0.105	0	0.068	0	0	0	0	0	0.005	0	0	0	0	0
SlopeLPredator	0	0	0	0	0	0	0	0	0	0	0	0	0	0	0	0	0	0	0	0	0	0	0	0	0	0	0	0	0	0
PelSmInvertFeeder	0.098	0.04	0	0.005	0.45	0.009	0	0.001	0	0	0	0	0	0	0	0	0.24	0	0	0	0	0	0	0	0	0	0	0	0.28	0
PelMInvertFeeder	0.049	0	0	0	0	0	0.01	0.005	0	0	0	0	0	0	0	0	0	0	0	0	0	0	0	0	0	0	0	0	0	0
PelMPredator	0.01	0	0	0	0	0.02	0.03	0.01	0	0	0	0	0	0	0	0	0	0	0	0	0	0	0	0	0	0	0	0	0	0
PelLInvertFeeder	0.025	0	0	0	0	0	0.045	0	0	0	0	0	0	0	0	0	0	0	0	0	0	0	0	0	0	0	0	0	0	0
PelLPredator	0	0	0	0	0	0	0.039	0	0	0	0	0	0	0	0	0	0	0	0	0	0	0	0	0	0	0	0	0	0	0
Mesopelagics	0.098	0.02	0	0.135	0	0.005	0	0	0	0.002	0.065	0.068	0	0	0.313	0.001	0.038	0.18	0	0	0	0	0.837	0	0	0	0	0	0.12	0.05
Squid	0.149	0	0.055	0.27	0.1	0.07	0.1	0.222	0	0.046	0	0.049	0	0.007	0	0.065	0	0.044	0.021	0	0.018	0.007	0	0.002	0	0.003	0.1	0	0.025	0.2
PelagicPrawns	0	0	0	0	0.05	0	0	0.029	0.084	0	0	0.025	0.011	0.012	0	0	0	0	0.014	0	0.006	0	0.01	0.027	0	0	0.05	0	0.075	0.01
Macrobenthos	0	0	0	0.025	0	0	0.045	0.005	0.114	0.021	0	0	0	0.001	0.009	0.004	0.006	0	0.029	0.1	0.139	0.48	0	0.215	0.1	0.1	0.05	0.36	0	0
Megabenthos	0	0	0	0	0.05	0	0.018	0.257	0.342	0.005	0	0.053	0.103	0.008	0.011	0.109	0.05	0	0.096	0.1	0.117	0.012	0.013	0.197	0.1	0.575	0.151	0.28	0.008	0
Polychaeta	0	0	0	0	0	0	0	0.003	0.209	0	0	0.001	0	0	0	0.124	0.002	0	0.007	0	0.05	0.464	0	0.144	0	0.05	0	0.21	0	0
Gelatinous nekton	0	0	0	0.005	0	0	0	0.001	0	0.901	0.412	0.001	0	0	0.011	0	0.024	0	0.347	0.4	0.073	0	0	0.003	0.056	0	0	0	0.001	0.7
Euphausids	0	0.1	0	0	0.05	0.01	0	0	0	0.005	0.15	0.09	0.03	0.005	0.094	0.425	0.001	0	0	0	0.285	0.016	0.052	0.016	0.005	0	0	0	0.001	0
L zooplankton	0	0	0	0	0	0	0	0.002	0.058	0	0.116	0.284	0.036	0.006	0.365	0.003	0.049	0	0.04	0	0.212	0.005	0.084	0.102	0.194	0.153	0.08	0.15	0.004	0
Sm zooplankton	0	0	0	0	0	0	0	0.026	0.126	0.002	0.256	0.126	0.005	0.003	0.19	0.252	0.05	0	0.038	0	0.084	0.006	0.004	0.29	0.082	0.011	0	0	0.004	0
Primary producers	0	0	0	0	0	0	0	0	0	0	0	0	0	0	0	0	0	0	0	0	0	0	0	0	0	0	0	0	0	0
Benthic producer	0	0	0	0	0	0	0	0	0	0	0	0	0	0	0	0	0	0	0	0	0	0	0	0	0	0.003	0	0	0	0
Detritus	0	0	0	0	0	0	0	0	0	0	0	0	0	0	0	0	0	0	0	0	0	0	0	0	0	0	0	0	0	0
Discards	0	0	0.03	0.04	0	0	0	0.028	0.01	0	0	0	0	0	0	0	0	0	0	0	0	0	0	0	0	0	0.003	0	0	0
Imports	0.47	0.8	0	0.5	0	0.5	0.25	0	0	0	0	0	0	0	0	0	0	0	0	0	0	0	0	0	0	0	0	0	0	0

Table S26**:** Diet matrix for the SE Australian Ecopath model - continued

Group	Blue grenadier	SlopeOceanPerch	Deepsea Cod	Oreos	SlopeSmInvertFeeder	SlopeSmPredator	SlopeMInverFeeder	SlopeMPredator	SlopeLInvertFeeder	SlopeLPredator	PelSmInvertFeeder	PelMInvertFeeder	PelMPredator	PelLInvertFeeder	PelLPredator	Mesopelagics	Squid	PelagicPrawns	Macrobenthos	Megabenthos	Polychaeta	Gelatinous nekton	Euphausids	L zooplankton	Sm zooplankton
Toothed whale	0	0	0	0	0	0	0	0	0	0	0	0	0	0	0	0	0	0	0	0	0	0	0	0	0
Baleen whale	0	0	0	0	0	0	0	0	0	0	0	0	0	0	0	0	0	0	0	0	0	0	0	0	0
Seal	0	0	0	0	0	0	0	0	0	0	0	0	0	0	0	0	0	0	0	0	0	0	0	0	0
Seabirds	0	0	0	0	0	0	0	0	0	0	0	0	0	0	0	0	0	0	0	0	0	0	0	0	0
Penguins	0	0	0	0	0	0	0	0	0	0	0	0	0	0	0	0	0	0	0	0	0	0	0	0	0
Tuna/billfish	0	0	0	0	0	0	0	0	0	0	0	0	0	0	0	0	0	0	0	0	0	0	0	0	0
Pelagic sharks	0	0	0	0	0	0	0	0	0	0	0	0	0	0	0	0	0	0	0	0	0	0	0	0	0
Demersal sharks	0	0	0	0	0	0	0	0	0	0	0	0	0	0	0	0	0	0	0	0	0	0	0	0	0
Rays	0	0	0	0	0	0	0	0	0	0.01	0	0	0	0	0	0	0	0	0	0	0	0	0	0	0
Warehous	0	0	0	0	0	0	0	0	0	0.01	0	0	0	0	0	0	0	0	0	0	0	0	0	0	0
Redbait	0	0	0	0	0	0	0	0	0	0	0	0	0.05	0	0	0	0.01	0	0	0	0	0	0	0	0
Redfish	0	0	0	0	0	0	0	0	0	0	0	0	0	0	0	0	0.002	0	0	0	0	0	0	0	0
Ling	0	0	0	0	0	0	0	0	0	0	0	0	0	0	0	0	0.001	0	0	0	0	0	0	0	0
Dories	0	0	0	0	0	0	0	0	0	0	0	0	0	0	0	0	0	0	0	0	0	0	0	0	0
Jack mackerel	0	0.06	0	0	0	0	0	0	0	0	0	0	0	0	0	0	0.001	0	0	0	0	0	0	0	0
Jackass morwong	0	0	0	0	0	0	0	0	0	0	0	0	0	0	0	0	0	0	0	0	0	0	0	0	0
Flathead	0	0	0	0	0	0	0	0	0	0	0	0	0	0	0	0	0	0	0	0	0	0	0	0	0
Gemfish	0.016	0.059	0	0	0	0	0	0	0	0	0	0	0	0	0	0	0	0	0	0	0	0	0	0	0
ShOceanPerch	0	0	0	0	0	0	0	0	0	0	0	0	0	0	0	0	0.001	0	0	0	0	0	0	0	0
Chinaman leatherjacket	0	0	0	0	0	0	0	0	0	0	0	0	0	0	0	0	0	0	0	0	0	0	0	0	0
Cucumberfish	0	0.009	0	0	0	0	0	0.094	0	0	0	0	0	0	0	0	0	0	0	0	0	0	0	0	0
Whiting	0	0	0	0	0	0	0	0	0	0	0	0	0	0	0	0	0.001	0	0	0	0	0	0	0	0
Cardinal	0.015	0.001	0	0	0	0	0	0.249	0	0	0	0	0	0	0	0	0	0	0	0	0	0	0	0	0
ShSmInvertFeeder	0.009	0.023	0	0	0	0	0	0.036	0	0	0	0	0.205	0	0	0	0.005	0	0	0	0	0	0	0	0
ShSmPredator	0	0	0	0	0	0	0	0.171	0	0	0	0	0.015	0	0	0	0.005	0	0	0	0	0	0	0	0
ShMedInvertFeeder	0	0	0	0	0	0	0	0.099	0	0	0	0	0	0	0	0	0.003	0	0	0	0	0	0	0	0
ShMedPredator	0	0	0	0	0	0	0	0.05	0	0	0	0	0	0	0	0	0.003	0	0	0	0	0	0	0	0
ShLInvertFeeder	0	0	0	0	0	0	0	0	0	0	0	0	0	0	0	0	0	0	0	0	0	0	0	0	0
ShLPredator	0	0	0	0	0	0	0	0	0	0	0	0	0	0	0	0	0.008	0	0	0	0	0	0	0	0
Blue-eye trevalla	0	0	0	0	0	0	0	0	0	0	0	0	0	0	0	0	0	0	0	0	0	0	0	0	0
Blue grenadier	0.041	0	0	0	0	0	0	0	0	0	0	0	0	0	0	0	0	0	0	0	0	0	0	0	0
SlopeOceanPerch	0	0.005	0	0	0	0	0	0.015	0	0	0	0	0	0	0	0	0	0	0	0	0	0	0	0	0
Deepsea Cod	0	0	0	0	0	0	0	0.01	0	0	0	0	0	0	0	0	0	0	0	0	0	0	0	0	0
Oreos	0	0	0	0	0	0	0	0.005	0	0	0	0	0	0	0	0	0	0	0	0	0	0	0	0	0
SlopeSmInvertFeeder	0.018	0.004	0.05	0	0	0	0	0	0	0.16	0	0	0	0	0	0	0	0	0	0	0	0	0	0	0

Group	Blue grenadier	SlopeOceanPerch	Deepsea Cod	Oreos	SlopeSmInvertFeeder	SlopeSmPredator	SlopeMInverFeeder	SlopeMPredator	SlopeLInvertFeeder	SlopeLPredator	PelSmInvertFeeder	PelMInvertFeeder	PelMPredator	PelLInvertFeeder	PelLPredator	Mesopelagics	Squid	PelagicPrawns	Macrobenthos	Megabenthos	Polychaeta	Gelatinous nekton	Euphausids	L zooplankton	Sm zooplankton
SlopeSmPredator	0	0	0.05	0	0	0	0	0.011	0	0.02	0	0	0	0	0	0	0	0	0	0	0	0	0	0	0
SlopeMInverFeeder	0.118	0.206	0	0	0	0	0.006	0.02	0	0.2	0	0	0	0	0	0	0	0	0	0	0	0	0	0	0
SlopeMPredator	0	0	0	0	0	0	0	0	0	0.025	0	0	0	0	0	0	0.002	0	0	0	0	0	0	0	0
SlopeLInvertFeeder	0	0	0	0	0	0	0	0	0	0	0	0	0	0	0	0	0	0	0	0	0	0	0	0	0
SlopeLPredator	0.008	0	0	0	0	0	0	0	0	0	0	0	0	0	0	0	0	0	0	0	0	0	0	0	0
PelSmInvertFeeder	0	0	0	0	0	0	0	0	0	0	0	0.05	0.2	0	0.48	0	0.16	0	0	0	0	0	0	0	0
PelMInvertFeeder	0	0	0	0	0	0	0	0	0	0	0	0	0	0	0.02	0	0.002	0	0	0	0	0	0	0	0
PelMPredator	0	0	0	0	0	0	0	0	0	0	0	0	0	0	0	0	0.002	0	0	0	0	0	0	0	0
PelLInvertFeeder	0	0	0	0	0	0	0	0	0	0	0	0	0	0	0	0	0	0	0	0	0	0	0	0	0
PelLPredator	0	0	0	0	0	0	0	0	0	0	0	0	0	0	0	0	0	0	0	0	0	0	0	0	0
Mesopelagics	0.717	0.068	0	0.381	0	0.534	0.251	0.189	0.061	0.025	0.024	0.06	0.42	0.152	0	0.07	0.215	0	0	0	0	0	0	0	0
Squid	0.03	0.001	0	0	0	0	0.012	0	0.003	0.35	0.003	0	0.01	0.02	0.3	0.002	0.11	0	0	0	0	0	0	0	0
PelagicPrawns	0.009	0	0	0	0.037	0.006	0.034	0.012	0	0.05	0	0	0	0	0	0	0.15	0	0	0	0	0	0	0	0
Macrobenthos	0.002	0.078	0.7	0.09	0.189	0.052	0.002	0	0.404	0	0.003	0.1	0	0.01	0	0	0	0	0	0.5	0.05	0	0	0	0
Megabenthos	0.007	0.176	0.2	0.03	0.252	0.136	0.1	0	0.525	0.15	0	0	0	0	0	0	0.2	0	0	0	0	0	0	0	0
Polychaeta	0	0.008	0	0	0.367	0.137	0.014	0.039	0.004	0	0	0	0	0	0	0	0	0	0	0.05	0	0	0	0	0
Gelatinous nekton	0.002	0.258	0	0	0.003	0	0	0.001	0	0	0	0.05	0	0.009	0	0	0	0	0	0	0	0	0	0	0
Euphausids	0	0.004	0	0	0.051	0.008	0	0	0	0	0	0.3	0	0	0	0.785	0.07	0	0	0	0	0	0	0	0
L zooplankton	0.009	0.041	0	0.399	0.008	0.045	0.581	0	0.003	0	0.239	0.3	0.05	0.762	0.2	0	0.05	0	0.1	0	0	0	0	0	0
Sm zooplankton	0.001	0.001	0	0.1	0.092	0.083	0.001	0	0	0	0.707	0.14	0.05	0.047	0	0.143	0	0.2	0.4	0.4	0.05	0.9	0.2	0.2	0
Primary producers	0	0	0	0	0	0	0	0	0	0	0.024	0	0	0	0	0	0	0.8	0	0	0.5	0.1	0.8	0.8	1
Benthic producer	0	0	0	0	0	0	0	0	0	0	0	0	0	0	0	0	0	0	0	0	0	0	0	0	0
Detritus	0	0	0	0	0	0	0	0	0	0	0	0	0	0	0	0	0	0	0.5	0.05	0.4	0	0	0	0
Discards	0	0	0	0	0	0	0	0	0	0	0	0	0	0	0	0	0	0	0	0	0	0	0	0	0
Imports	0	0	0	0	0	0	0	0	0	0	0	0	0	0	0	0	0	0	0	0	0	0	0	0	0

Table S27**:** Diet matrix for the SE Australian Ecopath model – Variant 2

**Group**	Toothed whale	Baleen whale	Seal	Seabirds	Penguins	Tuna/billfish	Pelagic sharks	Demersal sharks	Rays	Warehous	Redbait	Redfish	Ling	Dories	Jack mackerel	Jackass morwong	Flathead	Gemfish	ShOceanPerch	Chinaman leatherjacket	Cucumberfish	Whiting	Cardinal	ShSmInvertFeeder	ShSmPredator	ShMedInvertFeeder	ShMedPredator	ShLInvertFeeder	ShLPredator	Blue-eye trevalla
Toothed whale	0.001	0	0	0	0	0	0	0	0	0	0	0	0	0	0	0	0	0	0	0	0	0	0	0	0	0	0	0	0	0
Baleen whale	0	0	0	0	0	0	0	0	0	0	0	0	0	0	0	0	0	0	0	0	0	0	0	0	0	0	0	0	0	0
Seal	0.002	0	0	0	0	0	0.025	0	0	0	0	0	0	0	0	0	0	0	0	0	0	0	0	0	0	0	0	0	0	0
Seabirds	0.005	0	0	0	0	0	0.009	0	0	0	0	0	0	0	0	0	0	0	0	0	0	0	0	0	0	0	0	0	0	0
Penguins	0.003	0	0	0	0	0	0.009	0	0	0	0	0	0	0	0	0	0	0	0	0	0	0	0	0	0	0	0	0	0	0
Tuna/billfish	0.002	0	0	0	0	0	0.025	0	0	0	0	0	0	0	0	0	0	0	0	0	0	0	0	0	0	0	0	0	0	0
Pelagic sharks	0.002	0	0	0	0	0	0.006	0	0	0	0	0	0	0	0	0	0	0	0	0	0	0	0	0	0	0	0	0	0	0
Demersal sharks	0	0	0	0	0	0	0.134	0	0	0	0	0	0	0	0	0	0	0	0	0	0	0	0	0	0	0	0.01	0	0.03	0
Rays	0	0	0	0	0	0	0.134	0.006	0	0	0	0	0	0	0	0	0	0	0	0	0	0	0	0	0	0	0.01	0	0.05	0
Warehous	0	0	0.045	0	0.1	0	0.048	0	0	0	0	0	0	0	0	0	0	0	0	0	0	0	0	0	0	0	0	0	0.01	0
Redbait	0	0.01	0.251	0.01	0	0.159	0	0.008	0	0	0	0	0	0.096	0	0	0	0	0	0	0	0	0	0	0	0	0.05	0	0.017	0
Redfish	0	0	0.024	0	0	0	0.009	0.023	0	0	0	0	0	0.039	0	0	0	0	0	0	0	0	0	0	0	0	0	0	0.002	0
Ling	0	0	0.012	0	0	0	0.001	0.001	0	0	0	0	0	0	0	0	0.014	0	0	0	0	0	0	0	0	0	0	0	0	0
Dories	0	0	0.01	0	0	0	0.008	0.001	0	0	0	0	0	0	0	0	0.01	0	0	0	0	0	0	0	0	0	0.03	0	0	0
Jack mackerel	0.078	0	0.3	0	0	0.228	0.045	0.21	0	0	0	0	0	0.145	0	0	0.005	0	0	0	0	0	0	0	0	0	0.06	0	0.032	0
Jackass morwong	0	0	0.016	0	0	0	0	0.011	0	0	0	0	0	0	0	0	0	0	0	0	0	0	0	0	0	0	0.02	0	0.009	0
Flathead	0	0	0.035	0	0	0	0	0.007	0	0	0	0	0	0	0	0	0	0	0	0	0	0	0	0	0	0	0.01	0	0	0
Gemfish	0	0	0.015	0	0	0	0.008	0	0	0	0	0	0	0	0	0	0	0	0	0	0	0	0	0	0	0	0	0	0	0
ShOceanPerch	0	0	0.005	0	0	0	0	0.001	0	0	0	0	0	0.082	0	0	0.002	0	0	0	0	0	0	0	0	0	0	0	0.002	0
Chinaman leatherjacket	0	0	0	0	0	0	0	0	0	0	0	0	0	0	0	0	0	0	0	0	0	0	0	0	0	0	0	0	0	0
Cucumberfish	0	0	0	0	0	0	0	0.007	0	0	0	0.01	0.08	0.132	0	0	0.088	0	0.08	0	0	0	0	0	0.1	0.04	0.04	0	0.04	0
Whiting	0	0	0.01	0	0	0	0	0.003	0.02	0	0	0	0	0.006	0	0	0.1	0	0	0	0	0	0	0	0.1	0	0.002	0	0.083	0
Cardinal	0	0	0.008	0	0	0	0	0.037	0	0.018	0	0.253	0.192	0.14	0.006	0.103	0.05	0.534	0.124	0	0	0	0	0	0.101	0	0.025	0	0.181	0
ShSmInvertFeeder	0	0	0.092	0	0	0	0	0.03	0.022	0	0	0.01	0.477	0.124	0.001	0.007	0.085	0	0.083	0.1	0.01	0.005	0	0.002	0.151	0.064	0.251	0	0.014	0
ShSmPredator	0	0	0.051	0	0	0	0	0	0	0	0	0.01	0	0	0	0	0	0	0	0.1	0.005	0	0	0.001	0	0	0.012	0	0	0
ShMedInvertFeeder	0	0	0	0.005	0	0	0	0.013	0	0	0	0.001	0	0.172	0	0.007	0.025	0	0.082	0	0.001	0.005	0	0.001	0.005	0.001	0.025	0	0.01	0
ShMedPredator	0	0	0.04	0.005	0	0	0	0.005	0	0	0	0	0	0.017	0	0.002	0.005	0	0	0	0	0	0	0	0	0	0	0	0	0
ShLInvertFeeder	0	0	0	0	0.1	0	0	0	0	0	0	0	0	0	0	0	0	0	0	0	0	0	0	0	0	0	0	0	0	0
ShLPredator	0	0	0	0	0	0	0	0.008	0	0	0	0	0	0	0	0	0.01	0	0.007	0	0	0	0	0	0	0	0.02	0	0	0
Blue-eye trevalla	0.009	0	0	0	0	0	0	0.001	0	0	0	0	0	0	0	0	0	0	0	0	0	0	0	0	0	0	0	0	0	0
Blue grenadier	0	0	0	0	0	0	0	0	0	0	0	0	0	0	0	0	0	0.329	0	0	0	0	0	0	0	0	0	0	0	0.01
SlopeOceanPerch	0	0	0	0	0	0	0	0	0	0	0	0.001	0	0	0	0	0	0	0	0	0	0	0	0	0	0	0	0	0.002	0
Deepsea Cod	0	0	0	0	0	0	0	0	0	0	0	0	0	0	0	0	0	0	0	0	0	0	0	0	0	0	0	0	0	0
Oreos	0	0	0	0	0	0	0	0	0	0	0	0	0	0	0	0	0	0	0	0	0	0	0	0	0	0	0	0	0	0
SlopeSmInvertFeeder	0	0.02	0	0	0	0	0	0	0.001	0	0	0	0	0	0	0	0	0	0	0.1	0	0	0	0	0	0	0	0	0	0.01

Group	Toothed whale	Baleen whale	Seal	Seabirds	Penguins	Tuna/billfish	Pelagic sharks	Demersal sharks	Rays	Warehous	Redbait	Redfish	Ling	Dories	Jack mackerel	Jackass morwong	Flathead	Gemfish	ShOceanPerch	Chinaman leatherjacket	Cucumberfish	Whiting	Cardinal	ShSmInvertFeeder	ShSmPredator	ShMedInvertFeeder	ShMedPredator	ShLInvertFeeder	ShLPredator	Blue-eye trevalla
SlopeSmPredator	0	0.01	0	0	0	0	0	0.006	0.004	0	0	0.001	0.01	0	0	0	0	0	0.014	0.1	0.001	0	0	0	0	0	0	0	0	0.01
SlopeMInverFeeder	0	0	0	0	0	0	0	0.022	0.01	0	0	0.004	0	0	0	0	0	0.134	0	0	0	0	0	0	0	0	0.003	0	0	0.01
SlopeMPredator	0	0	0	0	0.1	0	0	0.006	0	0	0	0	0	0	0	0	0	0	0	0	0	0	0	0	0	0	0	0	0	0
SlopeLInvertFeeder	0	0	0	0	0	0	0	0.005	0	0	0	0.014	0.056	0	0	0	0.105	0	0.068	0	0	0	0	0	0.005	0	0	0	0	0
SlopeLPredator	0	0	0	0	0	0	0	0	0	0	0	0	0	0	0	0	0	0	0	0	0	0	0	0	0	0	0	0	0	0
PelSmInvertFeeder	0.098	0.04	0	0.005	0.45	0.009	0	0.001	0	0	0	0	0	0	0	0	0.133	0	0	0	0	0	0	0	0	0	0	0	0.28	0
PelMInvertFeeder	0.049	0	0	0	0	0	0.01	0.005	0	0	0	0	0	0	0	0	0	0	0	0	0	0	0	0	0	0	0	0	0	0
PelMPredator	0.01	0	0	0	0	0.02	0.03	0.01	0	0	0	0	0	0	0	0	0	0	0	0	0	0	0	0	0	0	0	0	0	0
PelLInvertFeeder	0.025	0	0	0	0	0	0.045	0	0	0	0	0	0	0	0	0	0	0	0	0	0	0	0	0	0	0	0	0	0	0
PelLPredator	0	0	0	0	0	0	0.039	0	0	0	0	0	0	0	0	0	0	0	0	0	0	0	0	0	0	0	0	0	0	0
Mesopelagics	0.098	0.02	0	0.135	0	0.005	0	0.001	0	0.002	0.065	0.068	0	0	0.513	0.001	0.038	0.001	0	0	0	0	0.837	0	0	0	0	0	0.12	0.05
Squid	0.149	0	0.055	0.27	0.1	0.07	0.1	0.222	0	0.246	0	0.049	0	0.007	0	0.006	0.003	0.002	0.021	0	0.018	0.007	0	0.002	0	0.003	0.1	0	0.025	0.2
PelagicPrawns	0	0	0	0	0.05	0	0	0.029	0.084	0.001	0	0.025	0.011	0.012	0	0.017	0.141	0	0.024	0	0.006	0	0.01	0.027	0	0	0.05	0	0.075	0.01
Macrobenthos	0	0	0	0.025	0	0	0.045	0.005	0.114	0.021	0.200	0.001	0	0.005	0.009	0.16	0.009	0	0.029	0.1	0.139	0.48	0	0.215	0.1	0.1	0.05	0.36	0	0
Megabenthos	0	0	0	0	0.05	0	0.018	0.256	0.342	0.152	0	0.052	0.103	0.008	0.011	0.186	0.05	0	0.176	0.1	0.117	0.012	0.013	0.197	0.1	0.575	0.151	0.28	0.008	0
Polychaeta	0	0	0	0	0	0	0	0.003	0.208	0.140	0	0.001	0	0	0.001	0.273	0.002	0	0.074	0	0.05	0.464	0	0.144	0	0.05	0.002	0.21	0	0
Gelatinous nekton	0	0	0	0.005	0	0	0	0.001	0	0.901	0.212	0.001	0	0	0.011	0.001	0.024	0	0.14	0.4	0.073	0	0	0.003	0.056	0	0.001	0	0.001	0.7
Euphausids	0	0.1	0	0	0.05	0.01	0	0.001	0.001	0.005	0.150	0.09	0.03	0.005	0.094	0.081	0.001	0	0.001	0	0.285	0.016	0.052	0.016	0.005	0	0	0	0.001	0
L zooplankton	0	0	0	0	0	0	0	0.002	0.058	0.001	0.116	0.284	0.036	0.006	0.164	0.08	0.049	0	0.04	0	0.212	0.005	0.084	0.102	0.194	0.153	0.08	0.15	0.004	0
Sm zooplankton	0	0	0	0	0	0	0	0.026	0.126	0.002	0.256	0.126	0.005	0.003	0.19	0.078	0.05	0	0.038	0	0.084	0.006	0.004	0.29	0.082	0.011	0.002	0	0.004	0
Primary producers	0	0	0	0	0	0	0	0	0	0	0	0	0	0	0	0	0	0	0	0	0	0	0	0	0	0	0	0	0	0
Benthic producer	0	0	0	0	0	0	0	0.001	0	0.001	0	0	0	0	0	0	0	0	0	0	0	0	0	0	0.001	0.003	0	0	0	0
Detritus	0	0	0	0	0	0	0	0	0	0	0	0	0	0	0	0	0	0	0	0	0	0	0	0	0	0	0	0	0	0
Discards	0	0	0.03	0.04	0	0	0	0.028	0.01	0	0	0	0	0	0	0	0	0	0	0	0	0	0	0	0	0	0.003	0	0	0
Imports	0.469	0.8	0	0.5	0	0.5	0.25	0	0	0	0	0	0	0	0	0	0	0	0	0	0	0	0	0	0	0	0	0	0	0

Table S27: Diet matrix for the SE Australian Ecopath model – variant 2 – continued

Group	Blue grenadier	SlopeOceanPerch	Deepsea Cod	Oreos	SlopeSmInvertFeeder	SlopeSmPredator	SlopeMInverFeeder	SlopeMPredator	SlopeLInvertFeeder	SlopeLPredator	PelSmInvertFeeder	PelMInvertFeeder	PelMPredator	PelLInvertFeeder	PelLPredator	Mesopelagics	Squid	PelagicPrawns	Macrobenthos	Megabenthos	Polychaeta	Gelatinous nekton	Euphausids	L zooplankton	Sm zooplankton
Toothed whale	0	0	0	0	0	0	0	0	0	0	0	0	0	0	0	0	0	0	0	0	0	0	0	0	0
Baleen whale	0	0	0	0	0	0	0	0	0	0	0	0	0	0	0	0	0	0	0	0	0	0	0	0	0
Seal	0	0	0	0	0	0	0	0	0	0	0	0	0	0	0	0	0	0	0	0	0	0	0	0	0
Seabirds	0	0	0	0	0	0	0	0	0	0	0	0	0	0	0	0	0	0	0	0	0	0	0	0	0
Penguins	0	0	0	0	0	0	0	0	0	0	0	0	0	0	0	0	0	0	0	0	0	0	0	0	0
Tuna/billfish	0	0	0	0	0	0	0	0	0	0	0	0	0	0	0	0	0	0	0	0	0	0	0	0	0
Pelagic sharks	0	0	0	0	0	0	0	0	0	0	0	0	0	0	0	0	0	0	0	0	0	0	0	0	0
Demersal sharks	0	0	0	0	0	0	0	0	0	0	0	0	0	0	0	0	0	0	0	0	0	0	0	0	0
Rays	0	0	0	0	0	0	0	0	0	0.01	0	0	0	0	0	0	0	0	0	0	0	0	0	0	0
Warehous	0	0	0	0	0	0	0	0	0	0.01	0	0	0	0	0	0	0	0	0	0	0	0	0	0	0
Redbait	0	0	0	0	0	0	0	0	0	0	0	0	0.05	0	0	0	0.01	0	0	0	0	0	0	0	0
Redfish	0	0	0	0	0	0	0	0	0	0	0	0	0	0	0	0	0.002	0	0	0	0	0	0	0	0
Ling	0	0	0	0	0	0	0	0	0	0	0	0	0	0	0	0	0.001	0	0	0	0	0	0	0	0
Dories	0	0	0	0	0	0	0	0	0	0	0	0	0	0	0	0	0	0	0	0	0	0	0	0	0
Jack mackerel	0	0.06	0	0	0	0	0	0	0	0	0	0	0	0	0	0	0.001	0	0	0	0	0	0	0	0
Jackass morwong	0	0	0	0	0	0	0	0	0	0	0	0	0	0	0	0	0	0	0	0	0	0	0	0	0
Flathead	0	0	0	0	0	0	0	0	0	0	0	0	0	0	0	0	0	0	0	0	0	0	0	0	0
Gemfish	0.016	0.059	0	0	0	0	0	0	0	0	0	0	0	0	0	0	0	0	0	0	0	0	0	0	0
ShOceanPerch	0	0	0	0	0	0	0	0	0	0	0	0	0	0	0	0	0.001	0	0	0	0	0	0	0	0
Chinaman leatherjacket	0	0	0	0	0	0	0	0	0	0	0	0	0	0	0	0	0	0	0	0	0	0	0	0	0
Cucumberfish	0	0.009	0	0	0	0	0	0.094	0	0	0	0	0	0	0	0	0	0	0	0	0	0	0	0	0
Whiting	0	0	0	0	0	0	0	0	0	0	0	0	0	0	0	0	0.001	0	0	0	0	0	0	0	0
Cardinal	0.015	0.001	0	0	0	0	0	0.249	0	0	0	0	0	0	0	0	0	0	0	0	0	0	0	0	0
ShSmInvertFeeder	0.009	0.023	0	0	0	0	0	0.036	0	0	0	0	0.205	0	0	0	0.005	0	0	0	0	0	0	0	0
ShSmPredator	0	0	0	0	0	0	0	0.171	0	0	0	0	0.015	0	0	0	0.005	0	0	0	0	0	0	0	0
ShMedInvertFeeder	0	0	0	0	0	0	0	0.099	0	0	0	0	0	0	0	0	0.003	0	0	0	0	0	0	0	0
ShMedPredator	0	0	0	0	0	0	0	0.05	0	0	0	0	0	0	0	0	0.003	0	0	0	0	0	0	0	0
ShLInvertFeeder	0	0	0	0	0	0	0	0	0	0	0	0	0	0	0	0	0	0	0	0	0	0	0	0	0
ShLPredator	0	0	0	0	0	0	0	0	0	0	0	0	0	0	0	0	0.008	0	0	0	0	0	0	0	0
Blue-eye trevalla	0	0	0	0	0	0	0	0	0	0	0	0	0	0	0	0	0	0	0	0	0	0	0	0	0
Blue grenadier	0.041	0	0	0	0	0	0	0	0	0	0	0	0	0	0	0	0	0	0	0	0	0	0	0	0
SlopeOceanPerch	0	0.005	0	0	0	0	0	0.015	0	0	0	0	0	0	0	0	0	0	0	0	0	0	0	0	0
Deepsea Cod	0	0	0	0	0	0	0	0.01	0	0	0	0	0	0	0	0	0	0	0	0	0	0	0	0	0
Oreos	0	0	0	0	0	0	0	0.005	0	0	0	0	0	0	0	0	0	0	0	0	0	0	0	0	0
SlopeSmInvertFeeder	0.018	0.004	0.05	0	0	0	0	0	0	0.16	0	0	0	0	0	0	0	0	0	0	0	0	0	0	0

Group	Blue grenadier	SlopeOceanPerch	Deepsea Cod	Oreos	SlopeSmInvertFeeder	SlopeSmPredator	SlopeMInverFeeder	SlopeMPredator	SlopeLInvertFeeder	SlopeLPredator	PelSmInvertFeeder	PelMInvertFeeder	PelMPredator	PelLInvertFeeder	PelLPredator	Mesopelagics	Squid	PelagicPrawns	Macrobenthos	Megabenthos	Polychaeta	Gelatinous nekton	Euphausids	L zooplankton	Sm zooplankton
SlopeSmPredator	0	0	0.05	0	0	0	0	0.011	0	0.02	0	0	0	0	0	0	0	0	0	0	0	0	0	0	0
SlopeMInverFeeder	0.118	0.206	0	0	0	0	0.006	0.02	0	0.2	0	0	0	0	0	0	0	0	0	0	0	0	0	0	0
SlopeMPredator	0	0	0	0	0	0	0	0	0	0.025	0	0	0	0	0	0	0.002	0	0	0	0	0	0	0	0
SlopeLInvertFeeder	0	0	0	0	0	0	0	0	0	0	0	0	0	0	0	0	0	0	0	0	0	0	0	0	0
SlopeLPredator	0.008	0	0	0	0	0	0	0	0	0	0	0	0	0	0	0	0	0	0	0	0	0	0	0	0
PelSmInvertFeeder	0	0	0	0	0	0	0	0	0	0	0	0.05	0.2	0	0.48	0	0.16	0	0	0	0	0	0	0	0
PelMInvertFeeder	0	0	0	0	0	0	0	0	0	0	0	0	0	0	0.02	0	0.002	0	0	0	0	0	0	0	0
PelMPredator	0	0	0	0	0	0	0	0	0	0	0	0	0	0	0	0	0.002	0	0	0	0	0	0	0	0
PelLInvertFeeder	0	0	0	0	0	0	0	0	0	0	0	0	0	0	0	0	0	0	0	0	0	0	0	0	0
PelLPredator	0	0	0	0	0	0	0	0	0	0	0	0	0	0	0	0	0	0	0	0	0	0	0	0	0
Mesopelagics	0.717	0.068	0	0.381	0	0.534	0.251	0.189	0.061	0.025	0.024	0.06	0.42	0.152	0	0.07	0.215	0	0	0	0	0	0	0	0
Squid	0.03	0.001	0	0	0	0	0.012	0	0.003	0.35	0.003	0	0.01	0.02	0.3	0.002	0.11	0	0	0	0	0	0	0	0
PelagicPrawns	0.009	0	0	0	0.037	0.006	0.034	0.012	0	0.05	0	0	0	0	0	0	0.15	0	0	0	0	0	0	0	0
Macrobenthos	0.002	0.078	0.7	0.09	0.189	0.052	0.002	0	0.404	0	0.003	0.1	0	0.01	0	0	0	0	0	0.5	0.05	0	0	0	0
Megabenthos	0.007	0.176	0.2	0.03	0.252	0.136	0.1	0	0.521	0.15	0	0	0	0	0	0	0.2	0	0	0	0	0	0	0	0
Polychaeta	0	0.008	0	0	0.367	0.137	0.014	0.039	0.004	0	0	0	0	0	0	0	0	0	0	0.05	0	0	0	0	0
Gelatinous nekton	0.002	0.258	0	0	0.003	0	0	0.001	0	0	0	0.05	0	0.009	0	0	0	0	0	0	0	0	0	0	0
Euphausids	0	0.004	0	0	0.051	0.008	0	0	0	0	0	0.3	0	0	0	0.785	0.07	0	0	0	0	0	0	0	0
L zooplankton	0.009	0.041	0	0.399	0.008	0.045	0.58	0	0.003	0	0.239	0.3	0.05	0.762	0.2	0	0.05	0	0.1	0	0	0	0	0	0
Sm zooplankton	0.001	0.001	0	0.1	0.092	0.083	0.001	0.001	0.001	0	0.707	0.14	0.05	0.047	0	0.143	0	0.2	0.4	0.4	0.05	0.9	0.2	0.2	0
Primary producers	0	0	0	0	0	0	0	0	0	0	0.024	0	0	0	0	0	0	0.8	0	0	0.5	0.1	0.8	0.8	1
Benthic producer	0	0	0	0	0	0	0	0	0	0	0	0	0	0	0	0	0	0	0	0	0	0	0	0	0
Detritus	0	0	0	0	0	0	0	0	0	0	0	0	0	0	0	0	0	0	0.5	0.05	0.4	0	0	0	0
Discards	0	0	0	0	0	0	0	0	0	0	0	0	0	0	0	0	0	0	0	0	0	0	0	0	0
Imports	0	0	0	0	0	0	0	0	0	0	0	0	0	0	0	0	0	0	0	0	0	0	0	0	0

Table S28: Landed catch for the SE Australian Ecopath model (units tkm^{-2}year^{-1})

Group	Trawl	Non-trawl	Line	NSW trawl	Vic trawl	Scallop	Squid	Trap	Danish Seine	Tuna Longline	Recreational
Toothed whale	0	0	0	0	0	0	0	0	0	0	0
Baleen whale	0	0	0	0	0	0	0	0	0	0	0
Seal	0	0	0	0	0	0	0	0	0	0	0
Seabirds	0	0	0	0	0	0	0	0	0	0	0
Penguins	0	0	0	0	0	0	0	0	0	0	0
Tuna/billfish	0	4.97E-04	1.45E-02	1.00E-07	0	0	0	0	0	6.18E-03	8.49E-03
Pelagic sharks	0	0	8.58E-05	7.00E-07	0	0	0	0	0	2.37E-04	1.31E-04
Demersal sharks	8.04E-04	5.61E-03	1.25E-04	8.14E-04	1.23E-04	0	0	0	5.43E-04	1.20E-05	1.29E-03
Rays	1.21E-03	5.00E-07	3.60E-06	1.72E-04	3.00E-05	0	0	0	2.40E-05	0	1.56E-04
Warehous	5.70E-02	7.69E-03	0	2.21E-05	1.28E-03	0	0	0	1.08E-04	0	3.60E-04
Redbait	1.00E-05	0	0	0	0	0	0	0	0	0	0
Redfish	2.08E-02	3.00E-07	0	8.91E-05	1.59E-04	0	0	0	3.00E-07	0	3.19E-05
Ling	1.47E-02	6.75E-04	5.38E-04	1.50E-05	9.52E-04	0	0	0	5.70E-05	0	0
Dories	7.49E-03	1.56E-05	0	3.26E-04	3.53E-04	0	0	0	3.14E-04	0	0
Jack mackerel	8.28E-04	7.76E-03	0	3.64E-05	2.70E-05	0	0	0	3.40E-05	0	6.67E-05
Jackass morwong	1.58E-02	2.60E-06	3.00E-07	2.75E-04	0	0	0	0	2.78E-04	0	0
Flathead	3.89E-02	8.90E-06	2.00E-07	1.89E-03	8.76E-04	0	0	0	1.96E-02	0	6.04E-03
Gemfish	2.02E-03	6.70E-06	1.24E-05	0	4.10E-05	0	0	0	0	0	0
ShOceanPerch	3.75E-03	0	9.86E-05	7.38E-05	1.53E-04	0	0	0	3.00E-06	0	0
Chinaman leatherjacket	4.88E-04	0	0	0	0	0	0	0	2.00E-05	0	0
Cucumberfish	0	0	0	0	0	0	0	0	0	0	0
Whiting	8.20E-06	3.00E-07	0	4.75E-05	6.00E-06	0	0	0	1.98E-02	0	0
Cardinal	0	0	0	0	0	0	0	0	0	0	0
ShSmInvertFeeder	5.32E-05	3.89E-05	6.12E-05	1.78E-05	2.00E-07	0	0	3.30E-06	1.68E-04	0	2.53E-04
ShSmPredator	3.75E-04	0	5.00E-07	5.70E-06	0	0	0	0	3.00E-06	0	2.08E-05
ShMedInvertFeeder	1.17E-03	7.26E-04	8.82E-05	5.42E-05	8.55E-04	0	0	0	2.60E-04	0	1.50E-03
ShMedPredator	1.36E-03	7.69E-04	2.54E-03	4.39E-04	8.80E-05	0	0	4.05E-04	6.91E-04	0	1.88E-04
ShLInvertFeeder	0	5.60E-06	3.60E-06	3.25E-05	0	0	0	0	3.30E-05	0	1.82E-04
ShLPredator	1.26E-02	5.54E-04	2.09E-04	8.76E-03	2.46E-04	0	0	5.00E-07	4.80E-05	2.23E-04	2.82E-03
Blue-eye trevalla	3.20E-04	5.72E-04	6.79E-04	0	5.00E-06	0	0	0	3.00E-07	0	0
Blue grenadier	4.85E-03	2.85E-05	1.00E-07	0	2.09E-04	0	0	0	0	0	0
SlopeOceanPerch	4.21E-03	0	0	0	0	0	0	0	0	0	0
Deepsea Cod	3.20E-04	0	0	0	9.00E-06	0	0	0	0	0	0
Oreos	6.64E-04	0	0	0	0	0	0	0	0	0	0
SlopeSmInvertFeeder	7.23E-05	0	0	0	0	0	0	0	0	0	0

Group	Trawl	Non-trawl	Line	NSW trawl	Vic trawl	Scallop	Squid	Trap	Danish Seine	Tuna Longline	Recreational
SlopeSmPredator	2.07E-04	0	0	0	0	0	0	0	0	0	0
SlopeMInverFeeder	1.70E-06	2.00E-07	0	0	0	0	0	0	0	0	0
SlopeMPredator	3.06E-04	2.73E-05	0	0	0	0	0	0	0	0	5.42E-07
SlopeLInvertFeeder	0	0	0	0	0	0	0	0	0	0	0
SlopeLPredator	1.93E-03	2.61E-05	1.39E-04	1.70E-06	0	0	0	0	0	0	0
PelSmInvertFeeder	0	5.76E-02	1.73E-05	0	0	0	0	0	0	0	3.39E-05
PelMInvertFeeder	7.23E-05	3.50E-05	7.80E-06	2.30E-06	0	0	0	0	1.00E-07	0	2.70E-04
PelMPredator	4.80E-06	1.63E-02	2.60E-04	1.55E-04	0	0	0	0	1.60E-05	3.30E-05	4.74E-03
PelLInvertFeeder	0	0	0	0	0	0	0	0	0	0	0
PelLPredator	0	4.90E-06	1.44E-05	6.00E-07	0	0	0	0	1.00E-07	2.20E-05	2.02E-03
Mesopelagics	0	0	0	0	0	0	0	0	0	0	0
Squid	2.81E-03	1.14E-05	8.00E-07	2.51E-04	3.12E-04	0	4.00E-06	8.60E-06	0	0	2.29E-04
PelagicPrawns	0	2.20E-06	0	1.60E-04	3.60E-05	0	0	0	0	0	0
Macrobenthos	0	0	0	3.84E-04	3.07E-04	0	0	0	0	0	0
Megabenthos	1.13E-02	1.50E-06	0	8.22E-04	1.08E-03	4.96E-03	0	1.31E-04	0	0	0
Polychaeta	0	0	0	0	0	0	0	0	0	0	0
Gelatinous nekton	0	0	0	0	0	0	0	0	0	0	0
Euphausids	0	0	0	0	0	0	0	0	0	0	0
L zooplankton	0	0	0	0	0	0	0	0	0	0	0
Sm zooplankton	0	0	0	0	0	0	0	0	0	0	0
Primary producers	0	0	0	0	0	0	0	0	0	0	0
Benthic producer	0	0	0	0	0	0	0	0	0	0	0
Detritus	0	0	0	0	0	0	0	0	0	0	0
Discards	0	0	0	0	0	0	0	0	0	0	0
Imports	0	0	0	0	0	0	0	0	0	0	0

Table S29: Discards for the SE Australian Ecopath model (units tkm^{-2}year^{-1})

Group	Trawl	Non-trawl	Line	NSW trawl	Vic trawl	Scallop	Squid	Trap	Danish Seine	Tuna Longline	Recreational
Toothed whale	0	0	0	0	0	0	0	0	0	0	0
Baleen whale	0	0	0	0	0	0	0	0	0	0	0
Seal	4.45E-04	0	0	3.27E-05	1.57E-05	0	0	0	0	0	0
Seabirds	0	0	0	0	0	0	0	0	0	0	0
Penguins	0	0	0	0	0	0	0	0	0	0	0
Tuna/billfish	0	5.00E-06	1.45E-04	0	0	0	0	0	0	1.23E-05	0
Pelagic sharks	0	0	9.00E-07	0	0	0	0	0	0	4.00E-09	0
Demersal sharks	4.97E-03	5.61E-05	1.30E-06	8.15E-04	1.23E-04	0	0	0	4.95E-03	1.00E-07	0
Rays	3.05E-03	5.00E-09	4.00E-08	1.73E-04	3.00E-05	0	0	0	8.46E-03	0	0
Warehous	1.69E-03	7.69E-05	0	2.33E-05	1.36E-03	0	0	0	5.97E-05	0	0
Redbait	0	0	0	0	0	0	0	0	0	0	0
Redfish	2.00E-02	3.00E-09	0	9.10E-05	1.62E-04	0	0	0	1.20E-06	0	0
Ling	1.67E-04	6.80E-06	5.40E-06	1.52E-05	9.66E-04	0	0	0	2.11E-05	0	0
Dories	3.39E-03	2.00E-07	0	3.28E-04	3.56E-04	0	0	0	7.76E-05	0	0
Jack mackerel	1.31E-02	7.76E-05	0	3.64E-05	2.66E-05	0	0	0	2.94E-05	0	0
Jackass morwong	4.24E-04	3.00E-08	3.00E-09	2.79E-04	1.50E-05	0	0	0	2.80E-06	0	0
Flathead	7.21E-04	1.00E-07	2.00E-09	1.97E-03	9.11E-04	0	0	0	5.00E-04	0	0
Gemfish	1.59E-03	1.00E-07	1.00E-07	1.16E-04	4.10E-05	0	0	0	1.01E-05	0	0
ShOceanPerch	3.79E-03	0	1.00E-06	7.41E-05	1.54E-04	0	0	0	2.90E-06	0	0
Chinaman leatherjacket	1.12E-05	0	0	8.00E-07	4.00E-07	0	0	0	0	0	0
Cucumberfish	1.08E-03	0	0	7.93E-05	3.82E-05	0	0	0	8.10E-04	0	0
Whiting	5.00E-07	3.00E-09	0	4.75E-05	5.90E-06	0	0	0	9.91E-04	0	0
Cardinal	3.60E-05	0	0	3.21E-04	1.14E-05	0	0	0	0	0	0
ShSmInvertFeeder	1.47E-02	4.00E-07	6.00E-07	1.78E-05	2.00E-07	0	0	3.00E-08	1.19E-02	0	0
ShSmPredator	3.77E-04	0	1.00E-08	5.70E-06	1.33E-05	0	0	0	1.86E-05	0	0
ShMedInvertFeeder	9.10E-03	7.30E-06	9.00E-07	5.43E-05	8.56E-04	0	0	0	9.71E-03	0	0
ShMedPredator	1.68E-04	7.70E-06	2.54E-05	4.40E-04	8.76E-05	0	0	4.00E-06	6.02E-05	0	0
ShLInvertFeeder	0	1.00E-07	3.60E-08	0	0	0	0	0	0	0	0
ShLPredator	1.42E-02	5.50E-06	2.10E-06	8.87E-03	2.49E-04	0	0	0	3.20E-06	0	0
Blue-eye trevalla	0	5.70E-06	6.80E-06	0	0	0	0	0	0	0	0
Blue grenadier	4.80E-06	3.00E-07	1.00E-09	0	2.10E-04	0	0	0	0	0	0
SlopeOceanPerch	2.36E-04	0	0	0	0	0	0	0	0	0	0
Deepsea Cod	3.20E-05	0	0	0	0	0	0	0	0	0	0
Oreos	2.30E-06	0	0	0	0	0	0	0	0	0	0
SlopeSmInvertFeeder	8.11E-05	0	0	0	0	0	0	0	2.50E-06	0	0

Group	Trawl	Non-trawl	Line	NSW trawl	Vic trawl	Scallop	Squid	Trap	Danish Seine	Tuna Longline	Recreational
SlopeSmPredator	1.31E-03	0	0	0	0	0	0	0	0	0	0
SlopeMInverFeeder	8.12E-03	2.00E-09	0	0	0	0	0	0	0	0	0
SlopeMPredator	2.27E-04	3.00E-07	0	0	0	0	0	0	0	0	0
SlopeLInvertFeeder	1.00E-07	0	0	0	0	0	0	0	0	0	0
SlopeLPredator	1.84E-03	3.00E-07	1.40E-06	1.70E-06	0	0	0	0	8.00E-07	0	0
PelSmInvertFeeder	0	5.76E-04	2.00E-07	0	0	0	0	0	0	0	0
PelMInvertFeeder	9.00E-07	4.00E-07	1.00E-07	2.30E-06	0	0	0	0	1.70E-06	0	0
PelMPredator	8.70E-06	1.63E-04	2.60E-06	1.55E-04	0	0	0	0	0	0	0
PelLInvertFeeder	0	0	0	0	0	0	0	0	0	0	0
PelLPredator	0	5.00E-08	1.00E-07	0	0	0	0	0	0	0	0
Mesopelagics	8.10E-07	0	0	0	0	0	0	0	0	0	0
Squid	0	1.00E-07	1.00E-08	0	0	0	0	1.00E-07	0	0	0
PelagicPrawns	0	2.00E-08	0	0	0	0	0	0	0	0	0
Macrobenthos	1.15E-03	0	0	3.84E-04	3.07E-04	0	0	0	0	0	0
Megabenthos	1.53E-04	1.00E-08	0	8.31E-04	1.09E-03	2.48E-04	0	1.30E-06	0	0	0
Polychaeta	0	0	0	0	0	0	0	0	0	0	0
Gelatinous nekton	1.00E-06	0	0	1.00E-06	9.00E-08	0	0	0	0	0	0
Euphausids	0	0	0	0	0	0	0	0	0	0	0
L zooplankton	0	0	0	0	0	0	0	0	0	0	0
Sm zooplankton	0	0	0	0	0	0	0	0	0	0	0
Primary producers	0	0	0	0	0	0	0	0	0	0	0
Benthic producer	0	0	0	0	0	0	0	0	0	0	0
Detritus	0	0	0	0	0	0	0	0	0	0	0
Discards	0	0	0	0	0	0	0	0	0	0	0
Imports	0	0	0	0	0	0	0	0	0	0	0
